# Supplementary material for: Carbon emission quantification analysis of excavation engineering under road transport conditions
Source: PLoS One. 2024 Dec 31;19(12):e0315765. doi: 10.1371/journal.pone.0315765 (PMC11687867; doi:10.1371/journal.pone.0315765)
Supplement: S1 File — (PDF) [file pone.0315765.s001.pdf]

北京市建设工程计价依据
——预算消耗量标准

城市轨道交通工程

第一册　土建工程

北京市住房和城乡建设委员会

2021 年 9 月

北京市住房和城乡建设委员会文件

京建发〔2021〕201号

北京市住房和城乡建设委员会
关于印发2021年《北京市建设工程计价依据——预算消耗量标准》和《北京市房屋修缮工程计价依据——预算消耗量标准》的通知

各有关单位：

为建立健全我市工程造价市场化形成机制，引导市场主体合理确定工程造价，保障工程质量安全，提高社会投资效益，市住房城乡建设委组织编制了2021年《北京市建设工程计价依据——预算消耗量标准》和2021年《北京市房屋修缮工程计价依据——预算消耗量标准》(以下简称“标准”)，经审查同意，现予发布(查询下载网址：http://zjw.beijing.gov.cn/bjjs/gcjs/zczjxx/jjyjxt/de/index.shtml)。

本标准自2022年1月1日起执行，是国有资金投资项目编制最高投标限价的依据。

本标准由北京市建设工程造价管理总站和北京市房屋修缮工程定额管理站依职责分别负责解释和管理。

北京市住房和城乡建设委员会

2021 年 9 月 7 日

（此件公开发布）

抄送：住房和城乡建设部办公厅，市发展改革委、市财政局、市规划自然资源委、市商务局、市审计局、市国资委、市统计局、市税务局。

北京市住房和城乡建设委员会办公室　　2021 年 9 月 29 日印发

总　说　明

一、2021 年《北京市建设工程计价依据——预算消耗量标准》(以下简称本标准)共分七部分二十八册,包括:

01　房屋建筑与装饰工程预算消耗量标准:房屋建筑与装饰工程共一册;

02　仿古建筑工程预算消耗量标准:仿古建筑工程共一册;

03　通用安装工程预算消耗量标准:机械设备安装工程,热力设备安装工程,静置设备与工艺金属结构制作安装工程,电气设备安装工程,建筑智能化工程,自动化控制仪表安装工程,通风空调工程,工业管道工程,消防工程,给排水、采暖、燃气工程,信息通信设备与线缆安装工程,刷油、防腐蚀、绝热工程共十二册;

04　市政工程预算消耗量标准:通用项目,道路工程,桥涵工程,管网工程,水处理工程共五册;

05　园林绿化工程预算消耗量标准:园林绿化工程共一册;

06　构筑物工程预算消耗量标准:构筑物工程共一册;

07　城市轨道交通工程预算消耗量标准:土建工程,轨道工程,通信工程,信号工程,供电工程,智能工程,机电工程共七册。

二、本标准是在全国和本市有关计价依据的基础上,补充新技术、新工艺、新材料、新设备的应用,根据正常的施工条件、施工质量验收规范、质量评定标准、安全技术操作规程、标准图集和通用图集,施工现场文明安全施工及环境保护的要求,结合本市施工企业的技术装备状况、施工工艺水平、合理的劳动组织与工期安排等进行编制的。

三、本标准适用于北京市行政区域内的工业与民用建筑(含仿古)、市政、园林绿化、轨道交通工程的新

北京市住房和城乡建设委员会

建、扩建;市政改建以及行道新辟栽植和旧园林栽植改造等工程。不适用于房屋修缮工程(含整体更新改造)、轨道交通运营改造工程、临时性工程、山区工程、平原造林工程、道路及园林养护工程等。

四、本标准是完成规定计量单位分项工程所需的人工、材料、施工机械的消耗量标准;是北京市行政区域内国有资金投资工程编制最高投标限价的依据;是编制概算和估算指标的基础。

五、关于人工

1.人工消耗量包括:基本用工、超运距用工、辅助用工和人工幅度差。不分列工种和技术等级,以综合工日表示。

2.本标准的人工每工日按 8 小时工作制计算。

六、关于材料

1.本标准中的材料包括施工中消耗的主要材料、辅助材料和其他材料。

2.材料消耗量包括净用量和损耗量,其中损耗量包括从工地仓库、现场集中堆放地点或现场加工地点至操作或安装地点的运输损耗、施工操作损耗和施工现场堆放损耗。

七、关于机械

1.本标准中的机械按合理配备的常用机械、本市机械化装备程度,并结合工程实际综合确定。

2.本标准的机械台班消耗量是按正常机械施工工效,并考虑机械幅度差综合确定。

3.本标准的机械每台班按 8 小时工作制计算。

八、本标准工作内容除各章节已说明的主要工序外,还包括施工准备、配合质量检验、工种间交叉配合等次要工序。

九、本标准中不包括施工发生的水电。

十、本标准中对工程量计算规则中的计量单位和工程量计算有效位数统一规定如下:

1.“以体积计算”的工程量以“m^3”为计量单位,工程量保留小数点后两位数字。

2.“以面积计算”的工程量以“m^2”为计量单位,工程量保留小数点后两位数字。

北京市住房和城乡建设委员会

3.“以长度计算”的工程量以“m”为计量单位，工程量保留小数点后两位数字。

4.“以质量计算”的工程量以“t”为计量单位，工程量保留小数点后三位数字。

5.“以数量计算”的工程量以“台、块、个、套、件、根、组、系统等”为计量单位，工程量应取整数。

本标准各章计算规则另有具体规定，以其规定为准。

十一、本标准中凡注明“×××以内（下）”者，均包括“×××”本身；注明“×××以外（上）”者，则不包括“×××”本身。

北京市住房和城乡建设委员会

北京市住房和城乡建设委员会

册 说 明

一、城市轨道交通工程预算消耗量标准第一册“土建工程”(以下简称“本标准”)包括土石方工程、桩与支护工程、地基加固工程、主体结构混凝土与钢筋工程、砌筑工程、钢结构工程、盾构工程、防水工程、模板工程、降水工程、监测工程,共 11 章 767 个子目。

二、本标准中的材料材质、型号、规格、强度等级按常用标准编制,设计要求不同时,可调整。

三、本标准中凡注明厚度的子目,设计要求不同时,执行每增减厚度的相应子目。

四、本标准中钢筋子目已包含措施钢筋消耗量,但不含钢筋搭接消耗量,钢筋搭接量应按设计图纸或规范要求计算,图纸未注明搭接数量的,可按以下规则计算:

1.钢筋 ϕ10 以内,按每 12m 长计算 1 个搭接;

2.钢筋 ϕ10 以外,按每 8m 长计算 1 个搭接;

3.采用直螺纹套筒连接的不另计算搭接量。

五、本标准中混凝土工程子目均不含泵送,泵送消耗量应根据实际方案计算。

六、高架和地下车站(区间)土建工程执行《房屋建筑与装饰工程预算消耗量标准》相应子目的,其人工和机械消耗量应乘以系数 1.07。

七、装饰工程应执行《房屋建筑与装饰工程预算消耗量标准》相应子目,且其人工和机械消耗量应乘以系数 1.07。

八、箱涵顶进应执行《市政工程预算消耗量标准》相应子目。

九、车辆基地工程按如下规则划分:

1.车辆基地建筑工程执行《房屋建筑与装饰工程预算消耗量标准》相应子目;

北京市住房和城乡建设委员会

2.车辆基地场内的道路、涵洞、管道、园林绿化等工程，应执行《市政工程预算消耗量标准》和《园林绿化工程预算消耗量标准》等的相应子目。

北京市住房和城乡建设委员会

目　　录

第一章　土石方工程

说明 …………………………………………………………（3）
工程量计算规则 ………………………………………………（6）
第一节 明挖土石方 ……………………………………………（7）
一、土方 ………………………………………………………（7）
二、淤泥流砂 …………………………………………………（9）
三、石方 ………………………………………………………（10）
第二节 明挖大型支撑挖土石方 ………………………………（11）
一、土方 ………………………………………………………（11）
二、淤泥流砂 …………………………………………………（12）
三、石方 ………………………………………………………（13）
第三节 暗挖土石方 ……………………………………………（14）
一、土方 ………………………………………………………（14）
二、石方 ………………………………………………………（16）
第四节 盖挖土石方 ……………………………………………（17）
一、土方 ………………………………………………………（17）
二、石方 ………………………………………………………（18）

北京市住房和城乡建设委员会

第五节 竖井挖土石方 …………………………………………………………………………………… (19)
一、土方 ……………………………………………………………………………………………… (19)
二、石方 ……………………………………………………………………………………………… (20)
第六节 填方 …………………………………………………………………………………………… (21)
第七节 钢管柱抽泥浆 ………………………………………………………………………………… (24)
第八节 土方、石方、泥浆及盾构渣土运输 …………………………………………………………… (25)

第二章　桩与支护工程

说明…………………………………………………………………………………………………… (31)
工程量计算规则 ……………………………………………………………………………………… (32)
第一节 机械钻孔桩成孔 ……………………………………………………………………………… (35)
一、冲击式钻机钻孔 …………………………………………………………………………………… (35)
二、旋挖钻机钻孔 ……………………………………………………………………………………… (36)
三、隧道内机械钻孔 …………………………………………………………………………………… (37)
第二节 地下连续墙成槽与接头处理 …………………………………………………………………… (39)
一、导墙 ……………………………………………………………………………………………… (39)
二、成槽 ……………………………………………………………………………………………… (40)
三、接头处理…………………………………………………………………………………………… (42)
第三节 桩与支护工程混凝土 ………………………………………………………………………… (44)
一、桩混凝土…………………………………………………………………………………………… (44)
二、隧道内桩混凝土 …………………………………………………………………………………… (46)

北京市住房和城乡建设委员会

三、地下连续墙混凝土 …… (47)
四、冠梁与腰梁混凝土 …… (48)
五、凿除混凝土 …… (49)
第四节 护坡 …… (50)
一、护坡与台阶 …… (50)
二、砂石垫层 …… (52)
第五节 明挖基坑与边坡支护 …… (53)
一、预应力锚索 …… (53)
二、护坡土钉与锁脚锚管 …… (57)
三、基坑支护喷射混凝土 …… (59)
四、施工钢支撑与混凝土支撑 …… (60)
第六节 暗挖与盖挖支护 …… (62)
一、小导管与袖阀管 …… (62)
二、大管棚 …… (63)
三、小导管、袖阀管和大管棚注浆 …… (69)
四、深孔注浆 …… (70)
五、隧道内锚杆 …… (71)
第七节 钢筋 …… (72)
一、桩 …… (72)
二、隧道内桩 …… (73)
三、地下连续墙 …… (74)
北京市住房和城乡建设委员会

四、钢筋网制作安装 …………………………………………………………………… (76)

第三章　地基加固工程

说明…………………………………………………………………………………… (79)
工程量计算规则 ……………………………………………………………………… (79)
第一节 地基处理 ……………………………………………………………………… (81)
一、换填砂、石屑、块石 ……………………………………………………………… (81)
二、换填灰土………………………………………………………………………… (82)
三、水泥稳定土……………………………………………………………………… (83)
四、水泥粉煤灰碎石桩 ……………………………………………………………… (84)
五、夯填桩 …………………………………………………………………………… (85)
六、铺土工织物……………………………………………………………………… (86)
七、凿桩头 …………………………………………………………………………… (87)
第二节 地基加固 ……………………………………………………………………… (88)
一、分层注浆与压密注浆 …………………………………………………………… (88)
二、高压旋喷水泥桩 ………………………………………………………………… (89)
三、水泥搅拌桩……………………………………………………………………… (90)

第四章　主体结构混凝土与钢筋工程

说明…………………………………………………………………………………… (93)
工程量计算规则 ……………………………………………………………………… (94)

北京市住房和城乡建设委员会

第一节 高架结构工程 …… (97)
一、现浇混凝土 …… (97)
二、预制混凝土构件安装 …… (104)
三、支座安装 …… (107)
四、压浆管道与压浆 …… (109)
五、桥面栏杆与防撞墩 …… (110)
六、隔声屏障 …… (111)
七、伸缩缝 …… (112)
第二节 明挖结构工程 …… (113)
一、竖井混凝土 …… (113)
二、明挖结构混凝土 …… (114)
第三节 暗挖与盖挖结构工程 …… (117)
一、喷射混凝土 …… (117)
二、暗挖与盖挖车站混凝土结构 …… (118)
三、暗挖区间混凝土 …… (121)
四、暗挖与盖挖车站钢管柱混凝土 …… (122)
第四节 钢筋 …… (123)
一、钢筋制作安装 …… (123)
二、桥梁预应力钢筋 …… (128)
三、直螺纹套筒接头 …… (131)
四、植筋 …… (133)

北京市住房和城乡建设委员会

第五节 疏散平台 …… (137)
一、混凝土定型化学锚栓安装 …… (137)
二、疏散平台安装 …… (138)
三、疏散平台扶手安装 …… (139)
四、爬梯安装 …… (140)
五、疏散平台导向标识安装 …… (141)
第六节 结构拆除 …… (142)
一、拆除混凝土 …… (142)
二、水钻开孔 …… (143)

第五章 砌筑工程

说明 …… (147)
工程量计算规则 …… (147)
第一节 高架砌筑工程 …… (149)
一、浆砌片石及料石 …… (149)
二、浆砌混凝土预制块 …… (150)
三、砖砌体 …… (151)
四、砌块砌体 …… (152)
第二节 地面车站砌筑工程 …… (153)
一、砖砌体 …… (153)
二、砌块砌体 …… (154)

北京市住房和城乡建设委员会

第三节 地下车站砌筑工程……（155）
一、砖砌体……（155）
二、砌块砌体……（156）

第六章　钢结构工程

说明……（159）
工程量计算规则……（159）
第一节 高架钢结构……（161）
第二节 栏杆……（163）
第三节 钢管柱……（164）
一、高架钢管柱……（164）
二、暗挖车站隧道内钢管柱……（165）
三、盖挖车站钢管柱与钢套筒……（166）

第七章　盾构工程

说明……（171）
工程量计算规则……（172）
第一节 盾构机安装与拆除……（175）
第二节 车架安装与拆除……（176）
第三节 盾构掘进……（177）
一、ϕ6000 土压平衡式盾构掘进……（177）

北京市住房和城乡建设委员会

二、$\phi \leq 7000$ 土压平衡式盾构掘进 …… (179)
三、$\phi \leq 8000$ 土压平衡式盾构掘进 …… (180)
四、$\phi \leq 9000$ 土压平衡式盾构掘进 …… (181)
五、$\phi \leq 11000$ 土压平衡式盾构掘进 …… (182)
六、$\phi 6000$ 泥水平衡式盾构掘进 …… (183)
七、$\phi \leq 7000$ 泥水平衡式盾构掘进 …… (184)
八、$\phi \leq 8000$ 泥水平衡式盾构掘进 …… (185)
九、$\phi \leq 9000$ 泥水平衡式盾构掘进 …… (186)
十、$\phi \leq 11000$ 泥水平衡式盾构掘进 …… (187)
第四节 管片密封条与嵌缝 …… (188)
一、管片设置密封条 …… (188)
二、管片嵌缝 …… (190)
第五节 衬砌压浆 …… (191)
第六节 柔性接缝 …… (192)
一、临时阶段 …… (192)
二、正式阶段 …… (195)
第七节 负环段管片与隧道内管线拆除 …… (196)
第八节 盾构其他工程 …… (198)
一、盾构基座 …… (198)
二、手孔封堵 …… (199)
三、密封舱添加材料 …… (200)
北京市住房和城乡建设委员会

四、盾构过站、盾构过工作井、盾构平移和盾构掉头 ………………………… (201)

第八章 防水工程

说明 ………………………… (207)
工程量计算规则 ………………………… (207)
第一节 桥面防水层与泄水管 ………………………… (209)
一、桥面防水层 ………………………… (209)
二、泄水管安装 ………………………… (211)
第二节 明挖结构防水 ………………………… (212)
第三节 暗挖与盖挖结构防水 ………………………… (215)
第四节 施工缝与变形缝 ………………………… (218)

第九章 模板工程

说明 ………………………… (223)
工程量计算规则 ………………………… (223)
第一节 地上结构模板 ………………………… (227)
第二节 高架结构模板 ………………………… (228)
第三节 明挖车站模板 ………………………… (234)
第四节 明挖区间模板 ………………………… (237)
第五节 暗挖车站模板 ………………………… (238)

北京市住房和城乡建设委员会

第六节 盖挖车站模板 ………………………………………………………………… (242)
第七节 暗挖区间模板 ………………………………………………………………… (243)
第八节 竖井与其他模板 ……………………………………………………………… (244)

第十章 降水工程

说明 ……………………………………………………………………………………… (247)
工程量计算规则 ……………………………………………………………………… (247)
第一节 管井安装 ……………………………………………………………………… (249)
一、水泥管井安装 ……………………………………………………………………… (249)
二、钢管井安装 ………………………………………………………………………… (250)
三、引渗井安装 ………………………………………………………………………… (251)
四、辐射井安装 ………………………………………………………………………… (252)
五、辐射水平井安装 …………………………………………………………………… (253)
第二节 管井抽水 ……………………………………………………………………… (254)

第十一章 监测工程

说明 ……………………………………………………………………………………… (257)
工程量计算规则 ……………………………………………………………………… (257)
第一节 监测点布设 …………………………………………………………………… (259)
一、基准点布设 ………………………………………………………………………… (259)
二、地表沉降与位移测点布设 ………………………………………………………… (260)

北京市住房和城乡建设委员会

三、建筑物变形测点布设 …… (261)
四、土体分层沉降测点布设 …… (262)
五、桩体变形测点布设 …… (263)
六、孔隙水压力测孔布设 …… (264)
七、地下管线沉降与位移测点布设 …… (266)
八、混凝土构筑物钢筋应力与混凝土应变测点布设 …… (267)
九、界面土压力测点布设 …… (268)
十、隧道沉降与收敛测点布设 …… (269)
十一、既有线变形测点布设 …… (270)
第二节　监测 …… (271)
一、地上结构监测 …… (271)
二、土体分层沉降监测 …… (272)
三、桩体变形监测 …… (273)
四、钢筋应力与混凝土应变监测 …… (274)
五、孔隙水压力、界面土压力、水位和地下管线沉降位移监测 …… (275)
六、隧道沉降与收敛监测 …… (276)
七、既有线变形监测 …… (277)

北京市住房和城乡建设委员会

北京市住房和城乡建设委员会

第一章　土石方工程

北京市住房和城乡建设委员会

北京市住房和城乡建设委员会

说　明

一、本章包括明挖土石方,明挖大型支撑挖土石方,暗挖土石方,盖挖土石方,竖井挖土石方,填方,钢管柱抽泥浆,土方、石方、泥浆及盾构渣土运输等 8 节共 68 子目。

二、土壤及岩石分类

1.土壤按一、二类土,三类土和四类土分类(见附表一 土壤分类表)。

2.岩石按极软岩、软岩、较软岩、较硬岩、坚硬岩分类(见附表二 岩石分类表)。

3.人工挖土按一、二类土,三类土和四类土分别编制,机械挖土不分土质综合编制。

4.人工凿石和机械破碎岩石按极软岩和软岩综合编制,其他岩石类型按实际开挖方案计算。

三、土壤含水率大于 25% 执行挖湿土相应子目;土壤含水率大于 40% 执行挖淤泥相应子目。

四、大型支撑挖土石方适用于有围护结构且带支撑的基坑开挖子目(含施工竖井、风井、独立盾构井等)。

五、采用桩锚支护的基坑挖土石方执行大型支撑挖土石方相应子目,其人工和机械消耗量乘以系数 0.75。

六、盖挖土方开挖以盖挖顶板下表面标高划分,顶板下表面以上的土方执行明挖土方相应子目,顶板下表面标高以下的土方执行盖挖土方相应子目。

七、竖井挖土石方按倒挂井壁法施工编制,竖井挖土方按一、二类土,三类土和四类土分类。

八、土石方工程不包括地上、地下障碍物处理及建筑物拆除后的垃圾清运,发生时另行计算。

九、明挖机械挖土不含清槽工作内容,清槽须执行明挖人工挖土方子目;清槽厚度按设计(规范)要求计算,未规定的按 300mm 计算。

北京市住房和城乡建设委员会

十、人工开挖注浆加固土方的，执行相应的挖四类土子目；暗挖、盖挖机械开挖注浆加固土方的，执行相应的挖四类土子目。

十一、土方、石方、泥浆及盾构渣土运输的装车和运输分别列项；明挖土石方和明挖大型支撑挖土石方工作内容里已包含装车，不再执行装车子目。

十二、土石方的开挖、运输均按照开挖前的天然密实体积计算。

十三、混凝土结构拆除、凿桩头、凿地下连续墙顶混凝土等项目的废弃物装车和外运执行石方（碴）装车和运输相应子目。

附表一

土壤分类表

土分类	土名称	开挖方法
一、二类土	粉土、砂土（粉砂、细砂、中砂、粗砂、砾砂）、粉质黏土、弱中盐渍土、软土（淤泥质土、泥炭、泥炭质土）、软塑红黏土、冲填土	用锹，少许用镐、条锄开挖。机械能全部直接铲挖满载者。
三类土	黏土、碎石土（圆砾、角砾）混合土、可塑红黏土、硬塑红黏土、强盐渍土、素填土、压实填土	主要用镐、条锄，少许用锹开挖。机械需部分刨松方能铲挖满载者，或可直接铲挖但不能满载者。
四类土	碎石土（卵石、碎石、漂石、块石）、坚硬红黏土、超盐渍土、杂填土	全部用镐、条锄挖掘，少许用撬棍挖掘。机械须普遍刨松方能铲挖满载者。

注：本表土的名称及其含义按现行国家标准《岩土工程勘察规范》GB50021-2001（2009 年局部修订版）定义。

北京市住房和城乡建设委员会

附表二

岩石分类表

<table>
<tr><th colspan="2">岩石分类</th><th>代表性岩石</th><th>开挖方法</th><th>单轴饱和抗压强度（Mpa）</th></tr>
<tr><td rowspan="3">软岩石</td><td>极软岩</td><td>1.全风化的各种岩石
2.各种半成岩</td><td>部分用手凿工具、部分用爆破法开挖</td><td><5</td></tr>
<tr><td>软　岩</td><td>1.强风化的坚硬岩或较硬岩
2.中等风化~强风化的较软岩
3.未风化~微风化的页岩、泥岩、泥质砂岩等</td><td>用风镐和爆破法开挖</td><td>5~15</td></tr>
<tr><td>较软岩</td><td>1.中等风化~强风化的坚硬岩或较硬岩
2 未风化~微风化的凝灰岩、千枚岩、泥灰岩、砂质泥岩等</td><td>用爆破法开挖</td><td>15~30</td></tr>
<tr><td rowspan="2">硬岩石</td><td>较硬岩</td><td>1.微风化的坚硬岩
2.未风化~微风化的大理岩、板岩、石灰岩、白云岩、钙质砂岩等</td><td rowspan="2">用爆破法开挖</td><td>30~60</td></tr>
<tr><td>坚硬岩</td><td>未风化~微风化的花岗岩、闪长岩、辉绿岩、玄武岩、安山岩、片麻岩、石英岩、石英砂岩、硅质砾岩、硅质石灰岩等</td><td>>60</td></tr>
</table>

注：本表依据现行国家标准《工程岩体分级标准》GB/T50218-2014 和《岩土工程勘察规范》GB50021-2001（2009 年局部修订版）整理。

北京市住房和城乡建设委员会

工程量计算规则

一、明挖土石方按设计图示尺寸以体积计算。

二、大型支撑挖土石方按设计图示尺寸以体积计算。

三、区间暗挖土石方按拱、墙部位设计结构初衬外侧各增加 100mm 与仰拱(底部初衬)下侧所围的面积乘以区间设计长度以体积计算。

四、车站暗挖土石方按初衬外侧墙各增加 100mm 宽度乘以顶板初衬结构外增加 100mm 至设计底板(或垫层)底的高度所计算出的面积乘以车站设计长度以体积计算。

五、盖挖土石方按设计结构外围断面面积乘以设计长度以体积计算,其设计结构外围断面面积指地下围护结构内侧之间的宽度乘以设计顶板底至底板(或垫层)底的高度所计算出的面积。

六、竖井挖土石方按设计图示尺寸以体积计算。

七、挖湿土、淤泥、流砂按设计图示尺寸以体积计算。

八、填方按挖方体积减去自然地坪以下埋设的基础体积(包括基础垫层及其他构筑物)以体积计算。钢管柱外填砂按设计图示尺寸以体积计算。隧道内回填按设计图示填充量以体积计算。

九、钢管柱抽泥浆按桩截面面积乘以钢管柱顶至钢管柱底的设计深度以体积计算。

十、土压(泥水)平衡盾构渣土(泥浆)按设计图示结构外径所围的断面面积乘以掘进长度再乘以系数 1.08 以体积计算,其中泥水平衡盾构渣土与泥浆分别按占总体积的 80%与 20%计算。

十一、土方、石方、泥浆及盾构渣土运输(装车)按设计图示尺寸以体积计算。

北京市住房和城乡建设委员会

第一节 明挖土石方

一、土方

工作内容：挖土、装车、修整底边、钎探等。　　　　**单位：**m^3

编号				1-1	1-2	1-3
项目				明挖人工挖土		
				一、二类土	三类土	四类土
工料机名称			单位	消耗量		
人工	00010701	综合用工三类	工日	0.187	0.274	0.361
机械	99030030	电动打钎机	台班	0.0039	0.0048	0.0056
	99460004	其他机具费 占人工费	%	1.50	1.50	1.50

北京市住房和城乡建设委员会

工作内容：挖土、装车、清理、修整边坡等。　　　　**单位：**m^3

编号				1-4	1-5	1-6	1-7
项目				明挖机械挖土(槽深)			
				≤5m	≤13m	≤21m	≤30m
工料机名称			单位	消耗量			
人工	00010701	综合用工三类	工日	0.009	0.010	0.011	0.013
机械	9901000301	履带式单斗挖土机 1.0m^3	台班	0.0035	0.0038	0.0042	0.0050
	9907000102	轮胎式装载机 1.5m^3	台班	0.0027	0.0031	0.0036	0.0040
	99460004	其他机具费 占人工费	%	1.50	1.50	1.50	1.50

北京市住房和城乡建设委员会

二、淤泥流砂

工作内容：1.人工挖淤泥、流砂：人工挖淤泥、流砂，修整底边，场内运输等。2.机械挖淤泥、流砂：机械挖淤泥、流砂，清理边坡和机下余土等。

单位：m^3

编号				1-8	1-9
项目				明挖人工挖淤泥、流砂	明挖机械挖淤泥、流砂
工料机名称			单位	消耗量	
人工	00010701	综合用工三类	工日	0.611	0.013
机械	9901000301	履带式单斗挖土机 1.0m^3	台班	–	0.0081
	99460004	其他机具费 占人工费	%	1.50	1.50

北京市住房和城乡建设委员会

三、石方

工作内容：1.人工凿石：凿石、清渣攒堆、清底修边等。2.机械破碎：凿石方、打碎、工作面排水、清理机下余渣等。 **单位**：m^3

编号				1-10	1-11
项目				明挖人工凿石	明挖机械破碎
工料机名称			单位	消耗量	
人工	00010701	综合用工三类	工日	0.525	0.067
机械	99010002	反铲挖掘机(带液压锤)	台班	-	0.0170
	9943000205	空压机 $6m^3/min$	台班	0.2500	-
	99330001	风镐	台班	0.5000	-
	9907000102	轮胎式装载机 $1.5m^3$	台班	-	0.0253
	99460004	其他机具费 占人工费	%	1.50	1.50

北京市住房和城乡建设委员会

第二节 明挖大型支撑挖土石方

一、土方

工作内容：机械挖土、推铲、挖引水沟、人工修整底面、清理边坡等。 **单位：**m^3

编号				1-12	1-13	1-14	1-15	1-16
项目				支撑下挖土方（槽深）				
				≤7m	≤13m	≤21m	≤30m	≤40m
工料机名称			单位	消耗量				
人工	00010701	综合用工三类	工日	0.024	0.025	0.026	0.027	0.028
机械	9901000501	挖掘机 $1m^3$	台班	0.0128	0.0162	0.0192	0.0238	0.0274
	9907000102	轮胎式装载机 $1.5m^3$	台班	0.0060	0.0064	0.0069	0.0075	0.0082
	99460004	其他机具费 占人工费	%	1.50	1.50	1.50	1.50	1.50

北京市住房和城乡建设委员会

二、淤泥流砂

工作内容：挖淤泥流砂、工作面排水、人工修整等。　　　　**单位：**m^3

编号				1-17
项目				支撑下挖淤泥流砂
工料机名称			单位	消耗量
人工	00010701	综合用工三类	工日	0.028
机械	9901000301	履带式单斗挖土机 1.0m^3	台班	0.0173
	99460004	其他机具费 占人工费	%	1.50

北京市住房和城乡建设委员会

三、石方

工作内容：1.人工凿石：凿石、清渣攒堆、清底修边等。2.机械破碎：凿石方、打碎、工作面排水、清理机下余渣等。　　**单位：**m^3

编号				1-18	1-19
项目				支撑下人工凿石	支撑下机械破碎
工料机名称			单位	消耗量	
人工	00010701	综合用工三类	工日	0.562	0.072
机械	99010002	反铲挖掘机(带液压锤)	台班	-	0.0182
	9943000205	空压机 6m^3/min	台班	0.2675	-
	99330001	风镐	台班	0.5350	-
	9907000102	轮胎式装载机 1.5m^3	台班	-	0.0271
	99460004	其他机具费 占人工费	%	1.50	1.50

北京市住房和城乡建设委员会

第三节 暗挖土石方

一、土方

工作内容:人机配合挖土方、装土、运土至洞口、由洞口提升至地面堆放、清理边墙等。 **单位:** m^3

编号				1-20	1-21	1-22	1-23	1-24	1-25
项目				暗挖区间			暗挖车站		
				一、二类土	三类土	四类土	一、二类土	三类土	四类土
工料机名称			单位	消耗量					
人工	00010704	综合用工三类	工日	0.440	0.575	0.713	0.415	0.557	0.691
机械	9909000403	桥式起重机 10t	台班	0.0075	0.0075	0.0075	0.0066	0.0066	0.0066
	99310001	电动三轮车	台班	0.0250	0.0250	0.0250	0.0220	0.0220	0.0220
	9901000401	履带式单斗挖掘机 $0.3m^3$	台班	0.0040	0.0050	0.0060	0.0040	0.0050	0.0060
	99460004	其他机具费 占人工费	%	1.50	1.50	1.50	1.50	1.50	1.50

北京市住房和城乡建设委员会

工作内容：挖淤泥、流砂、湿土、清理边墙、装运至洞口、由洞口提升至地面堆放等。 **单位：**m^3

编号				1-26	1-27
项目				挖淤泥、流砂	挖湿土
工料机名称			单位	消耗量	
人工	00010704	综合用工三类	工日	0.933	0.545
机械	9901000401	履带式单斗挖掘机 $0.3m^3$	台班	0.0070	0.0060
	9909000403	桥式起重机 10t	台班	0.0120	0.0090
	99310001	电动三轮车	台班	0.0400	0.0300
	99460004	其他机具费 占人工费	%	1.50	1.50

北京市住房和城乡建设委员会

二、石方

工作内容：1.人工凿石：凿石、清渣攒堆、清底修边、装运至洞口、由洞口提升至地面堆放等。2.机械破碎：凿石方、打碎、工作面排水、清理机下余渣、装运至洞口、由洞口提升至地面堆放等。

单位：m^3

编号				1-28	1-29
项目				暗挖人工凿石	暗挖机械破碎
工料机名称			单位	消耗量	
人工	00010704	综合用工三类	工日	1.082	0.784
机械	9901000401	履带式单斗挖掘机 $0.3m^3$	台班	–	0.0300
	9943000205	空压机 $6m^3/min$	台班	0.3300	0.0360
	99330001	风镐	台班	0.6600	0.0720
	9909000403	桥式起重机 10t	台班	0.0090	0.0090
	99310001	电动三轮车	台班	0.0350	0.0350
	9933000003	履带式液压岩石破碎机 105kW	台班	–	0.0300
	99460004	其他机具费 占人工费	%	1.50	1.50

北京市住房和城乡建设委员会

第四节 盖挖土石方

一、土方

工作内容:人机配合装土、卸土、运土至洞口、由洞口提升至地面堆放等。　　　　**单位:**m^3

编号				1-30	1-31	1-32
项目				盖挖土方		
				一、二类土	三类土	四类土
工料机名称			单位	消耗量		
人工	00010704	综合用工三类	工日	0.205	0.228	0.254
机械	9901000401	履带式单斗挖掘机 $0.3m^3$	台班	0.0129	0.0158	0.0186
	9909000403	桥式起重机 10t	台班	0.0050	0.0050	0.0050
	99310001	电动三轮车	台班	0.0250	0.0250	0.0250
	99460004	其他机具费 占人工费	%	1.50	1.50	1.50

北京市住房和城乡建设委员会

二、石方

工作内容：1.人工凿石：凿石、清渣攒堆、清底修边、装运至洞口、由洞口提升至地面堆放等。2.机械破碎：凿石方、打碎、工作面排水、清理机下余渣、装运至洞口、由洞口提升至地面堆放等。

单位：m^3

编号				1-33	1-34
项目				盖挖人工凿石	盖挖机械破碎
工料机名称			单位	消耗量	
人工	00010704	综合用工三类	工日	0.984	0.608
机械	99330001	风镐	台班	0.6600	0.0720
	9901000401	履带式单斗挖掘机 $0.3m^3$	台班	–	0.0234
	9933000003	履带式液压岩石破碎机 105kW	台班	–	0.0234
	9909000403	桥式起重机 10t	台班	0.0060	0.0060
	99310001	电动三轮车	台班	0.0300	0.0300
	9943000205	空压机 $6m^3/min$	台班	0.3300	0.0360
	99460004	其他机具费 占人工费	%	1.50	1.50

北京市住房和城乡建设委员会

第五节 竖井挖土石方

一、土方

工作内容:人工挖土方、清理基底、修理边墙、土方装运、提升至地面堆放等。 **单位:**m³

编号				1-35	1-36	1-37	1-38	1-39	1-40
项目				竖井挖土方(深度)					
				≤20m			≤40m		
				一、二类土	三类土	四类土	一、二类土	三类土	四类土
工料机名称			单位	消耗量					
人工	00010701	综合用工三类	工日	0.208	0.289	0.385	0.249	0.347	0.462
机械	9909000016	汽车式起重机 20t	台班	0.0071	0.0071	0.0071	0.0076	0.0076	0.0076
机械	99460004	其他机具费 占人工费	%	1.50	1.50	1.50	1.50	1.50	1.50

北京市住房和城乡建设委员会

二、石方

工作内容：人工挖石方、清理基底、修理边墙、石方装运、提升至地面堆放等。 **单位：**m^3

编号				1-41
项目				竖井人工凿石
工料机名称			单位	消耗量
人工	00010701	综合用工三类	工日	0.551
机械	9909000016	汽车式起重机 20t	台班	0.0076
	99330001	风镐	台班	0.5000
	9943000205	空压机 6m^3/min	台班	0.2500
	99460004	其他机具费 占人工费	%	1.50

北京市住房和城乡建设委员会

第六节 填方

工作内容:1.回填素土:土方摊铺、分层夯实等。2.回填3:7灰土:灰土配料、回填、分层夯实等。3.回填砂:铺料、整平、洒水、夯实等。4.回填级配砂石:回填、平整、分层碾压等。

单位:m^3

编号				1-42	1-43	1-44	1-45
项目				回填素土	回填3:7灰土	回填砂	回填级配砂石
工料机名称			单位	消耗量			
人工	00010701	综合用工三类	工日	0.084	0.274	0.084	0.109
材料	04090008	素土	m^3	1.4900	1.2100	-	-
	04090026	熟石灰	kg	-	413.1000	-	-
	0403000003-2	砂子 中粗砂	kg	-	-	1854.2000	-
	04050011-2	级配砂石	kg	-	-	-	2315.4000
	34000011	其他材料费 占材料费	%	1.00	1.00	1.00	1.00
机械	9913000202	电动夯实机 20~62kg/m	台班	0.0750	0.0800	0.0850	0.0860
	99460004	其他机具费 占人工费	%	1.50	1.50	1.50	1.50

北京市住房和城乡建设委员会

工作内容：1.隧道内回填素土：垂直及洞内运输、摊铺、分层夯实等。2.隧道内回填 3：7 灰土：垂直及洞内运输、分层铺筑、找平、压实等。3.隧道内回填级配砂石：垂直及洞内运输、拌合、分层铺筑、找平、压实等。4.隧道内回填混凝土：进料、垂直及洞内运输、浇筑、振捣及养护等。

单位：m^3

编号				1-46	1-47	1-48	1-49
项目				隧道内回填			
				素土	3：7 灰土	级配砂石	混凝土
工料机名称			单位	消耗量			
人工	00010704	综合用工三类	工日	0.099	0.302	0.242	0.395
材料	04090008	素土	m^3	1.4900	1.2100	–	–
	04090026	熟石灰	kg	–	413.1000	–	–
	04050011-2	级配砂石	kg	–	–	2315.4000	–
	8021000803	预拌混凝土 C20	m^3	–	–	–	1.0350
	5135000101	泵管 ϕ150	kg	–	–	–	0.8100
	34000011	其他材料费 占材料费	%	1.00	1.00	1.00	1.00
机械	9913000202	电动夯实机 20~62kg/m	台班	0.0825	0.0860	0.0900	–
	9909000403	桥式起重机 10t	台班	0.0140	0.0140	0.0140	–
	99310001	电动三轮车	台班	0.1700	0.1700	0.1700	–
	99050003	混凝土振捣器(插入式)	台班	–	–	–	0.0800
	99460004	其他机具费 占人工费	%	1.50	1.50	1.50	1.50

北京市住房和城乡建设委员会

工作内容：垂直及洞内运输、分层填筑、压实等。　　单位：m^3

编号				1-50	1-51
项目				钢管柱外填砂	
				盖挖车站	暗挖车站
工料机名称			单位	消耗量	
人工	00010704	综合用工三类	工日	0.377	0.484
材料	0403000003-2	砂子 中粗砂	kg	1895.0000	1895.0000
	34000011	其他材料费 占材料费	%	1.00	1.00
机械	9909000403	桥式起重机 10t	台班	-	0.0060
	99310001	电动三轮车	台班	0.1200	0.1700
	99460004	其他机具费 占人工费	%	1.50	1.50

北京市住房和城乡建设委员会

第七节 钢管柱抽泥浆

工作内容：搭拆工作平台、下放泥浆泵、抽泥浆、清理套筒等。 **单位：**m^3

编号				1-52	1-53
项目				钢管柱抽泥浆	
				盖挖车站	暗挖车站
工料机名称			单位	消耗量	
人工	00010704	综合用工三类	工日	0.144	0.173
机械	99440014	高压泥浆泵	台班	0.1080	0.1296
	99460004	其他机具费 占人工费	%	1.50	1.50

北京市住房和城乡建设委员会

第八节 土方、石方、泥浆及盾构渣土运输

工作内容：1.装车：机械装车、人工配合清理等。2.运输：自卸汽车运输、卸车、消纳等。　　**单位：**m^3

编号				1-54	1-55	1-56	1-57	1-58	1-59
项目				土方装车	土方运输		石方(碴)、渣土装车	石方(碴)、渣土运输	
					运距 1km 以内	运距每增 1km		运距 1km 以内	运距每增 1km
工料机名称			单位	消耗量					
人工	00010701	综合用工三类	工日	0.010	–	–	0.011	–	–
材料	34000009	弃土或渣土消纳	m^3	–	1.0000	–	–	1.0000	–
机械	9907000012	自卸汽车 12t	台班	–	0.0041	0.0018	–	0.0057	0.0025
	9907000102	轮胎式装载机 1.5m^3	台班	0.0027	–	–	0.0036	–	–
	9901000301	履带式单斗挖土机 1.0m^3	台班	0.0033	–	–	0.0046	–	–
	99460004	其他机具费 占人工费	%	1.50	–	–	1.50	–	–

北京市住房和城乡建设委员会

工作内容：1.装车：机械装车（泥浆泵抽泥浆）、人工配合清理等。2.运输：自卸汽车（泥浆运输车）运输、卸车（抽出泥浆）、消纳等。

单位：m^3

编号				1-60	1-61	1-62	1-63	1-64	1-65
项目				淤泥、流砂装车	淤泥、流砂运输		泥浆装车	泥浆运输	
					运距 1km 以内	运距每增 1km		运距 1km 以内	运距每增 1km
工料机名称			单位	消耗量					
人工	00010701	综合用工三类	工日	0.016	–	–	0.022	–	–
材料	34000009	弃土或渣土消纳	m^3	–	1.0000	–	–	1.0000	–
机械	9907000012	自卸汽车 12t	台班	–	0.0057	0.0025	–	–	–
	9901000301	履带式单斗挖土机 $1.0m^3$	台班	0.0079	–	–	–	–	–
	9944000006	泥浆泵 ϕ100mm	台班	–	–	–	0.0135	–	–
	9907001003	泥浆运输车 4000L	台班	–	–	–	–	0.0050	0.0016
	99460004	其他机具费 占人工费	%	1.50	–	–	1.50	–	–

北京市住房和城乡建设委员会

工作内容：1.装车：机械装车、人工配合清理等。2.运输：自卸汽车运输、卸盾构渣土、消纳等。 **单位：**m^3

编号				1-66	1-67	1-68
项目				盾构渣土装车	盾构渣土运输	
					运距 1km 以内	运距每增 1km
工料机名称			单位	消耗量		
人工	00010701	综合用工三类	工日	0.011	–	–
材料	34000009	弃土或渣土消纳	m^3	–	1.0000	–
机械	9907000012	自卸汽车 12t	台班	–	0.0057	0.0024
	9901000301	履带式单斗挖土机 1.0m^3	台班	0.0036	–	–
	9907000102	轮胎式装载机 1.5m^3	台班	0.0029	–	–
	99460004	其他机具费 占人工费	%	1.50	–	–

北京市住房和城乡建设委员会

北京市住房和城乡建设委员会

第二章　桩与支护工程

北京市住房和城乡建设委员会

北京市住房和城乡建设委员会

说　　明

一、本章包括：机械钻孔桩成孔，地下连续墙成槽与接头处理，桩与支护工程混凝土，护坡，明挖基坑与边坡支护，暗挖与盖挖支护，钢筋 7 节共 143 个子目。

二、本章适用于明挖、盖挖和暗挖的围护及支护等工程，同时适用于高架、盖挖和暗挖的桩基工程。

三、机械钻孔桩成孔

1.钻孔子目中已包含钢护筒埋设。

2.隧道内机械钻孔子目中不含泥浆制作及其材料，其费用按实际方案计算。

四、机械钻孔桩成孔和地下连续墙成槽按综合土质编制，遇到岩石或其他障碍时，按实际方案计算。

五、冲击钻钻孔桩按充盈系数 1.20 编制，旋挖钻机钻孔桩和隧道内机械钻孔桩按充盈系数 1.15 编制，地下连续墙按充盈系数 1.15 编制，设计图纸不同时，可调整材料消耗量。

六、地下连续墙清底置换的消耗量每段按 6m 编制，设计不同时可调整。

七、凿桩顶混凝土和地下连续墙顶混凝土子目中不包括剔凿渣土装车与运输，发生时执行第一章相应子目。

八、施工钢支撑制作主材（型钢/钢管）未考虑摊销，其摊销次数应根据工程情况确定。

九、暗挖与盖挖支护

1.小导管、袖阀管、大管棚和锚杆按综合土质编制。

2.大管棚适用于暗挖结构隧道内施工，若在隧道外作业或地面作业时，其人工、机械消耗量应乘以系数 0.75。

3.小导管、大管棚的主材规格和壁厚与设计图纸不同时可调整。

北京市住房和城乡建设委员会

4.大管棚不含管内取土和灌浆,发生时按设计方案另行计算。

5.若设计注浆浆液配比与本标准不同,可调整。

十、锁脚锚管注浆执行小导管与大管棚注浆相应子目。

十一、自进式锚杆子目中连接套按“3 米/节”编制,设计不同时可调整。

十二、深孔注浆已综合了成孔和注浆的工作内容,水泥砂浆水玻璃浆液配比按照 1∶1(体积比)编制,设计不同时可调整。

十三、钢筋

1.钢筋笼安装深度指设计桩顶标高与桩底标高之差。

2.钢筋安装采用直螺纹套筒连接的,执行第四章相应子目。

3.冠梁和腰梁钢筋执行第四章明挖工程相应子目。

4.钢筋网片搭接数量按实际发生计算。

十四、声测管按焊接钢管进行编制,设计材质不同时可调整。

十五、钻孔灌注桩(含隧道内钻孔灌注桩)、地下连续墙和锚索成孔等子目不含渣土和泥浆外弃,发生时执行第一章土石方相应子目。

工程量计算规则

一、冲击式钻机和旋挖钻机钻孔按设计图示的成孔长度乘以设计桩截面积,以体积计算。

二、隧道内机械钻孔按设计图示的成孔长度计算。

三、地下连续墙成槽

1.导墙开挖、连续墙挖土成槽按设计图示墙中心线长乘以厚度乘以槽深以体积计算。

北京市住房和城乡建设委员会

2.锁口管吊拔、接头箱吊拔、清底置换按设计图示数量以段计算,子目中已包括锁口管的摊销费用。

四、钻孔灌注桩、地下连续墙、锚索等成孔的渣土外弃数量按设计桩(墙、孔)截面面积乘以设计桩(墙、孔)成孔深度以体积计算;使用泥浆护壁的,弃土与泥浆所占比例按下表计算。

弃土泥浆工程量计算表

类型	钻孔灌注桩	地下连续墙	锚索成孔
弃土(石碴)占比(%)	50	50	90
弃泥浆量占比(%)	50	50	10

五、桩基及支护工程混凝土

1.旋挖钻、冲击钻和隧道内机械钻孔桩混凝土按设计图示桩截面积乘以设计桩长与加灌长度之和以体积计算。

2.灌注桩后注浆按设计图示注浆量以体积计算。

3.清底置换按设计图示以段计算。

4.导墙混凝土、地下连续墙混凝土浇筑按设计图示墙中心线长乘以墙宽乘以槽深以体积计算。

5.混凝土冠梁和腰梁按设计图示尺寸以体积计算。

6.钢腰梁按设计图示尺寸以质量计算,不扣除孔眼质量,焊条、铆钉、螺栓等不另增加质量。

7.凿除桩顶混凝土按设计图示桩水平截面积乘以设计图示凿除高度(设计未规定按规范要求)以体积计算。

8.凿除地下连续墙顶混凝土按设计图示墙水平截面积乘以设计图示凿除高度(设计未规定按规范要求)以体积计算。

六、护坡、块石锥型坡、台阶、混凝土垫层均按设计图示尺寸以体积计算。

北京市住房和城乡建设委员会

七、预应力锚索、护坡土钉、锁脚锚管

1.预应力锚索钻孔和预应力锚索钢绞线均按设计图示尺寸以钻孔深度计算。

2.预应力锚索注浆按设计图示尺寸以长度计算。

3.锚墩、承压板制作安装按设计图示以套计算。

4.护坡土钉制作、安装按设计图示尺寸乘理论重量以质量计算。

5.护坡土钉钻孔、注浆按设计图示尺寸以钻孔深度计算。

6.锁脚锚管按设计图示尺寸以长度计算。

八、基坑支护喷射混凝土按设计图示尺寸以面积计算。

九、施工钢支撑按设计图示尺寸乘理论重量以质量计算。

十、混凝土支撑梁按设计图示尺寸以体积计算。

十一、小导管、袖阀管和大管棚均按设计图示尺寸以长度计算。

十二、深孔注浆按设计图示以注浆浆液体积计算。

十三、小导管、袖阀管和大管棚注浆按设计图示以注浆浆液体积计算。

十四、砂浆锚杆、自进式锚杆(杆体)按设计图示尺寸以长度计算。

十五、钢筋

1.钢筋笼制作安装、隧道内钢筋笼制作安装按设计图示尺寸乘理论重量以质量计算。

2.型钢制作安装和连续墙钢筋制作安装按设计图示尺寸乘理论重量以质量计算。

3.导墙钢筋按设计图示尺寸乘理论质量计算。

4.声测管埋设按设计图示尺寸以长度计算。

5.注浆管埋设按设计图示尺寸以长度计算。

6.钢筋网制作安装按设计图示尺寸乘理论重量以质量计算。

北京市住房和城乡建设委员会

第一节 机械钻孔桩成孔

一、冲击式钻机钻孔

工作内容：定位、钢护筒埋设及拆除、钻机就位、泥浆制作、泥浆配送、钻进、出渣、清孔等。 **单位：**m^3

编号				2-1	2-2	2-3	2-4
项目				冲击钻机成孔(桩径)			
				≤1000mm	≤1200mm	≤1500mm	≤1800mm
工料机名称			单位	消耗量			
人工	00010501	综合用工二类	工日	1.268	1.245	1.132	1.098
材料	03130109	低合金钢焊条 E43 系列	kg	0.0507	0.0498	0.0453	0.0439
	03150906	铁件	kg	0.0127	0.0125	0.0113	0.0110
	05030007	板方材	m^3	0.0007	0.0007	0.0006	0.0006
	04090056	粘土	m^3	1.7762	1.7445	1.5859	1.5384
	33010026	钢护筒	kg	0.9942	0.9765	0.8877	0.8611
	34000011	其他材料费 占材料费	%	1.00	1.00	1.00	1.00
机械	9903001201	电动冲击钻 22 型	台班	0.7198	0.7070	-	-
	9909000401	轮胎起重机 16t	台班	0.0008	0.0008	0.0007	0.0007
	9901000301	履带式单斗挖土机 $1.0m^3$	台班	0.0011	0.0011	0.0010	0.0010
	9907000006	载重汽车 10t	台班	0.0008	0.0008	0.0007	0.0007
	9925000002	交流弧焊机 32kV·A	台班	0.0057	0.0056	0.0051	0.0049
	9903001202	电动冲击钻 30 型	台班	-	-	0.6427	0.6234
	99460004	其他机具费 占人工费	%	1.50	1.50	1.50	1.50

北京市住房和城乡建设委员会

二、旋挖钻机钻孔

工作内容：护筒埋设及拆除，钻机就位、钻孔、提钻、出渣、渣土清理堆放，造浆、压浆、清孔等。　　　　**单位：**m^3

编号				2-5	2-6	2-7	2-8	2-9
项目				旋挖钻机钻桩孔（桩径）				
				≤800mm	≤1000mm	≤1200mm	≤1500mm	≤2000mm
工料机名称			单位	消耗量				
人工	00010501	综合用工二类	工日	0.384	0.320	0.267	0.222	0.185
材料	04090007	膨润土	kg	27.7280	27.7280	27.7280	27.7280	27.7280
	33010026	钢护筒	kg	1.0688	1.0046	0.9440	0.8877	0.8344
	34000011	其他材料费 占材料费	%	1.00	1.00	1.00	1.00	1.00
机械	9907000102	轮胎式装载机 $1.5m^3$	台班	0.0385	0.0362	0.0285	0.0268	0.0252
	9901000006	履带式单斗液压挖掘机 $1m^3$	台班	0.0089	0.0084	0.0079	0.0074	0.0070
	9944000006	泥浆泵 ϕ100mm	台班	0.0769	0.0723	0.0570	0.0536	0.0504
	9944000007	潜水泵 ϕ100mm	台班	0.0446	0.0419	0.0394	0.0370	0.0348
	99030046	旋挖钻机 ϕ1000 及以内	台班	0.0769	0.0723	–	–	–
	99030047	旋挖钻机 ϕ1000 以外	台班	–	–	0.0570	0.0536	0.0504
	99460004	其他机具费 占人工费	%	1.50	1.50	1.50	1.50	1.50

北京市住房和城乡建设委员会

三、隧道内机械钻孔

工作内容：准备工作、垂直及洞内运输、装拆钻架、移机就位、钻进、提钻、出渣、清孔、测量孔径和孔深等。　　　　**单位：**m

编号				2-10	2-11	2-12
项目				隧道内机械钻孔		
				桩径≤800mm	桩径≤1000mm	桩径≤1200mm
				深度≤30m		
工料机名称			单位	消耗量		
人工	00010504	综合用工二类	工日	1.164	1.455	1.746
材料	03130101	电焊条（综合）	kg	0.0687	0.0859	0.1030
	03150906	铁件	kg	0.6398	0.7998	0.9597
	03151314	冲抓钻头	套	0.0661	0.0827	0.0992
	33010026	钢护筒	kg	0.2200	0.2600	0.4000
	34000011	其他材料费 占材料费	%	1.00	1.00	1.00
机械	9903001116	DZK-8 型钻机 125kw	台班	0.2659	0.3324	0.3989
	9909000403	桥式起重机 10t	台班	0.0132	0.0159	0.0192
	99310001	电动三轮车	台班	0.1250	0.1250	0.1250
	9925000202	交流电焊机 32kV·A	台班	0.0057	0.0072	0.0086
	99460004	其他机具费 占人工费	%	1.50	1.50	1.50

北京市住房和城乡建设委员会

工作内容：准备工作、垂直及洞内运输、装拆钻架、移机就位、钻进、提钻、出渣、清孔、测量孔径和孔深等。 **单位：**m

编号				2-13	2-14	2-15	2-16	2-17	2-18
项目				洞内机械钻孔	隧道内机械钻孔				
				桩径≤1500mm		桩径≤1800mm		桩径≤2000mm	
				深度≤30m	深度≤50m	深度≤30m	深度≤50m	深度≤30m	深度≤50m
工料机名称			单位	消耗量					
人工	00010504	综合用工二类	工日	2.327	2.756	3.095	3.250	4.047	4.249
材料	03130101	电焊条（综合）	kg	0.1374	0.1374	0.1827	0.1827	0.2238	0.2238
	03150906	铁件	kg	1.2796	1.4573	1.7019	2.0160	2.0848	2.4740
	03151314	冲抓钻头	套	0.1323	0.1507	0.1759	0.2090	0.2155	0.2560
	33010026	钢护筒	kg	0.5000	0.5000	0.5500	0.5500	0.6000	0.6000
	34000011	其他材料费 占材料费	%	1.00	1.00	1.00	1.00	1.00	1.00
机械	9903001117	DZK-10 型钻机 171kw	台班	0.5318	0.6298	0.7073	0.7427	0.8665	0.9098
	99310001	电动三轮车	台班	0.1250	0.1250	0.1250	0.1250	0.1250	0.1250
	9909000403	桥式起重机 10t	台班	0.0245	0.0245	0.0265	0.0265	0.0304	0.0304
	9925000202	交流电焊机 32kV·A	台班	0.0115	0.0115	0.0152	0.0152	0.0187	0.0187
	99460004	其他机具费 占人工费	%	1.50	1.50	1.50	1.50	1.50	1.50

北京市住房和城乡建设委员会

第二节 地下连续墙成槽与接头处理

一、导墙

工作内容：定位、人机配合挖土、场内运输堆放、人工整修、沟槽排水等。　　　　**单位：**m^3

编号				2-19
项目				导墙开挖
工料机名称			单位	消耗量
人工	00010501	综合用工二类	工日	0.054
机械	9901000301	履带式单斗挖土机 $1.0m^3$	台班	0.0111
	99460004	其他机具费 占人工费	%	1.50

北京市住房和城乡建设委员会

二、成槽

工作内容：机具定位、安放跑板导轨、制浆、输送、循环分离泥浆、挖土成槽、护壁整修测量、场内运输等。 **单位：**m^3

编号				2-20	2-21	2-22	2-23
项目				地下连续墙成槽			
				履带式液压抓斗(槽深)			
				≤25m	≤35m	≤45m	≤55m
工料机名称			单位	消耗量			
人工	00010501	综合用工二类	工日	0.513	0.580	0.725	0.834
材料	04090007	膨润土	kg	20.0800	20.0800	20.0800	20.0800
	34000011	其他材料费 占材料费	%	1.00	1.00	1.00	1.00
机械	99010003	履带式液压抓斗成槽机	台班	0.0630	0.0820	0.0900	0.0990
	99450017	超声波测壁机	台班	0.0260	0.0260	0.0260	0.0260
	99350001	泥浆制作循环设备	台班	0.0590	0.0780	0.0870	0.0957
	9907000102	轮胎式装载机 $1.5m^3$	台班	0.0226	0.0241	0.0304	0.0419
	99460004	其他机具费 占人工费	%	1.50	1.50	1.50	1.50

北京市住房和城乡建设委员会

工作内容：机具定位、安放跑板导轨、制浆、输送、循环分离泥浆、挖土成槽、护壁整修测量、场内运输、堆土等。 单位：m^3

编号				2-24	2-25
项目				地下连续墙成槽	
				二钻一抓(槽深)	
				≤25m	≤35m
工料机名称			单位	消耗量	
人工	00010501	综合用工二类	工日	0.612	0.757
材料	04090007	膨润土	kg	20.0800	20.0800
	34000011	其他材料费 占材料费	%	1.00	1.00
机械	99010003	履带式液压抓斗成槽机	台班	0.0590	0.0620
	99450017	超声波测壁机	台班	0.0260	0.0260
	99350001	泥浆制作循环设备	台班	0.0590	0.0620
	9944000006	泥浆泵 ϕ100mm	台班	0.1160	0.1435
	9907000010	自卸汽车 8t	台班	0.0178	0.0190
	9903001101	反循环钻机 60P45A	台班	0.0100	0.0130
	99460004	其他机具费 占人工费	%	1.50	1.50

北京市住房和城乡建设委员会

三、接头处理

工作内容：锁口管对接组装、入槽就位、上下移动、拔出、拆卸、冲洗堆放等。 **单位：**段

编号				2-26	2-27	2-28	2-29
项目				锁口管吊拔（槽深）			
				≤25m	≤35m	≤45m	≤55m
工料机名称			单位	消耗量			
人工	00010501	综合用工二类	工日	6.481	7.129	7.842	8.626
材料	18150041	锁口管	kg	48.1600	90.9500	122.8300	128.9700
	34000011	其他材料费 占材料费	%	1.00	1.00	1.00	1.00
机械	9909000006	履带式起重机 40t	台班	1.0870	1.0925	–	–
	99430018	锁口管顶升机	台班	1.1800	1.4600	1.7600	2.1243
	9909000032	履带式起重机 60t	台班	–	–	0.9813	–
	9909000007	履带式起重机 100t	台班	–	–	–	0.9833
	99460004	其他机具费 占人工费	%	1.50	1.50	1.50	1.50

北京市住房和城乡建设委员会

工作内容：接头箱安装、拔除、拆卸、冲洗、堆放等。　　**单位：**段

编号				2-30	2-31	2-32	2-33
项目				接头箱吊拔(槽深)			
				≤25m	≤35m	≤45m	≤55m
工料机名称			单位	消耗量			
人工	00010501	综合用工二类	工日	6.997	7.697	8.467	10.245
材料	01050001	钢丝绳	kg	0.3660	0.4880	0.6100	0.7381
	55490039	接头箱	kg	70.0400	120.9600	133.0560	160.9978
	34000011	其他材料费 占材料费	%	1.00	1.00	1.00	1.00
机械	9909000006	履带式起重机 40t	台班	0.7285	0.9021	1.0850	1.3129
	99430018	锁口管顶升机	台班	1.0691	1.3239	1.5923	1.9267
	99460004	其他机具费 占人工费	%	1.50	1.50	1.50	1.50

北京市住房和城乡建设委员会

第三节 桩与支护工程混凝土

一、桩混凝土

工作内容:安拆导管、混凝土浇筑、振捣养护等。 **单位:**m³

编号				2-34	2-35
项目				灌注桩混凝土	
				冲击钻	旋挖钻
工料机名称			单位	消耗量	
人工	00010501	综合用工二类	工日	0.362	0.321
材料	8021000102	预拌水下混凝土 C30	m^3	1.2120	1.1615
	34000011	其他材料费 占材料费	%	1.00	1.00
机械	9909000004	履带式起重机 15t	台班	0.0267	0.0267
	99460004	其他机具费 占人工费	%	1.50	1.50

北京市住房和城乡建设委员会

工作内容：准备机具、浆液配置、压水泥浆等。　　　　**单位：**m³

编号				2-36
项目				灌注桩后注浆
				水泥浆
工料机名称			单位	消耗量
人工	00010501	综合用工二类	工日	0.895
材料	0401030001	普通硅酸盐水泥 P.O 42.5	kg	765.0000
	34000011	其他材料费 占材料费	%	1.00
机械	99050006	电动灌浆机	台班	0.1210
	9905000008	灰浆搅拌机 200L	台班	0.1000
	99460004	其他机具费 占人工费	%	1.50

北京市住房和城乡建设委员会

二、隧道内桩混凝土

工作内容:1.混凝土桩:垂直及洞内运输、浇捣、养护等。2.隧道内灌注桩后注水泥浆:垂直及洞内运输、准备机具、浆液配置、压水泥浆等。

单位:m^3

编号				2-37	2-38
项目				混凝土桩	隧道内灌注桩后注水泥浆
工料机名称			单位	消耗量	
人工	00010504	综合用工二类	工日	0.577	1.074
材料	0401030001	普通硅酸盐水泥 P.O 42.5	kg	-	765.0000
	8021000102	预拌水下混凝土 C30	m^3	1.1615	-
	34000011	其他材料费 占材料费	%	1.00	1.00
机械	9909000403	桥式起重机 10t	台班	0.0300	0.0435
	99310001	电动三轮车	台班	0.0500	0.1800
	99050006	电动灌浆机	台班	-	0.1452
	9905000008	灰浆搅拌机 200L	台班	-	0.1200
	99460004	其他机具费 占人工费	%	1.50	1.50

北京市住房和城乡建设委员会

三、地下连续墙混凝土

工作内容：1.清底置换：接缝清刷、吹气、搅拌吸泥、清底等。2.导墙混凝土：设置分隔板、混凝土浇筑、振捣、养护等。3.地下连续墙混凝土：混凝土浇捣、养护配合等。

单位：见表

编号				2-39	2-40	2-41
项目				清底置换	导墙混凝土	地下连续墙混凝土
				段	m^3	
工料机名称			单位	消耗量		
人工	00010501	综合用工二类	工日	7.218	0.323	0.331
材料	8021000806	预拌混凝土 C30	m^3	-	1.0150	-
	04090007	膨润土	kg	47.5600	-	-
	8021000102	预拌水下混凝土 C30	m^3	-	-	1.1615
	34000011	其他材料费 占材料费	%	1.00	1.00	1.00
机械	9944000006	泥浆泵 ϕ100mm	台班	0.9000	-	0.0250
	9943000004	电动空气压缩机 $3m^3$/min	台班	0.9000	-	-
	99050005	地下墙混凝土浇捣架	台班	-	-	0.0250
	9909000017	汽车式起重机 25t	台班	0.9000	-	-
	99050003	混凝土振捣器（插入式）	台班	-	0.0770	-
	9909000005	履带式起重机 25t	台班	-	-	0.0086
	99460004	其他机具费 占人工费	%	1.50	1.50	1.50

北京市住房和城乡建设委员会

四、冠梁与腰梁混凝土

工作内容：1.混凝土冠(圈)梁：混凝土浇筑、振捣、养护等。2.混凝土腰梁：混凝土浇筑、振捣、养护等。3.钢腰梁：制作、安装等。

单位：见表

编号				2-42	2-43	2-44
项目				混凝土冠(圈)梁	混凝土腰梁	钢腰梁
				m^3		t
工料机名称			单位	消耗量		
人工	00010501	综合用工二类	工日	0.273	0.287	1.960
材料	8021000806	预拌混凝土 C30	m^3	1.0150	1.0150	-
	01170001-1	工字钢 综合	kg	-	-	823.2000
	01290043	普通钢板 31 以上	kg	-	-	221.6000
	03130103	低碳钢焊条 J422 综合	kg	-	-	7.7851
	34000011	其他材料费 占材料费	%	1.00	1.00	1.00
机械	9909000017	汽车式起重机 25t	台班	-	-	0.0337
	9925000002	交流弧焊机 32kV·A	台班	-	-	1.1130
	99460004	其他机具费 占人工费	%	1.50	1.50	1.50

北京市住房和城乡建设委员会

五、凿除混凝土

工作内容:1.凿除桩头混凝土:凿除桩头混凝土、修平、清理、装运至附近地点堆放等。2.凿除隧道内桩头混凝土:凿除桩头混凝土、修平、清理、垂直及洞内运输等。3.凿除地下连续墙顶混凝土:凿除地下连续墙顶混凝土、修平、清理、装运至附近地点堆放等。

单位: m^3

编号				2-45	2-46	2-47
项目				凿除桩头混凝土	凿除隧道内桩头混凝土	凿除地下连续墙顶混凝土
工料机名称			单位	消耗量		
人工	00010501	综合用工二类	工日	1.274	–	1.274
	00010504	综合用工二类	工日	–	1.656	–
机械	99330001	风镐	台班	0.9952	0.9952	–
	9909000403	桥式起重机 10t	台班	–	0.0090	–
	99310001	电动三轮车	台班	–	0.0350	–
	9943000004	电动空气压缩机 $3m^3/min$	台班	0.9952	0.9952	0.4144
	99330003	手持式风动凿岩机	台班	–	–	0.4144
	99460004	其他机具费 占人工费	%	1.50	1.50	1.50

北京市住房和城乡建设委员会

第四节 护坡

一、护坡与台阶

工作内容：选修石料、砌筑、养护等。　　　　**单位：**m^3

编号				2-48	2-49	2-50	2-51
项目				护坡			
				干砌块石	干砌块石(灌浆)	浆砌块石	浆砌预制块
工料机名称			单位	消耗量			
人工	00010301	综合用工一类	工日	0.665	0.703	0.785	0.700
材料	04050033-2	碎石	kg	1749.0000	1749.0000	1729.5000	-
	8001000612	砌筑砂浆 DM10-MR	m^3	-	0.0150	0.3670	0.1300
	04270009	混凝土预制块	m^3	-	-	-	0.9100
	34000011	其他材料费 占材料费	%	1.00	1.00	1.00	1.00
机械	9905000303	干混砂浆搅拌机 200L	台班	-	0.0028	0.0690	0.0244
	99460004	其他机具费 占人工费	%	1.50	1.50	1.50	1.50

北京市住房和城乡建设委员会

工作内容：1.块石锥型坡、浆砌台阶：选修石料、砌筑、养护等。2.混凝土垫层：混凝土浇筑、振捣、养护等。　　**单位**：m^3

编号				2-52	2-53	2-54	2-55	2-56	2-57
项目				块石锥型坡		浆砌台阶			混凝土垫层
				浆砌	干砌	块石	粗料石	预制混凝土块	
工料机名称			单位	消耗量					
人工	00010301	综合用工一类	工日	0.965	1.126	1.325	1.670	1.182	-
	00010501	综合用工二类	工日	-	-	-	-	-	0.554
材料	04050033-2	碎石	kg	1729.5000	1749.0000	1729.5000	-	-	-
	8001000612	砌筑砂浆 DM10-MR	m^3	0.3670	-	0.3670	0.2200	0.1300	-
	04270009	混凝土预制块	m^3	-	-	-	-	0.9100	-
	04110002	料石	m^3	-	-	-	0.9100	-	-
	8021000802	预拌混凝土 C15	m^3	-	-	-	-	-	1.0200
	34000011	其他材料费 占材料费	%	1.00	1.00	1.00	1.00	1.00	1.00
机械	9905000303	干混砂浆搅拌机 200L	台班	0.0690	-	-	-	-	-
	99050004	混凝土振捣器(平板式)	台班	-	-	-	-	-	0.0770
	99460004	其他机具费 占人工费	%	1.50	1.50	1.50	1.50	1.50	1.50

北京市住房和城乡建设委员会

二、砂石垫层

工作内容：整理基层、垫层铺筑、夯实、捣固、整平、养护等。　　　　**单位：**m^3

编号				2-58	2-59	2-60	2-61	2-62	2-63
项目				碎石		毛石		级配砂石	砂
				干铺	灌浆	干铺	灌浆		
工料机名称			单位	消耗量					
人工	00010501	综合用工二类	工日	0.390	0.605	0.451	0.702	0.214	0.268
材料	8001000612	砌筑砂浆 DM10-MR	m^3	–	0.2500	–	0.2500	–	–
	04110003-2	毛石	t	–	–	1.8945	1.8945	–	–
	04050033-2	碎石	kg	1963.8000	1963.8000	–	–	–	–
	0403000003-2	砂子 中粗砂	kg	316.8000	–	316.8000	–	–	2032.0000
	04050011-2	级配砂石	kg	–	–	–	–	2315.4000	–
	34000011	其他材料费 占材料费	%	1.00	1.00	1.00	1.00	1.00	1.00
机械	9913000202	电动夯实机 20~62kg/m	台班	0.0230	0.0230	–	–	0.0220	0.0140
	9905000303	干混砂浆搅拌机 200L	台班	–	0.0280	–	0.0280	–	–
	99460004	其他机具费 占人工费	%	1.50	1.50	1.50	1.50	1.50	1.50

北京市住房和城乡建设委员会

第五节 明挖基坑与边坡支护

一、预应力锚索

工作内容：1.预应力锚索钻孔：孔点测定、钻孔机安装、移动和拆除、钻孔、清孔等。2.预应力锚索注浆：放置注浆管、搅拌灰浆、注浆等。

单位：m

编号				2-64	2-65	2-66	2-67
项目				预应力锚索钻孔(孔径)	预应力锚索注浆(孔径)	预应力锚索钻孔(孔径)	预应力锚索注浆(孔径)
				≤150mm		≤200mm	
工料机名称			单位	消耗量			
人工	00010501	综合用工二类	工日	0.121	0.030	0.142	0.035
材料	0401030004-1	水泥 42.5#	kg	-	49.5000	-	88.3125
	22450010	高压胶皮风管	m	0.9800	-	0.9800	-
	34000011	其他材料费 占材料费	%	1.00	-	1.00	-
机械	9903000302	锚杆钻孔机 DHR80A	台班	0.0146	-	0.0164	-
	9905000303	干混砂浆搅拌机 200L	台班	-	0.0070	-	0.0090
	99440008	高压注浆泵	台班	-	0.0070	-	0.0090
	99460004	其他机具费 占人工费	%	1.50	1.50	1.50	1.50

北京市住房和城乡建设委员会

工作内容:制作、安装、张拉等。 **单位:**m

编号				2-68	2-69	2-70	2-71	2-72	2-73
项目				预应力钢绞线 φ12.7					
				2 束	3 束	4 束	5 束	6 束	7 束
工料机名称			单位	消耗量					
人工	00010501	综合用工二类	工日	0.108	0.119	0.131	0.144	0.158	0.174
材料	01070001-1	钢绞线	kg	1.7910	2.6860	3.5820	4.4770	5.3730	6.2680
	34000011	其他材料费 占材料费	%	1.00	1.00	1.00	1.00	1.00	1.00
机械	99170008	钢筋切断机 φ40 以内	台班	0.0020	0.0020	0.0030	0.0030	0.0040	0.0040
	9917000101	预应力拉伸机 YCW-100	台班	0.0170	0.0190	0.0230	0.0250	0.0280	0.0310
	9944000009	高压油泵 80MPa	台班	0.0170	0.0190	0.0230	0.0250	0.0280	0.0310
	9943000012	立式油压千斤顶 200t	台班	0.0170	0.0190	0.0230	0.0250	0.0280	0.0310
	99460004	其他机具费 占人工费	%	1.50	1.50	1.50	1.50	1.50	1.50

北京市住房和城乡建设委员会

工作内容：制作、安装、张拉等。 单位：m

编号				2-74	2-75	2-76	2-77	2-78	2-79
项目				预应力钢绞线 ϕ15.2					
				2 束	3 束	4 束	5 束	6 束	7 束
工料机名称			单位	消耗量					
人工	00010501	综合用工二类	工日	0.119	0.131	0.144	0.158	0.174	0.191
材料	01070001-1	钢绞线	kg	2.5440	3.8160	5.0880	6.3600	7.6330	8.9050
	34000011	其他材料费 占材料费	%	1.00	1.00	1.00	1.00	1.00	1.00
机械	99170008	钢筋切断机 ϕ40 以内	台班	0.0020	0.0020	0.0030	0.0030	0.0040	0.0040
	9917000101	预应力拉伸机 YCW-100	台班	0.0180	0.0210	0.0250	0.0280	0.0310	0.0340
	9944000009	高压油泵 80MPa	台班	0.0180	0.0210	0.0250	0.0280	0.0310	0.0340
	9943000012	立式油压千斤顶 200t	台班	0.0180	0.0210	0.0250	0.0280	0.0310	0.0340
	99460004	其他机具费 占人工费	%	1.50	1.50	1.50	1.50	1.50	1.50

北京市住房和城乡建设委员会

工作内容：制作、安装、锚固、防锈处理等。　　　　　　　　**单位：**套

编号				2-80
项目				锚墩、承压板制作安装
工料机名称			单位	消耗量
人工	00010501	综合用工二类	工日	0.420
材料	0129001119	普通钢板 δ16mm~20mm	kg	11.5000
	03151102	锚具 综合	套	1.0200
	34000011	其他材料费 占材料费	%	1.00
机械	99460004	其他机具费 占人工费	%	1.50

北京市住房和城乡建设委员会

二、护坡土钉与锁脚锚管

工作内容：1.土钉制作、安装：制作、安装等。2.土钉钻孔、注浆：钻孔、浆液制作、压浆、土钉施工平台搭设、拆除等。　　**单位：**见表

编号				2-81	2-82	2-83
项目				土钉制作、安装		土钉钻孔、注浆
				钢筋	钢管	
				t		m
工料机名称			单位	消耗量		
人工	00010501	综合用工二类	工日	2.040	1.761	0.091
材料	0401030004-1	水泥 42.5#	kg	-	-	35.0000
	2245000002	高压胶皮风管 ϕ25-6P-20m	m	-	-	0.9800
	01010003	钢筋 ϕ10 以外	kg	1025.0000	-	-
	03130109	低合金钢焊条 E43 系列	kg	3.0000	2.1600	-
	17010001-2	焊接钢管 综合	kg	-	1020.0000	-
	34000011	其他材料费 占材料费	%	1.00	1.00	1.00
机械	9903000302	锚杆钻孔机 DHR80A	台班	-	-	0.0130
	99440008	高压注浆泵	台班	-	-	0.0090
	9905000303	干混砂浆搅拌机 200L	台班	-	-	0.4500
	9909000017	汽车式起重机 25t	台班	0.2800	0.2800	-
	9925000002	交流弧焊机 32kV·A	台班	0.2500	0.1800	-
	99460004	其他机具费 占人工费	%	1.50	1.50	1.50

北京市住房和城乡建设委员会

工作内容：锚管制作、垂直运输、布眼、钻孔、安放就位等。 **单位：**m

编号				2-84
项目				锁脚锚管
工料机名称			单位	消耗量
人工	00010501	综合用工二类	工日	0.104
材料	1701000020	焊接钢管 $DN32\times3.25$	kg	3.2200
	03151305	合金钢钻头	个	0.0250
	17090002	六角空心钢	kg	0.0770
	14290005-1	乙炔气	m^3	0.0200
	14290003	氧气	m^3	0.0650
	34000011	其他材料费 占材料费	%	1.00
机械	9943000204	空压机 $3m^3/min$	台班	0.0120
	99330001	风镐	台班	0.0280
	9919000008	立式钻床 25mm	台班	0.0040
	99230021	管子切断机 60~150	台班	0.0020
	9909000005	履带式起重机 25t	台班	0.0003
	99460004	其他机具费 占人工费	%	1.50

北京市住房和城乡建设委员会

三、基坑支护喷射混凝土

工作内容：修整边坡、喷射混凝土、找平面层等。 **单位：**m^2

编号				2-85	2-86	2-87	2-88
项目				喷射混凝土护坡			
				初喷厚 50mm		每增减 10mm	
				垂直面	斜面	垂直面	斜面
工料机名称			单位	消耗量			
人工	00010501	综合用工二类	工日	0.076	0.069	0.014	0.013
材料	8021001001	预拌喷射混凝土干料 C20	m^3	0.0600	0.0550	0.0120	0.0110
	17270008	耐压胶管	m	0.0186	0.0186	0.0007	0.0007
	34000011	其他材料费 占材料费	%	1.00	1.00	1.00	1.00
机械	9905000007	混凝土湿喷机 $5m^3/h$	台班	0.0062	0.0056	0.0002	0.0002
	9943000007	电动空气压缩机 $10m^3/min$	台班	0.0062	0.0056	0.0002	0.0002
	99460004	其他机具费 占人工费	%	1.50	1.50	1.50	1.50

北京市住房和城乡建设委员会

四、施工钢支撑与混凝土支撑

工作内容：1.钢支撑制作：下料、切割、焊接、油漆、整理堆放等。2.混凝土支撑梁：混凝土浇筑、振捣、养护等。　　**单位：**见表

编号				2-89	2-90	2-91
项目				钢支撑制作		混凝土支撑梁
				钢管	型钢	
				t		m^3
工料机名称			单位	消耗量		
人工	00010501	综合用工二类	工日	6.090	6.408	0.305
材料	17010018-2	钢管 综合	kg	1060.0000	-	-
	03130101	电焊条（综合）	kg	10.6250	12.4100	-
	05030007	板方材	m^3	0.0010	0.0010	-
	13010029	环氧富锌底漆	kg	5.4000	5.6820	-
	13050004	防锈漆	kg	2.3000	2.4201	-
	01000001-1	型钢 综合	kg	-	1060.0000	-
	8021000805	预拌混凝土 C25	m^3	-	-	1.0100
	34000011	其他材料费 占材料费	%	1.00	1.00	1.00
机械	9925000202	交流电焊机 32kV·A	台班	0.8850	1.0340	-
	9909000013	汽车式起重机 10t	台班	0.0250	0.0250	-
	99460004	其他机具费 占人工费	%	1.50	1.50	1.50

北京市住房和城乡建设委员会

工作内容：1.钢支撑安装及拆除(明挖)：定位、槽壁面凿出预埋件、钢牛腿焊接、支撑拼装、安装定位、活络接头固定、切割、吊运至地面、堆放及装车等。2.钢支撑安装及拆除(暗挖)：定位、槽壁面凿出预埋件、钢牛腿焊接、支撑拼装、安装定位、活络接头固定、切割、垂直及洞内运输、地面堆放及装车等。

单位：t

编号				2-92	2-93
项目				钢支撑安装及拆除	
				明挖	暗挖
工料机名称			单位	消耗量	
人工	00010501	综合用工二类	工日	2.382	-
	00010504	综合用工二类	工日	-	3.216
材料	03130101	电焊条（综合）	kg	0.6600	0.8750
	14290005-1	乙炔气	m^3	0.6000	0.7950
	14290003	氧气	m^3	1.8000	2.3850
	01290034	钢板(中厚)	kg	4.7500	6.3000
	37050002	沥青枕木	m^3	0.0200	0.0250
	0301050630	带母螺栓 M24×(110～150)	套	22.0000	22.0000
	34000011	其他材料费 占材料费	%	1.00	1.00
机械	9925000202	交流电焊机 32kV·A	台班	0.0550	0.0729
	9907000006	载重汽车 10t	台班	0.1000	-
	9943000007	电动空气压缩机 10m^3/min	台班	0.0600	0.0600
	9909000006	履带式起重机 40t	台班	0.0900	-
	9909000257	电动卷扬机 双筒慢速 5t	台班	-	0.8200
	9909000403	桥式起重机 10t	台班	-	0.1640
	99310001	电动三轮车	台班	-	0.4100
	9943000012	立式油压千斤顶 200t	台班	0.1300	0.1750
	99330001	风镐	台班	0.0600	0.0600
	99460004	其他机具费 占人工费	%	1.50	1.50

北京市住房和城乡建设委员会

第六节 暗挖与盖挖支护

一、小导管与袖阀管

工作内容：材料制作、材料垂直及洞内运输、布眼、钻孔、安放就位等。 **单位：**m

编		号		2-94	2-95
项		目		小导管	袖阀管
工料机名称			单位	消耗量	
人工	00010504	综合用工二类	工日	0.127	0.150
材料	1701000020	焊接钢管 $DN32\times3.25$	kg	3.2200	-
	03151305	合金钢钻头	个	0.0250	0.0300
	17090002	六角空心钢	kg	0.0770	0.0800
	14290005-1	乙炔气	m^3	0.0200	-
	14290003	氧气	m^3	0.0700	-
	17310045	塑料注浆阀管	m	-	1.0200
	34000011	其他材料费 占材料费	%	1.00	1.00
机械	9943000204	空压机 $3m^3/min$	台班	0.0120	-
	99330001	风镐	台班	0.0280	-
	9919000008	立式钻床 25mm	台班	0.0040	-
	99230021	管子切断机 60~150	台班	0.0020	-
	99310001	电动三轮车	台班	0.0020	0.0020
	9909000403	桥式起重机 10t	台班	0.0004	0.0004
	99030006	钻孔机	台班	-	0.0280
	99460004	其他机具费 占人工费	%	1.50	1.50

北京市住房和城乡建设委员会

二、大管棚

工作内容：材料垂直及洞内运输、定位、开孔、钻孔、管棚制作安装、封口钢板焊接、移机就位等。 **单位：**m

编号				2-96	2-97	2-98	2-99
项目				大管棚 $\phi \leq 130$			
				钻机成孔			
				$L \leq 20$m	$L \leq 40$m	$L \leq 60$m	$L \leq 80$m
工料机名称			单位	消耗量			
人工	00010504	综合用工二类	工日	0.363	0.399	0.439	0.483
材料	1707000129	无缝钢管 D108×4	m	1.0500	1.0500	1.0500	1.0500
	0315130714	钻头 ϕ115	个	0.0063	0.0057	0.0052	0.0047
	0315070004	镀锌铁丝 22#	kg	0.1900	0.1900	0.1900	0.1900
	03130101	电焊条（综合）	kg	0.4130	0.4220	0.4320	0.4420
	0129000807	钢板 δ4.5mm～12mm	kg	0.1738	0.0809	0.0387	0.0290
	34000011	其他材料费 占材料费	%	1.00	1.00	1.00	1.00
机械	99030007	水平定向钻机	台班	0.0450	0.0470	0.0490	0.0510
	9943000007	电动空气压缩机 $10m^3$/min	台班	0.0450	0.0470	0.0490	0.0510
	9925000202	交流电焊机 32kV·A	台班	0.0344	0.0351	0.0360	0.0370
	99230021	管子切断机 60～150	台班	0.0130	0.0130	0.0130	0.0130
	9909000014	汽车式起重机 12t	台班	0.0250	0.0260	0.0270	0.0280
	9909000107	电动双梁起重机 5t	台班	0.0210	0.0210	0.0210	0.0210
	9909000403	桥式起重机 10t	台班	0.0014	0.0014	0.0014	0.0014
	99310001	电动三轮车	台班	0.0020	0.0020	0.0020	0.0020
	99460004	其他机具费 占人工费	%	1.50	1.50	1.50	1.50

北京市住房和城乡建设委员会

工作内容：材料垂直及洞内运输、定位、开孔、钻孔、套管跟进成孔、管棚制作安装、封口钢板焊接、移机就位等。　　**单位：**m

编号				2-100	2-101	2-102	2-103
项目				大管棚 $\phi\leqslant200$			
				套管跟进成孔			
				$L\leqslant20$m	$L\leqslant40$m	$L\leqslant60$m	$L\leqslant80$m
工料机名称			单位	消耗量			
人工	00010504	综合用工二类	工日	0.449	0.494	0.544	0.598
材料	1707000135	无缝钢管 D159×4.5	m	1.0500	1.0500	1.0500	1.0500
	1829000503	钢套管 168×7	m	0.1750	0.1750	0.1750	0.1750
	03151308	扩孔钻头	个	0.0050	0.0070	0.0090	0.0110
	35170001	冲击器	个	0.0100	0.0110	0.0120	0.0130
	37090039	丝扣	个	0.1700	0.1700	0.1890	0.2040
	03151301-2	钻头	个	0.0070	0.0064	0.0058	0.0053
	0315070004	镀锌铁丝 22#	kg	0.2850	0.2850	0.2850	0.2850
	03130101	电焊条（综合）	kg	0.7970	0.8160	0.8350	0.8540
	0129000807	钢板 δ4.5mm~12mm	kg	0.4100	0.2050	0.1367	0.1026
	34000011	其他材料费 占材料费	%	1.00	1.00	1.00	1.00
机械	99030007	水平定向钻机	台班	0.0610	0.0640	0.0670	0.0700
	9943000007	电动空气压缩机 10m^3/min	台班	0.0610	0.0640	0.0670	0.0700
	9925000202	交流电焊机 32kV·A	台班	0.0660	0.0680	0.0700	0.0711
	99230020	管子切断机	台班	0.0170	0.0170	0.0170	0.0170
	9909000014	汽车式起重机 12t	台班	0.0310	0.0320	0.0330	0.0340
	9909000107	电动双梁起重机 5t	台班	0.0390	0.0400	0.0410	0.0420
	9909000403	桥式起重机 10t	台班	0.0026	0.0026	0.0026	0.0026
	99310001	电动三轮车	台班	0.0020	0.0020	0.0020	0.0020
	99460004	其他机具费 占人工费	%	1.50	1.50	1.50	1.50

北京市住房和城乡建设委员会

工作内容：材料垂直及洞内运输、定位、开孔、钻孔、施工平台及后背支撑搭设、管棚制作夯进、注浆管制作安装、封口钢板焊接、移机就位等。

单位：m

编号				2-104	2-105	2-106	2-107
项目				大管棚 $\phi\leq300$			
				夯管成孔			
				$L\leq20m$	$L\leq40m$	$L\leq60m$	$L\leq80m$
工料机名称			单位	消耗量			
人工	00010504	综合用工二类	工日	0.752	0.827	0.910	1.001
材料	1707000142	无缝钢管 D273×6	m	1.0500	1.0500	1.0500	1.0500
	14070002	润滑油	kg	1.1300	1.1870	1.2460	1.3080
	55270501	互锁 CIF	kg	9.5310	9.5310	9.5310	9.5310
	37090036	内切削环	个	0.0340	0.0200	0.0150	0.0100
	17010018-2	钢管 综合	kg	6.5690	6.5690	6.5690	6.5690
	0315070004	镀锌铁丝 22#	kg	0.4280	0.4280	0.4280	0.4280
	03130101	电焊条（综合）	kg	1.9580	1.9970	2.0350	2.0740
	0129000807	钢板 δ4.5mm~12mm	kg	0.8200	0.4100	0.2735	0.2051
	01000001-1	型钢 综合	kg	2.3560	2.3560	2.3560	2.3560
	05030007	板方材	m^3	0.0080	0.0080	0.0080	0.0080
	14290003	氧气	m^3	0.6750	0.6750	0.6750	0.6750
	14290005-1	乙炔气	m^3	0.2250	0.2250	0.2250	0.2250
	34000011	其他材料费 占材料费	%	1.00	1.00	1.00	1.00
机械	9903000403	气动夯管锤 TT350	台班	0.1020	0.1070	0.1120	0.1180
	9943000007	电动空气压缩机 10m^3/min	台班	0.1020	0.1070	0.1120	0.1180
	9903000116	钻机 TT40/60	台班	0.1020	0.1070	0.1120	0.1180
	9909000014	汽车式起重机 12t	台班	0.0310	0.0320	0.0330	0.0340
	9909000107	电动双梁起重机 5t	台班	0.5640	0.5750	0.5870	0.5990
	9909000403	桥式起重机 10t	台班	0.0376	0.0384	0.0392	0.0400
	9925000202	交流电焊机 32kV·A	台班	0.1630	0.1660	0.1700	0.1730
	9919001204	切管机 9A151	台班	0.0200	0.0200	0.0200	0.0200
	99310001	电动三轮车	台班	0.0030	0.0030	0.0030	0.0030
	99460004	其他机具费 占人工费	%	1.50	1.50	1.50	1.50

北京市住房和城乡建设委员会

工作内容：材料垂直及洞内运输、定位、开孔、钻孔、施工平台及后背支撑搭设、管棚制作夯进、注浆管制作安装、封口钢板焊接、移机就位等。

单位：m

编号				2-108	2-109	2-110	2-111
项目				大管棚 $\phi \leq 400$			
				夯管成孔			
				$L \leq 20$m	$L \leq 40$m	$L \leq 60$m	$L \leq 80$m
工料机名称			单位	消耗量			
人工	00010504	综合用工二类	工日	1.730	1.989	2.287	2.630
材料	1707000148	无缝钢管 D377×8	m	1.0500	1.0500	1.0500	1.0500
	14070002	润滑油	kg	1.5080	1.5830	1.6620	1.7450
	55270501	互锁 CIF	kg	12.1210	12.1210	12.1210	12.1210
	37090036	内切削环	个	0.0450	0.0270	0.0200	0.0130
	17010018-2	钢管 综合	kg	8.3540	8.3540	8.3540	8.3540
	0315070004	镀锌铁丝 22#	kg	0.6410	0.6410	0.6410	0.6410
	03130101	电焊条（综合）	kg	2.4480	2.4960	2.5440	2.5920
	0129000807	钢板 δ4.5mm～12mm	kg	1.6400	0.8200	0.5409	0.4102
	01000001-1	型钢 综合	kg	2.3560	2.3560	2.3560	2.3560
	05030007	板方材	m^3	0.0080	0.0080	0.0080	0.0080
	14290003	氧气	m^3	0.9120	0.9120	0.9120	0.9120
	14290005-1	乙炔气	m^3	0.3040	0.3040	0.3040	0.3040
	34000011	其他材料费 占材料费	%	1.00	1.00	1.00	1.00
机械	9903000403	气动夯管锤 TT350	台班	0.1210	0.1270	0.1330	0.1400
	9943000007	电动空气压缩机 $10m^3/min$	台班	0.1210	0.1270	0.1330	0.1400
	9903000116	钻机 TT40/60	台班	0.1210	0.1270	0.1330	0.1400
	9909000014	汽车式起重机 12t	台班	0.0410	0.0420	0.0430	0.0440
	9909000107	电动双梁起重机 5t	台班	0.6600	0.6730	0.6860	0.7000
	9909000403	桥式起重机 10t	台班	0.0440	0.0448	0.0456	0.0466
	9925000202	交流电焊机 32kV·A	台班	0.2040	0.2080	0.2120	0.2160
	9919001204	切管机 9A151	台班	0.0270	0.0280	0.0290	0.0300
	99310001	电动三轮车	台班	0.0040	0.0040	0.0040	0.0040
	99460004	其他机具费 占人工费	%	1.50	1.50	1.50	1.50

北京市住房和城乡建设委员会

工作内容：材料垂直及洞内运输、定位、开孔、钻孔、施工平台及后背支撑搭设、管棚制作夯进、注浆管制作安装、封口钢板焊接、移机就位等。

单位：m

编号				2-112	2-113	2-114	2-115
项目				大管棚 $\phi\leq500$			
				夯管成孔			
				$L\leq20$m	$L\leq40$m	$L\leq60$m	$L\leq80$m
工料机名称			单位	消耗量			
人工	00010504	综合用工二类	工日	3.570	3.630	4.175	4.801
材料	1707000180	无缝钢管 D478×9	m	1.0500	1.0500	1.0500	1.0500
	14070002	润滑油	kg	1.8850	1.9790	2.0780	2.1820
	55270501	互锁 CIF	kg	19.8810	19.8810	19.8810	19.8810
	37090036	内切削环	个	0.0560	0.0340	0.0250	0.0160
	17010018-2	钢管 综合	kg	13.1380	13.1380	13.1380	13.1380
	0315070004	镀锌铁丝 22#	kg	0.9620	0.9620	0.9620	0.9620
	03130101	电焊条（综合）	kg	2.7070	2.7650	2.8220	2.8800
	0129000807	钢板 δ4.5mm～12mm	kg	3.0750	1.5375	1.0255	0.7691
	01000001-1	型钢 综合	kg	4.1700	4.1700	4.1700	4.1700
	05030007	板方材	m^3	0.0200	0.0200	0.0200	0.0200
	14290003	氧气	m^3	1.2300	1.2300	1.2300	1.2300
	14290005-1	乙炔气	m^3	0.4100	0.4100	0.4100	0.4100
	34000011	其他材料费 占材料费	%	1.00	1.00	1.00	1.00
机械	9903000404	气动夯管锤 TT380	台班	0.1430	0.1500	0.1580	0.1660
	9943000007	电动空气压缩机 10m^3/min	台班	0.1430	0.1500	0.1580	0.1660
	9903000116	钻机 TT40/60	台班	0.1430	0.1500	0.1580	0.1660
	9909000014	汽车式起重机 12t	台班	0.0520	0.0530	0.0540	0.0550
	9909000107	电动双梁起重机 5t	台班	0.7800	0.7960	0.8120	0.8280
	9909000403	桥式起重机 10t	台班	0.0520	0.0530	0.0540	0.0550
	9925000202	交流电焊机 32kV·A	台班	0.2260	0.2300	0.2350	0.2400
	9919001204	切管机 9A151	台班	0.0330	0.0340	0.0350	0.0360
	99310001	电动三轮车	台班	0.0050	0.0050	0.0050	0.0050
	99460004	其他机具费 占人工费	%	1.50	1.50	1.50	1.50

北京市住房和城乡建设委员会

工作内容：材料垂直及洞内运输、定位、开孔、钻孔、施工平台及后背支撑搭设、管棚制作顶进、注浆管制作安装、封口钢板焊接、移机就位等。

单位：m

编号				2-116	2-117	2-118	2-119
项目				大管棚 $\phi \leqslant 600$			
				顶管成孔			
				$L \leqslant 20m$	$L \leqslant 40m$	$L \leqslant 60m$	$L \leqslant 80m$
工料机名称			单位	消耗量			
人工	00010504	综合用工二类	工日	5.761	6.625	7.619	8.762
材料	1707000154	无缝钢管 D600×12	m	1.0500	1.0500	1.0500	1.0500
	14070002	润滑油	kg	2.0570	2.1600	2.2680	2.3810
	55270501	互锁 CIF	kg	25.8580	25.8580	25.8580	25.8580
	37090036	内切削环	个	0.0670	0.0410	0.0300	0.0190
	17010018-2	钢管 综合	kg	17.0880	17.0880	17.0880	17.0880
	0315070004	镀锌铁丝 22#	kg	1.4430	1.4430	1.4430	1.4430
	03130101	电焊条（综合）	kg	3.4750	3.5420	3.6100	3.6860
	0129000807	钢板 δ4.5mm～12mm	kg	5.1250	2.5625	1.7092	1.2819
	01000001-1	型钢 综合	kg	4.1700	4.1700	4.1700	4.1700
	05030007	板方材	m^3	0.0200	0.0200	0.0200	0.0200
	14290003	氧气	m^3	1.6620	1.6620	1.6620	1.6620
	14290005-1	乙炔气	m^3	0.5540	0.5540	0.5540	0.5540
	34000011	其他材料费 占材料费	%	1.00	1.00	1.00	1.00
机械	99350002	顶管机	台班	0.2730	0.2870	0.3010	0.3160
	9943000007	电动空气压缩机 $10m^3/min$	台班	0.2730	0.2870	0.3010	0.3160
	9903000116	钻机 TT40/60	台班	0.2730	0.2870	0.3010	0.3160
	9909000014	汽车式起重机 12t	台班	0.0620	0.0630	0.0640	0.0650
	9909000107	电动双梁起重机 5t	台班	0.9000	0.9180	0.9360	0.9550
	9909000403	桥式起重机 10t	台班	0.0600	0.0612	0.0624	0.0636
	9925000202	交流电焊机 32kV·A	台班	0.2900	0.2950	0.3000	0.3070
	9919001204	切管机 9A151	台班	0.0400	0.0410	0.0420	0.0430
	99310001	电动三轮车	台班	0.0060	0.0060	0.0060	0.0060
	99460004	其他机具费 占人工费	%	1.50	1.50	1.50	1.50

北京市住房和城乡建设委员会

三、小导管、袖阀管和大管棚注浆

工作内容：材料垂直及洞内运输、准备及清理、浆液制作、注浆、检查、堵孔等。　　　　**单位：**m^3

编号				2-120	2-121	2-122	2-123
项目				水泥砂浆	水泥水玻璃双液浆	水泥浆	改性水玻璃浆
工料机名称			单位	消耗量			
人工	00010504	综合用工二类	工日	0.905	1.087	0.865	0.988
材料	8001000004	水泥砂浆 1∶3	m^3	1.0200	–	–	–
	05030007	板方材	m^3	0.0100	0.0100	0.0100	0.0100
	0401030004-1	水泥 42.5#	kg	–	441.0000	765.0000	–
	14310009	硅酸钠(水玻璃)	kg	–	390.0000	–	440.0000
	14310010	硫酸	kg	–	6.6000	–	70.0000
	14310008	磷酸氢二钠	kg	–	–	–	20.0000
	34000011	其他材料费 占材料费	%	1.00	1.00	1.00	1.00
机械	9905000009	灰浆搅拌机 400L	台班	0.1410	0.1600	0.1400	0.1600
	99050006	电动灌浆机	台班	0.1410	0.1600	0.1400	0.1600
	99310001	电动三轮车	台班	0.1500	0.1560	0.1520	0.1580
	9909000403	桥式起重机 10t	台班	0.0060	0.0062	0.0053	0.0055
	99460004	其他机具费 占人工费	%	1.50	1.50	1.50	1.50

北京市住房和城乡建设委员会

四、深孔注浆

工作内容：材料垂直及洞内运输、工作准备、钻机就位、钻孔、浆液制作、注浆、检查、清理等。　　**单位：**m³

编号				2-124
项目				深孔注浆
工料机名称			单位	消耗量
人工	00010504	综合用工二类	工日	0.893
材料	0401030004-1	水泥 42.5#	kg	382.5000
	14310009	硅酸钠(水玻璃)	kg	482.4498
	34000011	其他材料费 占材料费	%	1.00
机械	9903001115	液压钻机 G2A	台班	0.1816
	9905000009	灰浆搅拌机 400L	台班	0.1600
	9944001301	液压注浆泵 HYB-5	台班	0.2177
	99310001	电动三轮车	台班	0.0300
	9909000403	桥式起重机 10t	台班	0.0062
	99460004	其他机具费 占人工费	%	1.50

北京市住房和城乡建设委员会

五、隧道内锚杆

工作内容：1.砂浆锚杆：材料垂直及洞内运输、定位、钻孔、砂浆制作、灌浆、安装锚杆、固定等。2.自进式锚杆：材料垂直及洞内运输、定位、钻孔、安装锚杆、固定等。

单位：m

编号				2-125	2-126	2-127	2-128
项目				砂浆锚杆	自进式锚杆（杆体直径）		
					≤32mm	≤51mm	≤76mm
工料机名称			单位	消耗量			
人工	00010504	综合用工二类	工日	0.110	0.146	0.161	0.193
材料	01010003	钢筋 ϕ10 以外	kg	4.0055	–	–	–
	1727000108	高压胶管 ϕ50	m	0.0200	–	–	–
	8001000003	水泥砂浆 1∶2.5	m^3	0.0260	–	–	–
	03151305	合金钢钻头	个	0.0160	–	–	–
	17090002	六角空心钢	kg	0.0280	–	–	–
	0315110901	中空注浆锚杆 R32N	m	–	1.0200	–	–
	0315000009	连接套 ϕ32	套	–	0.3400	–	–
	0315110902	中空注浆锚杆 R51N	m	–	–	1.0200	–
	0315000010	连接套 ϕ51	套	–	–	0.3400	–
	0315110903	中空注浆锚杆 R76N	m	–	–	–	1.0200
	0315000011	连接套 ϕ76	套	–	–	–	0.3400
	0315130201	十字合金钻头及后配套 ϕ32	套	–	0.1750	–	–
	0315130202	十字合金钻头及后配套 ϕ51	套	–	–	0.1750	–
	0315130203	十字合金钻头及后配套 ϕ76	套	–	–	–	0.1750
	34000011	其他材料费 占材料费	%	1.00	1.00	1.00	1.00
机械	9933000101	凿岩机 气腿 YTP26	台班	0.0240	–	–	–
	9905000008	灰浆搅拌机 200L	台班	0.0030	–	–	–
	99050006	电动灌浆机	台班	0.0050	–	–	–
	99170007	钢筋切断机	台班	0.0100	–	–	–
	9943000007	电动空气压缩机 $10m^3/min$	台班	0.0120	–	–	–
	99310001	电动三轮车	台班	0.0020	0.0020	0.0030	0.0040
	9909000403	桥式起重机 10t	台班	0.0002	0.0002	0.0004	0.0006
	9903000114	水平钻机 SH-1030	台班	–	0.0200	0.0300	0.0420
	99460004	其他机具费 占人工费	%	1.50	1.50	1.50	1.50

北京市住房和城乡建设委员会

第七节 钢筋

一、桩

工作内容：1.钢筋笼制作安装：钢筋加工、绑扎、焊接、材料成品现场水平搬运、吊装就位等。2.声测管埋设：制作、对接、预埋布设、固定安装等。3.注浆管埋设：注浆管制作、焊接、埋设安装等。

单位：见表

编号				2-129	2-130	2-131	2-132
项目				钢筋笼制作安装(孔深)		声测管埋设	注浆管埋设
				≤30m	≤50m		
				t		m	
工料机名称			单位	消耗量			
人工	00010301	综合用工一类	工日	6.409	6.729	0.010	0.012
材料	01010002-1	钢筋 ϕ10 以内	kg	162.0000	162.0000	–	–
	01010003	钢筋 ϕ10 以外	kg	868.0000	868.0000	–	–
	03130101	电焊条（综合）	kg	8.5345	8.7052	0.0150	0.0150
	03150710	镀锌铁丝 8#～12#	kg	2.9200	2.9200	–	–
	1701000444	钢管 D60×3.5	m	–	–	1.0600	–
	18250015	接头管箍	个	–	–	0.1550	0.1550
	1707000105	无缝钢管 D32×2.5	m	–	–	–	1.0600
	19410012	注浆阀	个	–	–	–	0.0517
	01210007	角钢	kg	–	–	–	0.3660
	34000011	其他材料费 占材料费	%	1.00	1.00	1.00	1.00
机械	9909000030	履带式起重机 50t	台班	0.1532	0.1838	–	–
	99170005	钢筋调直机	台班	0.3900	0.3900	–	–
	99170007	钢筋切断机	台班	0.5400	0.5400	–	–
	99170009	钢筋弯曲机 ϕ40 内	台班	0.3200	0.3200	–	–
	9925000202	交流电焊机 32kV·A	台班	0.7110	0.7254	0.0025	0.0025
	9923000021	管子切断机 250mm	台班	–	–	0.0011	0.0011
	99460004	其他机具费 占人工费	%	1.50	1.50	1.50	1.50

北京市住房和城乡建设委员会

二、隧道内桩

工作内容：1.隧道内钢筋笼制作安装：钢筋制作、绑扎、焊接成形、吊装、垂直及洞内运输、对接、校正等。2.声测管埋设：制作、对接、垂直及洞内运输、预埋布设、固定安装等。3.注浆管埋设：注浆管制作、焊接、垂直及洞内运输、埋设安装等。 **单位：**见表

编号				2-133	2-134	2-135	2-136
项目				隧道内钢筋笼制作安装（孔深）		声测管埋设	注浆管埋设
				≤30m	≤50m		
				t		m	
工料机名称			单位	消耗量			
人工	00010304	综合用工一类	工日	10.477	11.001	0.020	0.018
材料	01010002-1	钢筋 ϕ10 以内	kg	162.0000	162.0000	–	–
	01010003	钢筋 ϕ10 以外	kg	868.0000	877.7748	–	–
	03130101	电焊条（综合）	kg	8.6500	8.8230	–	–
	1701000005-1	焊接钢管 *DN*40	m	–	–	1.0600	–
	18250023	管箍	个	–	–	0.6000	–
	03150710	镀锌铁丝 8#~12#	kg	2.9200	2.9200	–	–
	1707000105	无缝钢管 D32×2.5	m	–	–	–	1.0600
	18250015	接头管箍	个	–	–	0.1550	0.1550
	19410012	注浆阀	个	–	–	–	0.0517
	01210007	角钢	kg	–	–	–	0.3660
	1803001617	钢制丝堵 *DN*32	个	–	–	–	0.0517
	34000011	其他材料费 占材料费	%	1.00	1.00	1.00	1.00
机械	99170005	钢筋调直机	台班	0.3900	0.3900	–	–
	99170007	钢筋切断机	台班	0.5400	0.5400	–	–
	99170009	钢筋弯曲机 ϕ40 内	台班	0.3200	0.3200	–	–
	9925000202	交流电焊机 32kV·A	台班	0.7200	0.7350	–	–
	9909000403	桥式起重机 10t	台班	0.0502	0.0502	0.0004	0.0004
	99310001	电动三轮车	台班	0.1704	0.1704	0.0020	0.0020
	9909000302	龙门起重机 30t	台班	0.1500	0.1800	–	–
	9923000021	管子切断机 250mm	台班	–	–	0.0017	0.0017
	99460004	其他机具费 占人工费	%	1.50	1.50	1.50	1.50

北京市住房和城乡建设委员会

三、地下连续墙

工作内容：1.导墙钢筋：制作、运输、入模安装、绑扎、焊接等。2.型钢制作安装：加工成型、点焊、场内运输、吊运安装等。 **单位：**t

编号				2-137	2-138
项目				导墙钢筋	型钢制作安装
工料机名称			单位	消耗量	
人工	00010301	综合用工一类	工日	7.452	7.398
材料	01000001-1	型钢 综合	kg	-	1060.0000
	03130101	电焊条（综合）	kg	7.2000	21.4840
	01010003	钢筋 ϕ10 以外	kg	1050.0000	-
	03150710	镀锌铁丝 $8^{\#}\sim12^{\#}$	kg	5.8700	-
	34000011	其他材料费 占材料费	%	1.00	1.00
机械	9925000202	交流电焊机 32kV·A	台班	1.2000	3.8364
	9923000024	型钢剪断机 500mm	台班	-	0.6200
	9909000006	履带式起重机 40t	台班	-	0.2000
	9909000032	履带式起重机 60t	台班	-	0.0660
	99170007	钢筋切断机	台班	0.3800	-
	99170009	钢筋弯曲机 ϕ40 内	台班	0.5700	-
	99170005	钢筋调直机	台班	0.3900	-
	99460004	其他机具费 占人工费	%	1.50	1.50

北京市住房和城乡建设委员会

工作内容：钢筋切断、绑扎成型、焊接、场内运输、吊装就位等。　　　　单位：t

编号				2-139	2-140	2-141	2-142
项目				连续墙钢筋制作安装(槽深)			
				≤25m	≤35m	≤45m	≤55m
工料机名称			单位	消耗量			
人工	00010301	综合用工一类	工日	6.872	7.088	7.116	7.546
材料	03130101	电焊条（综合）	kg	10.2550	10.3722	10.4894	10.6048
	01010003	钢筋 ϕ10 以外	kg	1050.0000	1050.0000	1050.0000	1050.0000
	03150710	镀锌铁丝 8#～12#	kg	1.6800	1.6800	1.6800	1.6800
	34000011	其他材料费 占材料费	%	1.00	1.00	1.00	1.00
机械	9925000202	交流电焊机 32kV·A	台班	1.7500	1.7700	1.7900	1.8100
	9909000006	履带式起重机 40t	台班	0.2000	–	–	–
	9909000032	履带式起重机 60t	台班	0.0660	0.1600	–	–
	99170007	钢筋切断机	台班	0.1200	0.1200	0.1200	0.1200
	99170009	钢筋弯曲机 ϕ40 内	台班	0.3900	0.3900	0.3900	0.3900
	99170005	钢筋调直机	台班	0.1200	0.1200	0.1200	0.1200
	9909000007	履带式起重机 100t	台班	–	0.0600	0.1200	0.1504
	9909000008	履带式起重机 200t	台班	–	–	0.0467	–
	9909000009	履带式起重机 300t	台班	–	–	–	0.0585
	99460004	其他机具费 占人工费	%	1.50	1.50	1.50	1.50

北京市住房和城乡建设委员会

四、钢筋网制作安装

工作内容：钢筋网制作、挂网、连接固定等。　　**单位：**t

编号				2-143
项目				钢筋网制作安装
工料机名称			单位	消耗量
人工	00010301	综合用工一类	工日	4.950
材料	01010002-1	钢筋 φ10 以内	kg	1025.0000
	03130101	电焊条（综合）	kg	4.3300
	34000011	其他材料费 占材料费	%	1.00
机械	99170007	钢筋切断机	台班	0.1000
	99170005	钢筋调直机	台班	0.1800
	9925000202	交流电焊机 32kV·A	台班	0.8660
	99460004	其他机具费 占人工费	%	1.50

北京市住房和城乡建设委员会

第三章　地基加固工程

北京市住房和城乡建设委员会

北京市住房和城乡建设委员会

说　　明

一、本章包括:地基处理,地基加固 2 节共 25 个子目。

二、本章子目中不包括复合地基检测、变形监测等费用,发生时另行计算。

三、换填项目适用于路基部分的地基处理,如基坑换填执行本章子目,其人工和机械消耗量乘以系数 1.1。

四、分层注浆、压密注浆、高压旋喷子目中使用的注浆材料与设计不同时,可调整。

五、深层水泥搅拌桩空桩部分按相应子目的人工、机械乘以系数 0.50 执行。

六、本章凿水泥桩桩头适用于水泥粉煤灰碎石桩、高压旋喷水泥桩和水泥搅拌桩。

七、高压旋喷桩、水泥搅拌桩泥浆外运执行第一章相应子目。

工程量计算规则

一、换填砂、石屑、块石、灰土、水泥稳定土均按设计图示尺寸以体积计算。

二、水泥粉煤灰碎石桩按设计桩截面面积乘以设计桩长,以体积计算。

三、夯填桩按设计桩截面乘以桩长以体积计算。

四、铺土工布、土工格栅按设计图示尺寸以面积计算。

五、分层注浆、压密注浆按设计图示以加固土体体积计算。

六、高压旋喷水泥桩成孔按设计桩长计算;喷浆按设计桩截面面积乘以桩长以体积计算。

七、水泥搅拌桩按设计桩截面面积乘以桩长,以体积计算。

北京市住房和城乡建设委员会

八、凿桩头按凿桩长度乘以桩截面面积以体积计算。

九、高压旋喷桩和水泥搅拌桩成孔泥浆量按设计桩体积乘以系数 0.1 以体积计算。

北京市住房和城乡建设委员会

第一节 地基处理

一、换填砂、石屑、块石

工作内容：挖土、掺料改换、整平、分层夯实、找平、清理杂物等。 **单位：**m³

编号				3-1	3-2	3-3
项目				换填砂	换填石屑	换填块石
工料机名称			单位	消耗量		
人工	00010501	综合用工二类	工日	0.214	0.314	0.336
材料	0403000003-2	砂子 中粗砂	kg	1854.2000	-	-
	04070010	石屑	kg	-	2413.0000	-
	04050033-2	碎石	kg	-	-	1789.5000
	34000011	其他材料费 占材料费	%	1.00	1.00	1.00
机械	9913000202	电动夯实机 20~62kg/m	台班	0.0750	0.0800	0.0850
	99460004	其他机具费 占人工费	%	1.50	1.50	1.50

北京市住房和城乡建设委员会

二、换填灰土

工作内容：1.人工换填：挖土、掺料改换、整平、分层夯实、找平、清理杂物等。2.机械换填：机械挖土、掺料、分层排压、找平、碾压、清理杂物等。

单位：m^3

编号				3-4	3-5	3-6	3-7
项目				人工换填		机械换填	
				含灰量 5%	含灰量 8%	含灰量 5%	含灰量 8%
工料机名称			单位	消耗量			
人工	00010501	综合用工二类	工日	0.330	0.336	0.079	0.108
材料	04090013	白灰	kg	85.0000	136.0000	85.0000	136.0000
	04090008	素土	m^3	(1.4200)	(1.3750)	(1.4200)	(1.3750)
	34000011	其他材料费 占材料费	%	1.00	1.00	1.00	1.00
机械	9913000202	电动夯实机 20~62kg/m	台班	0.0800	0.0800	–	–
	9907000502	履带式推土机 75kW	台班	–	–	0.0150	0.0150
	99130002	光轮压路机(综合)	台班	–	–	0.0170	0.0170
	99460004	其他机具费 占人工费	%	1.50	1.50	1.50	1.50

北京市住房和城乡建设委员会

三、水泥稳定土

工作内容：运料、上料、人工或人机配合摊铺土方、拌和、找平、碾压等。 **单位：**m^3

编号				3-8	3-9
项目				水泥稳定土	
				人工拌和	人机配合
工料机名称			单位	消耗量	
人工	00010501	综合用工二类	工日	0.500	0.192
材料	04011101	水泥(综合)	kg	86.6670	86.6670
	04090008	素土	m^3	(1.4200)	(1.4200)
	34000011	其他材料费 占材料费	%	1.00	1.00
机械	99130002	光轮压路机(综合)	台班	0.0070	0.0070
	9907000502	履带式推土机 75kW	台班	–	0.0200
	9913000301	平地机 HP90	台班	–	0.0070
	99460004	其他机具费 占人工费	%	1.50	1.50

北京市住房和城乡建设委员会

四、水泥粉煤灰碎石桩

工作内容:准备机具,移动钻机,钻孔,校正;拌合,灌注,提管移位,操作范围内料具搬运等。 **单位:**m^3

编号				3-10	3-11
项目				水泥粉煤灰碎石桩(桩径)	
				≤600mm	≤800mm
工料机名称			单位	消耗量	
人工	00010501	综合用工二类	工日	0.353	0.336
材料	04090037	水泥粉煤灰碎石混合料	m^3	1.1730	1.1730
	34000011	其他材料费 占材料费	%	1.00	1.00
机械	9903000108	螺旋钻机 800mm	台班	-	0.0921
	9903000107	螺旋钻机 600mm	台班	0.0986	-
	99460004	其他机具费 占人工费	%	1.50	1.50

北京市住房和城乡建设委员会

五、夯填桩

工作内容：1.夯填混凝土：准备机具、锤击成孔、提护筒、孔深检测、填料、分层夯实、桩端夯扩、低锤密击、挤扩混凝土成桩等。2. 夯填碎石：准备机具、锤击成孔、提护筒、孔深检测、填料、分层夯实、桩端夯扩、低锤密击、挤扩碎石成桩等。

单位：m^3

编号				3-12	3-13
项目				夯填混凝土	夯填碎石
工料机名称			单位	消耗量	
人工	00010501	综合用工二类	工日	0.578	0.598
材料	03151101	钢护筒	t	0.0050	0.0050
	05030010	方木	m^3	0.0040	0.0040
	8021000806	预拌混凝土 C30	m^3	1.2100	–
	1503010001	岩棉板 δ30mm	m^2	0.2040	0.2040
	04050033-2	碎石	kg	–	1905.0000
	34000011	其他材料费 占材料费	%	1.00	1.00
机械	9907000701	轮胎式装载机 $1m^3$	台班	0.0638	0.0638
	9903001118	振动沉管钻机 ZTJ-4	台班	0.0720	0.0720
	99460004	其他机具费 占人工费	%	1.50	1.50

北京市住房和城乡建设委员会

六、铺土工织物

工作内容:清理整平路基、铺设土工布或土工格栅、缝合及锚固等。 **单位:**m^2

编号				3-14	3-15
项目				铺土工布<br>软土	铺土工格栅
工料机名称			单位	消耗量	
人工	00010501	综合用工二类	工日	0.032	0.046
材料	02310002	土工布	m^2	1.1150	–
	36030001	土工格栅	m^2	–	1.1000
	34000011	其他材料费 占材料费	%	1.00	1.00
机械	99460004	其他机具费 占人工费	%	1.50	1.50

北京市住房和城乡建设委员会

七、凿桩头

工作内容：桩头凿除、清理等。 **单位：**m^3

编号				3-16
项目				凿水泥桩桩头
工料机名称			单位	消耗量
人工	00010501	综合用工二类	工日	0.221
机械	99330003	手持式风动凿岩机	台班	0.2180
	9943000004	电动空气压缩机 $3m^3/min$	台班	0.2180
	99460004	其他机具费 占人工费	%	1.50

北京市住房和城乡建设委员会

第二节 地基加固

一、分层注浆与压密注浆

工作内容:1.分层注浆:定位、钻孔、泥浆护壁、放置注浆阀管、配置浆液、插注浆芯管、分层注浆、检测注浆效果等。2.压密注浆:定位、钻孔、泥浆护壁、配置浆液、安插注浆管、分段压密注浆、检测注浆效果等。

单位:m^3

编		号		3-17	3-18
项		目		分层注浆	压密注浆
工料机名称			单位	消耗量	
人工	00010501	综合用工二类	工日	0.728	0.617
材料	0401030004-1	水泥 42.5#	kg	109.3500	101.2400
	04090003-1	粉煤灰	kg	80.0800	68.0600
	04090007	膨润土	kg	39.7380	35.3290
	14310009	硅酸钠(水玻璃)	kg	5.6700	8.0000
	1435000029	表面活性剂 KA	kg	10.3000	-
	17310045	塑料注浆阀管	m	3.5390	-
	1731001906	注浆导管 ϕ12	m	-	3.5390
	34000011	其他材料费 占材料费	%	1.00	1.00
机械	9903001115	液压钻机 G2A	台班	0.1350	0.1130
	9944000005	泥浆泵 ϕ50mm	台班	0.1350	0.1130
	9905000008	灰浆搅拌机 200L	台班	0.2060	0.1724
	9944001301	液压注浆泵 HYB-5	台班	0.2060	0.1724
	99460004	其他机具费 占人工费	%	1.50	1.50

北京市住房和城乡建设委员会

二、高压旋喷水泥桩

工作内容：1.钻孔：定位、钻孔等。2.喷浆：配置浆液、接管喷浆、提升成桩等。 **单位：**见表

编号				3-19	3-20	3-21	3-22
项目				旋喷桩成孔	旋喷桩喷浆		
					单管法	双管法	三管法
				m	m^3		
工料机名称			单位	消耗量			
人工	00010501	综合用工二类	工日	0.022	0.241	0.321	0.398
材料	0401030004-1	水泥 42.5#	kg	–	255.0000	306.0000	464.1000
	34000011	其他材料费 占材料费	%	1.00	1.00	1.00	1.00
机械	9903001113	地质钻机 150 型	台班	0.0120	–	–	–
	9905000303	干混砂浆搅拌机 200L	台班	–	0.0300	0.0400	0.0500
	99440008	高压注浆泵	台班	–	0.0300	0.0300	0.0300
	99030001	单重管旋喷机	台班	–	0.0300	–	–
	9943000004	电动空气压缩机 $3m^3/min$	台班	–	–	0.0400	0.0500
	99030002	双重管旋喷机	台班	–	–	0.0400	–
	99030003	三重管旋喷机	台班	–	–	–	0.0500
	99440001	电动多级离心清水泵 ϕ100mm 120m 以下	台班	–	–	–	0.0500
	99460004	其他机具费 占人工费	%	1.50	1.50	1.50	1.50

北京市住房和城乡建设委员会

三、水泥搅拌桩

工作内容：定位钻进、喷浆、搅拌、提升、调制水泥浆、输送、压浆等。 **单位：**m^3

编号				3-23	3-24	3-25
项目				水泥搅拌桩		
				粉喷桩(水泥掺量13%)	浆喷桩(水泥掺量13%)	水泥掺量每增加1%
工料机名称			单位	消耗量		
人工	00010501	综合用工二类	工日	0.329	0.236	-
材料	0401030004-1	水泥 42.5#	kg	238.7000	238.7000	18.3600
	34000011	其他材料费 占材料费	%	1.00	1.00	1.00
机械	9905000303	干混砂浆搅拌机 200L	台班	-	0.0592	-
	99030015	粉喷桩机	台班	0.0592	0.0592	-
	9943000004	电动空气压缩机 $3m^3/min$	台班	0.0592	-	-
	9915000002	偏心式振动筛 $16m^3/h$	台班	0.0200	-	-
	99440008	高压注浆泵	台班	-	0.0300	-
	99460004	其他机具费 占人工费	%	1.50	1.50	-

北京市住房和城乡建设委员会

第四章　主体结构混凝土与钢筋工程

北京市住房和城乡建设委员会

北京市住房和城乡建设委员会

说　　明

一、本章包括:高架结构工程,明挖结构工程,暗挖与盖挖结构工程,钢筋工程,疏散平台,结构拆除 6 节共 187 个子目。

二、本章预制构件安装子目均不含构件场外运输的消耗量,其费用应包含在预制构件价格中。

三、明挖结构工程

1.喷射混凝土按 C20 预拌喷射混凝土干料编制,设计强度等级不同时,可调整。

2.混凝土楼梯包括休息平台、平台梁、斜梁及楼梯与楼板连接的梁、踏步板及踏步。

3.竖井钢筋执行明挖工程钢筋相应子目。

四、暗挖与盖挖结构工程:

1.暗挖和盖挖喷射混凝土不区分部位综合编制。

2.暗挖(盖挖)桩间喷射混凝土执行暗挖与盖挖喷射混凝土矩形隧道子目。

3.盖挖顶板混凝土执行本章明挖结构顶板子目。

五、钢筋工程

1.现浇构件的钢筋按绑扎和焊接综合编制,如设计采用机械连接时,执行本章第四节“直螺纹套筒接头”相应子目。

2.后张法钢筋(钢绞线)子目包含锚具安装工作内容,但不包括锚具消耗量,锚具应按设计型号和数量列入相应子目并乘以系数 1.02。

3.钢格栅中螺栓已综合编制,重量不另计算。

4.直螺纹套筒接头和植筋子目中 ϕ 均指设计图纸标明的钢筋直径。

北京市住房和城乡建设委员会

5.钢筋网片搭接数量按设计(规范)要求计算。

6.植筋锚固深度按 15d(d 为钢筋直径)编制,设计不同时执行增减钢筋锚固深度调整子目。

六、疏散平台钢面板按 1m×1m×0.04m 规格编制。

七、结构拆除:

1.拆除混凝土子目不包含拆除后渣土外运和消纳,应执行本册第一章石方(碴)、渣土装车及运输子目。

2.水钻开孔区分砌体和混凝土结构。

工程量计算规则

一、高架结构工程

1.现浇和预制混凝土结构(基础、垫层、承台、墩柱、桥台、台帽、挡土墙、盖梁、梁、板、接头及灌缝、桥头搭板等)均按设计图示尺寸以体积计算,不扣除钢筋、铁件、预留压浆孔道和螺栓所占体积;预制空心构件应按设计图示尺寸扣除空心部分体积。

2.桥头搭板橡胶板按设计图示尺寸以面积计算。

3.板式橡胶支座按设计规格以体积计算。

4.盆式橡胶支座按设计图示以数量计算。

5.压浆管道按设计图示尺寸以长度计算;压水泥浆按设计孔道断面面积乘以孔道长度以体积计算。

6.安装混凝土挂板按设计图示尺寸以长度计算。

7.混凝土栏杆、扶手和防撞墩按设计图示尺寸以体积计算。

8.隔声屏障钢骨架按设计图示尺寸乘理论重量以质量计算。

9.隔声屏障板材按设计图示尺寸以面积计算。

北京市住房和城乡建设委员会

10.伸缩缝按设计图示尺寸以长度计算。

二、明挖结构工程

1.混凝土柱按设计图示截面面积乘以高度以体积计算。

(1)柱高自柱基上表面(或板上表面)至上一层板或梁的下表面之间的高度计算。

(2)依附柱上的牛腿和柱帽,并入柱身体积计算。

2.混凝土梁按设计图示尺寸以体积计算。

(1)梁与柱连接时,梁长算至柱的内侧面;伸入墙内部分的梁头并入梁的体积计算。

(2)主梁与次梁连接时,次梁长度算至主梁的内侧面。

(3)梁高自梁底算至板底,反梁自板顶算至梁顶。

(4)下翻地梁算入底板内,上翻地梁按梁单算。

3.混凝土板按设计图示尺寸以体积计算,靠墙的倒角并入墙混凝土计算,不靠墙的倒角并入相邻板混凝土计算。

4.混凝土墙按设计图示尺寸以体积计算。

(1)墙的体积按墙的设计中心线长度乘以墙高和厚度计算。

(2)墙垛(附墙柱)、暗柱、暗梁及墙突出部分并入墙混凝土计算。

(3)墙的体积中,板与墙相叠加部位按墙计算。

5.现浇混凝土不扣除构件内钢筋、预埋铁件及单孔面积≤$0.3m^2$ 的孔洞所占体积。

6.混凝土楼梯按设计图示尺寸以体积计算。

三、暗挖与盖挖结构工程

1.喷射混凝土按设计图示尺寸以体积计算。

2.结构混凝土按设计图示尺寸以体积计算,不扣除构件内钢筋、预埋铁件及单孔面积≤$0.3m^2$ 的孔洞所占体积。

北京市住房和城乡建设委员会

3.钢管柱预拌混凝土和封口预拌混凝土按设计图示尺寸以体积计算。

四、钢筋工程

1.现浇构件的钢筋、钢筋网片、钢筋笼均按设计图示钢筋(网)长度乘以单位理论质量计算。现浇构件中伸出构件的锚固钢筋应并入钢筋工程量内。

2.明挖结构工程钢筋及钢构件按设计图示尺寸乘理论重量以质量计算。

3.暗挖和盖挖结构工程钢筋、钢格栅和钢筋网片均按设计图示尺寸乘理论重量以质量计算。

4.先张法和后张法预应力钢筋(钢绞线)按设计图示长度(应包括工作长度)乘理论重量以质量计算。

5.直螺纹套筒接头按设计图示以数量计算。

6.植筋按设计图示以数量计算。

五、疏散平台

1.混凝土定型化学锚栓安装按设计图示数量计算。

2.水泥基复合材料疏散平台支架按设计图示数量以套计算;钢结构材料疏散平台安装、爬梯安装按设计图示乘理论重量以质量计算。

3.疏散平台面板按设计图示尺寸以面积计算。

4.疏散平台扶手按设计图示尺寸以长度计算。

5.疏散平台导向标识按设计图示以数量计算。

六、结构拆除

1.拆除混凝土按设计图示尺寸以体积计算。

2.水钻开孔按设计图示尺寸以开孔深度计算。

北京市住房和城乡建设委员会

第一节 高架结构工程

一、现浇混凝土

工作内容：混凝土浇筑、振捣、抹平、养护等。 **单位：**m^3

编号				4-1	4-2	4-3
项目				垫层	基础	承台
工料机名称			单位	消耗量		
人工	00010501	综合用工二类	工日	0.259	0.326	0.342
材料	8021000803	预拌混凝土 C20	m^3	1.0100	–	–
	8021000807	预拌混凝土 C35	m^3	–	1.0100	1.0100
	34000011	其他材料费 占材料费	%	1.00	1.00	1.00
机械	99050003	混凝土振捣器(插入式)	台班	–	0.0770	0.0780
	99460004	其他机具费 占人工费	%	1.50	1.50	1.50

北京市住房和城乡建设委员会

工作内容：混凝土浇筑、振捣、抹平、养护等。　　　　　　　　　　　　　　　　　　　　　　　　**单位：**m^3

编号				4-4	4-5	4-6	4-7	4-8	4-9
项目				整体式墩台	柱式墩台	V 型、Y 型墩	轻型桥台	重力式墩台	墩台帽
工料机名称			单位	消耗量					
人工	00010501	综合用工二类	工日	0.493	0.615	0.688	0.730	0.604	0.604
材料	8021000807	预拌混凝土 C35	m^3	1.0100	1.0100	1.0100	1.0100	1.0100	1.0100
	34000011	其他材料费 占材料费	%	1.00	1.00	1.00	1.00	1.00	1.00
机械	99050003	混凝土振捣器(插入式)	台班	0.1100	0.1220	0.1240	0.1320	0.1000	0.1000
	99460004	其他机具费 占人工费	%	1.50	1.50	1.50	1.50	1.50	1.50

北京市住房和城乡建设委员会

工作内容：混凝土浇筑、振捣、抹平、养护等。 **单位：**m^3

编号				4-10	4-11	4-12	4-13	4-14
项目				挡土墙		预拌片石混凝土	盖梁	预应力盖梁
				高度≤3m	高度>3m			
工料机名称			单位	消耗量				
人工	00010501	综合用工二类	工日	0.450	0.476	0.259	0.677	0.766
材料	8021000805	预拌混凝土 C25	m^3	1.0100	1.0100	–	–	–
	8021000803	预拌混凝土 C20	m^3	–	–	0.8630	–	–
	04050033-2	碎石	kg	–	–	364.5000	–	–
	8021000808	预拌混凝土 C40	m^3	–	–	–	1.0100	1.0100
	34000011	其他材料费 占材料费	%	1.00	1.00	1.00	1.00	1.00
机械	99050003	混凝土振捣器(插入式)	台班	0.0830	0.0950	0.0690	0.1230	0.1350
	99460004	其他机具费 占人工费	%	1.50	1.50	1.50	1.50	1.50

北京市住房和城乡建设委员会

工作内容：混凝土浇筑、振捣、抹平、养护等。　　　　单位：m³

编号				4-15
项目				钢管柱混凝土
工料机名称			单位	消耗量
人工	00010501	综合用工二类	工日	0.501
材料	8021000810	预拌混凝土 C50	m^3	1.0100
	5135000101	泵管 ϕ150	kg	0.8100
	34000011	其他材料费 占材料费	%	1.00
机械	99460004	其他机具费 占人工费	%	1.50

北京市住房和城乡建设委员会

工作内容：混凝土浇筑、捣固、抹平、养护等。　　　　单位：m^3

编		号		4-16	4-17
项		目		现浇 0#块混凝土	箱梁混凝土
工料机名称			单位	消耗量	
人工	00010501	综合用工二类	工日	1.037	0.996
材料	8021000808	预拌混凝土 C40	m^3	1.0100	1.0100
	0129001109	普通钢板 δ0.5mm~0.65mm	kg	0.3994	0.6657
	34000011	其他材料费 占材料费	%	1.00	1.00
机械	99050003	混凝土振捣器(插入式)	台班	0.1490	0.1380
	99460004	其他机具费 占人工费	%	1.50	1.50

北京市住房和城乡建设委员会

工作内容：混凝土浇筑、捣固、抹平、养护等。　　　　单位：m^3

编号				4-18	4-19	4-20	4-21	4-22
项目				矩形梁板		连续实体板	异型连续板	支座垫石
				实心	空心			
工料机名称			单位	消耗量				
人工	00010501	综合用工二类	工日	0.665	0.760	0.756	0.774	0.952
材料	8021000808	预拌混凝土 C40	m^3	–	–	1.0100	1.0100	1.0100
	8021000809	预拌混凝土 C45	m^3	1.0100	1.0100	–	–	–
	34000011	其他材料费 占材料费	%	1.00	1.00	1.00	1.00	1.00
机械	99050003	混凝土振捣器(插入式)	台班	0.0800	0.0950	0.0980	0.1350	–
	99460004	其他机具费 占人工费	%	1.50	1.50	1.50	1.50	1.50

北京市住房和城乡建设委员会

工作内容：1.桥头搭板及枕梁：混凝土浇筑、捣固、抹平、养护等。2.桥头搭板橡胶板：橡胶板安装等。 **单位**：见表

编号				4-23	4-24
项目				桥头搭板及枕梁	桥头搭板橡胶板
				m^3	m^2
工料机名称			单位	消耗量	
人工	00010501	综合用工二类	工日	0.661	0.024
材料	8021000809	预拌混凝土 C45	m^3	1.0100	-
	0201000005	橡胶板 δ10mm	kg	-	15.3000
	34000011	其他材料费 占材料费	%	1.00	-
机械	99050003	混凝土振捣器(插入式)	台班	0.1150	-
	99460004	其他机具费 占人工费	%	1.50	1.50

北京市住房和城乡建设委员会

二、预制混凝土构件安装

工作内容：预制构件吊装、就位、固定等。　　　　**单位：**m^3

编号				4-25	4-26	4-27	4-28
项目				矩形梁板	混凝土桥梯	预应力空心板	预应力T型梁、I型梁
工料机名称			单位	消耗量			
人工	00010501	综合用工二类	工日	0.306	1.066	0.262	0.330
材料	0427002103	预制矩形梁板 C35	m^3	1.0000	–	–	–
	04290022	楼梯、休息板	m^3	–	1.0000	–	–
	03130101	电焊条（综合）	kg	–	0.5450	–	–
	0427002302	预制预应力空心板 C45	m^3	–	–	1.0000	–
	0427001103	预制预应力I型梁 C50	m^3	–	–	–	1.0000
	34000011	其他材料费 占材料费	%	1.00	1.00	1.00	1.00
机械	9925000202	交流电焊机 32kV·A	台班	–	0.2470	–	–
	9909000016	汽车式起重机 20t	台班	0.0400	0.0840	0.0400	–
	9925000017	电焊条烘干箱 60×50×75cm^3	台班	–	0.0890	–	–
	9909000028	汽车式起重机 120t	台班	–	–	–	0.0143
	99460004	其他机具费 占人工费	%	1.50	1.50	1.50	1.50

北京市住房和城乡建设委员会

工作内容：预制构件吊装、就位、固定等。 **单位**：m^3

编号				4-29	4-30
项目				预应力箱梁	
				吊车架设	架桥机架设
工料机名称			单位	消耗量	
人工	00010501	综合用工二类	工日	0.403	0.202
材料	0427002503	预制预应力箱梁 C50	m^3	1.0000	1.0000
	01010015	钢筋	kg	-	0.4810
	0129000209	热轧中厚钢板 δ10mm～16mm	kg	-	0.6820
	34000011	其他材料费 占材料费	%	1.00	1.00
机械	9909000008	履带式起重机 200t	台班	0.0220	-
	9909000030	履带式起重机 50t	台班	-	0.0211
	9909000803	架桥机 160t	台班	-	0.0877
	9943001101	立式油压千斤顶 300t	台班	-	0.0600
	9909000703	平台升降车 20m	台班	-	0.0705
	99460004	其他机具费 占人工费	%	1.50	1.50

北京市住房和城乡建设委员会

工作内容：混凝土浇筑、捣固、抹平、养护等。　　单位：m^3

编		号		4-31	4-32
项		目		板梁间接头	梁与梁接头
工　料　机　名　称			单位	消　耗　量	
人工	00010501	综合用工二类	工日	1.041	1.058
材料	8021000807	预拌混凝土 C35	m^3	1.0200	1.0200
	34000011	其他材料费 占材料费	%	1.00	1.00
机械	99050003	混凝土振捣器(插入式)	台班	0.1520	0.1530
	99460004	其他机具费 占人工费	%	1.50	1.50

北京市住房和城乡建设委员会

三、支座安装

工作内容：定位、支座吊装、固定等。　　　　**单位：**dm^3

编号				4-33	4-34
项目				板式橡胶支座	四氟板式橡胶支座
工料机名称			单位	消耗量	
人工	00010501	综合用工二类	工日	0.054	0.088
材料	36310012	橡胶支座	dm^3	1.0200	－
	02070023	四氟板支座	dm^3	－	1.0200
	0129001113	普通钢板 δ2mm～2.5mm	kg	－	0.0110
	03130101	电焊条（综合）	kg	－	0.1000
	34000011	其他材料费 占材料费	%	1.00	1.00
机械	9909000016	汽车式起重机 20t	台班	0.0050	0.0050
	9925000202	交流电焊机 32kV·A	台班	－	0.0077
	99460004	其他机具费 占人工费	%	1.50	1.50

北京市住房和城乡建设委员会

工作内容:支座定位、安装、固定、焊接等。　　　　单位:个

编号				4-35	4-36	4-37	4-38	4-39	4-40
项目				盆式金属橡胶组合支座(承载力)					
				≤3000KN	≤4000KN	≤5000KN	≤7000KN	≤10000KN	≤15000KN
工料机名称			单位	消耗量					
人工	00010501	综合用工二类	工日	1.100	1.271	1.425	2.211	2.937	3.603
材料	03130101	电焊条（综合）	kg	0.9000	1.0000	1.1000	1.3000	1.6000	1.9000
	36310015	钢盆式橡胶支座 ≤3000kN	个	1.0000	–	–	–	–	–
	36310016	钢盆式橡胶支座 ≤4000kN	个	–	1.0000	–	–	–	–
	36310017	钢盆式橡胶支座 ≤5000kN	个	–	–	1.0000	–	–	–
	36310020	钢盆式橡胶支座 ≤15000kN	个	–	–	–	–	–	1.0000
	36310018	钢盆式橡胶支座 ≤7000kN	个	–	–	–	1.0000	–	–
	36310019	钢盆式橡胶支座 ≤10000kN	个	–	–	–	–	1.0000	–
	34000011	其他材料费 占材料费	%	1.00	1.00	1.00	1.00	1.00	1.00
机械	9909000016	汽车式起重机 20t	台班	0.1400	0.1500	0.1600	0.1600	0.0300	0.0400
	9925000202	交流电焊机 32kV·A	台班	0.0692	0.0769	0.0846	0.1000	0.1231	0.1462
	9909000018	汽车式起重机 30t	台班	–	–	–	–	0.2600	0.2600
	99460004	其他机具费 占人工费	%	1.50	1.50	1.50	1.50	1.50	1.50

北京市住房和城乡建设委员会

四、压浆管道与压浆

工作内容:1.波纹管、铁皮管:压浆管定位、安装、固定、清孔等。2.橡胶管:管内塞筋或充气、安放就位、缠裹接头、抽拔、清洗胶管、清孔等。3.压水泥浆:水泥浆配制、拌合、运输、管道压浆等。

单位:见表

编号				4-41	4-42	4-43	4-44
项目				压浆管道			压水泥浆
				波纹管	铁皮管	橡胶管	
				m			m^3
工料机名称			单位	消耗量			
人工	00010501	综合用工二类	工日	0.037	0.037	0.019	3.298
材料	1721000003	波纹管 ϕ100	m	1.0780	–	–	–
	1711000551	铁皮管 ϕ100	m	–	1.0500	–	–
	17270003	橡胶管	m	–	–	1.0270	–
	80110001	素水泥浆	m^3	–	–	–	1.0500
	34000011	其他材料费 占材料费	%	1.00	1.00	1.00	1.00
机械	9905000008	灰浆搅拌机 200L	台班	–	–	–	0.5800
	99440009	注浆泵	台班	–	–	–	0.8200
	99460004	其他机具费 占人工费	%	1.50	1.50	1.50	1.50

北京市住房和城乡建设委员会

五、桥面栏杆与防撞墩

工作内容:1.安装混凝土挂板:构件制作、定位、吊运、安装、固定等。2.混凝土栏杆、扶手、混凝土防撞墩:混凝土配送、运输、浇筑、捣固、抹平、养护等。

单位:见表

编号				4-45	4-46	4-47
项目				安装混凝土挂板	混凝土栏杆、扶手	混凝土防撞墩
				m	m^3	
工料机名称			单位	消耗量		
人工	00010501	综合用工二类	工日	0.074	2.238	1.677
材料	04270031	预制混凝土挂板	m	1.0000	-	-
	8021000807	预拌混凝土 C35	m^3	-	1.0200	1.0200
	8001000010	水泥砂浆 M10	m^3	0.0010	-	-
	8001000008	水泥砂浆 M5	m^3	0.0030	-	-
	02310003	无纺土工布	m^2	-	0.7380	-
	34000011	其他材料费 占材料费	%	1.00	1.00	1.00
机械	9909000015	汽车式起重机 16t	台班	0.0200	-	-
	99460004	其他机具费 占人工费	%	1.50	1.50	1.50

北京市住房和城乡建设委员会

六、隔声屏障

工作内容：制作、场内运输、安装、就位等。 **单位：**见表

编号				4-48	4-49
项目				隔声屏障	
				钢骨架	隔声屏障板材
				t	m^2
工料机名称			单位	消耗量	
人工	00010501	综合用工二类	工日	8.508	0.183
材料	01490002	铝材	kg	62.1700	-
	09090003	卡普龙板	m^2	-	1.0500
	01630011	型钢耐候钢	t	1.0600	-
	03010103	抽芯铆钉	个	1097.2500	-
	03010555-1	螺栓	套	9.8000	-
	03130101	电焊条（综合）	kg	38.7500	-
	14290005-1	乙炔气	m^3	0.3980	-
	14290003	氧气	m^3	1.2000	-
	34000011	其他材料费 占材料费	%	1.00	1.00
机械	9907000005	载重汽车 8t	台班	1.7700	0.0059
	9909000016	汽车式起重机 20t	台班	0.4665	0.0020
	9925000202	交流电焊机 32kV·A	台班	2.1500	-
	99460004	其他机具费 占人工费	%	1.50	1.50

北京市住房和城乡建设委员会

七、伸缩缝

工作内容：定位、切割临时接头、焊接、安装等。　　**单位：**m

编		号		4-50	4-51	4-52
项		目		梳型钢板伸缩缝	钢板伸缩缝	橡胶板伸缩缝
工料机名称			单位	消	耗	量
人工	00010501	综合用工二类	工日	0.464	0.348	0.492
材料	33210003	梳型钢板伸缩缝	m	1.0000	–	–
	33210001	钢板伸缩缝	m	–	1.0000	–
	33210004	橡胶板伸缩缝	m	–	–	1.0000
	01010002-1	钢筋 ϕ10 以内	kg	0.7000	0.6000	0.2000
	03130101	电焊条（综合）	kg	2.6580	2.5340	1.0380
	14210001	环氧树脂	kg	–	–	0.0500
	34000011	其他材料费 占材料费	%	1.00	1.00	1.00
机械	9909000013	汽车式起重机 10t	台班	0.0060	0.0060	0.0060
	9925000202	交流电焊机 32kV·A	台班	0.3470	0.3080	0.1310
	9925000017	电焊条烘干箱 60×50×75cm^3	台班	0.0890	0.0890	0.0890
	99250003	电焊条恒温箱	台班	0.0890	0.0890	0.0890
	99460004	其他机具费 占人工费	%	1.50	1.50	1.50

北京市住房和城乡建设委员会

第二节 明挖结构工程

一、竖井混凝土

工作内容：1.喷射混凝土：施工准备、配料、材料场内水平运输、喷射、养护、清理等。2.二衬混凝土：施工准备、材料场内水平运输、混凝土浇筑、振捣、清理、养护、人工配合等。

单位：m^3

编号				4-53	4-54
项目				喷射混凝土	二衬混凝土
工料机名称			单位	消耗量	
人工	00010501	综合用工二类	工日	2.141	0.459
材料	8021001001	预拌喷射混凝土干料 C20	m^3	1.2500	-
	8021000807	预拌混凝土 C35	m^3	-	1.0200
	22450010	高压胶皮风管	m	0.2620	-
	34000011	其他材料费 占材料费	%	1.00	1.00
机械	9905000401	混凝土喷射机 $5m^3/h$	台班	0.1200	-
	9943000007	电动空气压缩机 $10m^3/min$	台班	0.1200	-
	99050003	混凝土振捣器(插入式)	台班	-	0.0810
	99460004	其他机具费 占人工费	%	1.50	1.50

北京市住房和城乡建设委员会

二、明挖结构混凝土

工作内容：施工准备、材料场内水平运输、浇筑、振捣、养护等。 **单位：**m^3

编号				4-55	4-56	4-57	4-58	4-59
项目				垫层	底板	中层(顶)板	站台板	侧墙(内衬墙)
工料机名称			单位	消耗量				
人工	00010501	综合用工二类	工日	0.212	0.275	0.290	0.286	0.369
材料	8021000803	预拌混凝土 C20	m^3	1.0100	–	–	–	–
	8021000905	预拌抗渗混凝土 C35	m^3	–	1.0100	1.0100	–	1.0100
	8021000807	预拌混凝土 C35	m^3	–	–	–	1.0100	–
	34000011	其他材料费 占材料费	%	1.00	1.00	1.00	1.00	1.00
机械	99050004	混凝土振捣器(平板式)	台班	0.1610	–	–	–	–
	99050003	混凝土振捣器(插入式)	台班	–	0.0550	0.0620	0.0600	0.0780
	99460004	其他机具费 占人工费	%	1.50	1.50	1.50	1.50	1.50

北京市住房和城乡建设委员会

工作内容：施工准备、材料场内水平运输、浇筑、振捣、养护等。 **单位：**m^3

编号				4-60	4-61	4-62	4-63	4-64	4-65
项目				中隔墙	站台板下墙	地梁	梁	柱	楼梯
工料机名称			单位	消耗量					
人工	00010501	综合用工二类	工日	0.372	0.369	0.264	0.273	0.517	1.176
材料	8021000807	预拌混凝土 C35	m^3	1.0100	1.0100	–	–	1.0100	1.0100
	8021000905	预拌抗渗混凝土 C35	m^3	–	–	1.0100	1.0100	–	–
	34000011	其他材料费 占材料费	%	1.00	1.00	1.00	1.00	1.00	1.00
机械	99050003	混凝土振捣器(插入式)	台班	0.0780	0.0780	0.0540	0.0550	0.1000	0.1700
	99460004	其他机具费 占人工费	%	1.50	1.50	1.50	1.50	1.50	1.50

北京市住房和城乡建设委员会

工作内容：施工准备、材料场内水平运输、浇筑、振捣、养护等。　　　　**单位：**m^3

编号				4-66	4-67	4-68	4-69
项目				电缆沟	风道	轨顶风道	电缆夹层
工料机名称			单位	消耗量			
人工	00010501	综合用工二类	工日	0.950	1.323	2.398	0.959
材料	8021000807	预拌混凝土 C35	m^3	1.0100	1.0100	1.0100	1.0100
材料	34000011	其他材料费 占材料费	%	1.00	1.00	1.00	1.00
机械	99050003	混凝土振捣器(插入式)	台班	0.1500	0.1810	0.3010	0.1510
机械	99460004	其他机具费 占人工费	%	1.50	1.50	1.50	1.50

北京市住房和城乡建设委员会

第三节 暗挖与盖挖结构工程

一、喷射混凝土

工作内容：施工准备、配料、垂直及洞内运输、喷射、养护、清理等。 **单位：**m^3

编号				4-70	4-71	4-72	4-73	4-74
项目				喷射混凝土		喷射钢纤维混凝土		喷射混凝土
				弧形隧道	矩形隧道	弧形隧道	矩形隧道	临时支护
工料机名称			单位	消耗量				
人工	00010504	综合用工二类	工日	2.619	2.663	3.113	3.170	2.471
材料	8021000204	预拌喷射混凝土干料 C25	m^3	1.3000	1.3200	1.1460	1.1960	1.2300
	05010001	原木	m^3	–	–	0.0010	0.0010	–
	05030007	板方材	m^3	–	–	0.0010	0.0010	–
	1727000108	高压胶管 ϕ50	m	–	–	0.3510	0.3650	–
	14350065	液体速凝剂	kg	–	–	24.4310	25.4080	–
	15550001	钢纤维	kg	–	–	84.7040	88.0920	–
	22450010	高压胶皮风管	m	0.2500	0.2500	–	–	0.2500
	34000011	其他材料费 占材料费	%	1.00	1.00	1.00	1.00	1.00
机械	9905000401	混凝土喷射机 5m^3/h	台班	0.1300	0.1350	0.1320	0.1370	0.1230
	9943000007	电动空气压缩机 10m^3/min	台班	0.1300	0.1350	0.1320	0.1370	0.1230
	99310001	电动三轮车	台班	0.1500	0.1560	0.1520	0.1580	0.1400
	9909000403	桥式起重机 10t	台班	0.0060	0.0062	0.0053	0.0055	0.0057
	99460004	其他机具费 占人工费	%	1.50	1.50	1.50	1.50	1.50

北京市住房和城乡建设委员会

二、暗挖与盖挖车站混凝土结构

工作内容：施工准备、浇筑、振捣、养护、垂直及洞内运输等。　　　　**单位：**m^3

编号				4-75	4-76	4-77	4-78	4-79	4-80
项目				垫层	底板	中层板	暗挖顶板	站台板	轨顶风道
工料机名称			单位	消耗量					
人工	00010504	综合用工二类	工日	0.522	0.557	0.629	0.692	0.539	2.488
材料	8021000807	预拌混凝土 C35	m^3	1.0400	–	–	–	1.0400	1.0400
	8021000905	预拌抗渗混凝土 C35	m^3	–	1.0400	1.0400	1.0400	–	–
	5135000101	泵管 ϕ150	kg	0.8100	0.8100	0.8100	0.8100	0.8100	0.8100
	34000011	其他材料费 占材料费	%	1.00	1.00	1.00	1.00	1.00	1.00
机械	99050004	混凝土振捣器(平板式)	台班	0.1800	–	–	–	–	–
	99050003	混凝土振捣器(插入式)	台班	–	0.1250	0.1370	0.1410	0.1230	0.3060
	99460004	其他机具费 占人工费	%	1.50	1.50	1.50	1.50	1.50	1.50

北京市住房和城乡建设委员会

工作内容：施工准备、浇筑、振捣、养护、垂直及洞内运输等。　　　　单位：m^3

编号				4-81	4-82	4-83	4-84	4-85
项目				电缆夹层	内衬墙	中隔墙	矩形柱	圆形柱
工料机名称			单位	消耗量				
人工	00010504	综合用工二类	工日	0.579	0.649	0.687	0.723	0.726
材料	8021000905	预拌抗渗混凝土 C35	m^3	1.0400	1.0400	1.0400	1.0400	1.0400
	5135000101	泵管 ϕ150	kg	0.8100	0.8100	0.8100	0.8100	0.8100
	34000011	其他材料费 占材料费	%	1.00	1.00	1.00	1.00	1.00
机械	99050003	混凝土振捣器(插入式)	台班	0.1250	0.1380	0.1400	0.1410	0.1430
	99460004	其他机具费 占人工费	%	1.50	1.50	1.50	1.50	1.50

北京市住房和城乡建设委员会

工作内容：施工准备、浇筑、振捣、养护、垂直及洞内运输等。　　　　单位：m^3

编号				4-86	4-87	4-88	4-89
项目				底梁	天梁	中板梁	填充混凝土
工料机名称			单位	消耗量			
人工	00010504	综合用工二类	工日	0.511	0.866	0.588	0.495
材料	8021000905	预拌抗渗混凝土 C35	m^3	1.0400	1.0400	1.0400	1.0400
	5135000101	泵管 ϕ150	kg	0.8100	0.8100	0.8100	0.8100
	34000011	其他材料费 占材料费	%	1.00	1.00	1.00	1.00
机械	99050003	混凝土振捣器(插入式)	台班	0.1220	0.1530	0.1330	0.1100
	99460004	其他机具费 占人工费	%	1.50	1.50	1.50	1.50

北京市住房和城乡建设委员会

三、暗挖区间混凝土

工作内容：施工准备、浇筑、振捣、养护、垂直及洞内运输等。 **单位：**m^3

编号				4-90	4-91	4-92	4-93	4-94	4-95
项目				弧形隧道			矩形隧道		
				底板	边墙	顶板	底板	直墙	顶板
工料机名称			单位	消耗量					
人工	00010504	综合用工二类	工日	0.557	0.649	0.692	0.585	0.681	0.727
材料	8021000905	预拌抗渗混凝土 C35	m^3	1.0400	1.0400	1.0400	1.0400	1.0400	1.0400
	5135000101	泵管 ϕ150	kg	0.8700	0.8700	0.8700	0.8700	0.8700	0.8700
	34000011	其他材料费 占材料费	%	1.00	1.00	1.00	1.00	1.00	1.00
机械	99050003	混凝土振捣器(插入式)	台班	0.1250	0.1380	0.1410	0.1320	0.1400	0.1430
	99460004	其他机具费 占人工费	%	1.50	1.50	1.50	1.50	1.50	1.50

北京市住房和城乡建设委员会

四、暗挖与盖挖车站钢管柱混凝土

工作内容：导管下放、混凝土浇捣、拔管、测量深度等。　　　　**单位：**m^3

编号				4-96	4-97	4-98	4-99
项目				盖挖		暗挖	
				钢管柱混凝土	钢管柱混凝土封口	钢管柱混凝土	钢管柱混凝土封口
工料机名称			单位	消耗量			
人工	00010504	综合用工二类	工日	0.557	0.648	0.668	0.778
材料	8021000810	预拌混凝土 C50	m^3	1.0400	–	1.0400	–
	8021000807	预拌混凝土 C35	m^3	–	1.0400	–	1.0400
	5135000101	泵管 ϕ150	kg	0.8100	0.8100	0.8100	0.8100
	34000011	其他材料费 占材料费	%	1.00	1.00	1.00	1.00
机械	99050003	混凝土振捣器(插入式)	台班	–	0.1380	–	0.1470
	99460004	其他机具费 占人工费	%	1.50	1.50	1.50	1.50

北京市住房和城乡建设委员会

第四节 钢筋

一、钢筋制作安装

工作内容：1.制作：钢筋的解捆、除锈、调直、切割、制作等。2.安装：钢筋场内水平运输、安装。 **单位：**t

编号				4-100	4-101	4-102	4-103	4-104	4-105
项目				钢筋制作		钢筋安装			
						高架		明挖	
				ϕ10 以内	ϕ10 以外	ϕ10 以内	ϕ10 以外	ϕ10 以内	ϕ10 以外
工料机名称			单位	消耗量					
人工	00010301	综合用工一类	工日	1.964	1.079	4.903	3.073	4.911	3.084
材料	01010002-1	钢筋 ϕ10 以内	kg	1050.0000	–	–	–	–	–
	01010003	钢筋 ϕ10 以外	kg	–	1050.0000	–	–	–	–
	03150710	镀锌铁丝 $8^{\#}$～$12^{\#}$	kg	–	–	5.5000	1.5000	5.5000	1.5000
	03130101	电焊条（综合）	kg	–	–	–	9.6500	–	9.6500
	34000011	其他材料费 占材料费	%	1.00	1.00	1.00	1.00	1.00	1.00
机械	9909000015	汽车式起重机 16t	台班	–	–	0.0800	0.0800	–	–
	99170005	钢筋调直机	台班	0.2400	–	–	–	–	–
	99170009	钢筋弯曲机 ϕ40 内	台班	0.5800	0.4800	–	–	–	–
	99170007	钢筋切断机	台班	0.2400	0.1100	–	–	–	–
	9925000202	交流电焊机 32kV·A	台班	–	–	–	0.8040	–	0.8040
	9909000301	龙门式起重机 10t	台班	–	–	–	–	0.0080	0.0070
	9907000121	平板拖车 10t	台班	–	–	0.0300	0.0300	0.0300	0.0300
	99460004	其他机具费 占人工费	%	1.50	1.50	1.50	1.50	1.50	1.50

北京市住房和城乡建设委员会

工作内容：1.钢筋安装(竖井)：钢筋安装等。2.钢筋安装(暗挖)：钢筋垂直及洞内运输、安装等。　　**单位：**t

编号				4-106	4-107	4-108	4-109
项目				钢筋安装			
				竖井		暗挖	
				ϕ10 以内	ϕ10 以外	ϕ10 以内	ϕ10 以外
工料机名称			单位	消耗量			
人工	00010301	综合用工一类	工日	6.340	2.970	–	–
	00010304	综合用工一类	工日	–	–	6.500	3.583
材料	03130101	电焊条（综合）	kg	–	9.6500	–	9.6500
	03150710	镀锌铁丝 8#～12#	kg	5.5000	1.5000	5.5000	1.5000
	34000011	其他材料费 占材料费	%	1.00	1.00	1.00	1.00
机械	99310001	电动三轮车	台班	–	–	0.1840	0.1050
	9909000403	桥式起重机 10t	台班	–	–	0.0568	0.0410
	9925000202	交流电焊机 32kV·A	台班	–	0.8040	–	0.8040
	9909000016	汽车式起重机 20t	台班	0.0050	0.0040	–	–
	9907000121	平板拖车 10t	台班	0.0300	0.0300	0.0300	0.0300
	99460004	其他机具费 占人工费	%	1.50	1.50	1.50	1.50

北京市住房和城乡建设委员会

工作内容：1.钢筋网片(竖井)、连接筋(竖井)和钢筋格栅(竖井)：制作、安装等。2.钢筋网片(暗挖)、连接筋(暗挖)和钢筋格栅(暗挖、盖挖)：垂直及洞内运输、制作、安装等。

单位：t

编号				4-110	4-111	4-112	4-113	4-114	4-115
项目				钢筋网片		连接筋		钢筋格栅	
				竖井	暗挖	竖井	暗挖	竖井	暗挖、盖挖
工料机名称			单位	消耗量					
人工	00010301	综合用工一类	工日	7.599	–	5.346	–	11.359	–
	00010304	综合用工一类	工日	–	9.783	–	6.163	–	13.041
材料	01010002-1	钢筋 ϕ10 以内	kg	1020.0000	1020.0000	–	–	134.0000	134.0000
	01010003	钢筋 ϕ10 以外	kg	–	–	1050.0000	1050.0000	756.0000	756.0000
	03130101	电焊条（综合）	kg	4.3300	4.4300	13.0910	13.0910	15.0000	15.2900
	03150710	镀锌铁丝 $8^{\#}\sim12^{\#}$	kg	5.2400	–	–	–	–	–
	01000001-1	型钢 综合	kg	–	–	–	–	153.0000	153.0000
	0301050216	六角螺栓 M16×250	个	–	–	–	–	30.0000	36.0000
	34000011	其他材料费 占材料费	%	1.00	1.00	1.00	1.00	1.00	1.00
机械	99170005	钢筋调直机	台班	0.2000	0.2000	–	–	0.0420	0.1420
	99170007	钢筋切断机	台班	0.1000	0.1000	0.1000	0.1000	0.1980	0.2480
	9925000202	交流电焊机 32kV·A	台班	0.3600	0.3600	1.0900	1.0900	1.2500	1.2740
	99310001	电动三轮车	台班	–	0.2840	–	0.2560	–	0.2340
	9909000403	桥式起重机 10t	台班	–	0.0413	–	0.0372	–	0.0340
	9909000016	汽车式起重机 20t	台班	–	–	0.0050	–	0.0036	–
	99170009	钢筋弯曲机 ϕ40 内	台班	–	–	–	–	0.3190	0.5190
	9907000121	平板拖车 10t	台班	0.0300	0.0300	0.0300	0.0300	0.0300	0.0300
	9909000013	汽车式起重机 10t	台班	0.0066	–	–	–	–	–
	99460004	其他机具费 占人工费	%	1.50	1.50	1.50	1.50	1.50	1.50

北京市住房和城乡建设委员会

工作内容:1.型钢格栅(竖井)、钢梯(明挖):制作、安装等。2.型钢格栅(暗挖、盖挖)、钢梯(暗挖、盖挖):垂直及洞内运输、制作、安装等。

单位:t

编号				4-116	4-117	4-118	4-119
项目				型钢格栅		钢梯	
				竖井	暗挖、盖挖	明挖	暗挖、盖挖
工料机名称			单位	消耗量			
人工	00010301	综合用工一类	工日	7.047	–	14.653	–
	00010304	综合用工一类	工日	–	8.088	–	15.151
材料	01010003	钢筋 ϕ10 以外	kg	23.0000	23.0000	231.0000	231.0000
	03130101	电焊条(综合)	kg	9.0000	9.0000	26.5000	26.5000
	01000001-1	型钢 综合	kg	1014.0000	1014.0000	–	–
	0301050216	六角螺栓 M16×250	个	32.0000	38.0000	–	–
	05030007	板方材	m^3	–	–	0.0200	0.0200
	01210010	角钢 63 以外	kg	–	–	74.0000	74.0000
	01190009	槽钢 16 以内	kg	–	–	85.0000	85.0000
	0129000808	钢板 δ4.5mm~20mm	kg	–	–	670.0000	670.0000
	03010556	螺栓(带帽)(综合)	kg	–	–	1.7400	1.7400
	14290005-1	乙炔气	m^3	–	–	–	2.0500
	14290003	氧气	m^3	–	–	–	6.1600
	34000011	其他材料费 占材料费	%	1.00	1.00	1.00	1.00
机械	99170007	钢筋切断机	台班	0.1500	–	–	–
	9925000202	交流电焊机 32kV·A	台班	0.7500	0.7500	2.2000	2.2000
	99310001	电动三轮车	台班	–	0.2240	–	0.1800
	9909000403	桥式起重机 10t	台班	–	0.0325	–	0.0560
	9909000016	汽车式起重机 20t	台班	0.0036	–	–	–
	99190019	型钢校正机	台班	0.2000	0.2000	0.1100	0.1100
	9923000024	型钢剪断机 500mm	台班	0.1000	0.1000	0.1100	0.1100
	9907000121	平板拖车 10t	台班	0.0300	0.0300	0.0300	0.0300
	99460004	其他机具费 占人工费	%	1.50	1.50	1.50	1.50

北京市住房和城乡建设委员会

工作内容：1.预埋件(高架、明挖)：制作、安装等。2.预埋件(暗挖、盖挖)：垂直及洞内运输、制作、安装等。3.玻璃纤维筋：制作、安装等。

单位：t

编号				4-120	4-121	4-122	4-123
项目				预埋件			玻璃纤维筋
				高架	明挖	暗挖、盖挖	
工料机名称			单位	消耗量			
人工	00010301	综合用工一类	工日	11.910	11.870	–	11.816
	00010304	综合用工一类	工日	–	–	14.244	–
材料	14290005-1	乙炔气	m^3	3.5300	3.5300	3.5300	–
	14290003	氧气	m^3	10.6000	10.6000	10.6000	–
	03130101	电焊条（综合）	kg	30.6600	30.6600	32.8000	–
	02350001	玻璃纤维筋	kg	–	–	–	1050.0000
	1825002104	U 型卡子 108	个	–	–	–	205.0000
	03150710	镀锌铁丝 8#～12#	kg	–	–	–	5.8400
	0129000808	钢板 δ4.5mm～20mm	kg	1050.0000	1050.0000	1050.0000	–
	34000011	其他材料费 占材料费	%	1.00	1.00	1.00	0.10
机械	99190015	切割机	台班	0.0600	0.0600	0.0600	–
	9925000005	直流弧焊机 32kV·A	台班	2.5550	2.5550	2.7300	–
	99310001	电动三轮车	台班	–	–	0.1800	–
	9909000403	桥式起重机 10t	台班	–	–	0.0560	–
	9909000016	汽车式起重机 20t	台班	0.1400	–	–	0.2400
	9907000121	平板拖车 10t	台班	–	–	0.0300	–
	99460004	其他机具费 占人工费	%	1.50	1.50	1.50	0.10

北京市住房和城乡建设委员会

二、桥梁预应力钢筋

工作内容：调直、下料、安拆夹具、张拉锚固、切断等。　　　　**单位：**t

编号				4-124	4-125
项目				预应力先张法	
				钢绞线	钢筋
工料机名称			单位	消耗量	
人工	00010301	综合用工一类	工日	5.694	4.398
材料	01010016	预应力钢筋	kg	–	1025.0000
	01070001-1	钢绞线	kg	1025.0000	–
	03150906	铁件	kg	4.3700	1.3400
	0129001117	普通钢板 δ8mm～15mm	kg	13.0000	4.0000
	14290003	氧气	m^3	1.0300	0.6300
	14290005-1	乙炔气	m^3	0.3400	0.2100
	03130101	电焊条（综合）	kg	–	8.0000
	34000011	其他材料费 占材料费	%	1.00	1.00
机械	99170007	钢筋切断机	台班	–	0.1000
	9917000104	预应力拉伸机 YCW-400	台班	0.5000	0.3200
	9925000008	对焊机 75kV·A	台班	–	0.6670
	9944000009	高压油泵 80MPa	台班	0.5000	0.3200
	9909000016	汽车式起重机 20t	台班	0.1200	0.1200
	99460004	其他机具费 占人工费	%	1.50	1.50

北京市住房和城乡建设委员会

工作内容：钢绞线制作安装、锚具安装、张拉、锚固等。　　　　**单位：**t

编号				4-126	4-127	4-128	4-129	4-130	4-131
项目				预应力后张法钢绞线					
				束长≤20m			束长≤40m		
				3孔以内	7孔以内	12孔以内	7孔以内	12孔以内	19孔以内
工料机名称			单位	消耗量					
人工	00010301	综合用工一类	工日	16.648	8.890	6.601	5.818	4.797	4.606
材料	01070001-1	钢绞线	kg	1025.0000	1025.0000	1025.0000	1025.0000	1025.0000	1025.0000
	03150710	镀锌铁丝 8#~12#	kg	0.7500	0.3200	0.1800	0.3200	0.1800	0.1200
	14290003	氧气	m^3	0.6300	0.6300	0.6300	0.2900	0.2900	0.2900
	14290005-1	乙炔气	m^3	0.2100	0.2100	0.2100	0.1000	0.1000	0.1000
	34000011	其他材料费 占材料费	%	1.00	1.00	1.00	1.00	1.00	1.00
机械	9944000009	高压油泵 80MPa	台班	3.9200	2.3520	1.5288	1.6464	1.0396	0.6956
	9917000101	预应力拉伸机 YCW-100	台班	3.9200	-	-	-	-	-
	9917000102	预应力拉伸机 YCW-150	台班	-	2.3520	-	1.6464	-	-
	9917000103	预应力拉伸机 YCW-250	台班	-	-	1.5288	-	1.0396	-
	9917000104	预应力拉伸机 YCW-400	台班	-	-	-	-	-	0.6956
	9909000016	汽车式起重机 20t	台班	0.1200	0.1200	0.1200	0.1200	0.1200	0.1200
	99460004	其他机具费 占人工费	%	1.50	1.50	1.50	1.50	1.50	1.50

北京市住房和城乡建设委员会

工作内容：调直、切断、编束、穿束、安装锚具、张拉、锚固、拆除、切割钢丝(束)等。　　**单位：**t

编号				4-132	4-133
项目				预应力后张法钢筋	
				JM12 型锚	螺栓锚
工料机名称			单位	消耗量	
人工	00010301	综合用工一类	工日	10.114	9.639
材料	01010016	预应力钢筋	kg	1025.0000	1025.0000
	03150710	镀锌铁丝 8#～12#	kg	0.6800	-
	14290003	氧气	m^3	0.2000	1.1100
	14290005-1	乙炔气	m^3	0.0700	0.3700
	34000011	其他材料费 占材料费	%	1.00	1.00
机械	9917000201	预应力液压拉伸机 90t	台班	1.1500	1.3900
	99440007	高压油泵	台班	1.1500	1.3900
	99190015	切割机	台班	0.8100	-
	9909000016	汽车式起重机 20t	台班	0.1200	0.1200
	99460004	其他机具费 占人工费	%	1.50	1.50

北京市住房和城乡建设委员会

三、直螺纹套筒接头

工作内容：除锈、调直、校正、配料、钢筋端头加工、机具准备、材料运输、连接等。 **单位：**个

编号				4-134	4-135	4-136	4-137	4-138	4-139
项目				明挖套筒接头					
				$\phi\leq20$	$\phi\leq22$	$\phi\leq25$	$\phi\leq28$	$\phi\leq32$	$\phi>32$
工料机名称			单位	消耗量					
人工	00010301	综合用工一类	工日	0.019	0.022	0.025	0.028	0.031	0.034
材料	0315110501	钢筋直螺纹套筒 18	个	1.0100	–	–	–	–	–
	0315110502	钢筋直螺纹套筒 22	个	–	1.0100	–	–	–	–
	0315110504	钢筋直螺纹套筒 28	个	–	–	1.0100	1.0100	–	–
	0315110505	钢筋直螺纹套筒 32	个	–	–	–	–	1.0100	1.0100
	29060603	塑护套	个	2.0400	2.0400	2.0400	2.0400	2.0400	2.0400
	34000011	其他材料费 占材料费	%	1.00	1.00	1.00	1.00	1.00	1.00
机械	9919000012	螺栓套丝机 39mm	台班	0.0150	0.0180	0.0210	0.0240	0.0270	0.0300
	99460004	其他机具费 占人工费	%	1.50	1.50	1.50	1.50	1.50	1.50

北京市住房和城乡建设委员会

工作内容：除锈、调直、校正、配料、钢筋端头加工、机具准备、材料运输、连接等。　　　　单位：个

编号				4-140	4-141	4-142	4-143	4-144	4-145
项目				暗挖套筒接头					
				$\phi\leqslant20$	$\phi\leqslant22$	$\phi\leqslant25$	$\phi\leqslant28$	$\phi\leqslant32$	$\phi>32$
工料机名称			单位	消耗量					
人工	00010304	综合用工一类	工日	0.023	0.026	0.030	0.034	0.037	0.041
材料	0315110501	钢筋直螺纹套筒 18	个	1.0100	–	–	–	–	–
	0315110502	钢筋直螺纹套筒 22	个	–	1.0100	–	–	–	–
	0315110504	钢筋直螺纹套筒 28	个	–	–	1.0100	1.0100	–	–
	0315110505	钢筋直螺纹套筒 32	个	–	–	–	–	1.0100	1.0100
	29060603	塑护套	个	2.0400	2.0400	2.0400	2.0400	2.0400	2.0400
	34000011	其他材料费 占材料费	%	1.00	1.00	1.00	1.00	1.00	1.00
机械	9919000012	螺栓套丝机 39mm	台班	0.0150	0.0180	0.0210	0.0240	0.0270	0.0300
	9909000403	桥式起重机 10t	台班	0.0002	0.0002	0.0002	0.0002	0.0002	0.0002
	99310001	电动三轮车	台班	0.0010	0.0010	0.0010	0.0010	0.0010	0.0010
	99460004	其他机具费 占人工费	%	1.50	1.50	1.50	1.50	1.50	1.50

北京市住房和城乡建设委员会

四、植筋

工作内容:定位、钻孔、清孔、钢筋加工成型、注胶、植筋、养护等。 **单位:**根

编号				4-146	4-147	4-148	4-149
项目				植筋			
				锚固深度 15d			
				ϕ10	ϕ12	ϕ14	ϕ16
工料机名称			单位	消耗量			
人工	00010501	综合用工二类	工日	0.077	0.097	0.119	0.142
材料	01010002-1	钢筋 ϕ10 以内	kg	0.2520	-	-	-
	01010003	钢筋 ϕ10 以外	kg	-	0.3990	0.6440	0.7510
	14410082	植筋胶粘剂	ml	15.8560	24.8510	36.6940	62.6450
	03151305	合金钢钻头	个	0.0158	0.0199	0.0243	0.0292
	34000011	其他材料费 占材料费	%	1.00	1.00	1.00	1.00
机械	99030029	手动钻孔机	台班	0.0095	0.0119	0.0146	0.0175
	99460004	其他机具费 占人工费	%	1.50	1.50	1.50	1.50

北京市住房和城乡建设委员会

工作内容：定位、钻孔、清孔、钢筋加工成型、注胶、植筋、养护等。　　　　**单位：**根

编号				4-150	4-151	4-152	4-153
项目				植筋			
				锚固深度 15d			
				ϕ18	ϕ20	ϕ22	ϕ25
工料机名称			单位	消耗量			
人工	00010501	综合用工二类	工日	0.179	0.207	0.239	0.282
材料	01010003	钢筋 ϕ10 以外	kg	0.8770	1.2110	1.6220	2.3960
	14410082	植筋胶粘剂	ml	91.0070	126.8440	160.1730	207.0920
	03151305	合金钢钻头	个	0.0345	0.0384	0.0444	0.0530
	34000011	其他材料费 占材料费	%	1.00	1.00	1.00	1.00
机械	99030029	手动钻孔机	台班	0.0207	0.0246	0.0284	0.0339
	99460004	其他机具费 占人工费	%	1.50	1.50	1.50	1.50

北京市住房和城乡建设委员会

工作内容：定位、钻孔、清孔、钢筋加工成型、注胶、植筋、养护等。　　**单位：**根

编号				4-154	4-155	4-156	4-157
项目				植筋			
				每增减 1cm（锚固深度）			
				$\phi10$	$\phi12$	$\phi14$	$\phi16$
工料机名称			单位	消耗量			
人工	00010501	综合用工二类	工日	0.005	0.005	0.006	0.006
材料	01010002-1	钢筋 $\phi10$ 以内	kg	0.0070	–	–	–
	01010003	钢筋 $\phi10$ 以外	kg	–	0.0090	0.0120	0.0170
	14410082	植筋胶粘剂	ml	1.0570	1.3810	1.7470	2.6100
	03151305	合金钢钻头	个	0.0004	0.0004	0.0005	0.0007
	34000011	其他材料费 占材料费	%	1.00	1.00	1.00	1.00
机械	99030029	手动钻孔机	台班	0.0003	0.0003	0.0003	0.0004
	99460004	其他机具费 占人工费	%	1.50	1.50	1.50	1.50

北京市住房和城乡建设委员会

工作内容：定位、钻孔、清孔、钢筋加工成型、注胶、植筋、养护等。 单位：根

编号				4-158	4-159	4-160	4-161
项目				植筋			
				每增减 1cm(锚固深度)			
				ϕ18	ϕ20	ϕ22	ϕ25
工料机名称			单位	消耗量			
人工	00010501	综合用工二类	工日	0.006	0.007	0.007	0.007
材料	01010003	钢筋 ϕ10 以外	kg	0.0210	0.0260	0.0310	0.0370
	14410082	植筋胶粘剂	ml	3.3710	4.2280	4.8540	5.5220
	03151305	合金钢钻头	个	0.0008	0.0008	0.0008	0.0008
	34000011	其他材料费 占材料费	%	1.00	1.00	1.00	1.00
机械	99030029	手动钻孔机	台班	0.0005	0.0005	0.0005	0.0005
	99460004	其他机具费 占人工费	%	1.50	1.50	1.50	1.50

北京市住房和城乡建设委员会

第五节 疏散平台

一、混凝土定型化学锚栓安装

工作内容:画线、钻孔、清孔、注胶、安装锚栓等。 **单位:**套

编号				4-162	4-163	4-164	4-165
项目				混凝土定型化学锚栓安装			
				M10	M12	M16	M24
工料机名称			单位	消耗量			
人工	00010501	综合用工二类	工日	0.174	0.176	0.177	0.178
材料	0301080901	混凝土定型化学锚栓 M10	套	1.0200	–	–	–
	0315130003-2	冲击钻头 ϕ12	根	0.0192	–	–	–
	0315130404	水钻钻头 12	根	0.0425	–	–	–
	35110010	胶枪	把	0.0125	0.0125	0.0200	0.0200
	35110014	毛刷	把	0.0200	0.0200	0.0200	0.0200
	0301080902	混凝土定型化学锚栓 M12	套	–	1.0200	–	–
	0315130004-2	冲击钻头 ϕ14	根	–	0.0192	–	–
	0315130405	水钻钻头 14	根	–	0.0425	–	–
	0301080903	混凝土定型化学锚栓 M16	套	–	–	1.0200	–
	0315130006	冲击钻头 ϕ18	根	–	–	0.0192	–
	0315130406	水钻钻头 18	根	–	–	0.0425	–
	0301080904	混凝土定型化学锚栓 M24	套	–	–	–	1.0200
	0315130010	冲击钻头 ϕ26	根	–	–	–	0.0192
	0315130407	水钻钻头 26	根	–	–	–	0.0425
	34000011	其他材料费 占材料费	%	1.00	1.00	1.00	1.00
机械	99410001	水钻	台班	0.0150	0.0151	0.0153	0.0155
	9945000039	电锤 520W	台班	0.0068	0.0069	0.0070	0.0072
	99460004	其他机具费 占人工费	%	1.50	1.50	1.50	1.50

北京市住房和城乡建设委员会

二、疏散平台安装

工作内容：施工准备、定位、运输、安装、调平。　　　　**单位：**见表

编号				4-166	4-167	4-168	4-169
项目				疏散平台支架安装		疏散平台面板安装	
				水泥基复合材料	钢结构	水泥基复合材料	钢质材料
				套	t	m^2	
工料机名称			单位	消耗量			
人工	00010501	综合用工二类	工日	0.417	–	0.487	0.459
	00010301	综合用工一类	工日	–	6.719	–	–
材料	04270018	水泥基支架复合材料	套	(1.0200)	–	–	–
	20330002	调节垫片	kg	2.0000	143.0000	–	–
	33010053	零星钢构件	t	–	0.4561	–	–
	01210011	镀锌角钢	kg	–	108.1510	–	–
	0129000613	镀锌钢板 δ1.0mm~1.5mm	kg	–	475.8490	–	–
	03130101	电焊条（综合）	kg	–	11.0000	–	–
	80050009	水泥基复合材料	m^2	–	–	1.0400	–
	0301070010-1	膨胀螺栓 M16	套	–	–	4.0800	4.0800
	04011101	水泥(综合)	kg	–	–	1.5000	–
	02010014	硅胶板	m^2	–	–	–	0.0240
	33010038	钢质疏散平台板	m^2	–	–	–	1.0400
	34000011	其他材料费 占材料费	%	1.00	1.00	1.00	1.00
机械	99070005	轨道平车	台班	0.0334	0.2940	0.0700	0.0630
	9909000015	汽车式起重机 16t	台班	0.0167	0.2940	0.0700	0.0600
	9943000104	汽油发电机 10kW	台班	0.1187	0.3550	0.0220	0.0218
	9925000201	交流电焊机 21kV·A	台班	–	0.9170	–	–
	9907000202	轨道车 ≤210kW	台班	0.0334	0.2940	0.0700	0.0630
	9907000005	载重汽车 8t	台班	0.0271	0.3230	0.0200	0.0198
	99460004	其他机具费 占人工费	%	1.50	1.50	1.50	1.50

北京市住房和城乡建设委员会

三、疏散平台扶手安装

工作内容：放样、下料、安装等。　　　　**单位：**m

编		号		4-170
项		目		疏散平台扶手
工料机名称			单位	消耗量
人工	00010301	综合用工一类	工日	0.120
材料	1223000101	不锈钢扶手 ϕ75	m	1.0500
	1223000104	不锈钢弯头 ϕ75	个	0.8700
	03130309	不锈钢焊丝	kg	0.2300
	0301050510	螺栓 M22×80	个	10.0000
	14290002	氩气	m^3	0.0540
	34000011	其他材料费 占材料费	%	1.00
机械	99190015	切割机	台班	0.0200
	99460004	其他机具费 占人工费	%	1.50

北京市住房和城乡建设委员会

四、爬梯安装

工作内容:施工准备、运输、定位、打孔、化学锚栓安装、爬梯安装、调平、焊接、防锈防腐处理等。　　　　**单位:**t

编号				4-171	4-172
项目				钢爬梯	铝合金爬梯
工料机名称			单位	消耗量	
人工	00010301	综合用工一类	工日	8.867	17.042
材料	33010027	钢爬梯	t	1.0000	-
	03150710	镀锌铁丝 8#~12#	kg	6.0900	-
	03130101	电焊条（综合）	kg	15.1000	43.6390
	0301050802	不锈钢螺栓 M12×120	套	4.0800	-
	0301080703	化学螺栓及胶 M12	套	8.1600	-
	02010014	硅胶板	m^2	0.0240	-
	33010059	铝合金爬梯	t	-	1.0000
	34000011	其他材料费 占材料费	%	1.00	1.00
机械	99250008	电弧焊机	台班	2.5010	7.2279
	9907000202	轨道车 ≤210kW	台班	0.3161	0.6849
	99070005	轨道平车	台班	0.3161	0.6849
	9909000015	汽车式起重机 16t	台班	0.3162	0.6849
	9907000005	载重汽车 8t	台班	0.3162	0.6849
	99460004	其他机具费 占人工费	%	1.50	1.50

北京市住房和城乡建设委员会

五、疏散平台导向标识安装

工作内容：施工准备、运输、定位、固定、安装等。 **单位：**个

编号				4-173
项目				导向标识安装
工料机名称			单位	消耗量
人工	00010501	综合用工二类	工日	0.034
材料	36210004	导向标识	个	1.0100
	34000011	其他材料费 占材料费	%	1.00

北京市住房和城乡建设委员会

第六节 结构拆除

一、拆除混凝土

工作内容：1.拆除混凝土：拆除素混凝土或钢筋混凝土等。2.拆除隧道内混凝土：拆除素混凝土或钢筋混凝土、废料洞内及垂直运输至地面堆放等。

单位：m^3

编号				4-174	4-175	4-176	4-177
项目				拆除素混凝土	拆除钢筋混凝土	拆除隧道内素混凝土	拆除隧道内钢筋混凝土
工料机名称			单位	消耗量			
人工	00010501	综合用工二类	工日	0.990	1.672	1.237	2.090
材料	03151305	合金钢钻头	个	0.1000	0.1400	0.1000	0.1400
	01630001	工具钢	kg	0.3200	0.4000	0.3200	0.4000
	22450010	高压胶皮风管	m	0.1000	0.1000	0.1000	0.1000
	14290005-1	乙炔气	m^3	–	0.0300	–	0.0300
	14290003	氧气	m^3	–	0.1000	–	0.1000
	34000011	其他材料费 占材料费	%	1.00	1.00	1.00	1.00
机械	9943000007	电动空气压缩机 10m^3/min	台班	0.1800	0.2520	0.1800	0.2520
	99330001	风镐	台班	0.3600	0.5040	0.3600	0.5040
	9909000403	桥式起重机 10t	台班	–	–	0.0300	0.0300
	99310001	电动三轮车	台班	–	–	0.1500	0.1500
	99460004	其他机具费 占人工费	%	1.50	1.50	1.50	1.50

北京市住房和城乡建设委员会

二、水钻开孔

工作内容：准备、钻孔、清理孔洞等。　　　　**单位：**m

编号				4-178	4-179	4-180	4-181	4-182
项目				水钻开孔（孔径）				
				≤100mm	≤150mm	≤200mm	≤250mm	≤300mm
				砌体结构				
工料机名称			单位	消耗量				
人工	00010501	综合用工二类	工日	0.120	0.129	0.137	0.149	0.160
材料	0315130408	水钻钻头 ϕ150	个	–	0.1332	–	–	–
	0315130401	水钻钻头 ϕ100	个	0.1332	–	–	–	–
	0315130409	水钻钻头 ϕ250	个	–	–	–	0.1332	–
	0315130402	水钻钻头 ϕ200	个	–	–	0.1332	–	–
	0315130403	水钻钻头 ϕ300	个	–	–	–	–	0.1332
	34000011	其他材料费 占材料费	%	1.00	1.00	1.00	1.00	1.00
机械	9941000001	水钻 1.1kW	台班	0.1000	0.1070	0.1144	0.1240	0.1332
	99460004	其他机具费 占人工费	%	1.50	1.50	1.50	1.50	1.50

北京市住房和城乡建设委员会

工作内容：准备、钻孔、清理孔洞等。　　　　**单位：**m

编号				4-183	4-184	4-185	4-186	4-187
项目				水钻开孔（孔径）				
				≤100mm	≤150mm	≤200mm	≤250mm	≤300mm
				混凝土结构				
工料机名称			单位	消耗量				
人工	00010501	综合用工二类	工日	0.300	0.322	0.343	0.372	0.400
材料	0315130401	水钻钻头 ϕ100	个	0.3330	–	–	–	–
	0315130402	水钻钻头 ϕ200	个	–	–	0.3200	–	–
	0315130403	水钻钻头 ϕ300	个	–	–	–	–	0.3100
	0315130409	水钻钻头 ϕ250	个	–	–	–	0.3150	–
	0315130408	水钻钻头 ϕ150	个	–	0.3265	–	–	–
	34000011	其他材料费 占材料费	%	1.00	1.00	1.00	1.00	1.00
机械	9941000001	水钻 1.1kW	台班	0.2500	0.2680	0.2860	0.3100	0.3330
	99460004	其他机具费 占人工费	%	1.50	1.50	1.50	1.50	1.50

北京市住房和城乡建设委员会

第五章　砌筑工程

北京市住房和城乡建设委员会

北京市住房和城乡建设委员会

说　　明

一、本章包括:高架砌筑工程,地面车站砌筑工程,地下车站砌筑工程3节共22个子目。

二、墙体砌筑高度按6m以内编制,超过6m时,其超过部分的综合工日乘以系数1.1。

三、砌筑工程中墙体拉结筋、钢筋网片、预埋铁件等执行"第四章主体结构混凝土及钢筋工程"相应子目。

四、车站砌筑均按直形砌筑编制,设计为弧形的,按相应子目的人工乘以系数1.1,砖、砌块、石材及砂浆(粘结剂)用量乘以系数1.03。

五、砌块墙中的钢筋混凝土抱框柱、钢筋混凝土构造柱、钢筋混凝土水平系梁、钢筋混凝土过梁、钢筋混凝土圈梁等梁柱执行"第四章主体结构混凝土及钢筋工程"相应子目。

工程量计算规则

一、高架砌筑工程:浆砌片石及料石、浆砌混凝土预制块、砖砌体、零星砌砖、砌块砌体按设计图示尺寸以体积计算,不扣除嵌入砌体中钢筋、铁件、沉降缝、伸缩缝以及单孔面积≤$0.3m^2$的孔洞所占体积。

二、地面和地下车站砌筑工程

1.墙体按设计图示尺寸以体积计算。扣除门窗洞口、过人洞、空圈、嵌入墙内的钢筋混凝土柱、梁、圈梁、挑梁 、过梁及凹进墙内的壁龛、管槽、暖气槽、消火栓箱所占体积。

2.零星砌砖、零星蒸压加气混凝土砌块按设计图示尺寸以体积计算。

三、圆弧形墙按设计图示墙中心线长乘以高度再乘以厚度以体积计算。

北京市住房和城乡建设委员会

北京市住房和城乡建设委员会

第一节 高架砌筑工程

一、浆砌片石及料石

工作内容:清洗石料、砂浆拌制、运料、砌筑等。　　　　**单位:**m^3

编号				5-1	5-2	5-3	5-4	5-5
项目				浆砌片石				浆砌料石
				墩台身	挡土墙	护墙(坡)	基础	
工料机名称			单位	消耗量				
人工	00010301	综合用工一类	工日	1.226	0.896	1.270	0.760	1.765
材料	8001000615	普通干混砂浆 砌筑砂浆 DM10	m^3	0.3670	0.3670	0.3670	0.3670	0.4110
	04050026	片石	m^3	1.3400	1.3400	1.3400	1.3400	–
	04110002	料石	m^3	–	–	–	–	0.7252
	34000011	其他材料费 占材料费	%	1.00	1.00	1.00	1.00	1.00
机械	9905000303	干混砂浆搅拌机 200L	台班	0.0587	0.0587	0.0587	0.0587	0.0658
	9909000012	汽车式起重机 8t	台班	0.1300	–	0.1080	0.1080	0.1080
	99460004	其他机具费 占人工费	%	1.50	1.50	1.50	1.50	1.50

北京市住房和城乡建设委员会

二、浆砌混凝土预制块

工作内容：整理预制块料、砂浆拌制、运料、砌筑等。 **单位：**m³

编号				5-6	5-7
项目				挡墙	栏杆
工料机名称			单位	消耗量	
人工	00010301	综合用工一类	工日	0.145	2.702
材料	8001000615	普通干混砂浆 砌筑砂浆 DM10	m^3	0.0920	–
	0427002002	预制混凝土小型构件 C30	m^3	1.0200	1.0200
	8021000703	预拌豆石混凝土 C20	m^3	–	0.4580
	34000011	其他材料费 占材料费	%	1.00	1.00
机械	9905000303	干混砂浆搅拌机 200L	台班	0.0110	–
	9909000015	汽车式起重机 16t	台班	0.0400	0.0200
	99460004	其他机具费 占人工费	%	1.50	1.50

北京市住房和城乡建设委员会

三、砖砌体

工作内容：整理砖料、砂浆拌制、运料、砌筑等。 **单位：**m³

编号				5-8	5-9	5-10	5-11
项目				基础、护底	墩台	挡墙	零星砌砖
工料机名称			单位	消耗量			
人工	00010301	综合用工一类	工日	1.242	1.385	1.422	2.842
材料	8001000615	普通干混砂浆 砌筑砂浆 DM10	m³	0.2630	0.2630	0.2630	0.2453
	0413000901	标准砖 240mm×115mm×53mm	块	523.0000	523.0000	523.0000	554.0000
	34000011	其他材料费 占材料费	%	1.00	1.00	1.00	1.00
机械	9905000303	干混砂浆搅拌机 200L	台班	0.0280	0.0280	0.0280	0.0350
	9909000016	汽车式起重机 20t	台班	–	–	–	0.2280
	99460004	其他机具费 占人工费	%	1.50	1.50	1.50	1.50

北京市住房和城乡建设委员会

四、砌块砌体

工作内容：运料、砂浆拌合、砌筑等。 **单位：**m^3

编号				5-12	5-13	5-14
项目				蒸压加气混凝土砌块墙(厚度)		零星蒸压加气混凝土砌块砌筑
				≤200mm	>200mm	
工料机名称			单位	消耗量		
人工	00010301	综合用工一类	工日	1.477	1.472	2.842
材料	0415002606	蒸压加气混凝土砌块 600mm×300mm×250mm	m^3	–	0.9907	1.0074
	8001000615	普通干混砂浆 砌筑砂浆 DM10	m^3	0.1048	0.0981	0.1218
	0415002605	蒸压加气混凝土砌块 600mm×200mm×250mm	m^3	0.9907	–	–
	34000011	其他材料费 占材料费	%	1.00	1.00	1.00
机械	9905000303	干混砂浆搅拌机 200L	台班	0.0710	0.0710	0.0350
	9909000016	汽车式起重机 20t	台班	0.0840	0.0840	0.0840
	99460004	其他机具费 占人工费	%	1.50	1.50	1.50

北京市住房和城乡建设委员会

第二节 地面车站砌筑工程

一、砖砌体

工作内容：运料、清理基层、砂浆拌合、砌砖、勾缝等。　　　　**单位：**m^3

编		号		5-15
项		目		零星砌砖
工料机名称			单位	消耗量
人工	00010301	综合用工一类	工日	2.657
材料	0413000901	标准砖 240mm×115mm×53mm	块	554.0000
	8001000615	普通干混砂浆 砌筑砂浆 DM10	m^3	0.2453
	34000011	其他材料费 占材料费	%	1.00
机械	9905000303	干混砂浆搅拌机 200L	台班	0.0350
	99460004	其他机具费 占人工费	%	1.50

北京市住房和城乡建设委员会

二、砌块砌体

工作内容：运料、清理基层、砂浆拌合、砌筑等。 **单位：**m^3

编号				5-16	5-17	5-18
项目				蒸压加气混凝土砌块墙(厚度)		零星蒸压加气混凝土砌块砌筑
				≤200mm	>200mm	
工料机名称			单位	消耗量		
人工	00010301	综合用工一类	工日	1.381	1.376	2.657
材料	0415002605	蒸压加气混凝土砌块 600mm×200mm×250mm	m^3	0.9907	-	-
	8001000615	普通干混砂浆 砌筑砂浆 DM10	m^3	0.1048	0.0981	0.1218
	0415002606	蒸压加气混凝土砌块 600mm×300mm×250mm	m^3	-	0.9907	1.0074
	34000011	其他材料费 占材料费	%	1.00	1.00	1.00
机械	9905000303	干混砂浆搅拌机 200L	台班	0.0710	0.0710	0.0350
	99460004	其他机具费 占人工费	%	1.50	1.50	1.50

北京市住房和城乡建设委员会

第三节 地下车站砌筑工程

一、砖砌体

工作内容：运料、清理基层、砂浆拌合、砌砖、刮缝等。 **单位：**m^3

编		号		5-19
项		目		零星砌砖
工料机名称			单位	消耗量
人工	00010301	综合用工一类	工日	3.055
材料	8001000615	普通干混砂浆 砌筑砂浆 DM10	m^3	0.2453
	0413000901	标准砖 240mm×115mm×53mm	块	554.0000
	34000011	其他材料费 占材料费	%	1.00
机械	9905000303	干混砂浆搅拌机 200L	台班	0.0350
	99310001	电动三轮车	台班	0.1500
	9909000403	桥式起重机 10t	台班	0.0600
	99460004	其他机具费 占人工费	%	1.50

北京市住房和城乡建设委员会

二、砌块砌体

工作内容：运料、砂浆拌合、砌筑等。　　　　**单位：**m^3

编号				5-20	5-21	5-22
项目				蒸压加气混凝土砌块墙(厚度)		零星蒸压加气混凝土砌块砌筑
				≤200mm	>200mm	
工料机名称			单位	消	耗	量
人工	00010301	综合用工一类	工日	1.588	1.583	3.055
材料	0415002605	蒸压加气混凝土砌块 600mm×200mm×250mm	m^3	0.9907	–	–
	8001000615	普通干混砂浆 砌筑砂浆 DM10	m^3	0.1048	0.0981	0.1218
	0415002606	蒸压加气混凝土砌块 600mm×300mm×250mm	m^3	–	0.9907	1.0074
	34000011	其他材料费 占材料费	%	1.00	1.00	1.00
机械	9905000303	干混砂浆搅拌机 200L	台班	0.0710	0.0710	0.0350
	99310001	电动三轮车	台班	0.0900	0.0900	0.0900
	9909000403	桥式起重机 10t	台班	0.0300	0.0300	0.0300
	99460004	其他机具费 占人工费	%	1.50	1.50	1.50

北京市住房和城乡建设委员会

第六章　钢结构工程

北京市住房和城乡建设委员会

北京市住房和城乡建设委员会

说　　明

一、本章包括:高架钢结构,栏杆,钢管柱 3 节共 25 个子目。

二、本章预制构件场外运输费应包含在材料价格中。

三、钢箱梁制作安装按单跨重量 200t 以内编制,钢箱梁、钢桥梯不扣除孔眼的质量,焊条、铆钉、螺栓、焊缝等不另增加质量。

四、钢套筒和钢管柱分高度编制,其高度是指设计钢管柱顶标高与钢管柱底标高之差。

五、钢套筒主材按 3 次摊销编制,螺栓和法兰重量不另计算;若钢套筒无法拔出时,按一次性投入计算。

六、钢管柱主材按成品编制,螺栓重量不另计算。

工程量计算规则

一、高架钢结构按设计图示尺寸乘理论重量以质量计算。

二、钢栏杆制作安装按设计图示尺寸乘理论重量以质量计算。

三、不锈钢栏杆安装按设计图示尺寸以长度计算。

四、高架钢管柱,暗挖钢管柱,盖挖钢套筒和钢管柱按设计图示尺寸乘理论重量以质量计算。

五、定位器安装按设计图示以数量计算。

北京市住房和城乡建设委员会

北京市住房和城乡建设委员会

第一节 高架钢结构

工作内容：1.钢箱梁安装：场内运输、安装等。2.抗震锚栓制作安装：锚栓制作、场内运输、安装等。3.连接钢板制作安装：钢板或型钢制作、定位、场内运输、安装等。

单位：t

编号				6-1	6-2	6-3
项目				高架		
				钢箱梁安装	抗震锚栓制作安装	连接钢板制作安装
工料机名称			单位	消耗量		
人工	00010301	综合用工一类	工日	2.029	13.235	15.820
材料	33010075	型钢式梁	t	1.0000	–	–
	01010003	钢筋 ϕ10 以外	kg	–	1040.0000	–
	01000001-1	型钢 综合	kg	–	16.0000	–
	03130101	电焊条（综合）	kg	4.1100	12.6500	41.6600
	03010503-2	高强螺栓	个	12.2600	–	–
	03150906	铁件	kg	1.6500	–	–
	03110102	砂轮片	片	10.0000	–	–
	14290003	氧气	m^3	36.5000	–	30.4700
	14290005-1	乙炔气	m^3	12.1700	–	10.1600
	13050004	防锈漆	kg	–	6.6800	–
	0129001117	普通钢板 δ8mm～15mm	kg	–	–	1060.0000
	34000011	其他材料费 占材料费	%	1.00	1.00	1.00
机械	9925000202	交流电焊机 32kV·A	台班	0.3425	1.0540	3.4700
	9925000017	电焊条烘干箱 60×50×75cm^3	台班	–	0.0900	–
	99250003	电焊条恒温箱	台班	–	0.0900	–
	9909000008	履带式起重机 200t	台班	–	0.0150	–
	9943000007	电动空气压缩机 10m^3/min	台班	0.0500	–	–
	9909000016	汽车式起重机 20t	台班	–	–	0.1200
	99190015	切割机	台班	–	–	0.1800
	9909000023	汽车式起重机 160t	台班	0.0100	–	–
	99460004	其他机具费 占人工费	%	1.50	1.50	1.50

北京市住房和城乡建设委员会

工作内容：人行天桥钢主梁和钢梯场内运输、就位、拼装、固定等。　　　　**单位：**t

编号				6-4	6-5
项目				高架人行天桥	
				钢主梁安装	钢桥梯安装
工料机名称			单位	消耗量	
人工	00010301	综合用工一类	工日	2.029	1.655
材料	33010064	人行天桥钢箱梯、坡梁	t	–	1.0000
	01000001-1	型钢 综合	kg	8.6000	–
	03150906	铁件	kg	2.7970	2.7970
	14290003	氧气	m^3	42.0000	29.9000
	14290005-1	乙炔气	m^3	14.0000	9.9700
	03130101	电焊条（综合）	kg	3.3000	2.3500
	3301002501	箱型梁 主梁	t	1.0000	–
	01010002-1	钢筋 ϕ10 以内	kg	11.4000	11.4000
	03110102	砂轮片	片	10.0000	10.0000
	34000011	其他材料费 占材料费	%	1.00	1.00
机械	9925000202	交流电焊机 32kV·A	台班	0.2780	0.1960
	9909000037	汽车式起重机 80t	台班	0.0400	–
	9909000026	汽车式起重机 40t	台班	–	0.0800
	99460004	其他机具费 占人工费	%	1.50	1.50

北京市住房和城乡建设委员会

第二节 栏杆

工作内容：选料、切口、挖孔、切割、制作、安装、焊接、校正固定等。　　　　**单位：**见表

编号				6-6	6-7	6-8	6-9
项目				高架		地下	
				钢栏杆制作安装	不锈钢栏杆安装	钢栏杆制作安装	不锈钢栏杆安装
				t	m	t	m
工料机名称			单位	消耗量			
人工	00010301	综合用工一类	工日	21.508	0.214	23.659	0.235
材料	01000001-1	型钢 综合	kg	1020.0000	–	1020.0000	–
	12210003	复合不锈钢管栏杆	m	–	1.0000	–	1.0000
	14290003	氧气	m^3	28.7400	–	28.7400	–
	14290005-1	乙炔气	m^3	9.5800	–	9.5800	–
	13050004	防锈漆	kg	17.0000	–	17.0000	–
	03130101	电焊条（综合）	kg	24.9900	–	24.9900	–
	03130111	不锈钢电焊条	kg	–	0.2900	–	0.2900
	34000011	其他材料费 占材料费	%	1.00	1.00	1.00	1.00
机械	9909000016	汽车式起重机 20t	台班	0.1020	0.0030	–	–
	9925000202	交流电焊机 32kV·A	台班	2.0800	0.0240	2.0800	0.0240
	9909000403	桥式起重机 10t	台班	–	–	0.2520	0.0070
	9925000017	电焊条烘干箱 60×50×75cm^3	台班	–	0.0096	–	0.0096
	99250003	电焊条恒温箱	台班	–	0.0096	–	0.0096
	99460004	其他机具费 占人工费	%	1.50	1.50	1.50	1.50

北京市住房和城乡建设委员会

第三节 钢管柱

一、高架钢管柱

工作内容：场内运输、定位、吊装、螺栓连接、安装就位等。 **单位：**t

编		号		6-10
项		目		钢管柱
工料机名称			单位	消耗量
人工	00010301	综合用工一类	工日	3.930
材料	01000001-1	型钢 综合	kg	54.0000
	3301003001	钢管柱 ＞ 3t	t	1.0000
	05030006	木方	m^3	0.0300
	0301050216	六角螺栓 M16×250	个	12.5500
	03130101	电焊条（综合）	kg	1.5800
	14290003	氧气	m^3	9.8100
	14290005-1	乙炔气	m^3	3.8260
	34000011	其他材料费 占材料费	%	1.00
机械	9907000006	载重汽车 10t	台班	0.0520
	9909000006	履带式起重机 40t	台班	0.1600
	9925000203	交流电焊机 40kV·A	台班	0.1317
	99460004	其他机具费 占人工费	%	1.50

北京市住房和城乡建设委员会

二、暗挖车站隧道内钢管柱

工作内容：材料场内运输、定位、吊装、对接、螺栓连接、校正等。　　　　**单位：**t

编号				6-11	6-12	6-13	6-14	6-15
项目				隧道内钢管柱安装(直径)				
				≤800mm	≤200mm	≤1600mm	≤1800mm	≤2000mm
工料机名称			单位	消耗量				
人工	00010304	综合用工一类	工日	8.496	8.243	7.998	7.760	7.534
材料	33010025	钢管柱	t	1.0000	1.0000	1.0000	1.0000	1.0000
	01000001-1	型钢 综合	kg	12.4500	13.0725	13.7261	14.4124	15.1330
	03010503-2	高强螺栓	个	5.0000	4.7500	4.5125	4.2869	4.0725
	0301050931	镀锌带母螺栓 M20×(180~300)	套	20.0000	19.0000	18.0500	17.1475	16.2901
	03130101	电焊条（综合）	kg	2.0296	1.9264	1.8146	1.7093	1.6101
	34000011	其他材料费 占材料费	%	1.00	1.00	1.00	1.00	1.00
机械	9925000202	交流电焊机 32kV·A	台班	0.1690	0.1600	0.1510	0.1420	0.1340
	9909000302	龙门起重机 30t	台班	0.1500	0.1500	0.1500	0.1500	0.1500
	9909000403	桥式起重机 10t	台班	0.0820	0.0820	0.0820	0.0820	0.0820
	99310001	电动三轮车	台班	0.2050	0.2050	0.2050	0.2050	0.2050
	99460004	其他机具费 占人工费	%	1.50	1.50	1.50	1.50	1.50

北京市住房和城乡建设委员会

三、盖挖车站钢管柱与钢套筒

工作内容：钢管柱场内运输、定位、吊装、螺栓连接、安装就位等。 **单位：**t

编号				6-16	6-17	6-18
项目				钢管柱安装(高度)		
				≤20m	≤30m	≤40m
工料机名称			单位	消耗量		
人工	00010304	综合用工一类	工日	4.080	4.284	4.500
材料	01000001-1	型钢 综合	kg	12.4500	12.4500	12.4500
	3301003001	钢管柱 ＞ 3t	t	1.0000	1.0000	1.0000
	03010503-2	高强螺栓	个	12.0000	12.0000	12.0000
	03010101-2	铆钉	kg	23.7400	23.7400	23.7400
	03130101	电焊条（综合）	kg	1.0500	1.0500	1.0500
	34000011	其他材料费 占材料费	%	1.00	1.00	1.00
机械	9907000006	载重汽车 10t	台班	0.0520	0.0520	0.0520
	9925000202	交流电焊机 32kV·A	台班	0.0875	0.0875	0.0875
	9909000031	履带式起重机 20t	台班	0.2200	-	-
	9909000006	履带式起重机 40t	台班	0.2200	0.1900	-
	9909000032	履带式起重机 60t	台班	-	0.1900	0.1600
	9909000007	履带式起重机 100t	台班	-	-	0.1600
	99460004	其他机具费 占人工费	%	1.50	1.50	1.50

北京市住房和城乡建设委员会

工作内容：钢套筒场内运输、定位、吊装、螺栓连接、安装就位等。　　　　**单位：**t

编号				6-19	6-20	6-21
项目				钢套筒安装(高度)		
				≤20m	≤30m	≤40m
工料机名称			单位	消耗量		
人工	00010304	综合用工一类	工日	3.390	3.729	4.102
材料	18290001-2	钢套筒	t	0.3400	0.3400	0.3400
	0301050624	带母螺栓 M20×(85~100)	套	10.0319	10.0319	10.0319
	03130101	电焊条（综合）	kg	1.9680	1.9680	1.9680
	20010021	钢制法兰盘	kg	30.0000	30.0000	30.0000
	34000011	其他材料费 占材料费	%	1.00	1.00	1.00
机械	9907000006	载重汽车 10t	台班	0.0520	0.0520	0.0520
	9925000202	交流电焊机 32kV·A	台班	0.1640	0.1640	0.1640
	9909000031	履带式起重机 20t	台班	0.2200	-	-
	9909000006	履带式起重机 40t	台班	0.0700	0.1900	-
	9909000032	履带式起重机 60t	台班	-	0.0660	0.1600
	9909000007	履带式起重机 100t	台班	-	-	0.0600
	99460004	其他机具费 占人工费	%	1.50	1.50	1.50

北京市住房和城乡建设委员会

工作内容：1.钢套筒拆除：拆除、吊运、清理堆放等。2.定位器安装：材料场内运输、定位、吊装、安装就位等。 **单位：**见表

编号				6-22	6-23	6-24	6-25
项目				钢套筒拆除		定位器安装（直径）	
				吊拔	切割	≤800mm	≤1200mm
				t		套	
工料机名称			单位	消耗量			
人工	00010304	综合用工一类	工日	1.890	1.956	1.122	1.140
材料	37250001	定位器	kg	-	-	246.4900	354.9456
	0301070011	膨胀螺栓 M20	套	-	-	4.0000	4.0000
	14290003	氧气	m^3	-	5.0000	-	-
	14290005-1	乙炔气	m^3	-	1.2500	-	-
	34000011	其他材料费 占材料费	%	1.00	1.00	1.00	1.00
机械	9943000801	千斤顶 300t	台班	0.2000	-	-	-
	9909000021	汽车式起重机 100t	台班	0.0500	-	-	-
	99230024	割炬	台班	-	0.8860	-	-
	99310001	电动三轮车	台班	-	0.3600	-	-
	9907000006	载重汽车 10t	台班	-	-	0.0500	0.0500
	99460004	其他机具费 占人工费	%	1.50	1.50	1.50	1.50

北京市住房和城乡建设委员会

第七章　盾构工程

北京市住房和城乡建设委员会

北京市住房和城乡建设委员会

说　　明

一、本章包括：盾构机安装与拆除，车架安装与拆除，盾构掘进，管片密封条与嵌缝，衬砌压浆，柔性接缝，负环段管片与隧道内管线拆除，盾构其他工程 8 节共 105 个子目。

二、盾构掘进子目按综合土质编制，遇漂石、岩石以及其他特殊地层时，应按实际方案计算。

三、盾构工程中"ф"是指盾构设计图示结构（管片）外径。

四、车架安拆子目按单台车的重量划分。

五、土压平衡盾构掘进的土方以吊出井口至堆土场地为止，场外运输执行本册第一章相应子目。

六、泥水平衡盾构掘进子目中不含泥浆制作与处理系统，应按实际方案计算；其渣土（泥浆）的场外运输应执行本册第一章相应子目。

七、盾构掘进子目包含预制混凝土盾构管片，采用钢管片的应调整材料并删除材料中的管片连接螺栓。预制混凝土管片及钢管片按成品市场价格计入（预制混凝土管片价格应包含管片混凝土、钢筋、管片螺栓预埋钢垫片及管片运至施工现场的运输费用；钢管片价格应包含管片安装螺栓和管片运至施工现场的运输费用）。

八、管片密封条分氯丁橡胶条和三元乙丙橡胶条两种，设计与本标准不同时，可调整。

九、负环段管片拆除按工作井上方可直接吊拆的施工情况编制，其他工况应按实际方案计算。

十、盾构基座用于盾构机组装、始发和接收阶段，按钢结构编制，若采用混凝土基座，应执行明挖车站混凝土结构相应子目。

十一、同步压浆中的浆液类型及配比与设计不同时，可调整。

十二、洞口钢筋混凝土环圈已包含钢筋制作安装与模板等内容，不另计算。

北京市住房和城乡建设委员会

十三、盾构过站按长度260m车站编制,盾构过工作井按长度20m编制,盾构平移按长度50m编制,长度不同时可按比例调整。

十四、盾构机穿越既有建(构)筑物、公(铁)路或其他设施以及对地表沉降有特殊要求所产生的措施费用应另行计算。

工程量计算规则

一、盾构机吊装吊拆按设计安拆次数计算。

二、车架安装与拆除按设计方案以单线盾构配套的台车数量计算。

三、盾构掘进按以下标准以长度计算:

1.负环段:从拼装后靠管片起至盾尾离开工作井内壁止(始发井长度)。

2.始发段:从盾尾离开工作井内壁至盾尾距工作井内壁10倍盾构结构外径止。

3.正常段:从始发段掘进结束至到达段掘进开始的全段长度。

4.到达段:按刀盘距接收工作井外壁5倍盾构结构外径长度计算。

四、管片设置密封条和管片嵌缝均按设计图示以环计算。

五、同步压浆按设计管片外径和盾构壳体最大外径所形成的充填体积计算,分块压浆按设计要求以注浆体积计算。

六、临时防水环板和钢环板按设计图示尺寸乘理论重量以质量计算。

七、临时止水缝和柔性接缝环按每环结构中心线周长以长度计算。

八、拆除临时钢环板按设计图示尺寸乘理论重量以质量计算。

九、拆除洞口环管片按设计图示尺寸以体积计算。

北京市住房和城乡建设委员会

十、洞口钢筋混凝土环圈按设计图示尺寸以体积计算。

十一、负环管片拆除以负环段长度计算。

十二、隧道内管线拆除按隧道设计长度加 50m 计算。

十三、盾构基座制作安装按设计图示尺寸乘理论重量以质量计算。

十四、手孔封堵按设计图示以数量计算。

十五、密封舱添加材料按盾构掘进长度计算。

十六、盾构过站、过工作井、平移、调头按设计过站、过工作井及平移、调头次数以“台·次”计算。

北京市住房和城乡建设委员会

北京市住房和城乡建设委员会

第一节 盾构机安装与拆除

工作内容: 1.盾构吊装:起吊机械设备就位、盾构吊入井底基座、盾构安装、调试等。2.盾构吊拆:拆除盾构与车架连杆、起吊机械及附属设备就位、盾构机吊出井口、上托架装车等。

单位: 台·次

编号				7-1	7-2	7-3	7-4
项目				盾构吊装	盾构吊拆	盾构吊装	盾构吊拆
				$\phi \leqslant 7000mm$		$\phi \leqslant 11000mm$	
工料机名称			单位	消耗量			
人工	00010504	综合用工二类	工日	205.174	163.864	615.520	491.590
材料	01000001-1	型钢 综合	kg	760.0000	530.0000	2280.0000	1590.0000
	0129001115	普通钢板 δ3.5mm~4.0mm	kg	550.0000	330.0000	1650.0000	990.0000
	37090002	盾构托架	t	0.9200	0.7400	2.7600	2.2200
	01050001	钢丝绳	kg	190.0000	190.0000	570.0000	570.0000
	03130101	电焊条(综合)	kg	51.0000	25.5000	153.0000	76.5000
	02010002	橡胶板 δ1mm~3mm	kg	16.3000	-	48.9000	-
	14290005-1	乙炔气	m^3	-	40.0000	-	120.0000
	14290003	氧气	m^3	-	120.0000	-	360.0000
	34000011	其他材料费 占材料费	%	1.00	1.00	1.00	1.00
机械	9909000021	汽车式起重机 100t	台班	18.0000	14.4000	-	-
	9909000008	履带式起重机 200t	台班	8.0000	6.4000	-	-
	9909000403	桥式起重机 10t	台班	5.2000	4.1600	15.6000	12.4800
	9925000202	交流电焊机 32kV·A	台班	4.2500	2.1250	12.7500	6.3750
	9909000023	汽车式起重机 160t	台班	-	-	24.0000	20.4000
	9909000009	履带式起重机 300t	台班	-	-	14.0000	12.4000
	99460004	其他机具费 占人工费	%	1.00	1.00	1.00	1.00

北京市住房和城乡建设委员会

第二节 车架安装与拆除

工作内容:1.整体始发:车架吊入井底、井下组装就位与盾构连接、车架上设备、水电气管安装等。2.分体始发:车架地面安装、延长管线安装、延长管线拆除、车架吊入井底、车架隧道内安装等。3.车架拆除:车架、车架设备和管道拆除,吊运出井等。 **单位:**节

编号				7-5	7-6	7-7	7-8	7-9	7-10
项目				车架安装				车架拆除	
				整体始发		分体始发		≤30t	>30t
				≤30t	>30t	≤30t	>30t		
工料机名称			单位	消耗量					
人工	00010504	综合用工二类	工日	16.256	19.508	47.144	56.572	14.630	17.556
材料	01290034	钢板(中厚)	kg	300.0000	360.0000	300.0000	360.0000	169.0000	208.0000
	37010009	轻轨	kg	127.5000	130.0000	127.5000	130.0000	-	-
	37050005	枕木	m^3	0.2813	0.3125	0.2813	0.3125	0.3220	0.3640
	03010554	带帽螺栓	套	38.4000	39.2000	38.4000	39.2000	-	-
	03130101	电焊条(综合)	kg	10.8000	16.2000	21.6000	32.4000	5.4000	8.1000
	14290005-1	乙炔气	m^3	2.8930	3.1560	5.7860	9.4680	3.7620	4.1040
	14290003	氧气	m^3	8.6790	9.4680	17.3580	28.4040	11.2860	12.3120
	1727000108	高压胶管 $\phi50$	m	-	-	173.3330	208.0000	-	-
	1703000008-1	镀锌钢管 *DN*50	m	-	-	67.5000	81.0000	-	-
	1703000016	镀锌钢管 *DN*125	m	-	-	7.6250	9.1500	-	-
	2811000501	动力电缆 YC3×120+2×70	m	-	-	112.5000	135.0000	-	-
	2803001301	控制电缆 KkV-24×1m^2	m	-	-	498.3330	598.0000	-	-
	34000011	其他材料费 占材料费	%	1.00	1.00	1.00	1.00	1.00	1.00
机械	9909000007	履带式起重机 100t	台班	0.6580	0.7900	1.3160	1.5800	0.6250	0.7503
	9909000008	履带式起重机 200t	台班	-	-	0.7500	0.9000	-	-
	99090007	门式起重机 50t 以内	台班	0.7436	0.8920	1.4870	1.7840	0.7064	0.8476
	9909000403	桥式起重机 10t	台班	0.3625	0.4350	0.7250	0.8700	0.2900	0.3480
	9925000202	交流电焊机 32kV·A	台班	0.9000	1.3500	1.8000	2.7000	0.4500	0.6750
	99460004	其他机具费 占人工费	%	1.00	1.00	1.00	1.00	1.00	1.00

北京市住房和城乡建设委员会

第三节 盾构掘进

一、ϕ6000 土压平衡式盾构掘进

工作内容：盾构掘进、出土、管片洞内运输、拼装、连接螺栓紧固、装拉杆、施工管线铺设（照明、运输、供气、通风、通信）、土方吊运至地面堆放等。

单位：m

编号				7-11	7-12	7-13	7-14
项目				ϕ6000 土压平衡式盾构掘进			
				负环段	始发段	正常段	到达段
工料机名称			单位	消耗量			
人工	00010504	综合用工二类	工日	20.742	10.910	5.866	7.426
材料	03010551	管片连接螺栓	kg	32.0000	64.0000	64.0000	64.0000
	37010009	轻轨	kg	8.7700	8.7700	8.7700	8.7700
	37050004	钢轨枕	kg	11.2900	11.2900	11.2900	11.2900
	33350011	走道板	kg	16.0400	16.0400	16.0400	16.0400
	03150037	金属支架	kg	10.1300	10.1300	10.1300	10.1300
	1701000445	钢管 80mm	kg	4.3700	4.3700	4.3700	4.3700
	12210001	钢管栏杆	kg	8.1500	8.1500	8.1500	8.1500
	8021000803	预拌混凝土 C20	m^3	0.4600	–	–	–
	35030011	钢支撑	kg	147.7000	–	–	–
	22450007	风管	kg	18.5400	18.5400	18.5400	18.5400

北京市住房和城乡建设委员会

续前

编号				7-11	7-12	7-13	7-14
项目				ϕ6000 土压平衡式盾构掘进			
				负环段	始发段	正常段	到达段
材料	0313010302	电焊条 结 422 ϕ4	kg	8.0100	4.0050	4.0050	4.0050
	14090020	油脂	kg	–	26.6700	26.6700	26.6700
	14070003	机油	kg	42.6300	40.5800	21.0600	27.1600
	0429000601	混凝土盾构管片 ϕ6000mm	m	1.0100	1.0100	1.0100	1.0100
	2811000125	电缆 YC-3×35^2+2×16^2	m	0.4200	0.4200	0.4200	0.4200
	34000011	其他材料费 占材料费	%	1.00	1.00	1.00	1.00
机械	9909000105	门式起重机 50t	台班	0.2083	0.3385	0.2734	0.3149
	9935000203	刀盘式土压平衡盾构机 ϕ6000	台班	0.2498	0.4058	0.3278	0.3776
	9943000207	空压机 20m^3/min	台班	1.3000	–	–	–
	99440005	单机离心清水泵 ≤60	台班	0.7700	0.6800	0.6500	0.7250
	9945000006	轴流通风机 7.5kW	台班	1.5400	1.3600	1.3000	1.4500
	9925000202	交流电焊机 32kV·A	台班	0.6675	0.3340	0.3340	0.3340
	99430010	硅整流充电机	台班	–	0.3100	0.1620	0.2093
	9907000402	轨道式电瓶车 45t	台班	–	0.3100	0.1620	0.2093
	99460004	其他机具费 占人工费	%	2.00	2.00	3.00	2.00

北京市住房和城乡建设委员会

二、$\phi \leq 7000$ 土压平衡式盾构掘进

工作内容：盾构掘进、出土、管片洞内运输、拼装、连接螺栓紧固、装拉杆、施工管线铺设（照明、运输、供气、通风、通信）、土方吊运至地面堆放等。

单位：m

编号				7-15	7-16	7-17	7-18
项目				$\phi \leq 7000$ 土压平衡式盾构掘进			
				负环段	始发段	正常段	到达段
工料机名称			单位	消耗量			
人工	00010504	综合用工二类	工日	23.854	12.547	6.746	8.540
材料	03010551	管片连接螺栓	kg	37.1200	74.2400	74.2400	74.2400
	37010009	轻轨	kg	9.6900	9.6900	9.6900	9.6900
	37050004	钢轨枕	kg	19.0500	19.0500	19.0500	19.0500
	33350011	走道板	kg	20.2700	20.2700	20.2700	20.2700
	03150037	金属支架	kg	14.5800	14.5800	14.5800	14.5800
	1701000445	钢管 80mm	kg	4.3700	4.3700	4.3700	4.3700
	12210001	钢管栏杆	kg	8.1500	8.1500	8.1500	8.1500
	8021000803	预拌混凝土 C20	m^3	0.5600	–	–	–
	35030011	钢支撑	kg	209.9000	–	–	–
	22450007	风管	kg	20.4400	20.4400	20.4400	20.4400
	0313010302	电焊条 结 422 $\phi 4$	kg	8.4000	4.2000	4.2000	4.2000
	14090020	油脂	kg	–	31.1900	31.1900	31.1900
	14070003	机油	kg	45.6300	43.5800	24.0600	30.1600
	0429000602	混凝土盾构管片 ϕ7000mm 以内	m	1.0100	1.0100	1.0100	1.0100
	2811000125	电缆 YC-$3\times35^2+2\times16^2$	m	0.4200	0.4200	0.4200	0.4200
	34000011	其他材料费 占材料费	%	1.00	1.00	1.00	1.00
机械	9909000105	门式起重机 50t	台班	0.2083	0.3385	0.2734	0.3149
	9943000207	空压机 $20m^3$/min	台班	1.5400	–	–	–
	99440005	单机离心清水泵 ≤60	台班	0.8850	0.8300	0.7800	0.8600
	9945000006	轴流通风机 7.5kW	台班	1.7700	1.6600	1.5600	1.7200
	9925000202	交流电焊机 32kV·A	台班	0.7000	0.3500	0.3500	0.3500
	99430010	硅整流充电机	台班	–	0.3751	0.1960	0.2532
	9907000403	轨道式电瓶车 50t	台班	–	0.3410	0.1782	0.2302
	9935000204	刀盘式土压平衡盾构机 ϕ7000	台班	0.2523	0.4099	0.3311	0.3814
	99460004	其他机具费 占人工费	%	2.00	2.00	3.00	2.00

北京市住房和城乡建设委员会

三、$\phi \leqslant 8000$ 土压平衡式盾构掘进

工作内容：盾构掘进、出土、管片洞内运输、拼装、连接螺栓紧固、装拉杆、施工管线铺设（照明、运输、供气、通风、通信）、土方吊运至地面堆放等。

单位：m

编号				7-19	7-20	7-21	7-22
项目				$\phi \leqslant 8000$ 土压平衡式盾构掘进			
				负环段	始发段	正常段	到达段
工料机名称			单位	消耗量			
人工	00010504	综合用工二类	工日	27.135	14.132	7.461	9.525
材料	14070003	机油	kg	48.6300	46.5800	27.0600	33.1600
	03010551	管片连接螺栓	kg	42.2400	84.4800	84.4800	84.4800
	37010009	轻轨	kg	9.6900	9.6900	9.6900	9.6900
	37050004	钢轨枕	kg	19.0500	19.0500	19.0500	19.0500
	33350011	走道板	kg	20.2700	20.2700	20.2700	20.2700
	03150037	金属支架	kg	19.0300	19.0300	19.0300	19.0300
	1701000445	钢管 80mm	kg	4.3700	4.3700	4.3700	4.3700
	12210001	钢管栏杆	kg	8.1500	8.1500	8.1500	8.1500
	8021000803	预拌混凝土 C20	m^3	0.5600	–	–	–
	35030011	钢支撑	kg	239.9000	–	–	–
	22450007	风管	kg	22.4840	22.4840	22.4840	22.4840
	0313010302	电焊条 结 422 $\phi 4$	kg	8.7000	4.3500	4.3500	4.3500
	14090020	油脂	kg	–	36.4611	36.4611	36.4611
	0429000603	混凝土盾构管片 $\phi 8000$mm 以内	m	1.0100	1.0100	1.0100	1.0100
	2811000125	电缆 YC−$3\times35^2+2\times16^2$	m	0.4200	0.4200	0.4200	0.4200
	34000011	其他材料费 占材料费	%	1.00	1.00	1.00	1.00
机械	9909000128	门式起重机 60t	台班	0.1812	0.2944	0.2378	0.2740
	9943000207	空压机 $20m^3/min$	台班	1.8172	–	–	–
	99440005	单机离心清水泵 ≤60	台班	1.0178	0.9545	0.8970	0.9890
	9945000006	轴流通风机 7.5kW	台班	2.0355	1.9090	1.7940	1.9780
	9925000202	交流电焊机 32kV·A	台班	0.7250	0.3625	0.3625	0.3625
	99430010	硅整流充电机	台班	–	0.4951	0.2587	0.3342
	9907000404	轨道式电瓶车 60t	台班	–	0.3751	0.1960	0.2532
	9935000205	刀盘式土压平衡盾构机 $\phi 8000$	台班	0.2548	0.4140	0.3344	0.3852
	99460004	其他机具费 占人工费	%	2.00	2.00	3.00	2.00

北京市住房和城乡建设委员会

四、$\phi\leqslant9000$ 土压平衡式盾构掘进

工作内容:盾构掘进、出土、管片洞内运输、拼装、连接螺栓紧固、装拉杆、施工管线铺设(照明、运输、供气、通风、通信)、土方吊运至地面堆放等。

单位:m

编号				7-23	7-24	7-25	7-26
项目				$\phi\leqslant9000$ 土压平衡式盾构掘进			
				负环段	始发段	正常段	到达段
工料机名称			单位	消耗量			
人工	00010504	综合用工二类	工日	31.115	16.162	8.490	10.863
材料	14070003	机油	kg	51.6300	49.5800	30.0600	36.1600
	03010551	管片连接螺栓	kg	47.3600	94.7200	94.7200	94.7200
	37010009	轻轨	kg	9.6900	9.6900	9.6900	9.6900
	37050004	钢轨枕	kg	19.0500	19.0500	19.0500	19.0500
	33350011	走道板	kg	20.2700	20.2700	20.2700	20.2700
	03150037	金属支架	kg	23.4800	23.4800	23.4800	23.4800
	1701000445	钢管 80mm	kg	4.3700	4.3700	4.3700	4.3700
	12210001	钢管栏杆	kg	8.1500	8.1500	8.1500	8.1500
	8021000803	预拌混凝土 C20	m^3	0.6600	–	–	–
	35030011	钢支撑	kg	269.9000	–	–	–
	22450007	风管	kg	24.7324	24.7324	24.7324	24.7324
	0313010302	电焊条 结 422 $\phi4$	kg	9.0000	4.5000	4.5000	4.5000
	14090020	油脂	kg	–	42.6230	42.6230	42.6230
	0429000604	混凝土盾构管片 ϕ9000mm 以内	m	1.0100	1.0100	1.0100	1.0100
	2811000125	电缆 YC-$3\times35^2+2\times16^2$	m	0.4200	0.4200	0.4200	0.4200
	34000011	其他材料费 占材料费	%	1.00	1.00	1.00	1.00
机械	9909000128	门式起重机 60t	台班	0.1812	0.2944	0.2378	0.2740
	9943000207	空压机 $20m^3$/min	台班	2.1443	–	–	–
	99440005	单机离心清水泵 ≤60	台班	1.1704	1.0977	1.0316	1.1374
	9945000006	轴流通风机 7.5kW	台班	2.3408	2.1954	2.0631	2.2747
	9925000202	交流电焊机 32kV·A	台班	0.7500	0.3750	0.3750	0.3750
	99430010	硅整流充电机	台班	–	0.7244	0.3785	0.4890
	9907000111	轨道式电瓶车 80t	台班	–	0.4126	0.2156	0.2785
	9935000206	刀盘式土压平衡盾构机 ϕ9000	台班	0.2573	0.4181	0.3377	0.3890
	99460004	其他机具费 占人工费	%	2.00	2.00	3.00	2.00

北京市住房和城乡建设委员会

五、$\phi\leqslant11000$ 土压平衡式盾构掘进

工作内容： 盾构掘进、出土、管片洞内运输、拼装、连接螺栓紧固、装拉杆、施工管线铺设（照明、运输、供气、通风、通信）、土方吊运至地面堆放等。

单位： m

编号				7-27	7-28	7-29	7-30
项目				$\phi\leqslant11000$ 土压平衡式盾构掘进			
				负环段	始发段	正常段	到达段
工料机名称			单位	消耗量			
人工	00010504	综合用工二类	工日	58.871	28.752	15.276	19.512
材料	14070003	机油	kg	54.6300	52.5800	33.0600	39.1600
	37010009	轻轨	kg	26.0600	26.0600	26.0600	26.0600
	37050004	钢轨枕	kg	29.1800	29.1800	29.1800	29.1800
	33350011	走道板	kg	31.0000	31.0000	31.0000	31.0000
	03150037	金属支架	kg	35.7200	35.7200	35.7200	35.7200
	1701000445	钢管 80mm	kg	16.9600	16.9600	16.9600	16.9600
	12210001	钢管栏杆	kg	23.7200	23.7200	23.7200	23.7200
	8021000803	预拌混凝土 C20	m^3	0.6600	–	–	–
	35030011	钢支撑	kg	322.2200	–	–	–
	22450007	风管	kg	40.8800	40.8800	40.8800	40.8800
	0313010302	电焊条 结 422 $\phi4$	kg	9.1650	4.5820	4.5820	4.5820
	14090020	油脂	kg	–	48.9300	48.9300	48.9300
	0301050302	管片连接螺栓 M36	套	29.0000	58.0000	58.0000	58.0000
	2811000125	电缆 $YC-3\times35^2+2\times16^2$	m	0.4200	0.4200	0.4200	0.4200
	0429000605	混凝土盾构管片 $\phi11000$mm 以内	m	1.0100	1.0100	1.0100	1.0100
	34000011	其他材料费 占材料费	%	1.00	1.00	1.00	1.00
机械	9943000207	空压机 $20m^3/min$	台班	2.6800	–	–	–
	99440005	单机离心清水泵 ≤60	台班	1.3400	1.2700	1.2200	1.3100
	9945000006	轴流通风机 7.5kW	台班	2.6800	2.5400	2.4400	2.6200
	9925000202	交流电焊机 32kV·A	台班	0.7630	0.3815	0.3815	0.3815
	99430010	硅整流充电机	台班	–	0.8693	0.4543	0.5867
	9909000106	门式起重机 150t	台班	0.2656	0.4315	0.3485	0.4015
	9935000207	刀盘式土压平衡盾构机 $\phi11000$	台班	0.3045	0.4946	0.3995	0.4602
	9907000111	轨道式电瓶车 80t	台班	–	0.4951	0.2587	0.3342
	99460004	其他机具费 占人工费	%	2.00	2.00	3.00	2.00

北京市住房和城乡建设委员会

六、ϕ6000 泥水平衡式盾构掘进

工作内容：盾构掘进、管片洞内运输、拼装、连接螺栓紧固、装拉杆、施工管线路铺设（照明、运输、供气、通风、通信）、排泥水至沉淀池等。

单位：m

编号				7-31	7-32	7-33	7-34
项目				ϕ6000 泥水平衡式盾构掘进			
				负环段	始发段	正常段	到达段
工料机名称			单位	消耗量			
人工	00010504	综合用工二类	工日	24.618	15.013	6.981	11.653
材料	03010551	管片连接螺栓	kg	32.0000	64.0000	64.0000	64.0000
	37010009	轻轨	kg	6.7200	6.7200	6.7200	6.7200
	37050004	钢轨枕	kg	10.2700	10.2700	10.2700	10.2700
	33350011	走道板	kg	16.0400	16.0400	16.0400	16.0400
	03150037	金属支架	kg	10.1300	10.1300	10.1300	10.1300
	12210001	钢管栏杆	kg	8.1500	8.1500	8.1500	8.1500
	8021000803	预拌混凝土 C20	m^3	0.4600	–	–	–
	35030011	钢支撑	kg	147.7000	–	–	–
	22450007	风管	kg	18.5400	18.5400	18.5400	18.5400
	0313010302	电焊条 结 422 ϕ4	kg	8.0100	4.0050	4.0050	4.0050
	14090020	油脂	kg	–	26.6700	26.6700	26.6700
	14070003	机油	kg	38.1400	35.7700	14.7400	27.6200
	1703000018	镀锌钢管 *DN*200	m	1.3118	1.3118	1.3118	1.3118
	1703000012	镀锌钢管 *DN*80	m	1.0200	1.0200	1.0200	1.0200
	2811000125	电缆 YC−$3\times35^2+2\times16^2$	m	0.4200	0.4200	0.4200	0.4200
	0429000601	混凝土盾构管片 ϕ6000mm	m	1.0100	1.0100	1.0100	1.0100
	34000011	其他材料费 占材料费	%	1.00	1.00	1.00	1.00
机械	9909000105	门式起重机 50t	台班	0.1890	0.3072	0.2482	0.2858
	9943000207	空压机 $20m^3/min$	台班	1.3000	–	–	–
	99440005	单机离心清水泵 ≤60	台班	1.5750	2.9600	1.2200	2.2900
	9945000006	轴流通风机 7.5kW	台班	1.5400	1.3600	1.3000	1.4500
	9925000202	交流电焊机 32kV·A	台班	0.6600	0.3300	0.3300	0.3300
	99430010	硅整流充电机	台班	–	0.2944	0.1426	0.2530
	9907000402	轨道式电瓶车 45t	台班	–	0.2944	0.1426	0.2530
	9935000101	刀盘式泥水平衡盾构机 ϕ6000	台班	0.2363	0.3840	0.3102	0.3573
	99460004	其他机具费 占人工费	%	2.00	2.00	3.00	2.00

北京市住房和城乡建设委员会

七、$\phi \leqslant 7000$ 泥水平衡式盾构掘进

工作内容：盾构掘进、管片洞内运输、拼装、连接螺栓紧固、装拉杆、施工管线路铺设(照明、运输、供气、通风、通信)、排泥水至沉淀池等。

单位：m

编号				7-35	7-36	7-37	7-38
项目				$\phi \leqslant 7000$ 泥水平衡式盾构掘进			
				负环段	始发段	正常段	到达段
工料机名称			单位	消耗量			
人工	00010504	综合用工二类	工日	28.330	17.284	8.049	13.420
材料	03010551	管片连接螺栓	kg	37.1200	74.2400	74.2400	74.2400
	37010009	轻轨	kg	8.5100	8.5100	8.5100	8.5100
	37050004	钢轨枕	kg	18.6400	18.6400	18.6400	18.6400
	33350011	走道板	kg	20.2700	20.2700	20.2700	20.2700
	03150037	金属支架	kg	14.5800	14.5800	14.5800	14.5800
	12210001	钢管栏杆	kg	8.1500	8.1500	8.1500	8.1500
	8021000803	预拌混凝土 C20	m^3	0.5600	–	–	–
	35030011	钢支撑	kg	209.9000	–	–	–
	22450007	风管	kg	20.4400	20.4400	20.4400	20.4400
	0313010302	电焊条 结 422 $\phi 4$	kg	8.4000	4.2000	4.2000	4.2000
	14090020	油脂	kg	–	31.1900	31.1900	31.1900
	14070003	机油	kg	40.9200	40.3100	19.4800	28.5700
	1703000018	镀锌钢管 *DN*200	m	1.3118	1.3118	1.3118	1.3118
	1703000012	镀锌钢管 *DN*80	m	1.0200	1.0200	1.0200	1.0200
	2811000125	电缆 YC-$3\times35^2+2\times16^2$	m	0.4200	0.4200	0.4200	0.4200
	0429000602	混凝土盾构管片 ϕ7000mm 以内	m	1.0100	1.0100	1.0100	1.0100
	34000011	其他材料费 占材料费	%	1.00	1.00	1.00	1.00
机械	9909000105	门式起重机 50t	台班	0.1890	0.3072	0.2482	0.2858
	9943000207	空压机 $20m^3/min$	台班	1.5400	–	–	–
	99440005	单机离心清水泵 ≤60	台班	1.7550	3.5400	1.4600	2.7300
	9945000006	轴流通风机 7.5kW	台班	1.6900	1.7000	1.4000	1.6300
	9925000202	交流电焊机 32kV·A	台班	0.7000	0.3500	0.3500	0.3500
	99430010	硅整流充电机	台班	–	0.3562	0.1725	0.3061
	9907000403	轨道式电瓶车 50t	台班	–	0.3238	0.1569	0.2783
	9935000102	刀盘式泥水平衡盾构机 ϕ7000	台班	0.2387	0.3878	0.3133	0.3609
	99460004	其他机具费 占人工费	%	2.00	2.00	3.00	2.00

北京市住房和城乡建设委员会

八、$\phi\leqslant8000$ 泥水平衡式盾构掘进

工作内容：盾构掘进、管片洞内运输、拼装、连接螺栓紧固、装拉杆、施工管线路铺设(照明、运输、供气、通风、通信)、排泥水至沉淀池等。

单位：m

编号				7-39	7-40	7-41	7-42
项目				$\phi\leqslant8000$ 泥水平衡式盾构掘进			
				负环段	始发段	正常段	到达段
工料机名称			单位	消耗量			
人工	00010504	综合用工二类	工日	32.489	19.786	9.164	15.343
材料	03010551	管片连接螺栓	kg	42.2400	84.4800	84.4800	84.4800
	37010009	轻轨	kg	8.5100	8.5100	8.5100	8.5100
	37050004	钢轨枕	kg	18.6400	18.6400	18.6400	18.6400
	33350011	走道板	kg	20.2700	20.2700	20.2700	20.2700
	03150037	金属支架	kg	19.0300	19.0300	19.0300	19.0300
	12210001	钢管栏杆	kg	8.1500	8.1500	8.1500	8.1500
	8021000803	预拌混凝土 C20	m^3	0.5600	–	–	–
	35030011	钢支撑	kg	239.9000	–	–	–
	22450007	风管	kg	22.4840	22.4840	22.4840	22.4840
	0313010302	电焊条 结 422 $\phi4$	kg	8.7000	4.3500	4.3500	4.3500
	14090020	油脂	kg	–	36.4611	36.4611	36.4611
	14070003	机油	kg	43.7844	43.1317	20.8436	29.4999
	1703000018	镀锌钢管 *DN*200	m	1.3118	1.3118	1.3118	1.3118
	1703000012	镀锌钢管 *DN*80	m	1.0200	1.0200	1.0200	1.0200
	2811000125	电缆 YC-$3\times35^2+2\times16^2$	m	0.4200	0.4200	0.4200	0.4200
	0429000603	混凝土盾构管片 ϕ8000mm 以内	m	1.0100	1.0100	1.0100	1.0100
	34000011	其他材料费 占材料费	%	1.00	1.00	1.00	1.00
机械	9909000128	门式起重机 60t	台班	0.1645	0.2673	0.2159	0.2487
	9943000207	空压机 $20m^3/min$	台班	1.8172	–	–	–
	99440005	单机离心清水泵 ≤60	台班	1.9305	3.8940	1.6060	3.0030
	9945000006	轴流通风机 7.5kW	台班	1.8590	1.8700	1.5400	1.7930
	9925000202	交流电焊机 32kV·A	台班	0.7250	0.3625	0.3625	0.3625
	99430010	硅整流充电机	台班	–	0.4702	0.2278	0.4041
	9907000404	轨道式电瓶车 60t	台班	–	0.3562	0.1725	0.3061
	9935000103	刀盘式泥水平衡盾构机 ϕ8000	台班	0.2410	0.3917	0.3164	0.3645
	99460004	其他机具费 占人工费	%	2.00	2.00	3.00	2.00

北京市住房和城乡建设委员会

九、$\phi \leqslant 9000$ 泥水平衡式盾构掘进

工作内容：盾构掘进、管片洞内运输、拼装、连接螺栓紧固、装拉杆、施工管线路铺设(照明、运输、供气、通风、通信)、排泥水至沉淀池等。

单位：m

编号				7-43	7-44	7-45	7-46
项目				$\phi \leqslant 9000$ 泥水平衡式盾构掘进			
				负环段	始发段	正常段	到达段
工料机名称			单位	消耗量			
人工	00010504	综合用工二类	工日	37.288	22.679	10.464	25.099
材料	03010551	管片连接螺栓	kg	47.3600	94.7200	94.7200	94.7200
	37010009	轻轨	kg	8.5100	8.5100	8.5100	8.5100
	37050004	钢轨枕	kg	18.6400	18.6400	18.6400	18.6400
	33350011	走道板	kg	20.2700	20.2700	20.2700	20.2700
	03150037	金属支架	kg	23.4800	23.4800	23.4800	23.4800
	12210001	钢管栏杆	kg	8.1500	8.1500	8.1500	8.1500
	8021000803	预拌混凝土 C20	m^3	0.5600	–	–	–
	35030011	钢支撑	kg	269.9000	–	–	–
	22450007	风管	kg	24.7324	24.7324	24.7324	24.7324
	0313010302	电焊条 结 422 $\phi 4$	kg	9.0000	4.5000	4.5000	4.5000
	14090020	油脂	kg	–	42.6230	42.6230	42.6230
	14070003	机油	kg	46.8493	46.1509	22.3027	31.5649
	1703000018	镀锌钢管 *DN*200	m	1.3118	1.3118	1.3118	1.3118
	1703000012	镀锌钢管 *DN*80	m	1.0200	1.0200	1.0200	1.0200
	2811000125	电缆 YC−$3\times35^2+2\times16^2$	m	0.4200	0.4200	0.4200	0.4200
	0429000604	混凝土盾构管片 ϕ9000mm 以内	m	1.0100	1.0100	1.0100	1.0100
	34000011	其他材料费 占材料费	%	1.00	1.00	1.00	1.00
机械	9909000128	门式起重机 60t	台班	0.1645	0.2673	0.2159	0.2487
	9943000207	空压机 $20m^3/min$	台班	2.1443	–	–	–
	99440005	单机离心清水泵 ≤60	台班	2.1236	4.2834	1.7666	3.3033
	9945000006	轴流通风机 7.5kW	台班	2.0449	2.0570	1.6940	1.9723
	9925000202	交流电焊机 32kV·A	台班	0.7500	0.3750	0.3750	0.3750
	99430010	硅整流充电机	台班	–	0.6724	0.3257	0.5779
	9907000111	轨道式电瓶车 80t	台班	–	0.3918	0.1898	0.3367
	9935000104	刀盘式泥水平衡盾构机 ϕ9000	台班	0.2435	0.3956	0.3196	0.3681
	99460004	其他机具费 占人工费	%	2.00	2.00	3.00	2.00

北京市住房和城乡建设委员会

十、$\phi \leqslant 11000$ 泥水平衡式盾构掘进

工作内容：盾构掘进、管片洞内运输、拼装、连接螺栓紧固、装拉杆、施工管线路铺设(照明、运输、供气、通风、通信)、排泥水至沉淀池等。

单位：m

编号				7-47	7-48	7-49	7-50
项目				$\phi \leqslant 11000$ 泥水平衡式盾构掘进			
				负环段	始发段	正常段	到达段
工料机名称			单位	消耗量			
人工	00010504	综合用工二类	工日	73.478	33.177	16.853	25.535
材料	37010009	轻轨	kg	21.4600	21.4600	21.4600	21.4600
	37050004	钢轨枕	kg	24.0300	24.0300	24.0300	24.0300
	33350011	走道板	kg	31.0000	31.0000	31.0000	31.0000
	03150037	金属支架	kg	35.7200	35.7200	35.7200	35.7200
	12210001	钢管栏杆	kg	22.1700	22.1700	22.1700	22.1700
	8021000803	预拌混凝土 C20	m^3	0.6600	–	–	–
	35030011	钢支撑	kg	265.3000	–	–	–
	22450007	风管	kg	40.8800	40.8800	40.8800	40.8800
	0313010302	电焊条 结 422 $\phi 4$	kg	9.1650	4.5820	4.5820	4.5820
	14090020	油脂	kg	–	48.9300	48.9300	48.9300
	0301050302	管片连接螺栓 M36	套	29.0000	58.0000	58.0000	58.0000
	14070003	机油	kg	84.2100	66.2800	31.4000	51.2400
	1703000018	镀锌钢管 $DN200$	m	2.6400	2.6400	2.6400	2.6400
	1703000012	镀锌钢管 $DN80$	m	2.0400	2.0400	2.0400	2.0400
	2811000125	电缆 YC−$3\times35^2+2\times16^2$	m	0.4200	0.4200	0.4200	0.4200
	0429000605	混凝土盾构管片 ϕ11000mm 以内	m	1.0100	1.0100	1.0100	1.0100
	34000011	其他材料费 占材料费	%	1.00	1.00	1.00	1.00
机械	9943000207	空压机 $20m^3/min$	台班	2.6800	–	–	–
	99440005	单机离心清水泵 ≤60	台班	3.1350	4.9400	2.0300	3.8200
	9945000006	轴流通风机 7.5kW	台班	2.6800	2.5400	2.4400	2.6200
	9925000202	交流电焊机 32kV·A	台班	0.7640	0.3820	0.3820	0.3820
	99430010	硅整流充电机	台班	–	0.8069	0.3908	0.6934
	9909000106	门式起重机 150t	台班	0.2637	0.4285	0.3461	0.3987
	9907000111	轨道式电瓶车 80t	台班	–	0.4702	0.2278	0.4041
	9935000105	刀盘式泥水平衡盾构机 ϕ11000	台班	0.3139	0.5101	0.4120	0.4746
	99460004	其他机具费 占人工费	%	2.00	2.00	3.00	2.00

北京市住房和城乡建设委员会

第四节 管片密封条与嵌缝

一、管片设置密封条

工作内容:管片吊运、堆放、编号、表面清理、涂刷、粘贴橡胶条、管片边角嵌缝等。 **单位:**环

编号				7-51	7-52	7-53	7-54
项目				管片设置密封条			
				氯丁橡胶条			
				$\phi \leq 7000$mm	$\phi \leq 8000$mm	$\phi \leq 9000$mm	$\phi \leq 11000$mm
工料机名称			单位	消耗量			
人工	00010504	综合用工二类	工日	0.190	0.249	0.294	0.347
材料	02030001	氯丁橡胶条	m	52.0800	68.2248	80.5053	95.2000
	14410065	氯丁粘接剂	kg	2.4400	3.1964	3.7718	5.7500
	13010051	丁醛自粘腻子	kg	2.4400	3.1964	3.7718	5.7500
	13330501	胶粉油毡衬垫	kg	6.8600	7.3825	7.9050	8.9500
	15130304	聚氨酯泡沫塑料	m^3	0.0190	0.0210	0.0230	0.0270
	34000011	其他材料费 占材料费	%	1.00	1.00	1.00	1.00
机械	99460004	其他机具费 占人工费	%	1.00	1.00	1.00	1.00

北京市住房和城乡建设委员会

工作内容：管片吊运、堆放、编号、表面清理、涂刷、粘贴橡胶条、管片边角嵌缝等。　　　　**单位：**环

编号				7-55	7-56	7-57	7-58
项目				管片设置密封条			
				三元乙丙			
				$\phi \leq 7000$mm	$\phi \leq 8000$mm	$\phi \leq 9000$mm	$\phi \leq 11000$mm
工料机名称			单位	消耗量			
人工	00010504	综合用工二类	工日	0.190	0.249	0.294	0.347
材料	02030007	三元乙丙橡胶条	m	52.0800	68.2248	80.5053	95.2000
	14410065	氯丁粘接剂	kg	1.5100	1.9781	2.3342	1.6700
	13010051	丁醛自粘腻子	kg	1.5100	1.9781	2.3342	1.6700
	13330501	胶粉油毡衬垫	kg	2.5100	2.9450	3.3800	4.2500
	15130304	聚氨酯泡沫塑料	m^3	0.0030	0.0040	0.0050	0.0070
	34000011	其他材料费 占材料费	%	1.00	1.00	1.00	1.00
机械	99460004	其他机具费 占人工费	%	1.00	1.00	1.00	1.00

北京市住房和城乡建设委员会

二、管片嵌缝

工作内容：管片嵌缝槽表面处理、配料嵌缝等。　　　　**单位：**环

编号				7-59	7-60	7-61	7-62
项目				管片嵌缝			
				ϕ≤7000mm	ϕ≤8000mm	ϕ≤9000mm	ϕ≤11000mm
工料机名称			单位	消耗量			
人工	00010504	综合用工二类	工日	3.178	4.386	5.570	7.965
材料	33010037	钢制台座	kg	9.1000	10.2200	11.3400	13.5800
	14410053	环氧聚氨脂嵌缝膏	kg	17.7500	24.4175	31.0850	44.4200
	34000011	其他材料费 占材料费	%	1.00	1.00	1.00	1.00
机械	9909000301	龙门式起重机 10t	台班	0.0584	0.0913	0.1241	0.1898
	99460004	其他机具费 占人工费	%	1.00	1.00	1.00	1.00

北京市住房和城乡建设委员会

第五节 衬砌压浆

工作内容：制浆、送浆、盾尾同步(分块)压浆、封堵、清洗等。 **单位：**m^3

编号				7-63	7-64	7-65	7-66
项目				同步压浆		分块压浆	
				预拌注浆料(水泥砂浆)	水泥水玻璃	预拌注浆料(水泥砂浆)	水泥水玻璃
工料机名称			单位	消耗量			
人工	00010504	综合用工二类	工日	1.144	1.366	1.398	1.640
材料	8009000104	预拌盾构注浆料 有砂	m^3	1.0700	–	1.0700	–
	18310031	盖堵 小于 75mm	个	0.0360	0.0360	0.0360	0.0360
	14310009	硅酸钠(水玻璃)	kg	–	235.4000	–	235.4000
	14310008	磷酸氢二钠	kg	–	6.6000	–	6.6000
	1731001907	高压龙皮管 150mm×3mm	根	0.0040	0.0040	0.0040	0.0040
	0401030004-1	水泥 42.5#	kg	–	441.0000	–	441.0000
	34000011	其他材料费 占材料费	%	1.00	1.00	1.00	1.00
机械	9905000008	灰浆搅拌机 200L	台班	0.2100	0.2303	0.8510	0.9332
	99050006	电动灌浆机	台班	–	–	0.5550	0.6086
	9944000501	盾构同步压浆泵 INJECTQ	台班	0.0370	0.0406	–	–
	9909000403	桥式起重机 10t	台班	0.0622	0.1405	0.1037	–
	99460004	其他机具费 占人工费	%	1.00	1.00	1.00	1.00

北京市住房和城乡建设委员会

第六节 柔性接缝

一、临时阶段

工作内容：1.临时防水环板：接缝处淤泥清除、钢板环圈定位、焊接、预留压浆孔等。2.临时止水缝：安装止水带及防水圈、环后安装后堵压、防水材料封堵等。3.洞口钢筋混凝土环圈：配模、立模、拆模、钢筋制作、吊运、安装、绑扎焊接、混凝土浇筑养护等。 **单位：**见表

编号				7-67	7-68	7-69
项目				临时防水环板	临时止水缝	洞口钢筋混凝土环圈
				t	m	m^3
工料机名称			单位	消耗量		
人工	00010504	综合用工二类	工日	9.694	1.206	6.712
材料	0129001115	普通钢板 δ3.5mm~4.0mm	kg	4.7700	-	-
	01010002-1	钢筋 ϕ10 以内	kg	-	-	70.0000
	01010003	钢筋 ϕ10 以外	kg	-	-	180.0000
	35010004-1	组合钢模板	m^2	-	-	3.1000
	01290036	环圈钢板	t	0.3530	-	-
	0401030004-1	水泥 42.5#	kg	-	90.0000	-
	8021000806	预拌混凝土 C30	m^3	-	-	1.0200
	37050008	道木	m^3	0.0700	-	-
	03130101	电焊条（综合）	kg	32.3400	-	3.1400
	03010556	螺栓(带帽)(综合)	kg	4.6600	1.3100	-

北京市住房和城乡建设委员会

续前

编		号		7-67	7-68	7-69
项		目		临时防水环板	临时止水缝	洞口钢筋混凝土环圈
				t	m	m^3
材料	03010341	压浆孔螺丝	个	12.1200	–	–
	02030002	帘布橡胶条	kg	–	4.3300	–
	14290005-1	乙炔气	m^3	1.5700	–	–
	14290003	氧气	m^3	4.7100	–	–
	1441000207	聚氨酯粘合剂	kg	–	19.9800	–
	15130304	聚氨酯泡沫塑料	m^3	–	0.0868	–
	34000011	其他材料费 占材料费	%	1.00	1.00	1.00

北京市住房和城乡建设委员会

机械	9925000202	交流电焊机 32kV·A	台班	7.1500	–	0.6280
	99050006	电动灌浆机	台班	–	0.4700	–
	9909000013	汽车式起重机 10t	台班	–	–	0.1900
	99170005	钢筋调直机	台班	–	–	0.5400
	99170009	钢筋弯曲机 ϕ40 内	台班	–	–	0.8900
	99170007	钢筋切断机	台班	–	–	0.5400
	99210001	木工圆锯机（综合）	台班	–	–	0.0400
	99050003	混凝土振捣器(插入式)	台班	–	–	0.1220
	99460004	其他机具费 占人工费	%	1.00	1.00	1.00

北京市住房和城乡建设委员会

二、正式阶段

工作内容:1.拆除临时钢环板:钢环板切割、吊拆堆放等。2.拆除洞口环管片:拆卸连接螺栓、吊车配合拆除管片、凿除涂料、壁面清洗等。3.安装钢环板:钢环板分块吊装、焊接固定等。4.柔性接缝环:壁内刷涂料、安装内外壁止水带、压乳胶水泥等。 **单位:**见表

编号				7-70	7-71	7-72	7-73
项目				拆除临时钢环板	拆除洞口环管片	安装钢环板	柔性接缝环
				t	m^3	t	m
工料机名称			单位	消耗量			
人工	00010504	综合用工二类	工日	1.127	2.826	11.631	1.644
材料	01000001-1	型钢 综合	kg	1.6200	-	-	-
	01290036	环圈钢板	t	-	-	1.0600	-
	37050008	道木	m^3	0.0500	-	0.0800	-
	03130101	电焊条(综合)	kg	-	-	21.7600	-
	13370008	内防水橡胶止水带	m	-	-	-	1.0500
	02070021	氯丁橡胶	kg	-	-	-	0.4000
	02010005	接皮海绵橡胶板	kg	-	-	-	8.2500
	02050004	水膨胀橡胶圈	个	-	-	128.0000	-
	14290005-1	乙炔气	m^3	3.5700	0.3900	1.6500	-
	14290003	氧气	m^3	10.7000	1.1700	4.9600	-
	13010015	环氧磁漆	kg	-	-	-	0.7400
	13350030	外防水氯丁酚醛胶	kg	-	-	-	10.1600
	13050042	焦油聚氨酯涂料	kg	-	-	-	2.5200
	15130107	聚苯乙烯硬泡沫塑料	m^3	-	-	-	0.0600
	80010016	乳胶水泥	kg	-	-	-	18.1200
	34000011	其他材料费 占材料费	%	1.00	1.00	1.00	1.00
机械	9943000007	电动空气压缩机 $10m^3/min$	台班	-	0.2300	-	-
	99330001	风镐	台班	-	0.6900	-	-
	99230024	割炬	台班	0.9650	-	-	-
	9925000202	交流电焊机 32kV·A	台班	-	-	1.8100	-
	99050006	电动灌浆机	台班	-	-	-	1.0400
	9909000403	桥式起重机 10t	台班	0.1593	0.1420	1.5399	0.5546
	99460004	其他机具费 占人工费	%	1.00	1.00	1.00	1.00

第七节 负环段管片与隧道内管线拆除

工作内容:拆除钢支撑、清除洞内污垢杂物、拆除井内轨道、清除井内污泥、凿除后背混凝土、切割连接螺栓、管片吊出井口、地面堆放等。

单位:m

编号				7-74	7-75	7-76	7-77
项目				负环段管片拆除			
				$\phi\leq$7000mm	$\phi\leq$8000mm	$\phi\leq$9000mm	$\phi\leq$11000mm
工料机名称			单位	消耗量			
人工	00010504	综合用工二类	工日	18.668	21.598	24.528	25.700
材料	35030011	钢支撑	kg	5.9000	8.4075	10.9150	15.9300
	03130101	电焊条(综合)	kg	3.8900	5.5430	7.1960	10.5030
	14290005-1	乙炔气	m^3	0.9500	1.3537	1.7574	2.5650
	14290003	氧气	m^3	2.8600	4.0755	5.2910	7.7220
	34000011	其他材料费 占材料费	%	1.00	1.00	1.00	1.00
机械	9943000007	电动空气压缩机 10m^3/min	台班	1.2600	1.7955	2.3310	3.4020
	99330001	风镐	台班	2.5200	3.5910	4.6620	6.8040
	9925000202	交流电焊机 32kV·A	台班	0.3240	0.4620	0.6000	0.8750
	9909000004	履带式起重机 15t	台班	1.7400	2.4795	3.2190	4.6980
	99460004	其他机具费 占人工费	%	1.00	1.00	1.00	1.00

北京市住房和城乡建设委员会

工作内容：隧道内水管、风管、走道板、栏杆、钢轨、轨枕、各种施工支架拆除、吊运出井口、装车或堆放、隧道内淤泥清除等。 **单位**：m

编号				7-78	7-79	7-80	7-81
项目				隧道内管线路拆除			
				$\phi\leqslant$7000mm	$\phi\leqslant$8000mm	$\phi\leqslant$9000mm	$\phi\leqslant$11000mm
工料机名称			单位	消耗量			
人工	00010504	综合用工二类	工日	0.721	0.897	1.073	1.142
材料	14290005-1	乙炔气	m^3	0.0280	0.0375	0.0470	0.0660
	14290003	氧气	m^3	0.0830	0.1115	0.1400	0.1970
	34000011	其他材料费 占材料费	%	1.00	1.00	1.00	1.00
机械	9909000403	桥式起重机 10t	台班	0.0252	0.0289	0.0326	0.0400
	99310001	电动三轮车	台班	0.1420	0.1628	0.1836	0.2250
	99440006	电动多级离心清水泵 H<12	台班	0.1900	0.2108	0.2316	0.3000
	99460004	其他机具费 占人工费	%	1.00	1.00	1.00	1.00

北京市住房和城乡建设委员会

第八节 盾构其他工程

一、盾构基座

工作内容：型钢切割、焊接、刷漆、吊运安装等。　　**单位：**t

编号				7-82
项目				盾构基座制作安装
工料机名称			单位	消耗量
人工	00010504	综合用工二类	工日	7.569
材料	01000001-1	型钢 综合	kg	1041.0000
	0129001115	普通钢板 δ3.5mm～4.0mm	kg	19.0000
	33010037	钢制台座	kg	0.0949
	03130101	电焊条（综合）	kg	14.1100
	13050004	防锈漆	kg	17.0000
	14290005-1	乙炔气	m^3	1.6000
	14290003	氧气	m^3	4.8000
	34000011	其他材料费 占材料费	%	1.00
机械	9925000202	交流电焊机 32kV·A	台班	1.7560
	9909000004	履带式起重机 15t	台班	1.2400
	99460004	其他机具费 占人工费	%	1.00

北京市住房和城乡建设委员会

二、手孔封堵

工作内容：手孔清洗、封堵、抹平等。 **单位：**个

编号				7-83	7-84	7-85
项目				手孔封堵		
				ϕ≤7000mm	ϕ≤9000mm	ϕ≤11000mm
工料机名称			单位	消耗量		
人工	00010504	综合用工二类	工日	0.054	0.077	0.081
材料	33010028	钢平台	t	0.0002	0.0002	0.0002
	8001000003	水泥砂浆 1∶2.5	m^3	0.0125	0.0188	0.0209
	14350016	外掺剂	kg	1.5000	2.2500	2.5000
	29060603	塑护套	个	1.0100	1.0100	1.0100
	34000011	其他材料费 占材料费	%	1.00	1.00	1.00
机械	9909000403	桥式起重机 10t	台班	0.0033	0.0042	0.0050
	99310001	电动三轮车	台班	0.0183	0.0229	0.0275
	9909000301	龙门式起重机 10t	台班	0.0267	0.0334	0.0402
	99460004	其他机具费 占人工费	%	1.00	1.00	1.00

北京市住房和城乡建设委员会

三、密封舱添加材料

工作内容：材料运输、拌合、盾构密封舱中添加泡沫和膨润土、对土壤进行改良等。 **单位：**m

编号				7-86	7-87	7-88	7-89
项目				密封舱添加材料			
				$\phi \leq 7000$mm	$\phi \leq 8000$mm	$\phi \leq 9000$mm	$\phi \leq 11000$mm
工料机名称			单位	消耗量			
人工	00010504	综合用工二类	工日	1.164	2.029	2.893	3.239
材料	14350017	泡沫剂	升	90.0000	125.4269	169.3792	270.0000
	04090007	膨润土	kg	180.0000	234.0000	295.2000	441.0000
	34000011	其他材料费 占材料费	%	1.00	1.00	1.00	1.00
机械	99440008	高压注浆泵	台班	0.0500	0.0697	0.0941	0.1390
	99310001	电动三轮车	台班	0.0300	0.0418	0.0564	0.0834
	99460004	其他机具费 占人工费	%	1.00	1.00	1.00	1.00

北京市住房和城乡建设委员会

四、盾构过站、盾构过工作井、盾构平移和盾构掉头

工作内容：托架制作安装、铺设钢轨、千斤顶等设备就位、顶进牵引盾构设备、抬升放低、横移、连接管线、调试及拆除等。 **单位：**台·次

编号				7-90	7-91	7-92	7-93
项目				盾构过站			
				$\phi \leq 7000$mm	$\phi \leq 8000$mm	$\phi \leq 9000$mm	$\phi \leq 11000$mm
工料机名称			单位	消耗量			
人工	00010504	综合用工二类	工日	510.000	616.250	722.500	765.000
材料	37010009	轻轨	kg	1893.0000	2129.6250	2366.2500	2839.5000
	01290034	钢板(中厚)	kg	1.4000	1.5750	1.7500	2.1000
	01000001-1	型钢 综合	kg	1029.4250	1158.1150	1286.8050	1544.1800
	01050001	钢丝绳	kg	590.0000	663.7500	737.5000	885.0000
	37050005	枕木	m^3	12.2000	13.7250	15.2500	18.3000
	03130101	电焊条（综合）	kg	18.0000	20.2500	22.5000	27.0000
	14290005-1	乙炔气	m^3	10.5780	11.8980	13.2180	15.8670
	14290003	氧气	m^3	31.7340	35.7010	39.6680	47.6010
	34000011	其他材料费 占材料费	%	1.00	1.00	1.00	1.00
机械	9935000203	刀盘式土压平衡盾构机 ϕ6000	台班	10.0000	–	–	–
	9935000205	刀盘式土压平衡盾构机 ϕ8000	台班	–	10.0000	–	–
	9935000206	刀盘式土压平衡盾构机 ϕ9000	台班	–	–	10.0000	–
	9935000207	刀盘式土压平衡盾构机 ϕ11000	台班	–	–	–	10.0000
	9909000403	桥式起重机 10t	台班	20.5420	23.1098	25.6775	30.8130
	9943000013	液压千斤顶 200t	台班	84.0000	94.5000	105.0000	126.0000
	99090007	门式起重机 50t 以内	台班	31.5288	35.4699	39.4110	47.2930
	9925000203	交流电焊机 40kV·A	台班	1.5000	1.6875	1.8750	2.2500
	99230023	半自动割刀	台班	90.0000	101.2500	112.5000	135.0000
	99460004	其他机具费 占人工费	%	1.00	1.00	1.00	1.00

北京市住房和城乡建设委员会

工作内容：铺设基座、托架制作安装、铺设钢轨、负环管片拼装、负环管片拆除、吊出井口至地面，基座拆除等。 **单位：**台·次

编号				7-94	7-95	7-96	7-97
项目				过工作井			
				$\phi\leq$7000mm	$\phi\leq$8000mm	$\phi\leq$9000mm	$\phi\leq$11000mm
工料机名称			单位	消耗量			
人工	00010504	综合用工二类	工日	153.719	206.313	209.616	314.804
材料	37010009	轻轨	kg	273.0000	292.5000	312.0000	351.0000
	01290034	钢板(中厚)	kg	245.0000	245.0000	245.0000	245.0000
	01000001-1	型钢 综合	kg	530.0000	620.0000	710.0000	890.0000
	01050001	钢丝绳	kg	77.4000	82.9500	88.5000	99.6000
	37050005	枕木	m^3	1.6500	1.7400	1.8300	2.0100
	03130101	电焊条（综合）	kg	9.9000	11.7000	13.5000	17.1000
	14290005-1	乙炔气	m^3	1.3880	1.4875	1.5870	1.7860
	14290003	氧气	m^3	4.1600	4.4600	4.7600	5.3600
	34000011	其他材料费 占材料费	%	1.00	1.00	1.00	1.00
机械	9935000203	刀盘式土压平衡盾构机 ϕ6000	台班	2.0000	–	–	–
	9935000205	刀盘式土压平衡盾构机 ϕ8000	台班	–	2.0000	–	–
	9935000206	刀盘式土压平衡盾构机 ϕ9000	台班	–	–	2.0000	–
	9935000207	刀盘式土压平衡盾构机 ϕ11000	台班	–	–	–	2.0000
	9909000403	桥式起重机 10t	台班	5.2200	5.5680	5.9160	6.6120
	9943000013	液压千斤顶 200t	台班	21.4000	22.7960	24.1920	26.9840
	99090007	门式起重机 50t 以内	台班	7.9500	8.5150	9.0800	10.2100
	9925000203	交流电焊机 40kV·A	台班	0.1160	0.9750	1.1250	1.4250
	99230023	半自动割刀	台班	22.9000	24.4100	25.9200	28.9400
	99460004	其他机具费 占人工费	%	1.00	1.00	1.00	1.00

北京市住房和城乡建设委员会

工作内容:布设预埋铁件,铺设钢轨,连接管线,吊装设施,铺设车架轨道,盾构机抬高,盾构牵引,放低,横移等。 **单位**:台·次

编号				7-98	7-99	7-100	7-101
项目				盾构平移			
				$\phi \leqslant 7000$mm	$\phi \leqslant 8000$mm	$\phi \leqslant 9000$mm	$\phi \leqslant 11000$mm
工料机名称			单位	消耗量			
人工	00010504	综合用工二类	工日	400.387	473.177	545.951	691.557
材料	37010009	轻轨	kg	15926.0000	26898.6164	39184.2541	55885.1859
	01290034	钢板(中厚)	kg	43380.0000	43380.0000	43380.0000	43380.0000
	01000001-1	型钢 综合	kg	5286.0000	6183.5628	7081.4161	8876.5551
	01050001	钢丝绳	kg	1180.0000	1264.6060	1349.2081	1518.3988
	3301003012	钢制滚筒	t	2.4500	2.4500	2.4500	2.4500
	03130101	电焊条(综合)	kg	1007.6000	1079.5426	1151.5481	1295.4916
	14290003	氧气	m^3	311.6000	334.0664	356.5491	401.5099
	14290005-1	乙炔气	m^3	144.3040	154.6506	164.9967	185.6873
	3301003011	钢制转向基座	t	3.6800	3.9428	4.2058	4.7315
	03010556	螺栓(带帽)(综合)	kg	6.0000	6.4284	6.8572	7.7144
	35030011	钢支撑	kg	6.8000	7.2855	7.7714	8.7428
	33350011	走道板	kg	26.9000	26.9000	26.9000	26.9000
	34000011	其他材料费 占材料费	%	1.00	1.00	1.00	1.00
机械	9907000302	轨道平车 10t	台班	19.2000	20.4518	21.7035	24.2081
	9907000401	轨道式电瓶车 25t	台班	9.6000	10.2259	10.8517	12.1040
	9943000013	液压千斤顶 200t	台班	192.0000	204.5184	217.0349	242.0807
	99090017	手扳葫芦	台班	480.0000	511.2960	542.5873	605.2019
	9925000202	交流电焊机 32kV·A	台班	83.9700	89.9600	95.9600	107.9600
	9925000203	交流电焊机 40kV·A	台班	96.0000	102.2592	108.5175	121.0404
	9944001402	污水泵 ϕ150mm	台班	54.0000	57.5208	61.0411	68.0852
	9943001001	分离式油压千斤顶 10t	台班	96.0000	102.2592	108.5175	121.0404
	9943000602	液压千斤顶 100t	台班	192.0000	204.5184	217.0349	242.0807
	9943001002	分离式油压千斤顶 50t	台班	96.0000	102.2592	108.5175	121.0404
	9909000005	履带式起重机 25t	台班	14.4000	15.3389	16.2776	18.1560
	99460004	其他机具费 占人工费	%	1.00	1.00	1.00	1.00

北京市住房和城乡建设委员会

工作内容：站台布设预埋铁件，铺设钢轨，连接管线，吊装设施，铺设车架轨道，盾构机抬高，盾构牵引，放低，横移等。 **单位：**台·次

编号				7-102	7-103	7-104	7-105
项目				盾构调头			
				$\phi \leq$7000mm	$\phi \leq$8000mm	$\phi \leq$9000mm	$\phi \leq$11000mm
工料机名称			单位	消耗量			
人工	00010504	综合用工二类	工日	621.376	734.342	847.284	1073.255
材料	37010009	轻轨	kg	1147.0000	1228.8958	1310.8631	1474.7210
	01290034	钢板(中厚)	kg	30966.0000	30966.0000	30966.0000	30966.0000
	01000001-1	型钢 综合	kg	4020.0000	4702.5960	5385.4129	6750.6151
	01090010	圆钢 ϕ10 以外	kg	1374.0000	1607.3052	1840.6859	2307.2998
	3301003012	钢制滚筒	t	2.4500	2.4500	2.4500	2.4500
	03130101	电焊条（综合）	kg	256.3200	274.6212	292.9384	329.5557
	14290003	氧气	m^3	180.9000	193.9429	206.9953	233.0974
	14290005-1	乙炔气	m^3	59.1000	63.3375	67.5748	76.0487
	0403000003-2	砂子 中粗砂	kg	96.0000	96.0000	96.0000	96.0000
	3301003011	钢制转向基座	t	3.6800	3.9428	4.2058	4.7315
	13050004	防锈漆	kg	102.0000	109.2828	116.5720	131.1435
	37050005	枕木	m^3	0.0120	0.0140	0.0160	0.0201
	34000011	其他材料费 占材料费	%	1.00	1.00	1.00	1.00
机械	9907000002	载重汽车 4t	台班	0.0180	0.0213	0.0246	0.0312
	9909000003	履带式起重机 10t	台班	7.9240	9.3646	10.8049	13.6866
	9909000005	履带式起重机 25t	台班	5.7980	6.8521	7.9060	10.0145
	9909000032	履带式起重机 60t	台班	6.0000	7.0908	8.1814	10.3634
	9909000009	履带式起重机 300t	台班	6.0000	7.0908	8.1814	10.3634
	9909000403	桥式起重机 10t	台班	1.0760	1.2716	1.4672	1.8585
	99170008	钢筋切断机 ϕ40 以内	台班	1.2000	1.4182	1.6363	2.0727
	9919000011	摇臂钻床 ϕ63m	台班	14.8800	17.5852	20.2898	25.7011
	99250008	电弧焊机	台班	46.0736	54.4498	62.8241	79.5793
	99460004	其他机具费 占人工费	%	1.00	1.00	1.00	1.00

北京市住房和城乡建设委员会

第八章　防水工程

北京市住房和城乡建设委员会

北京市住房和城乡建设委员会

说　　明

一、本章包括:桥面防水层与泄水管,明挖结构防水,盖挖与暗挖结构防水,施工缝与变形缝 4 节共 33 个子目。

二、防水保护层按 50mm 厚编制,设计厚度不同时按比例换算。

三、防水主材消耗量已包含搭接用量,但不包含附加层用量。

四、设计泄水管材质与本标准不同时,可调整。

工程量计算规则

一、桥面防水层与泄水管

1.涂沥青、油毡、防水砂浆、防水橡胶板、聚氨酯防水涂料均按设计图示尺寸以面积计算。

2.聚丙烯纤维网混凝土保护层按设计图示尺寸以体积计算。

3.泄水管按设计图示尺寸以长度计算。

二、明挖结构防水:明挖防水找平层、防水保护层、防水卷材、防水毯、防水涂料均按设计图示以面积计算,不扣除单孔面积≤0.3 ㎡的孔洞所占面积。

三、盖挖与暗挖防水:土工布、防水卷材、防水板橡胶沥青涂层均按设计图示尺寸以面积计算,不扣除单孔面积≤0.3 ㎡的孔洞所占面积。

四、施工缝与变形缝:止水带、遇水膨胀止水条、遇水膨胀止水胶、不锈钢接水盒均按设计图示尺寸以长度计算。

北京市住房和城乡建设委员会

北京市住房和城乡建设委员会

第一节 桥面防水层与泄水管

一、桥面防水层

工作内容：洒沥青、铺油毡、浇筑防水砂浆或防水橡胶板铺设、固定、检查等。　　**单位：**m^2

编号				8-1	8-2	8-3	8-4
项目				桥面防水层			
				涂沥青	一层油毡	防水砂浆 20mm	防水橡胶板 2mm
工料机名称			单位	消耗量			
人工	00010501	综合用工二类	工日	0.142	0.016	0.029	0.104
材料	13050049	煤焦沥青漆	kg	0.0367	-	-	-
	34110005-2	煤	t	0.0043	-	-	-
	34110003	木柴	kg	1.0500	-	-	-
	13390012	油毡	m^2	-	1.2200	-	-
	14350003	防水剂	kg	-	-	0.5610	-
	80010021	干混地面砂浆	m^3	-	-	0.0210	-
	02010002	橡胶板 δ1mm~3mm	kg	-	-	-	10.2000
	14410063	氯丁胶沥青胶剂	kg	-	-	-	12.0000
	34000011	其他材料费 占材料费	%	1.00	1.00	1.00	1.00
机械	99460004	其他机具费 占人工费	%	1.50	1.50	1.50	1.50

北京市住房和城乡建设委员会

工作内容：1.聚氨酯防水涂料：清理面层、运料、喷涂防水涂料、抹平、养护等。
2.聚丙烯纤维网混凝土保护层：模板制作、安装、拆除、混凝土浇筑、捣固、养护等。

单位：见表

编号				8-5	8-6
项目				桥面防水层	
				聚氨酯防水涂料	聚丙烯纤维网混凝土保护层
				1.5mm 厚	
				m^2	m^3
工料机名称			单位	消耗量	
人工	00010501	综合用工二类	工日	0.056	2.020
材料	13050043	聚氨酯防水涂料	kg	1.8900	-
	8021000808	预拌混凝土 C40	m^3	-	1.0150
	03150516	聚丙烯纤维网	kg	-	1.8000
	05030007	板方材	m^3	-	0.0020
	14350050	模板嵌缝料	kg	-	0.0080
	14350009	脱模剂	kg	-	0.0150
	34000011	其他材料费 占材料费	%	1.00	1.00
机械	99130008	柏油喷布器	台班	0.0030	-
	99460004	其他机具费 占人工费	%	1.50	1.50

北京市住房和城乡建设委员会

二、泄水管安装

工作内容：划线、截料、打眼钻孔、安装管箍、接口涂料等。　　　　单位：m

编号				8-7	8-8	8-9
项目				泄水管安装		
				钢管	铸铁管	塑料管
工料机名称			单位	消耗量		
人工	00010501	综合用工二类	工日	0.047	0.057	0.046
材料	1701000012-1	焊接钢管 *DN*150	m	1.0200	-	-
	1711001306	下水铸铁管 200	m	-	1.0200	-
	1725011410	硬塑料管 100	m	-	-	1.0200
	13310003	石油沥青	kg	2.6490	2.6490	-
	34000011	其他材料费 占材料费	%	1.00	1.00	1.00
机械	99460004	其他机具费 占人工费	%	1.50	1.50	1.50

北京市住房和城乡建设委员会

第二节 明挖结构防水

工作内容：1.找平层和保护层：基层处理、混凝土搅拌、输送、浇筑、养护等。2.SBS 改性沥青防水卷材：刷冷底子油、卷材铺设、搭接、收边、检查验收等。

单位：m^2

编号				8-10	8-11	8-12	8-13
项目				防水找平层	防水保护层	SBS 改性沥青防水卷材	
					50mm 厚	单层	每增一层
工料机名称			单位	消耗量			
人工	00010501	综合用工二类	工日	0.066	0.067	0.108	0.081
材料	8021000703	预拌豆石混凝土 C20	m^3	–	0.0510	–	–
	8001000001	水泥砂浆 1 :1	m^3	0.0204	–	–	–
	1333030206	SBS 改性沥青油毡防水卷材 4mm	m^2	–	–	1.2000	1.2000
	13310010	水乳型橡胶沥青	kg	–	–	0.3000	–
	14350048	冷底子油	kg	–	–	0.1000	–
	34000011	其他材料费 占材料费	%	1.00	1.00	1.00	1.00
机械	9909000301	龙门式起重机 10t	台班	0.0100	0.0100	0.0050	0.0050
	99460004	其他机具费 占人工费	%	1.50	1.50	1.50	1.50

北京市住房和城乡建设委员会

工作内容：基层表面清理、修整、节点处理、定位、弹线、试铺、铺贴卷材、收头、节点密封、清理、检修、修整等。 **单位：**m^2

编号				8-14	8-15
项目				高分子自粘胶膜防水卷材	
				顶板(底板)	侧墙
工料机名称			单位	消耗量	
人工	00010501	综合用工二类	工日	0.084	0.104
材料	1333090307	高分子自粘胶膜防水卷材 1.5mm	m^2	1.2420	1.2420
	34000011	其他材料费 占材料费	%	1.00	1.00
机械	9909000301	龙门式起重机 10t	台班	0.0050	0.0050
	99460004	其他机具费 占人工费	%	1.50	1.50

北京市住房和城乡建设委员会

工作内容：1.膨润土防水毯：基层表面清理、修整、喷涂基层处理剂、定位、弹线、试铺、铺贴卷材、收头、节点密封、清理、检修、修整等。2.防水涂料：清理基层、刷基层处理剂、刷防水涂料等。

单位：m^2

编号				8-16	8-17	8-18
项目				膨润土防水毯	聚氨酯防水涂料 2.5mm厚	水泥基渗透结晶型防水涂料
工料机名称			单位	消耗量		
人工	00010501	综合用工二类	工日	0.118	0.074	0.090
材料	03150103	射钉	个	4.5000	–	–
	20330001	垫片	个	4.5000	–	–
	13390008	膨润土防水毯	m^2	1.2420	–	–
	13050043	聚氨酯防水涂料	kg	–	3.1500	–
	13050051	塞柏斯防水涂料	kg	–	–	1.8000
	34000011	其他材料费 占材料费	%	1.00	1.00	1.00
机械	9909000301	龙门式起重机 10t	台班	0.0050	0.0050	0.0050
	99460004	其他机具费 占人工费	%	1.50	1.50	1.50

北京市住房和城乡建设委员会

第三节 暗挖与盖挖结构防水

工作内容：1.土工布：垂直及洞内运输、清理整平、挖填锚固沟、铺设土工布、缝合及锚固土工布等。2.EVA 防水卷材、ECB 防水板：垂直及洞内运输、基层表面清理、修整、喷涂基层处理剂、节点附加增强处理、定位、弹线、试铺、铺贴卷材、收头、节点密封、清理、检修、修整等。

单位：m^2

编号				8-19	8-20	8-21	8-22
项目				土工布		EVA 防水卷材	ECB 防水板
				单层	每增一层		
工料机名称			单位	消耗量			
人工	00010504	综合用工二类	工日	0.078	0.084	0.170	0.171
材料	02310010	无纺布	m^2	1.2450	1.2420	–	–
	03150103	射钉	个	4.5000	–	6.0000	6.0000
	20330001	垫片	个	4.5000	–	5.0000	5.0000
	0211000102	塑料防水板 2.0mmECB	m^2	–	–	–	1.2420
	0211000101	塑料防水板 1.5mmEVA	m^2	–	–	1.2420	–
	03130108	塑料焊条	kg	–	–	–	0.6000
	34000011	其他材料费 占材料费	%	1.00	1.00	1.00	1.00
机械	9909000403	桥式起重机 10t	台班	0.0006	0.0006	0.0006	0.0006
	99250013	热缝焊机	台班	–	–	0.0200	0.0500
	99310001	电动三轮车	台班	0.0120	0.0120	0.0200	0.0200
	99460004	其他机具费 占人工费	%	1.50	1.50	1.50	1.50

北京市住房和城乡建设委员会

工作内容：基层表面清理、修整、节点处理、定位、弹线、试铺、铺贴卷材、收头、节点密封、清理、检修、修整等。　　**单位：**m^2

编号				8-23	8-24	8-25
项目				高分子自粘胶膜防水卷材		
				底板	侧板	顶板
工料机名称			单位	消耗量		
人工	00010504	综合用工二类	工日	0.122	0.150	0.158
材料	1333090307	高分子自粘胶膜防水卷材 1.5mm	m^2	1.2420	1.2420	1.2420
	34000011	其他材料费 占材料费	%	1.00	1.00	1.00
机械	9909000403	桥式起重机 10t	台班	0.0006	0.0006	0.0006
	99310001	电动三轮车	台班	0.0200	0.0200	0.0200
	99460004	其他机具费 占人工费	%	1.50	1.50	1.50

北京市住房和城乡建设委员会

工作内容：清理基层、刷基层处理剂、刷防水涂料等。 **单位：**m^2

编号				8-26	8-27
项目				橡胶沥青涂层	
				2mm 厚	每增减 0.5mm
工料机名称			单位	消耗量	
人工	00010504	综合用工二类	工日	0.034	0.012
材料	13050017	速凝橡胶沥青甲组份	kg	3.7500	0.9375
	13050018	速凝橡胶沥青乙组份	kg	0.7500	0.1875
	13050019	速凝橡胶沥青单组份	kg	0.3040	0.0760
	34000011	其他材料费 占材料费	%	1.00	1.00
机械	99450006	双组份涂料喷涂机	台班	0.0070	0.0018
	99460004	其他机具费 占人工费	%	1.50	1.50

北京市住房和城乡建设委员会

第四节 施工缝与变形缝

工作内容：垂直及洞内运输、混凝土表面清理、止水带安装、嵌缝密封等。 **单位：**m

编号				8-28	8-29	8-30
项目				止水带		
				中埋式钢边橡胶	外贴式橡胶	中埋式注浆
工料机名称			单位	消耗量		
人工	00010501	综合用工二类	工日	0.116	0.078	0.116
材料	13370013	中埋式钢边橡胶止水带	m	1.0500	-	-
	1337000601	橡胶止水带 市政	m	-	1.0500	-
	13370003	注浆止水带	m	-	-	1.0500
	1731001906	注浆导管 ϕ12	m	-	-	0.1365
	34000011	其他材料费 占材料费	%	1.00	1.00	1.00
机械	9909000403	桥式起重机 10t	台班	0.0004	0.0004	0.0004
	99310001	电动三轮车	台班	0.0020	0.0020	0.0020
	99460004	其他机具费 占人工费	%	1.50	1.50	1.50

北京市住房和城乡建设委员会

工作内容：1.止水条：垂直及洞内运输、混凝土表面清理、防水胶条安装、嵌缝密封等。2.止水胶：垂直及洞内运输、混凝土表面清理、刷止水胶、嵌缝密封等。3.不锈钢接水盒：垂直及洞内运输、清缝、涂抹聚硫密封胶、接水盒安装、固定、密封处理等。 **单位：**m

编号				8-31	8-32	8-33
项目				遇水膨胀止水条	遇水膨胀止水胶(一道)	不锈钢接水盒
工料机名称			单位	消耗量		
人工	00010501	综合用工二类	工日	0.066	0.098	0.173
材料	13370004	遇水膨胀止水条	m	1.0500	–	–
	13350032	遇水膨胀止水胶	升	–	0.2010	–
	3407000201	不锈钢接水盒 1mm	m	–	–	1.0200
	03150122	水泥钉	kg	–	–	0.2000
	14410031	嵌缝胶	升	–	–	0.1008
	34000011	其他材料费 占材料费	%	1.00	1.00	1.00
机械	9909000403	桥式起重机 10t	台班	0.0004	0.0004	0.0008
	99310001	电动三轮车	台班	0.0020	0.0020	0.0040
	99460004	其他机具费 占人工费	%	1.50	1.50	1.50

北京市住房和城乡建设委员会

北京市住房和城乡建设委员会

第九章　模板工程

北京市住房和城乡建设委员会

北京市住房和城乡建设委员会

说　　明

一、本章包括：地上结构模板，桥梁模板，明挖车站模板，明挖区间模板，暗挖车站模板，盖挖车站模板，暗挖区间模板，竖井与其他模板8节共78个子目。

二、高架结构模板中现浇梁(板)的模板需另执行支架子目，其余子目均已包含支架消耗量。

三、明挖车站、暗挖车站和盖挖车站的柱、梁、墙、板的支模高度按6m以内编制，支模高度指板的上表面至上一层楼板的下表面之间的高度。超过6m的部分，执行模板支撑高度6m以上每增1m相应子目。

四、模板台车按隧道二衬结构内宽7m以内编制，超过7m的按实际方案计算。

五、盖挖车站顶板和中板按地模编制，盖挖车站其他部位模板执行暗挖车站模板相应子目。

六、模板支架均不包括支架底座(垫木)以下的基础和地基处理等工作，发生时另行计算。

工程量计算规则

一、混凝土结构(楼梯除外)模板工程量均按模板与混凝土的接触面积计算。

二、桥梁支架按设计梁板外缘宽度乘以长度以面积计算；满堂式支架按梁板下支架体积算。

三、柱

1.柱模板及支架按柱周长乘以柱高计算，不扣除柱与梁连接重叠部分的面积。牛腿的模板面积并入柱模板工程量中。

2.柱高从柱基或板上表面算至上一层楼板上表面，无梁板算至柱帽底部标高。

四、梁

北京市住房和城乡建设委员会

1.梁模板及支架按展开面积计算，不扣除梁与梁连接重叠部分的面积。梁侧面按展开面积并入梁模板工程量中。

2.梁长的计算规定：

(1)梁与柱连接时，梁长算至柱侧面。

(2)主梁与次梁连接时，次梁长算至主梁侧面。

(3)梁与墙连接时，梁长算至墙侧面。如墙为砌块(砖)墙时，嵌入墙内的梁头和梁垫的面积并入梁的工程量中。

3.过梁按图示尺寸以面积计算。

五、墙

墙模板及支架按模板与现浇混凝土构件的接触面积计算，附墙柱侧面积并入墙模板工程量。单孔面积≤$0.3m^2$ 的孔洞不予扣除，洞侧壁模板亦不增加；单孔面积>$0.3m^2$ 的孔洞应予扣除，洞侧壁模板面积并入墙模板工程量中。

1.墙模板及支架按墙图示长度乘以墙高计算，外墙高度由楼板表面算至上一层楼板上表面，内墙高度由楼板上表面算至上一层楼板(或梁)下表面。

2.暗梁、暗柱模板不单独计算。

3.采用定型大钢模板时，洞口面积不予扣除，洞侧壁模板亦不增加。

六、板

板模板及支架按模板与现浇混凝土构件的接触面积计算，单孔面积≤$0.3m^2$ 的孔洞不予扣除，洞侧壁模板亦不增加；单孔面积>$0.3m^2$ 的孔洞应予扣除，洞侧壁模板面积并入板模板工程量中。对于预留的出土孔等后期需二次施工的孔洞，该孔洞按模板与现浇混凝土构件的接触面积计算两次。

1.梁所占面积应予扣除。

2.有梁板按板与次梁的模板面积之和计算。

北京市住房和城乡建设委员会

3.柱帽按展开面积计算,并入无梁板工程量中。

七、倒角模板并入板模板计算。

八、模板支撑高度大于6m时,按超过部分全部面积计算工程量。

九、楼梯(包括休息平台、平台梁、斜梁和楼层板的连接梁)按水平投影面积计算,不扣除宽度≤500mm的楼梯井所占面积,楼梯踏步、踏步板、平台梁等侧面模板面积不另行计算,伸入墙内部分亦不增加。

十、其他混凝土结构模板均按设计图示接触面积计算。

十一、暗挖模板台车吊装吊拆按实际方案以台次计算。

北京市住房和城乡建设委员会

北京市住房和城乡建设委员会

第一节 地上结构模板

工作内容：模板及支架场内运输、安装、涂脱模剂、拆除、整理堆放等。 **单位：**m^2

编号				9-1	9-2	9-3	9-4
项目				导墙	垫层	挡土墙	压顶
工料机名称			单位	消耗量			
人工	00010301	综合用工一类	工日	0.304	0.117	0.493	0.220
材料	05030007	板方材	m^3	0.0010	0.0076	0.0030	0.0050
	35010003	复合木模板	m^2	0.1736	–	–	0.3800
	14350009	脱模剂	kg	0.1000	0.1000	0.1000	0.1000
	35010004-3	组合钢模板	m^2·日	–	–	5.6490	–
	01010015	钢筋	kg	–	2.5657	–	–
	03150101	铁钉	kg	–	0.0236	–	–
	34000011	其他材料费 占材料费	%	5.00	5.00	5.00	5.00
机械	9909000015	汽车式起重机 16t	台班	0.0150	0.0010	0.0020	0.0010
	99210001	木工圆锯机（综合）	台班	0.0010	0.0010	0.0014	0.0010
	9907000007	载重汽车 15t	台班	0.0024	0.0030	0.0034	0.0320
	99460004	其他机具费 占人工费	%	1.50	1.50	1.50	1.50

北京市住房和城乡建设委员会

第二节 高架结构模板

工作内容：模板及支架场内运输、安装、涂脱模剂、拆除、整理堆放等。　　**单位：**m^2

编号				9-5	9-6	9-7	9-8	9-9	9-10
项目				垫层	基础	承台	整体式墩台	重力式墩台	柱式墩台
工料机名称			单位	消耗量					
人工	00010301	综合用工一类	工日	0.117	0.224	0.288	0.334	0.239	0.303
材料	35010003	复合木模板	m^2	–	0.1230	0.2468	–	–	–
	3509000501	板方材 模板背楞	m^3	0.0076	0.0025	0.0046	0.0116	0.0084	0.0116
	35010017	定型钢模板	m^2·日	–	–	–	7.1000	7.1000	7.1000
	03150101	铁钉	kg	0.0236	0.0279	0.0150	–	–	–
	14350009	脱模剂	kg	0.1000	0.1000	0.1000	0.1000	0.1000	0.1000
	34000011	其他材料费 占材料费	%	5.00	5.00	5.00	5.00	5.00	5.00
机械	9921000001	木工圆锯机 500mm	台班	0.0030	0.0080	0.0080	–	–	–
	9909000015	汽车式起重机 16t	台班	0.0011	0.0020	0.0026	0.0036	0.0036	0.0036
	9907000007	载重汽车 15t	台班	0.0011	0.0020	0.0026	0.0036	0.0036	0.0036
	99460004	其他机具费 占人工费	%	1.50	1.50	1.50	1.50	1.50	1.50

北京市住房和城乡建设委员会

工作内容：模板及支架场内运输、安装、涂脱模剂、拆除、整理堆放等。　　　　**单位：**m^2

编号				9-11	9-12	9-13	9-14	9-15
项目				V 型、Y 型墩	轻型桥台	墩台帽	盖梁	箱梁
工料机名称			单位	消耗量				
人工	00010301	综合用工一类	工日	0.309	0.236	0.331	0.330	0.504
材料	35010003	复合木模板	m^2	–	–	0.3387	0.3387	0.3500
	3509000501	板方材 模板背楞	m^3	0.0116	0.0130	0.0170	0.0192	0.0150
	35010017	定型钢模板	m^2·日	7.1000	7.1000	–	–	–
	03150101	铁钉	kg	–	–	0.2960	0.0910	0.1430
	14350009	脱模剂	kg	0.1000	0.1000	0.1000	0.1000	0.1000
	34000011	其他材料费 占材料费	%	5.00	5.00	5.00	5.00	5.00
机械	9921000001	木工圆锯机 500mm	台班	–	–	0.0170	0.0036	0.0018
	9909000015	汽车式起重机 16t	台班	0.0036	0.0036	0.0020	0.0047	0.0047
	9907000007	载重汽车 15t	台班	0.0036	0.0036	0.0020	0.0047	0.0047
	99460004	其他机具费 占人工费	%	1.50	1.50	1.50	1.50	1.50

北京市住房和城乡建设委员会

工作内容：模板及支架场内运输、安装、涂脱模剂、拆除、整理堆放等。 **单位**：m^2

编		号		9-16
项		目		现浇 0#块混凝土
工料机名称			单位	消耗量
人工	00010301	综合用工一类	工日	1.160
材料	05030007	板方材	m^3	0.0080
	35030011	钢支撑	kg	0.2180
	35010004-3	组合钢模板	m^2·日	20.7700
	03150007	零星卡具	kg	0.4200
	03150141	圆钉	kg	0.1220
	03150906	铁件	kg	0.1480
	14350009	脱模剂	kg	0.1000
	14350050	模板嵌缝料	kg	0.0500
	34000011	其他材料费 占材料费	%	5.00
机械	9909000015	汽车式起重机 16t	台班	0.0047
	9921000001	木工圆锯机 500mm	台班	0.0180
	9907000007	载重汽车 15t	台班	0.0047
	99460004	其他机具费 占人工费	%	1.50

北京市住房和城乡建设委员会

工作内容：模板及支架场内运输、安装、涂脱模剂、拆除、整理堆放等。　　　　**单位**：m^2

编号				9-17	9-18	9-19	9-20	9-21	9-22
项目				矩形梁板		连续实体板	异形连续板	桥头搭板及枕梁	桥梁接头
				实心	空心				
工料机名称			单位	消耗量					
人工	00010301	综合用工一类	工日	0.218	0.278	0.218	0.294	0.200	0.348
材料	3509000501	板方材 模板背楞	m^3	0.0082	0.0082	0.0082	0.0082	0.0081	0.0011
	14350009	脱模剂	kg	0.1000	0.1000	0.1000	0.1000	0.1000	0.1000
	35010003	复合木模板	m^2	0.2468	0.2691	0.2468	0.2691	0.2468	0.1280
	03150101	铁钉	kg	0.0115	0.0204	0.0115	0.0204	0.0279	0.0450
	34000011	其他材料费 占材料费	%	5.00	5.00	5.00	5.00	5.00	5.00
机械	9909000015	汽车式起重机 16t	台班	0.0047	0.0047	0.0047	0.0047	0.0047	0.0047
	9921000001	木工圆锯机 500mm	台班	0.0020	0.0020	0.0020	0.0024	0.0024	0.0030
	9907000007	载重汽车 15t	台班	0.0047	0.0047	0.0047	0.0047	0.0047	0.0047
	99460004	其他机具费 占人工费	%	1.50	1.50	1.50	1.50	1.50	1.50

北京市住房和城乡建设委员会

工作内容：模板及支架场内运输、安装、涂脱模剂、拆除、整理堆放等。 **单位：**m^2

编号				9-23	9-24	9-25
项目				支座垫石	小型构件	挡土墙
工料机名称			单位	消耗量		
人工	00010301	综合用工一类	工日	0.271	0.382	0.279
材料	05030007	板方材	m^3	0.0030	0.0064	0.0109
	0301050401	对拉螺栓 M14	m	0.1350	0.1810	0.0612
	35010004-3	组合钢模板	m^2·日	-	-	5.6490
	14350009	脱模剂	kg	0.1000	0.1000	0.1000
	35010003	复合木模板	m^2	0.3053	0.1369	-
	34000011	其他材料费 占材料费	%	5.00	5.00	5.00
机械	9909000015	汽车式起重机 16t	台班	0.0013	0.0020	0.0028
	99210001	木工圆锯机（综合）	台班	0.0059	0.0630	0.0010
	9907000007	载重汽车 15t	台班	0.0013	0.0029	0.0028
	99460004	其他机具费 占人工费	%	1.50	1.50	1.50

北京市住房和城乡建设委员会

工作内容：立杆、连接、铺板、垫脚、挂安全网、完工后拆架、清场、分类归堆及场内运输等。　　**单位：**见表

编号				9-26	9-27	9-28
项目				桥梁支架		满堂式支架
				高 6m 以内	每增 1m	
				m^2		m^3
工料机名称			单位	消耗量		
人工	00010301	综合用工一类	工日	0.691	0.144	0.161
材料	3509000201	工字钢 20#，租赁	m·日	84.0000	22.0000	10.8800
	3509000202	工字钢 30#，租赁	m·日	17.0000	4.4000	8.8400
	3509000203	工字钢 56#，租赁	m·日	29.0000	7.6310	-
	37090038	强立柱	个·日	35.0000	7.0000	-
	35020003	顶托	个·日	12.0000	-	5.2310
	37090011	底托	个·日	12.0000	-	5.2310
	3503000601	拉杆 ϕ48mm	m·日	456.0000	120.0000	-
	35090025	卡子	个·日	118.0000	31.0520	-
	35020005	对接扣件	个·日	-	-	19.7400
	35030024	支架杆件	m·日	-	-	244.1410
	3509000101	方钢 70mm×50mm×2.5mm	kg·日	-	-	16.4210
	34000011	其他材料费 占材料费	%	5.00	5.00	5.00
机械	9907000016	载重汽车 20t	台班	-	-	0.0021
	9909000017	汽车式起重机 25t	台班	-	-	0.0042
	9907000007	载重汽车 15t	台班	0.0050	0.0017	-
	9909000015	汽车式起重机 16t	台班	0.0130	0.0046	-
	99460004	其他机具费 占人工费	%	1.50	1.50	1.50

北京市住房和城乡建设委员会

第三节 明挖车站模板

工作内容：模板及支架场内运输、刷脱模剂、安装、拆除、修理、清理模板粘结物及模内杂物、整理堆放等。 **单位：**m^2

编号				9-29	9-30	9-31	9-32	9-33
项目				底板	中板、顶板	站台板	内衬墙	侧墙(中隔墙)
工料机名称			单位	消耗量				
人工	00010301	综合用工一类	工日	0.250	0.522	0.340	0.277	0.320
材料	05030007	板方材	m^3	0.0030	0.0125	0.0250	–	0.0030
	35010003	复合木模板	m^2	0.3463	0.5100	0.3463	–	0.3463
	35090024	钢管	m·日	–	100.0000	60.1782	–	–
	14350009	脱模剂	kg	0.1000	0.1000	0.1000	0.1000	0.1000
	0301050401	对拉螺栓 M14	m	–	–	–	–	0.0500
	35020036	钢包木支撑	m·日	–	177.8250	107.0119	–	–
	35020003	顶托	个·日	–	107.1910	64.5056	–	–
	37090011	底托	个·日	–	107.1910	64.5056	–	–
	03150101	铁钉	kg	–	0.0205	0.0123	–	–
	35010006	定型大钢模板 标准及异型、接高，含螺栓	m^2·日	–	–	–	30.7200	–
	35020047	止水片	套	–	–	–	–	9.8765
	35020024	盘扣支撑	m·日	–	964.8335	241.2084	–	–
	34000011	其他材料费 占材料费	%	5.00	5.00	5.00	5.00	5.00
机械	9909000015	汽车式起重机 16t	台班	0.0020	0.0060	0.0017	0.0040	0.0040
	99210001	木工圆锯机（综合）	台班	0.0100	0.0200	0.0200	0.0200	0.0200
	9907000007	载重汽车 15t	台班	0.0020	0.0060	0.0017	0.0040	0.0040
	99460004	其他机具费 占人工费	%	1.50	1.50	1.50	1.50	1.50

北京市住房和城乡建设委员会

工作内容：模板及支架场内运输、刷脱模剂、安装、拆除、修理、清理模板粘结物及模内杂物、整理堆放等。 **单位：**m^2

编号				9-34	9-35	9-36	9-37	9-38
项目				梁<br>截面面积	柱	楼梯	电缆沟	轨顶风道
工料机名称			单位	消耗量				
人工	00010301	综合用工一类	工日	0.532	0.457	1.038	0.285	0.567
材料	05030007	板方材	m^3	0.0048	0.0020	0.0040	0.0140	0.0125
	35010003	复合木模板	m^2	0.3463	0.3463	0.2200	0.1220	0.3463
	14350009	脱模剂	kg	0.1000	0.1000	0.1000	0.1000	0.1000
	35090024	钢管	m·日	130.5050	88.8900	286.5240	–	76.6690
	35020036	钢包木支撑	m·日	177.8250	41.5308	–	–	136.3366
	35020003	顶托	个·日	107.1900	31.7460	32.1580	–	82.1822
	37090011	底托	个·日	107.1900	–	32.1580	–	82.1822
	03150101	铁钉	kg	0.0205	0.0609	–	–	0.0157
	35020023	扣件	个·日	–	57.1422	280.7000	–	–
	35020037	型钢(10#槽钢)柱箍	套·日	–	5.2500	–	–	–
	01010015	钢筋	kg	–	1.2232	–	–	–
	35020049	垫块	个	–	6.0020	–	–	–
	35020028	立杆垫板	套·日	–	–	32.1580	–	–
	0301050401	对拉螺栓 M14	m	0.0400	0.0500	–	–	0.0800
	35020024	盘扣支撑	m·日	964.8335	–	–	–	738.9169
	34000011	其他材料费 占材料费	%	5.00	5.00	5.00	5.00	5.00
机械	9909000015	汽车式起重机 16t	台班	0.0040	0.0040	0.0080	0.0020	0.0050
	99210001	木工圆锯机（综合）	台班	0.0160	0.0200	0.0200	0.0100	0.0200
	9907000007	载重汽车 15t	台班	0.0040	0.0040	0.0080	0.0020	0.0050
	99460004	其他机具费 占人工费	%	1.50	1.50	1.50	1.50	1.50

北京市住房和城乡建设委员会

工作内容：支架场内运输、安装、拆除、修理、整理堆放等。　　　　**单位**：m^2

编号				9-39	9-40	9-41	9-42	9-43
项目				板支撑	墙支撑	梁支撑	柱支撑	风道支撑
				高度 6m 以上				
				每增减 1m				
工料机名称			单位	消耗量				
人工	00010301	综合用工一类	工日	0.093	0.027	0.020	0.044	0.033
材料	05030007	板方材	m^3	–	0.0001	–	–	0.0001
	0301050401	对拉螺栓 M14	m	–	0.0056	–	–	0.0028
	35090024	钢管	m·日	14.8575	–	223.0250	2.2194	–
	35020024	盘扣支撑	m·日	121.0250	–	–	–	–
	35020023	扣件	个·日	–	–	246.6750	–	–
	35020036	钢包木支撑	m·日	–	–	–	5.3160	–
	35020037	型钢(10#槽钢)柱箍	套·日	–	–	–	0.4920	–
	01010015	钢筋	kg	–	–	–	0.0244	–
	35020049	垫块	个	–	–	–	0.2821	–
	34000011	其他材料费 占材料费	%	5.00	5.00	5.00	5.00	5.00
机械	9909000015	汽车式起重机 16t	台班	0.0010	0.0010	0.0010	0.0010	0.0010
	99210001	木工圆锯机（综合）	台班	0.0040	0.0040	0.0040	0.0040	0.0040
	9907000007	载重汽车 15t	台班	0.0010	0.0010	0.0010	0.0010	0.0010
	99460004	其他机具费 占人工费	%	1.50	1.50	1.50	1.50	1.50

北京市住房和城乡建设委员会

第四节 明挖区间模板

工作内容：模板及支架场内运输、刷脱模剂、安装、拆除、修理、清理模板粘结物及模内杂物、整理堆放等。 **单位：**m^2

编号				9-44	9-45	9-46	9-47	9-48
项目				底板	内衬墙	中隔墙、侧墙	顶板	检修沟
工料机名称			单位	消耗量				
人工	00010301	综合用工一类	工日	0.187	0.486	0.385	0.465	0.282
材料	05030007	板方材	m^3	0.0020	–	0.0020	0.0010	0.0100
	35010003	复合木模板	m^2	0.3463	–	0.3463	0.3463	0.1200
	14350009	脱模剂	kg	0.1000	0.1000	0.1000	0.1000	0.1000
	35090024	钢管	m·日	–	–	–	73.6100	–
	35010006	定型大钢模板 标准及异型、接高，含螺栓	m^2·日	–	30.7200	–	–	–
	0301050401	对拉螺栓 M14	m	–	–	0.0500	–	–
	35020036	钢包木支撑	m·日	–	–	–	130.9000	–
	35020003	顶托	个·日	–	–	–	78.9100	–
	37090011	底托	个·日	–	–	–	78.9100	–
	03150101	铁钉	kg	–	–	–	0.0200	–
	35020024	盘扣支撑	m·日	–	–	–	709.7047	–
	34000011	其他材料费 占材料费	%	5.00	5.00	5.00	5.00	5.00
机械	9909000015	汽车式起重机 16t	台班	0.0020	0.0030	0.0030	0.0040	0.0020
	99210001	木工圆锯机（综合）	台班	0.0100	0.0200	0.0200	0.0200	0.0100
	9907000007	载重汽车 15t	台班	0.0020	0.0030	0.0030	0.0040	0.0020
	99460004	其他机具费 占人工费	%	1.50	1.50	1.50	1.50	1.50

北京市住房和城乡建设委员会

第五节 暗挖车站模板

工作内容：模板及支架场内运输、刷脱模剂、垂直及洞内运输、安装、拆除、修理、清理模板粘结物及模内杂物、整理堆放等。　　**单位：**m^2

编号				9-49	9-50	9-51	9-52
项目				中板、底板	顶板	站台板	内衬墙
工料机名称			单位	消耗量			
人工	00010304	综合用工一类	工日	0.275	0.574	0.374	0.305
材料	05030007	板方材	m^3	0.0030	0.0125	0.0250	–
	35010003	复合木模板	m^2	0.0020	0.5100	0.3463	–
	35010004-3	组合钢模板	m^2·日	6.6160	–	–	–
	14350009	脱模剂	kg	0.1000	0.1000	0.1000	0.1000
	35090024	钢管	m·日	–	100.0000	60.1782	–
	35010006	定型大钢模板 标准及异型、接高，含螺栓	m^2·日	–	–	–	30.7200
	35020036	钢包木支撑	m·日	–	177.8250	107.0119	–
	35020003	顶托	个·日	–	107.1910	64.5056	–
	37090011	底托	个·日	–	107.1910	64.5056	–
	03150101	铁钉	kg	–	0.0205	0.0123	–
	35020024	盘扣支撑	m·日	–	964.8335	241.2084	–
	34000011	其他材料费 占材料费	%	5.00	5.00	5.00	5.00
机械	99210001	木工圆锯机（综合）	台班	0.0120	0.0240	0.0240	0.0240
	9907000007	载重汽车 15t	台班	0.0024	0.0072	0.0020	0.0048
	9909000403	桥式起重机 10t	台班	0.0008	0.0020	0.0010	0.0010
	99310001	电动三轮车	台班	0.0200	0.0200	0.0200	0.0200
	99460004	其他机具费 占人工费	%	1.50	1.50	1.50	1.50

北京市住房和城乡建设委员会

工作内容：模板及支架场内运输、刷脱模剂、垂直及洞内运输、安装、拆除、修理、清理模板粘结物及模内杂物、整理堆放等。 **单位：**m^2

编号				9-53	9-54
项目				中隔墙	轨顶风道
工料机名称			单位	消耗量	
人工	00010304	综合用工一类	工日	0.352	0.624
材料	05030007	板方材	m^3	0.0030	0.0125
	0301050401	对拉螺栓 M14	m	0.0500	0.0960
	35010003	复合木模板	m^2	0.3463	0.3463
	35090024	钢管	m·日	–	76.6700
	14350009	脱模剂	kg	0.1000	0.1000
	35020024	盘扣支撑	m·日	–	738.9169
	35020036	钢包木支撑	m·日	–	136.3366
	35020003	顶托	个·日	–	82.1822
	37090011	底托	个·日	–	82.1822
	03150101	铁钉	kg	–	0.0157
	34000011	其他材料费 占材料费	%	5.00	5.00
机械	99210001	木工圆锯机（综合）	台班	0.0240	0.0200
	9907000007	载重汽车 15t	台班	0.0060	0.0050
	9909000403	桥式起重机 10t	台班	0.0012	–
	99310001	电动三轮车	台班	0.0200	–
	99460004	其他机具费 占人工费	%	1.50	1.50

北京市住房和城乡建设委员会

工作内容：模板及支架场内运输、刷脱模剂、垂直及洞内运输、安装、拆除、修理、清理模板粘结物及模内杂物、整理堆放等。　　**单位：**m^2

编号				9-55	9-56	9-57	9-58	9-59	9-60
项目				底梁	天梁	中板梁	柱	楼梯	电缆沟
工料机名称			单位	消耗量					
人工	00010304	综合用工一类	工日	0.218	0.603	0.249	0.503	1.142	0.314
材料	05030007	板方材	m^3	0.0030	0.0060	0.0048	0.0020	0.0040	0.0140
	0301050401	对拉螺栓 M14	m	0.0400	0.0500	0.0400	0.0500	–	–
	35010003	复合木模板	m^2	0.0020	0.0020	0.0016	0.0010	0.2200	0.1220
	35010004-3	组合钢模板	m^2·日	4.6160	18.7760	15.0208	10.0590	–	–
	14350009	脱模剂	kg	0.1000	0.1000	0.1000	0.1000	0.1000	0.1000
	35090024	钢管	m·日	–	–	–	–	286.5240	–
	35020023	扣件	个·日	–	–	–	–	280.7000	–
	35020028	立杆垫板	套·日	–	–	–	–	32.1580	–
	35020003	顶托	个·日	–	–	–	–	32.1580	–
	37090011	底托	个·日	–	–	–	–	32.1580	–
	34000011	其他材料费 占材料费	%	5.00	5.00	5.00	5.00	5.00	5.00
机械	99210001	木工圆锯机（综合）	台班	0.0096	0.0240	0.0192	0.0240	0.0240	0.0120
	9907000007	载重汽车 15t	台班	0.0019	0.0060	0.0048	0.0048	0.0096	0.0024
	9909000403	桥式起重机 10t	台班	0.0006	0.0012	0.0010	0.0020	0.0016	0.0016
	99310001	电动三轮车	台班	0.0160	0.0200	0.0200	0.0200	0.0200	0.0200
	99460004	其他机具费 占人工费	%	1.50	1.50	1.50	1.50	1.50	1.50

北京市住房和城乡建设委员会

工作内容：支架场内运输、垂直及洞内运输、安装、拆除、修理、整理堆放等。　　　　**单位：**m^2

编号				9-61	9-62	9-63	9-64	9-65
项目				板支撑	墙支撑	梁支撑	柱支撑	风道支撑
				高度6m以上				
				每增减1m				
工料机名称			单位	消耗量				
人工	00010304	综合用工一类	工日	0.102	0.030	0.022	0.048	0.036
材料	05030007	板方材	m^3	–	0.0001	–	–	–
	0301050401	对拉螺栓 M14	m	–	0.0056	–	–	0.0028
	35090024	钢管	m·日	14.8575	–	223.0250	2.2194	–
	35020024	盘扣支撑	m·日	121.0250	–	–	–	–
	35020023	扣件	个·日	–	–	246.6750	–	–
	35020036	钢包木支撑	m·日	–	–	–	5.3160	–
	35020037	型钢(10#槽钢)柱箍	套·日	–	–	–	0.4920	–
	01010015	钢筋	kg	–	–	–	0.0244	–
	35020049	垫块	个	–	–	–	0.2821	–
	34000011	其他材料费 占材料费	%	5.00	5.00	5.00	5.00	5.00
机械	9909000015	汽车式起重机 16t	台班	0.0012	0.0012	0.0012	0.0012	0.0012
	99210001	木工圆锯机（综合）	台班	0.0040	0.0040	0.0040	0.0040	0.0040
	9907000007	载重汽车 15t	台班	0.0012	0.0012	0.0012	0.0012	0.0012
	99460004	其他机具费 占人工费	%	1.50	1.50	1.50	1.50	1.50

北京市住房和城乡建设委员会

第六节 盖挖车站模板

工作内容：平整场地、砌砖、浇筑混凝土、修理、整理、洞内运输及堆放等。 **单位：**m^2

编号				9-66	9-67	9-68	9-69
项目				盖挖中板		盖挖顶板	
				砖地模	混凝土地模	砖地模	混凝土地模
工料机名称			单位	消耗量			
人工	00010304	综合用工一类	工日	0.324	0.162	-	-
	00010301	综合用工一类	工日	-	-	0.292	0.113
材料	0413000901	标准砖 240mm×115mm×53mm	块	31.3720	-	28.5200	-
	8001000607	砌筑砂浆 DM7.5-HR	m^3	0.0168	-	0.0140	-
	8021000803	预拌混凝土 C20	m^3	-	0.0510	-	0.0510
	05030007	板方材	m^3	-	0.0060	-	0.0040
	5135000101	泵管 ϕ150	kg	-	0.0810	-	0.0470
	03150101	铁钉	kg	-	0.2500	-	0.2190
	34000011	其他材料费 占材料费	%	5.00	5.00	5.00	5.00
机械	9905000008	灰浆搅拌机 200L	台班	0.0060	-	0.0060	-
	99310001	电动三轮车	台班	0.0030	-	0.0020	-
	9909000403	桥式起重机 10t	台班	0.0006	0.0006	-	-
	99050003	混凝土振捣器(插入式)	台班	-	0.0050	-	0.0050
	99460004	其他机具费 占人工费	%	1.50	1.50	1.50	1.50

北京市住房和城乡建设委员会

第七节 暗挖区间模板

工作内容：1.模板台车：台车的吊装吊拆、移动就位、刷油、封堵加固等。2.钢模板：模板及支撑隧道内倒运、安装、拆除、堆放、刷油、清理等。

单位：见表

编号				9-70	9-71	9-72	9-73
项目				模板台车	弧形隧道		矩形隧道
				吊装吊拆	模板台车	钢模板	
				台次	m^2		
工料机名称			单位	消耗量			
人工	00010304	综合用工一类	工日	120.100	0.217	0.315	0.333
材料	37010001-2	钢轨	kg	10.0600	–	–	–
	37050008	道木	m^3	0.0250	–	–	–
	03150102	道钉	kg	0.5200	–	–	–
	05250002-1	垫木	m^3	1.5660	0.0050	–	–
	03130101	电焊条（综合）	kg	50.0400	0.3600	–	–
	05030007	板方材	m^3	–	0.0020	0.0030	0.0030
	0301050401	对拉螺栓 M14	m	–	0.0210	0.0400	0.0500
	35010003	复合木模板	m^2	–	0.0500	0.0020	0.0040
	14350009	脱模剂	kg	–	0.1000	0.1000	0.1000
	35010004-3	组合钢模板	m^2·日	–	–	8.7650	8.9920
	34000011	其他材料费 占材料费	%	5.00	5.00	5.00	5.00
机械	9925000202	交流电焊机 32kV·A	台班	8.3400	0.0400	–	–
	99210001	木工圆锯机（综合）	台班	10.6800	0.0200	0.0240	0.0240
	9907000007	载重汽车 15t	台班	1.7500	–	0.0048	0.0060
	9909000403	桥式起重机 10t	台班	1.3600	0.0002	0.0010	0.0012
	99310001	电动三轮车	台班	6.8000	0.0100	0.0200	0.0200
	99430016	模板台车	台班	–	0.0533	–	–
	99460004	其他机具费 占人工费	%	0.50	1.50	1.50	1.50

北京市住房和城乡建设委员会

第八节 竖井与其他模板

工作内容：模板及支架场内运输、刷脱模剂、垂直运输、安装、拆除、清理模板粘结物及模内杂物、整理堆放等。 **单位：**m^2

编号				9-74	9-75	9-76	9-77	9-78
项目				竖井内衬墙	风井、电梯井、电缆井、消防水池	小型构件	设备基础	腰梁
工料机名称			单位	消耗量				
人工	00010301	综合用工一类	工日	0.579	0.461	0.514	0.240	0.390
材料	05030007	板方材	m^3	0.0125	0.0030	0.0030	0.0030	0.0030
	0301050401	对拉螺栓 M14	m	0.0500	–	–	–	–
	35010003	复合木模板	m^2	0.5100	0.0020	0.0040	0.0020	0.3463
	35090024	钢管	m·日	100.0000	–	–	–	–
	35010004-3	组合钢模板	m^2·日	–	9.4150	10.9917	4.6160	0.0020
	14350009	脱模剂	kg	0.1000	0.1000	0.1000	0.1000	0.1000
	35020024	盘扣支撑	m·日	257.4447	–	–	–	–
	35020036	钢包木支撑	m·日	17.7825	–	–	–	–
	35020003	顶托	个·日	10.7191	–	–	–	–
	37090011	底托	个·日	10.7191	–	–	–	–
	03150101	铁钉	kg	0.0205	–	–	–	–
	34000011	其他材料费 占材料费	%	5.00	5.00	5.00	5.00	5.00
机械	99210001	木工圆锯机（综合）	台班	0.0240	0.0340	0.0540	0.0096	0.0014
	9907000007	载重汽车 15t	台班	0.0036	0.0048	0.0060	0.0019	0.0034
	9909000403	桥式起重机 10t	台班	0.0008	0.0010	0.0012	0.0006	0.0007
	99310001	电动三轮车	台班	0.0040	0.0050	0.0060	0.0032	–
	99460004	其他机具费 占人工费	%	1.50	1.50	1.50	1.50	1.50

北京市住房和城乡建设委员会

第十章　降水工程

北京市住房和城乡建设委员会

北京市住房和城乡建设委员会

说　　明

一、本章包括:管井安装,管井抽水 2 节共 16 个子目。

二、管井成孔中的土方外运执行第一章相应子目,泥浆制作及外运应另行计算。

三、管井成孔中子目中已包含管井回填,回填材料为级配砂石。

四、辐射井中钻机法施工中不含井字梁费用,井字梁混凝土及钢筋执行第四章明挖结构工程地梁相应子目。

五、降水项目中不含沟槽及沟槽内排水管线和管道铺设及障碍物处理费用,发生时另行计算。

六、管井抽水子目的水泵型号与设计图纸不同时,可调整。

工程量计算规则

一、水泥管井、钢管井、引渗井、辐射井的安装按设计图示以数量计算。

二、辐射水平井的安装按设计图示尺寸以长度计算。

三、管井和辐射井抽水以“根·日”计算。

北京市住房和城乡建设委员会

北京市住房和城乡建设委员会

第一节 管井安装

一、水泥管井安装

工作内容：挖探井、钻机成孔、安装井管、外壁灌砾料、井管装配、装水泵、洗井、封口、连接、试抽、管井拆除、灌砂回填等。 **单位：**根

编号				10-1	10-2	10-3	10-4
项目				水泥管井(ϕ600mm)		水泥管井(ϕ800mm)	
				井深 30m	每增减 1m	井深 30m	每增减 1m
工料机名称			单位	消耗量			
人工	00010501	综合用工二类	工日	4.247	0.156	5.662	0.208
材料	17290003	无砂混凝土管	m	31.5000	1.0500	31.5000	1.0500
	53330002	滤料(2-7mm)	t	8.8080	0.2936	15.6581	0.5220
	34070009	密目网	m^2	41.4500	1.3815	73.6889	2.4560
	01530004	铅丝	kg	20.2500	0.6750	36.0000	1.2000
	04050011-2	级配砂石	kg	6405.6000	213.5200	11387.7333	379.5911
	34000011	其他材料费 占材料费	%	1.00	1.00	1.00	1.00
机械	9903001101	反循环钻机 60P45A	台班	1.1000	0.0376	1.1000	0.0376
	9943000305	内燃空压机 ≤17m^3/min	台班	0.3200	0.0128	0.3200	0.0128
	9909000014	汽车式起重机 12t	台班	0.1152	0.0038	0.1152	0.0038
	9944000007	潜水泵 ϕ100mm	台班	0.3750	0.0125	0.6690	0.0223
	9944000006	泥浆泵 ϕ100mm	台班	0.6450	0.0215	1.1550	0.0385
	99460004	其他机具费 占人工费	%	1.50	1.50	1.50	1.50

北京市住房和城乡建设委员会

二、钢管井安装

工作内容：挖探井、钻机成孔、安装井管、外壁灌砾料、安装排水管、安装井盖、洗井、封口、灌砂回填等。 **单位：**根

编号				10-5	10-6
项目				钢管井（钻孔 ϕ600mm）	
				井深 35m	每增减 1m
工料机名称			单位	消耗量	
人工	00010501	综合用工二类	工日	4.721	0.149
材料	3601001702	井管（ϕ325 无缝钢管）	m	19.3800	0.7752
	3601001701	井管（ϕ325 桥式钢管）	m	16.3200	0.2448
	53330002	滤料（2-7mm）	t	13.2350	0.3781
	34070009	密目网	m^2	39.2900	1.1224
	01530004	铅丝	kg	19.0400	0.6400
	03130101	电焊条（综合）	kg	11.8300	0.3380
	04050011-2	级配砂石	kg	4782.8480	136.6528
	34000011	其他材料费 占材料费	%	1.00	1.00
机械	9903001101	反循环钻机 60P45A	台班	1.2833	0.0402
	9943000305	内燃空压机 ≤17m^3/min	台班	0.3200	0.0160
	9925000202	交流电焊机 32kV·A	台班	0.4760	0.0136
	9909000014	汽车式起重机 12t	台班	0.1344	0.0038
	9944000007	潜水泵 ϕ100mm	台班	0.3750	0.0125
	9944000006	泥浆泵 ϕ100mm	台班	0.6450	0.0215
	99460004	其他机具费 占人工费	%	1.50	1.50

北京市住房和城乡建设委员会

三、引渗井安装

工作内容：挖探井、钻机成孔、安装井管、外壁灌砾料、安装排水管、安装井盖、洗井、封口等。 **单位：**根

编号				10-7	10-8
项目				引渗井（钻孔 ϕ150mm）	
				井深 30m	每增减 1m
工料机名称			单位	消耗量	
人工	00010501	综合用工二类	工日	2.441	0.084
材料	1725150108	PP-R 管 ϕ50	m	64.4400	1.7184
	53330002	滤料（2-7mm）	t	7.2600	0.1940
	34070009	密目网	m^2	5.1800	0.1380
	01530004	铅丝	kg	7.5000	0.2000
	36010026	五防井盖（重型）	套	0.3400	-
	34000011	其他材料费 占材料费	%	1.00	1.00
机械	9903001113	地质钻机 150 型	台班	0.8920	0.0280
	9943000305	内燃空压机 ≤17m^3/min	台班	0.3200	0.0128
	99460004	其他机具费 占人工费	%	1.50	1.50

北京市住房和城乡建设委员会

四、辐射井安装

工作内容:挖探槽、护壁管安装、接口防水、井盖、封底、试抽、辐射井拆除、灌砂回填等。 **单位:**根

编号				10-9	10-10	10-11	10-12
项目				辐射井			
				沉井法混凝土管 ϕ3000mm		钻机法混凝土管 ϕ2600mm	
				井深 30m	每增减 1m	井深 30m	每增减 1m
工料机名称			单位	消耗量			
人工	00010501	综合用工二类	工日	15.480	0.516	4.644	0.155
材料	1729001427	钢筋混凝土管 ϕ3000	m	31.5000	1.0500	-	-
	1729001425	钢筋混凝土管 ϕ2600	m	-	-	31.5000	1.0500
	1333030206	SBS 改性沥青油毡防水卷材 4mm	m^2	-	-	244.3400	8.4780
	36010026	五防井盖(重型)	套	0.3400	-	0.3400	-
	13370002	橡胶止水带	m	105.6900	3.3630	-	-
	8021000803	预拌混凝土 C20	m^3	4.9500	0.2475	-	-
	04050011-2	级配砂石	kg	164.4000	5.4800	118.6500	3.3900
	34000011	其他材料费 占材料费	%	1.00	1.00	1.00	1.00
机械	9909000017	汽车式起重机 25t	台班	4.8000	0.1600	1.8000	0.0600
	9944000901	污泥泵 ϕ100mm	台班	3.6000	0.1000	5.4000	0.1800
	9909000403	桥式起重机 10t	台班	1.9200	0.0600	-	-
	9903001109	钻机 GD-35	台班	-	-	12.6000	0.4200
	99440012	气泵	台班	-	-	3.2000	0.1600
	99460004	其他机具费 占人工费	%	1.50	1.50	1.50	1.50

北京市住房和城乡建设委员会

五、辐射水平井安装

工作内容：安装施工平台、安全梯、护网、钻孔、安装井管、封口、连接、试抽等。　　　　**单位：**m

编号				10-13	10-14
项目				水平井	
				细颗粒地层	砂砾地层
工料机名称			单位	消耗量	
人工	00010501	综合用工二类	工日	0.129	0.206
材料	1701000904	钢管缠丝滤水管（ϕ114mm）	m	1.0000	1.0000
	03151315	钻杆	m	0.0100	0.0200
	0315130714	钻头 ϕ115	个	0.0100	0.0100
	33010049	井点托架	套	0.0010	0.0010
	34000011	其他材料费 占材料费	%	1.00	1.00
机械	9903001102	工程钻机 DFM1504	台班	0.0320	0.0480
	9909000252	电动卷扬机 单筒慢速 3t	台班	0.0160	0.0240
	9909000017	汽车式起重机 25t	台班	0.0040	0.0040
	99440012	气泵	台班	0.0160	0.0240
	9943000305	内燃空压机 ≤17m^3/min	台班	–	0.0480
	99460004	其他机具费 占人工费	%	1.50	1.50

北京市住房和城乡建设委员会

第二节 管井抽水

工作内容:抽水、值班、管道维护等。 **单位:**根·日

编号				10-15	10-16
项目				管井	辐射井
工料机名称			单位	消耗量	
人工	00010501	综合用工二类	工日	0.090	0.108
材料	34000011	其他材料费 占材料费	%	1.00	1.00
机械	9944001001	潜水泵 ϕ50mm	台班	3.0000	-
	9944000007	潜水泵 ϕ100mm	台班	-	3.0000
	99460004	其他机具费 占人工费	%	1.50	1.50

北京市住房和城乡建设委员会

第十一章　监测工程

北京市住房和城乡建设委员会

北京市住房和城乡建设委员会

说　　明

一、本章包括:监测点布设,监测 2 节共 65 个子目。

二、测点布设在行车道路上,需要进行交通导行的,其费用另行计算。

三、过既有线监测中不含与既有线相关运营单位的配合费用,发生时需根据实际方案另行计算。

四、连续监测时,监测室的建设费、场地租用费以及建立监测室所需用电线、连接线等费用均未计入子目内,发生时应根据工程实际方案另行计算。

五、监测工程中使用的仪器均按国产仪器综合编制。

六、按深度划分子目的监测项目,其深度指地面标高与监测项目设计底标高之差。

七、控制点布设执行基准点布设子目。

八、既有线变形监测以“5 个测点”为一组,不足 5 个时按一组计算。

工程量计算规则

一、基准点布设按设计图示以点计算。

二、地表沉降和位移测点布设按设计图示以点计算。

三、建筑物变形测点布设中沉降和倾斜按设计图示以点计算。

四、土体分层沉降、桩体变形、孔隙水压力、水位观察孔和界面土压力测点布设按设计图示以孔计算。

五、地下管线沉降、位移测点布设按设计图示以点计算。

六、混凝土构筑物钢筋应力、混凝土应变测点布设按设计图示以点计算。

北京市住房和城乡建设委员会

七、隧道沉降和收敛、既有线变形测点布设按设计图示以点计算。

八、沉降、位移监测按设计图示数量(不含基点数量)和监测频率的乘积以“点·次”计算;倾斜和裂缝监测按设计图示数量和监测频率的乘积分别以“点·次”和“条·次”计算。

九、土体分层沉降和桩体变形监测按设计图示数量和监测频率的乘积以“孔·次”计算。

十、钢筋应力、混凝土应变监测按设计图示数量和监测频率的乘积以“点·次”计算。

十一、孔隙水压力、界面土压力和水位观察孔监测按设计图示数量和监测频率的乘积以“孔·次”计算;地下管线沉降、位移监测按设计图示数量和监测频率的乘积以“点·次”计算。

十二、隧道沉降及收敛监测按设计图示数量和监测频率的乘积以“点·次”计算。

十三、既有线变形监测按监测方案以“组·日”(24小时)计算。

北京市住房和城乡建设委员会

第一节　监测点布设

一、基准点布设

工作内容:测点布置、挖孔或钻孔、取土及渣土外运、钢护管加工与埋设、预埋件加工埋设、钢筋制作安装、砌检查井、浇灌混凝土、保护盖加工与安装、测读初值等。

单位:点

编号				11-1	11-2
项目				深埋钢管	钢筋混凝土
工料机名称			单位	消耗量	
人工	00010501	综合用工二类	工日	2.778	3.402
材料	8021000805	预拌混凝土 C25	m^3	0.1000	0.6600
	01010003	钢筋 ϕ10 以外	kg	–	28.5000
	0413000201	页岩砖 240mm×115mm×53mm	块	242.5150	–
	8001000605	砌筑砂浆 DM5.0-LR	m^3	0.1206	–
	34130025	抗蚀金属标志	个	1.0000	1.0000
	03150920	抗蚀预埋件	套	1.0000	1.0000
	1703000014	镀锌钢管 *DN*100	m	6.0000	–
	02190014	保护圈盖	套	1.0000	1.0000
	34000011	其他材料费 占材料费	%	1.00	1.00
机械	99030042	汽车钻孔机 ϕ300~ϕ400	台班	0.3100	–
	99410001	水钻	台班	–	0.3100
	87110205	水准仪	台班	0.2000	0.2000
	9907000010	自卸汽车 8t	台班	0.1000	0.1200
	99460004	其他机具费 占人工费	%	1.50	1.50

北京市住房和城乡建设委员会

二、地表沉降与位移测点布设

工作内容：测点布置、挖孔或钻孔、取土及渣土外运、钢护管加工与埋设、保护盖加工与安装、预埋件加工埋设、浇灌混凝土、测读初值等。

单位：点

编号				11-3	11-4	11-5
项目				混凝土结构	沥青混凝土结构	其他地面
工料机名称			单位	消耗量		
人工	00010501	综合用工二类	工日	0.567	0.463	0.516
材料	8021000805	预拌混凝土 C25	m^3	0.0250	0.0200	0.0230
	01010003	钢筋 ϕ10 以外	kg	2.9640	2.9640	2.9640
	1703000017	镀锌钢管 *DN*150	m	0.5000	0.5000	0.5000
	02190014	保护圈盖	套	1.0000	1.0000	1.0000
	34000011	其他材料费 占材料费	%	1.00	1.00	1.00
机械	99030050	液压钻孔机	台班	0.0500	0.0400	0.0450
	9907000010	自卸汽车 8t	台班	0.0200	0.0200	0.0200
	87110205	水准仪	台班	0.1000	0.1000	0.1000
	87110204	全站仪	台班	0.1000	0.1000	0.1000
	99460004	其他机具费 占人工费	%	1.50	1.50	1.50

北京市住房和城乡建设委员会

三、建筑物变形测点布设

工作内容：1.沉降：测点布置、预埋件加工安装、测读初值等。2.倾斜：测点布置、手提钻打孔、预埋件加工安装、测读初值等。　　**单位：**点

编		号		11-6	11-7
项		目		沉降	倾斜
工料机名称			单位	消耗量	
人工	00010501	综合用工二类	工日	0.390	1.170
材料	03150920	抗蚀预埋件	套	1.0000	3.0000
	34000011	其他材料费 占材料费	%	1.00	1.00
机械	99030029	手动钻孔机	台班	0.3200	0.9600
	87110205	水准仪	台班	0.2000	–
	9909000703	平台升降车 20m	台班	–	0.9600
	87110204	全站仪	台班	–	0.4000
	99460004	其他机具费 占人工费	%	1.50	1.50

北京市住房和城乡建设委员会

四、土体分层沉降测点布设

工作内容：测孔布置、钻孔、取土及渣土外运、沉降管及磁环安装、回填、预埋件加工埋设、做保护盖、测读初值等。　　**单位：**孔

编号				11-8	11-9	11-10	11-11
项目				土体分层沉降			
				深 10m 以内	深 20m 以内	深 30m 以内	每增 1m
工料机名称			单位	消耗量			
人工	00010501	综合用工二类	工日	4.602	8.910	11.418	0.483
材料	04011101	水泥(综合)	kg	387.6000	775.2000	1162.8000	38.7600
	04090007	膨润土	kg	115.7100	231.4200	347.1300	11.5710
	14350001	促进剂 KA	kg	7.8300	15.6600	23.4800	0.7827
	17310045	塑料注浆阀管	m	10.5000	21.0000	31.5000	1.0500
	5533000001	磁环 ϕ70	个	(5.5000)	(11.0000)	(16.5000)	(0.5500)
	1713000001	导向铝管 ϕ30	m	11.0000	22.0000	33.0000	1.1000
	02190014	保护圈盖	套	1.0000	1.0000	1.0000	–
	34000011	其他材料费 占材料费	%	1.00	1.00	1.00	1.00
机械	9944000005	泥浆泵 ϕ50mm	台班	0.4800	0.9760	1.4890	0.0600
	9907000010	自卸汽车 8t	台班	0.1200	0.2400	0.3600	0.0120
	99030042	汽车钻孔机 ϕ300~ϕ400	台班	0.4800	0.9760	1.4890	0.0600
	87460407	分层沉降仪	台班	0.4000	0.8000	1.2000	0.0400
	99460004	其他机具费 占人工费	%	1.50	1.50	1.50	1.50

北京市住房和城乡建设委员会

五、桩体变形测点布设

工作内容：测斜管绑扎、安装、做保护圈盖、测读初值等。 **单位：**孔

编号				11-12	11-13	11-14	11-15
项目				桩体变形			
				深 10m 以内	深 20m 以内	深 30m 以内	每增 1m
工料机名称			单位	消耗量			
人工	00010501	综合用工二类	工日	1.320	1.716	2.111	0.139
材料	02190014	保护圈盖	套	1.0000	1.0000	1.0000	–
	17310043	塑料测斜管	m	(11.0000)	(22.0000)	(33.0000)	(1.1000)
	03150717	铁丝 13#～17#	kg	1.1000	2.2000	3.3000	0.1100
	34000011	其他材料费 占材料费	%	1.00	1.00	1.00	1.00
机械	87460404	测斜仪	台班	0.4000	0.5200	0.6400	0.0213
	9907000010	自卸汽车 8t	台班	0.0100	0.0100	0.0100	0.0003
	9909000004	履带式起重机 15t	台班	0.0100	0.0100	0.0100	0.0003
	99460004	其他机具费 占人工费	%	1.50	1.50	1.50	1.50

北京市住房和城乡建设委员会

六、孔隙水压力测孔布设

工作内容：测孔布置、钻孔、取土及渣土外运、测试元件安装、接线、埋设泥球止水隔离层、回填、做保护圈盖、测读初值等。　　**单位：**孔

编号				11-16	11-17	11-18	11-19
项目				孔隙水压力			
				深 10m 以内	深 20m 以内	深 30m 以内	每增 1m
工料机名称			单位	消耗量			
人工	00010501	综合用工二类	工日	3.948	4.935	5.922	0.415
材料	04090007	膨润土	kg	304.5000	304.5000	304.5000	-
	24110016	孔隙水压计	支	(2.0000)	(5.0000)	(7.0000)	(0.2333)
	2823000401	屏蔽线 二芯	m	12.6000	23.1000	33.6000	1.1200
	02190014	保护圈盖	套	1.0000	1.0000	1.0000	-
	34000011	其他材料费 占材料费	%	1.00	1.00	1.00	1.00
机械	99030042	汽车钻孔机 ϕ300~ϕ400	台班	0.4800	0.9760	1.4890	0.0570
	87310601	频率接收仪	台班	0.2000	0.2500	0.3000	0.0100
	9907000010	自卸汽车 8t	台班	0.1200	0.2400	0.3600	0.0120
	99460004	其他机具费 占人工费	%	1.50	1.50	1.50	1.50

北京市住房和城乡建设委员会

工作内容：测孔布置、钻孔、取土及渣土外运、测试元件安装、接线、埋设泥球止水隔离层、回填、做保护圈盖、测读初值等。　　**单位：**孔

编号				11-20	11-21	11-22	11-23
项目				水位观察孔			
				深 10m 以内	深 20m 以内	深 30m 以内	每增 1m
工料机名称			单位	消耗量			
人工	00010501	综合用工二类	工日	3.948	4.935	5.922	0.415
材料	04090007	膨润土	kg	304.5000	304.5000	304.5000	-
	17070020-2	无缝钢管	kg	67.2900	132.0000	201.8700	6.7290
	1731001002	软式透水管 ϕ50	m	1.1000	1.1000	1.1000	0.0367
	02190014	保护圈盖	套	1.0000	1.0000	1.0000	-
	34000011	其他材料费 占材料费	%	1.00	1.00	1.00	1.00
机械	99030042	汽车钻孔机 ϕ300～ϕ400	台班	0.4800	0.9760	1.4890	0.0570
	87010402	水位计	台班	0.2000	0.3200	0.4000	0.0133
	9907000010	自卸汽车 8t	台班	0.1200	0.2400	0.3600	0.0120
	99460004	其他机具费 占人工费	%	1.50	1.50	1.50	1.50

北京市住房和城乡建设委员会

七、地下管线沉降与位移测点布设

工作内容：测点布置、成孔、取土及渣土外运、预埋件加工制作安装、接线、做保护圈盖、回填、测读初值等。　　**单位：**点

编号				11-24	11-25
项目				地下管线沉降、位移	
				深 10m 以内	深 20m 以内
工料机名称			单位	消耗量	
人工	00010501	综合用工二类	工日	3.252	3.924
材料	34130020	管线抱箍标志	个	1.0000	1.0000
	34000011	其他材料费 占材料费	%	1.00	1.00
机械	9903001114	轻便钻机 XJ-100	台班	0.4800	0.9760
	87110205	水准仪	台班	0.1000	0.1000
	87110204	全站仪	台班	0.2000	0.2000
	9907000010	自卸汽车 8t	台班	0.1200	0.2400
	99460004	其他机具费 占人工费	%	1.50	1.50

北京市住房和城乡建设委员会

八、混凝土构筑物钢筋应力与混凝土应变测点布设

工作内容：测点布置、钢筋上安装钢筋应力计、排线固定、保护装置、测读初值等。　　**单位：**点

编号				11-26	11-27	11-28	11-29
项目				钢筋应力		混凝土应变	
				地下	地上	地下	地上
工料机名称			单位	消耗量			
人工	00010501	综合用工二类	工日	1.980	1.320	1.620	1.080
材料	02190014	保护圈盖	套	1.0000	1.0000	1.0000	1.0000
	2823000401	屏蔽线 二芯	m	31.5000	21.0000	31.5000	21.0000
	24690023	钢筋应力计	个	(1.0000)	(1.0000)	–	–
	24690024	混凝土应变计	个	–	–	(1.0000)	(1.0000)
	34000011	其他材料费 占材料费	%	1.00	1.00	1.00	1.00
机械	9909000703	平台升降车 20m	台班	–	0.1200	–	0.1200
	9925000202	交流电焊机 32kV·A	台班	0.1700	0.1700	–	–
	99030029	手动钻孔机	台班	–	–	0.2300	0.2300
	87060302	静态电阻应变仪	台班	–	–	0.2300	0.2300
	9909000004	履带式起重机 15t	台班	0.1700	–	0.1700	–
	87310601	频率接收仪	台班	0.1700	0.1700	–	–
	99460004	其他机具费 占人工费	%	1.50	1.50	1.50	1.50

北京市住房和城乡建设委员会

九、界面土压力测点布设

工作内容：测点布置、测试元件标定、安装土压计、测读初值等。 **单位：**孔

编号				11-30	11-31	11-32	11-33
项目				界面土压力			
				深 10m 以内	深 20m 以内	深 30m 以内	每增 1 米
工料机名称			单位	消耗量			
人工	00010501	综合用工二类	工日	3.948	4.738	5.685	0.415
材料	2823000401	屏蔽线 二芯	m	11.0000	21.0000	31.0000	1.0333
	1703000010	镀锌钢管 *DN*70	m	0.3000	0.3000	0.3000	0.0100
	24110017	土压计	只	(2.0000)	(5.0000)	(7.0000)	(0.2333)
	2906060102	PVC 阻燃塑料管 20	m	11.0000	21.0000	31.0000	1.0333
	34000011	其他材料费 占材料费	%	1.00	1.00	1.00	1.00
机械	99030042	汽车钻孔机 $\phi300 \sim \phi400$	台班	0.4800	0.9760	1.4890	0.0570
	9907000010	自卸汽车 8t	台班	0.1200	0.2400	0.3600	0.0200
	87310601	频率接收仪	台班	0.1700	0.1700	0.1700	0.0057
	99460004	其他机具费 占人工费	%	1.50	1.50	1.50	1.50

北京市住房和城乡建设委员会

十、隧道沉降与收敛测点布设

工作内容：测点布设、仪器标定、埋设、测读初值等。 **单位：**点

编号				11-34	11-35
项目				隧道沉降	隧道收敛
工料机名称			单位	消耗量	
人工	00010501	综合用工二类	工日	0.581	0.438
材料	03150920	抗蚀预埋件	套	1.0000	–
	03150926	收敛预埋件	套	–	4.0000
	34000011	其他材料费 占材料费	%	1.00	1.00
机械	99030029	手动钻孔机	台班	0.1200	0.1200
	87110205	水准仪	台班	0.2000	–
	8731030004	数显收敛计 JSS30A/30	台班	–	0.2000
	99460004	其他机具费 占人工费	%	1.50	1.50

北京市住房和城乡建设委员会

十一、既有线变形测点布设

工作内容：测点布设、仪器标定、埋设、测读初值等。　　　　**单位：**点

编号				11-36	11-37	11-38	11-39
项目				自动化监测			
				结构沉降	裂缝变形	走行轨水平变形	走行轨纵向变形
工料机名称			单位	消耗量			
人工	00010501	综合用工二类	工日	5.550	5.268	4.512	4.116
材料	24690007	静力水准仪	套	(1.0000)	–	–	–
	24240002	裂缝计	套	–	(1.0000)	–	–
	24240001	变位计	只	–	–	(1.0000)	–
	37270023	轨道扭转传感器	台	–	–	–	(1.0000)
	33010014	单向安装支架	套	1.0000	–	–	–
	18270014	三向安装支架	套	–	–	1.0000	1.0000
	2827000001	信号电缆 PY02 250V 24 芯	m	40.0000	40.0000	40.0000	40.0000
	55270502	瞬时保护模块	个	0.1300	0.1300	0.1300	0.1300
	57290007	集线箱	个	0.1300	0.1300	0.1300	0.1300
	17310037	连通管	m	3.2500	–	–	–
	13150010	连通液	kg	5.7800	–	–	–
	03150920	抗蚀预埋件	套	–	2.0000	–	–
	34000011	其他材料费 占材料费	%	1.00	1.00	1.00	1.00
机械	99030029	手动钻孔机	台班	0.3500	0.4900	0.3600	0.3600
	87311601	数据采集系统	台班	0.2000	0.2000	0.2000	0.2000
	99460004	其他机具费 占人工费	%	1.50	1.50	1.50	1.50

北京市住房和城乡建设委员会

第二节　监测

一、地上结构监测

工作内容：仪器架设、现场测量、数据记录、成果计算、分析处理和编制监测报告等。　　**单位：**见表

编号				11-40	11-41	11-42	11-43
项目				沉降	位移	倾斜	裂缝
				点·次		座·次	条·次
工料机名称			单位	消耗量			
人工	00010501	综合用工二类	工日	0.184	0.198	1.091	0.168
机械	87110205	水准仪	台班	0.1530	–	–	–
	87110204	全站仪	台班	–	0.1100	0.8800	–
	8746040008	智能裂缝测宽仪 PTS-C10	台班	–	–	–	0.1200
	99460004	其他机具费 占人工费	%	1.50	1.50	1.50	1.50

北京市住房和城乡建设委员会

二、土体分层沉降监测

工作内容：仪器架设、现场测量、数据记录、成果计算、分析处理和编制监测报告等。　　**单位：**孔·次

编号				11-44	11-45	11-46	11-47
项目				土体分层沉降			
				10m 以内	20m 以内	30m 以内	每增 1m
工料机名称			单位	消耗量			
人工	00010501	综合用工二类	工日	0.270	0.510	0.750	0.028
机械	87460407	分层沉降仪	台班	0.1500	0.3290	0.4840	0.0220
	99460004	其他机具费 占人工费	%	1.50	1.50	1.50	1.50

北京市住房和城乡建设委员会

三、桩体变形监测

工作内容：仪器架设、现场测量、数据记录、成果计算、分析处理和编制监测报告等。　　**单位：**孔·次

编号				11-48	11-49	11-50	11-51
项目				桩体变形			
				7m 以内	20m 以内	30m 以内	每增 1m
工料机名称			单位	消耗量			
人工	00010501	综合用工二类	工日	0.214	0.459	0.765	0.031
机械	87460404	测斜仪	台班	0.1200	0.2570	0.4290	0.0270
	99460004	其他机具费 占人工费	%	1.50	1.50	1.50	1.50

北京市住房和城乡建设委员会

四、钢筋应力与混凝土应变监测

工作内容：仪器架设、现场测量、数据记录、成果计算、分析处理和编制监测报告等。　　**单位：**点·次

编号				11-52	11-53	11-54	11-55
项目				钢筋应力		混凝土应变	
				地上	地下	地上	地下
工料机名称			单位	消耗量			
人工	00010501	综合用工二类	工日	0.144	0.168	0.173	0.202
机械	87310601	频率接收仪	台班	0.1200	0.1400	0.1440	0.1680
	99460004	其他机具费 占人工费	%	1.50	1.50	1.50	1.50

北京市住房和城乡建设委员会

五、孔隙水压力、界面土压力、水位和地下管线沉降位移监测

工作内容:仪器架设、现场测量、数据记录、成果计算、分析处理和编制监测报告等。　　**单位:**见表

编号				11-56	11-57	11-58	11-59
项目				孔隙水压力	界面土压力	水位	地下管线沉降、位移
				孔·次			点·次
工料机名称			单位	消耗量			
人工	00010501	综合用工二类	工日	0.720	0.780	0.132	0.408
机械	87310601	频率接收仪	台班	0.1800	0.1900	-	-
	87010402	水位计	台班	-	-	0.1500	-
	87110205	水准仪	台班	-	-	-	0.1600
	87110204	全站仪	台班	-	-	-	0.1800
	99460004	其他机具费 占人工费	%	1.50	1.50	1.50	1.50

北京市住房和城乡建设委员会

六、隧道沉降与收敛监测

工作内容：仪器架设、现场测量、数据记录、成果计算、分析处理和编制监测报告等。 **单位：**点·次

编		号		11-60	11-61
项		目		隧道沉降	隧道收敛
工料机名称			单位	消耗量	
人工	00010501	综合用工二类	工日	0.210	0.240
机械	87110205	水准仪	台班	0.1600	–
	8731030004	数显收敛计 JSS30A/30	台班	–	0.2000
	99460004	其他机具费 占人工费	%	1.50	1.50

北京市住房和城乡建设委员会

七、既有线变形监测

工作内容：测试、数据采集、成果计算、分析处理和编制监测报告等。 **单位：**组·日

编号				11-62	11-63	11-64	11-65
项目				自动化监测			
				结构沉降	裂缝变形	走行轨水平变形	走行轨纵向变形
工料机名称			单位	消耗量			
人工	00010501	综合用工二类	工日	0.720	0.726	0.732	0.738
机械	87311601	数据采集系统	台班	1.2000	1.2100	1.2200	1.2300
	99460004	其他机具费 占人工费	%	1.50	1.50	1.50	1.50

北京市住房和城乡建设委员会

编委会名单

主审单位： 北京市住房和城乡建设委员会

共同主编单位： 北京市建设工程造价管理总站(北京市房屋修缮工程定额管理站)
北京城建集团有限责任公司　北京建工集团有限责任公司
中国建筑一局(集团)有限公司　中国建筑第三工程局有限公司
中国建筑第八工程局有限公司　中铁建设集团有限公司
中国新兴建设开发有限责任公司　中建安装集团有限公司
北京市建设工程招标投标和造价管理协会

参编单位： 北京城建轨道交通建设工程有限公司
北京建工土木工程有限公司
北京住总集团有限责任公司

领导小组成员： 王　飞　赵英杰　丁　胜　李晓涛　宋其龙　冯志祥　陈炜文　阮景云
石向东　凌振军　宛　春　赵　勇　陈代华　樊　军　吴爱国　陈卫国
李永明　梅洪亮　马健峰　常永春　吴继华　林　萌

综合组成员： 冯志祥　王允国　王京阳　刘志超　李　卉　杨允斌

编审人员： 曾伟源　任文博　崔　凯　韩　冰　曹彤星　刘凤元　张勇胜　郭建国
顾　青　王志年　张爱平　杨立新　罗全勇　冯利华　李永洁　邵海真
沈春燚　刘军华　季明春　屈　望　臧秀华　李剑慧　郭彦霞　代丽丽
袁明省　王兴奎　胡云录　王凤华　苑晓敏　李铁占　闫明光　杨　娟
李丽娜　李　征　凌立静　李　英　宗　慧　凌研方　陈雪晶　郑克宇
庞建昕　谢　芳　窦丙凯　尹　伟　陈泽林　王根成　刘兵科　马云新
赵旭东　孙志欣

北京市住房和城乡建设委员会

北京市住房和城乡建设委员会
