# Supplementary material for: Carbon emission quantification analysis of excavation engineering under road transport conditions
Source: PLoS One. 2024 Dec 31;19(12):e0315765. doi: 10.1371/journal.pone.0315765 (PMC11687867; doi:10.1371/journal.pone.0315765)
Supplement: S2 File — (PDF) [file pone.0315765.s002.pdf]

前　　言

根据住房和城乡建设部《关于印发〈2014 年工程建设标准规范制订、修订计划〉的通知》(建标[2013]169 号)的要求，标准编制组经广泛调查研究，认真总结实践经验，参考有关国际标准和国外先进标准，并在广泛征求意见的基础上，编制了本标准。

本标准的主要技术内容是：1. 总则；2. 术语和符号；3. 基本规定；4. 运行阶段碳排放计算；5. 建造及拆除阶段碳排放计算；6. 建材生产及运输阶段碳排放计算。

本标准由住房和城乡建设部负责管理，由中国建筑科学研究院有限公司负责具体技术内容的解释。执行过程中如有意见或建议，请寄送中国建筑科学研究院有限公司(地址：北京市北三环东路 30 号，邮编：100013)。

本标准主编单位：中国建筑科学研究院有限公司
中国建筑标准设计研究院有限公司

本标准参编单位：中国建筑技术集团有限公司
四川大学
北京华通三可咨询有限公司
清华大学
中国建材检验认证集团股份有限公司
中国质量认证中心
中国标准化研究院
深圳市建筑科学研究院股份有限公司
北京建筑技术发展有限责任公司
北京环境交易所有限公司
广州碳排放权交易中心有限公司
北京天正软件股份有限公司

住房城乡建设部信息公开
浏览专用

四川华构住宅工业有限公司
中建三局第一建设工程有限责任公司
北京金茂绿建科技有限公司
成都亿科环境科技有限公司

本标准主要起草人员：徐　伟　李本强　张时聪　王建军
王洪涛　王侃宏　孙德宇　蒋　荃
鲁传一　佟　庆　马丽萍　韩建军
陈建华　刘俊跃　罗淑湘　邹　毅
保　佶　何更新　窦春伦　候键频
车大桥　尹　奎　左建波　张　莉

本标准主要审查人员：徐宏庆　孟庆林　冯　雅　白　泉
葛　坚　曹永敏　王云新　闫增峰
鲍宇清

住房城乡建设部信息公开
浏览专用

目　次

1　总则 …………………………………………………… 1
2　术语和符号 ………………………………………… 2
2.1　术语 …………………………………………… 2
2.2　符号 …………………………………………… 2
3　基本规定 …………………………………………… 6
4　运行阶段碳排放计算 ……………………………… 7
4.1　一般规定 ……………………………………… 7
4.2　暖通空调系统 ………………………………… 8
4.3　生活热水系统 ………………………………… 10
4.4　照明及电梯系统 ……………………………… 11
4.5　可再生能源系统 ……………………………… 12
5　建造及拆除阶段碳排放计算 ……………………… 14
5.1　一般规定 ……………………………………… 14
5.2　建筑建造 ……………………………………… 14
5.3　建筑拆除 ……………………………………… 16
6　建材生产及运输阶段碳排放计算 ………………… 18
6.1　一般规定 ……………………………………… 18
6.2　建材生产 ……………………………………… 18
6.3　建材运输 ……………………………………… 19
附录A　主要能源碳排放因子 ……………………… 21
附录B　建筑物运行特征 …………………………… 23
附录C　常用施工机械台班能源用量 ……………… 27
附录D　建材碳排放因子 …………………………… 34

住房城乡建设部信息公开
浏览专用

附录E　建材运输碳排放因子 …………………………………… 37
本标准用词说明 ………………………………………………… 38
引用标准名录 …………………………………………………… 39

住房城乡建设部信息公开
浏览专用

Contents

1 General Provisions ······ 1
2 Terms and Symbols ······ 2
2.1 Terms ······ 2
2.2 Symbols ······ 2
3 Basic Requirements ······ 6
4 Carbon Emission Calculation for Operation Period ······ 7
4.1 General Requirements ······ 7
4.2 Building HVAC System ······ 8
4.3 Domestic Hot Water System ······ 10
4.4 Lighting and Elevator System ······ 11
4.5 Renewable Energy System ······ 12
5 Carbon Emission Calculation for Construction and Demolition Period ······ 14
5.1 General Requirements ······ 14
5.2 Building Construction ······ 14
5.3 Building Demolition ······ 16
6 Carbon Emission Calculation for Building Material Production and Transportation Period ······ 18
6.1 General Requirements ······ 18
6.2 Building Material Production ······ 18
6.3 Building Material Transportation ······ 19
Appendix A Main Energy Carbon Emission Factor ······ 21
Appendix B Building Using Characteristics ······ 23
Appendix C Fuel Consumption Rating Per Machine Per Team ······ 27

住房城乡建设部信息公开 浏览专用

Appendix D Carbon Emission Factor for Building Material ······ 34
Appendix E Carbon Emission Factor for Building Material Transportation ······ 37
Explanation of Wording in This Standard ······ 38
List of Quoted Standards ······ 39

住房城乡建设部信息公开
浏览专用

1 总　　则

1.0.1　为贯彻国家有关应对气候变化和节能减排的方针政策，规范建筑碳排放计算方法，节约资源，保护环境，制定本标准。

1.0.2　本标准适用于新建、扩建和改建的民用建筑的运行、建造及拆除、建材生产及运输阶段的碳排放计算。

1.0.3　建筑碳排放计算除应符合本标准外，尚应符合国家现行有关标准的规定。

住房城乡建设部信息公开
浏览专用

2 术语和符号

2.1 术　　语

2.1.1 建筑碳排放　building carbon emission

建筑物在与其有关的建材生产及运输、建造及拆除、运行阶段产生的温室气体排放的总和，以二氧化碳当量表示。

2.1.2 计算边界　accounting boundary

与建筑物建材生产及运输、建造及拆除、运行等活动相关的温室气体排放的计算范围。

2.1.3 碳排放因子　carbon emission factor

将能源与材料消耗量与二氧化碳排放相对应的系数，用于量化建筑物不同阶段相关活动的碳排放。

2.1.4 建筑碳汇　carbon sink of buildings

在划定的建筑物项目范围内，绿化、植被从空气中吸收并存储的二氧化碳量。

2.1.5 全球变暖潜值　global warming potential

在固定时间范围内 1kg 物质与 1kg 二氧化碳（CO_2）的脉冲排放引起的时间累积辐射力的比率。

2.2 符　　号

2.2.1 几何尺寸

A——建筑面积；

A_c——太阳集热器面积；

A_i——第 i 个房间照明面积；

A_p——光伏系统光伏面板净面积；

A_w——风机叶片迎风面积；

D——风机叶片直径；

住房城乡建设部信息公开 浏览专用

D_i——第 i 种建材平均运输距离。

2.2.2 碳排放量

C_{CC}——建筑拆除阶段单位建筑面积的碳排放量；

C_p——建筑绿地碳汇系统年减碳量；

C_M——建筑运行阶段单位建筑面积碳排放量；

C_r——建筑使用制冷剂产生的碳排放量；

C_{JC}——建材生产及运输阶段单位建筑面积的碳排放量；

C_{JZ}——建筑建造阶段单位建筑面积的碳排放量；

C_{sc}——建材生产阶段碳排放；

C_{ys}——建材运输过程碳排放。

2.2.3 能源供给、消耗量

E_e——年电梯能耗；

E_i——建筑第 i 类能源年消耗量；

E_{cc}——建筑拆除阶段能源用量；

$E_{cc,i}$——建筑拆除阶段第 i 种能源总用量；

E_{cs}——措施项目总能源用量；

$E_{i,j}$——j 类系统的第 i 类能源消耗量；

E_{jz}——建筑建造阶段总能源用量；

$E_{jz,i}$——建筑建造阶段第 i 种能源总用量；

$E_{jj,i}$——第 i 个项目中，小型施工机具不列入机械台班消耗量，但其消耗的能源列入材料的部分能源用量；

E_{fx}——分部分项工程总能源用量；

EF_i——第 i 类能源的碳排放因子；

E_l——照明系统年能耗；

E_{pv}——光伏系统的年发电量；

$E_{standby}$——电梯待机时能耗；

$ER_{i,j}$——j 类系统消耗由可再生能源系统提供的第 i 类能源量；

E_{wt}——风力发电机组的年发电量；

E_w——生活热水系统年能源消耗；

住房城乡建设部信息公开
浏览专用

I——光伏电池表面的年太阳辐射照度；

J_T——太阳集热器采光面上的年平均太阳辐照量；

M_i——第 i 种主要建材的消耗量；

P——特定能量消耗；

$P_{i,j}$——第 j 日第 i 个房间照明功率密度值；

P_p——应急灯照明功率密度；

$Q_{cc,i}$——第 i 个拆除项目的工程量；

Q_r——生活热水年耗热量；

Q_{rp}——生活热水小时平均耗热量；

Q_s——太阳能系统提供的生活热水热量；

$Q_{s,a}$——太阳能热水系统的年供能量；

$Q_{fx,i}$——分部分项工程中第 i 个项目的工程量；

$Q_{cs,i}$——措施项目中第 i 个项目的工程量；

q_r——热水用水定额；

R_j——第 i 个项目第 j 种施工机械单位台班的能源用量；

T_i——第 i 种建材的运输方式下，单位重量运输距离的碳排放因子；

$T_{i,j}$——第 i 个项目单位工程量第 j 种施工机械台班消耗量；

$T_{A\text{-}i,j}$——第 i 个措施项目单位工程量第 j 种施工机械台班消耗量；

$T_{B\text{-}i,j}$——第 i 个拆除项目单位工程量第 j 种施工机械台班消耗量。

2.2.4 计算系数

APD——年平均能量密度；

$C_R(z)$——依据高度计算的粗糙系数；

EPF——根据典型气象年数据中逐时风速计算出的因子；

F_i——第 i 种主要建材的碳排放因子；

$f_{fx,i}$——分部分项工程中第 i 个项目的能耗系数；

$f_{cs,i}$——措施项目中第 i 个项目的能耗系数；

$f_{cc,i}$——第 i 个拆除项目每计量单位的能耗系数；

住房城乡建设部信息公开
浏览专用

GWP_r——制冷剂 r 的全球变暖潜值；
K_E——光伏电池的转换效率；
K_S——光伏系统的损失效率；
m——用水计算单位数；
K_{WT}——风力发电机组的转换效率；
η_r——生活热水输配效率；
η_w——生活热水系统热源年平均效率；
η_{cd}——基于总面积的集热器平均集热效率；
η_L——管路和储热装置的热损失率。

2.2.5 风速、温度、密度和时间

m_r——制冷剂充注量；
$t_{i,j}$——第 j 日第 i 个房间照明时间；
T——年生活热水使用小时数；
t_a——电梯年平均运行小时数；
t_r——设计热水温度；
t_l——设计冷水温度；
t_s——电梯年平均待机小时数；
V——电梯速度；
V_0——年可利用平均风速；
V_i——逐时风速；
W——电梯额定载重量；
y——建筑设计寿命；
y_e——设备使用寿命；
ρ——空气密度；
ρ_r——热水密度。

2.2.6 其他

K_R——场地因子；
r——制冷剂类型；
z_0——地表粗糙系数。

住房城乡建设部信息公开
浏览专用

3 基 本 规 定

3.0.1 建筑物碳排放计算应以单栋建筑或建筑群为计算对象。

3.0.2 建筑碳排放计算方法可用于建筑设计阶段对碳排放量进行计算，或在建筑物建造后对碳排放量进行核算。

3.0.3 建筑物碳排放计算应根据不同需求按阶段进行计算，并可将分段计算结果累计为建筑全生命期碳排放。

3.0.4 碳排放计算应包含《IPCC 国家温室气体清单指南》中列出的各类温室气体。

3.0.5 建筑运行、建造及拆除阶段中因电力消耗造成的碳排放计算，应采用由国家相关机构公布的区域电网平均碳排放因子。

3.0.6 建筑碳排放量应按本标准提供的方法和数据进行计算，宜采用基于本标准计算方法和数据开发的建筑碳排放计算软件计算。

住房城乡建设部信息公开
浏览专用

4 运行阶段碳排放计算

4.1 一 般 规 定

4.1.1 建筑运行阶段碳排放计算范围应包括暖通空调、生活热水、照明及电梯、可再生能源、建筑碳汇系统在建筑运行期间的碳排放量。

4.1.2 碳排放计算中采用的建筑设计寿命应与设计文件一致，当设计文件不能提供时，应按50年计算。

4.1.3 建筑物碳排放的计算范围应为建设工程规划许可证范围内能源消耗产生的碳排放量和可再生能源及碳汇系统的减碳量。

4.1.4 建筑运行阶段碳排放量应根据各系统不同类型能源消耗量和不同类型能源的碳排放因子确定，建筑运行阶段单位建筑面积的总碳排放量(C_M)应按下列公式计算：

$$C_M = \frac{\left[\sum_{i=1}^{n}(E_i EF_i) - C_p\right]y}{A} \qquad (4.1.4\text{-}1)$$

$$E_i = \sum_{j=1}^{n}(E_{i,j} - ER_{i,j}) \qquad (4.1.4\text{-}2)$$

式中：C_M ——建筑运行阶段单位建筑面积碳排放量($kgCO_2/m^2$)；

E_i ——建筑第 i 类能源年消耗量(单位/a)；

EF_i ——第 i 类能源的碳排放因子，按本标准附录 A 取值；

$E_{i,j}$ ——j 类系统的第 i 类能源消耗量(单位/a)；

$ER_{i,j}$ ——j 类系统消耗由可再生能源系统提供的第 i 类能源量(单位/a)；

i ——建筑消耗终端能源类型，包括电力、燃气、石油、市政热力等；

住房城乡建设部信息公开
浏览专用

j——建筑用能系统类型，包括供暖空调、照明、生活热水系统等；

C_p——建筑绿地碳汇系统年减碳量($kgCO_2/a$)；

y——建筑设计寿命(a)；

A——建筑面积(m^2)。

4.2 暖通空调系统

4.2.1 暖通空调系统能耗应包括冷源能耗、热源能耗、输配系统及末端空气处理设备能耗。

4.2.2 暖通空调系统能耗计算方法应符合下列规定：

1 应采用月平均方法计算年累计冷负荷和累计热负荷；

2 应分别设置工作日和节假日室内人员数量、照明功率、设备功率、室内设定温度、供暖和空调系统运行时间；

3 应根据负荷计算结果和室内环境参数计算供暖和供冷起止时间；

4 应反映建筑外围护结构热惰性对负荷的影响；

5 负荷计算时应能够计算不少于10个建筑分区；

6 应计算暖通空调系统间歇运行对负荷计算结果的影响；

7 应考虑能源系统形式、效率、部分负荷特性对能耗的影响；

8 计算结果应包括负荷计算结果、按能源类型输出系统能耗计算结果；

9 建筑运行参数可参照本标准附录B的建筑物运行特征确定。

4.2.3 建筑碳排放计算模型中建筑分区应考虑建筑物理分隔、建筑区域功能、为分区提供服务的暖通空调系统、区域内采光(通过外窗或天窗)情况。

4.2.4 年供暖(供冷)负荷应包括围护结构的热损失和处理新风的热(冷)需求；处理新风的热(冷)需求应扣除从排风中回收的热量(冷量)。

住房城乡建设部信息公开 浏览专用

4.2.5 建筑碳排放计算中建筑室内环境计算参数应与设计参数一致，并应符合国家现行相关标准的要求。

4.2.6 建筑碳排放计算气象参数的选取应符合现行行业标准《建筑节能气象参数标准》JGJ/T 346 的规定。

4.2.7 建筑碳排放计算应定义建筑围护结构，围护结构的热工性能及构造做法应与设计文件一致。

4.2.8 建筑碳排放计算中应分别计算建筑累积冷负荷和累积热负荷。

4.2.9 建筑碳排放计算中的累积冷热负荷应根据下列内容确定：

1 通过围护结构传入的热量；

2 透过透明围护结构进入的太阳辐射热量；

3 人体散热量；

4 照明散热量；

5 设备、器具、管道及其他内部热源的散热量；

6 食品或物料的散热量；

7 渗透空气带入的热量；

8 伴随各种散湿过程产生的潜热量。

4.2.10 建筑碳排放计算时应计算气密性、风压和热压的作用、人员密度、新风量、热回收系统效率对通风负荷的影响。

4.2.11 建筑累积冷负荷和热负荷应根据建筑物分区的空调系统计算，同一暖通空调系统服务的建筑物分区的冷负荷和热负荷应分别进行求和计算。

4.2.12 根据建筑年供冷负荷和年供暖负荷计算暖通空调系统终端能耗时应根据下列影响因素分别进行计算：

1 供冷供暖系统类型；

2 冷源和热源的效率；

3 泵与风机的能耗情况；

4 末端类型；

5 系统控制策略；

6 系统运行内部冷热抵消等情况；

住房城乡建设部信息公开
浏览专用

7 暖通空调系统能量输送介质的影响；

8 冷热回收措施。

4.2.13 暖通空调系统中由于制冷剂使用而产生的温室气体排放，应按下式计算：

$$C_r = \frac{m_r}{y_e} GWP_r / 1000 \tag{4.2.13}$$

式中：C_r ——建筑使用制冷剂产生的碳排放量(tCO_2e/a)；

r——制冷剂类型；

m_r ——设备的制冷剂充注量(kg/台)；

y_e——设备使用寿命(a)；

GWP_r ——制冷剂 r 的全球变暖潜值。

4.2.14 建筑物碳排放计算采用的冷热源及相关用能设备的性能参数应与设计文件一致。

4.2.15 建筑冷热源的能耗计算应计入负载、输送过程和末端的冷热量损失等因素的影响。

4.2.16 输送系统的能耗计算应计入水泵与风机的效率、运行时长、实际工作状态点的负载率、变频等因素的影响。

4.3 生活热水系统

4.3.1 建筑物生活热水年耗热量的计算应根据建筑物的实际运行情况，并应按下列公式计算：

$$Q_{rp} = 4.187 \frac{mq_r C_r (t_r - t_l) \rho_r}{1000} \tag{4.3.1-1}$$

$$Q_r = TQ_{rp} \tag{4.3.1-2}$$

式中：Q_r ——生活热水年耗热量(kWh/a)；

Q_{rp} ——生活热水小时平均耗热量(kW/h)；

T ——年生活热水使用小时数(h)；

m——用水计算单位数(人数或床位数，取其一)；

q_r ——热水用水定额(L/人)，按现行国家标准《民用建筑节水设计标准》GB 50555 确定；

住房城乡建设部信息公开 浏览专用

ρ_r ——热水密度(kg/L);

t_r ——设计热水温度(℃);

t_l ——设计冷水温度(℃)。

4.3.2 建筑生活热水系统能耗应按下式计算，且计算采用的生活热水系统的热源效率应与设计文件一致。

$$E_w = \frac{\frac{Q_r}{\eta_r} - Q_s}{\eta_w} \qquad (4.3.2)$$

式中：E_w ——生活热水系统年能源消耗(kWh/a);

Q_r ——生活热水年耗热量(kWh/a);

Q_s ——太阳能系统提供的生活热水热量(kWh/a);

η_r ——生活热水输配效率，包括热水系统的输配能耗、管道热损失、生活热水二次循环及储存的热损失(%);

η_w ——生活热水系统热源年平均效率(%)。

4.4 照明及电梯系统

4.4.1 建筑碳排放计算采用的照明功率密度值应同设计文件一致。

4.4.2 照明系统能耗计算应将自然采光、控制方式和使用习惯等因素影响计入。

4.4.3 照明系统无光电自动控制系统时，其能耗计算可按下式计算：

$$E_l = \frac{\sum_{j=1}^{365} \sum_i P_{i,j} A_i t_{i,j} + 24 P_p A}{1000} \qquad (4.4.3)$$

式中：E_l ——照明系统年能耗(kWh/a);

$P_{i,j}$ ——第 j 日第 i 个房间照明功率密度值(W/m^2);

A_i ——第 i 个房间照明面积(m^2);

$t_{i,j}$ ——第 j 日第 i 个房间照明时间(h);

P_p ——应急灯照明功率密度(W/m^2);

住房城乡建设部信息公开 浏览专用

A——建筑面积(m^2)。

4.4.4 电梯系统能耗应按下式计算，且计算中采用的电梯速度、额定载重量、特定能量消耗等参数应与设计文件或产品铭牌一致。

$$E_e = \frac{3.6Pt_aVW + E_{standby}t_s}{1000} \tag{4.4.4}$$

式中：E_e ——年电梯能耗(kWh/a)；

P ——特定能量消耗(mWh/kgm)；

t_a ——电梯年平均运行小时数(h)；

V ——电梯速度(m/s)；

W ——电梯额定载重量(kg)；

$E_{standby}$ ——电梯待机时能耗(W)；

t_s ——电梯年平均待机小时数(h)。

4.5 可再生能源系统

4.5.1 可再生能源系统应包括太阳能生活热水系统、光伏系统、地源热泵系统和风力发电系统。

4.5.2 太阳能热水系统提供能量可按下式计算：

$$Q_{s,a} = \frac{A_cJ_T(1-\eta_L)\eta_{cd}}{3.6} \tag{4.5.2}$$

式中：$Q_{s,a}$ ——太阳能热水系统的年供能量(kWh)；

A_c ——太阳集热器面积(m^2)；

J_T ——太阳集热器采光面上的年平均太阳辐照量(MJ/m^2)；

η_{cd} ——基于总面积的集热器平均集热效率(%)；

η_L ——管路和储热装置的热损失率(%)。

4.5.3 太阳能热水系统提供的能量不应计入生活热水的耗能量。

4.5.4 地源热泵系统的节能量应计算在暖通空调系统能耗内。

4.5.5 光伏系统的年发电量可按下式计算：

住房城乡建设部信息公开
浏览专用

$$E_{pv} = IK_{E}(1 - K_{S})A_{p} \qquad (4.5.5)$$

式中：E_{pv} ——光伏系统的年发电量(kWh)；

I——光伏电池表面的年太阳辐射照度(kWh/m^2)；

K_E ——光伏电池的转换效率(%)；

K_S ——光伏系统的损失效率(%)；

A_p ——光伏系统光伏面板净面积(m^2)。

4.5.6 风力发电机组年发电量可按下列公式计算：

$$E_{wt} = 0.5\rho C_{R}(z)V_{0}^{3}A_{w}\rho\frac{K_{WT}}{1000} \qquad (4.5.6\text{-}1)$$

$$C_{R}(z) = K_{R}\ln(z/z_{0}) \qquad (4.5.6\text{-}2)$$

$$A_{w} = 5D^{2}/4 \qquad (4.5.6\text{-}3)$$

$$EPF = \frac{APD}{0.5\rho V_{0}^{3}} \qquad (4.5.6\text{-}4)$$

$$APD = \frac{\sum_{i=1}^{8760} 0.50\rho V_{i}^{3}}{8760} \qquad (4.5.6\text{-}5)$$

式中：E_{wt} ——风力发电机组的年发电量(kWh)；

ρ——空气密度，取 1.225kg/m^3；

$C_R(z)$——依据高度计算的粗糙系数；

K_R ——场地因子；

z_0 ——地表粗糙系数；

V_0 ——年可利用平均风速(m/s)；

A_w——风机叶片迎风面积(m^2)；

D——风机叶片直径(m)；

EPF——根据典型气象年数据中逐时风速计算出的因子；

APD——年平均能量密度(W/m^2)；

V_i ——逐时风速(m/s)；

K_{WT} ——风力发电机组的转换效率。

住房城乡建设部信息公开
浏览专用

5 建造及拆除阶段碳排放计算

5.1 一 般 规 定

5.1.1 建筑建造阶段的碳排放应包括完成各分部分项工程施工产生的碳排放和各项措施项目实施过程产生的碳排放。

5.1.2 建筑拆除阶段的碳排放应包括人工拆除和使用小型机具机械拆除使用的机械设备消耗的各种能源动力产生的碳排放。

5.1.3 建筑建造和拆除阶段的碳排放的计算边界应符合下列规定：

1 建造阶段碳排放计算时间边界应从项目开工起至项目竣工验收止，拆除阶段碳排放计算时间边界应从拆除起至拆除肢解并从楼层运出止；

2 建筑施工场地区域内的机械设备、小型机具、临时设施等使用过程中消耗的能源产生的碳排放应计入；

3 现场搅拌的混凝土和砂浆、现场制作的构件和部品，其产生的碳排放应计入；

4 建造阶段使用的办公用房、生活用房和材料库房等临时设施的施工和拆除可不计入。

5.2 建 筑 建 造

5.2.1 建筑建造阶段的碳排放量应按下式计算：

$$C_{JZ} = \frac{\sum_{i=1}^{n} E_{jz,i} EF_i}{A} \qquad (5.2.1)$$

式中：C_{JZ}——建筑建造阶段单位建筑面积的碳排放量（$kgCO_2/m^2$）；

$E_{jz,i}$——建筑建造阶段第 i 种能源总用量（kWh 或 kg）；

住房城乡建设部信息公开
浏览专用

EF_i——第 i 类能源的碳排放因子($kgCO_2/kWh$ 或 $kgCO_2/kg$)，按本标准附录 A 确定；

A——建筑面积(m^2)。

5.2.2 建造阶段的能源总用量宜采用施工工序能耗估算法计算。

5.2.3 施工工序能耗估算法的能源用量应按下式计算：

$$E_{jz}=E_{fx}+E_{cs} \tag{5.2.3}$$

式中：E_{jz}——建筑建造阶段总能源用量(kWh 或 kg)；

E_{fx}——分部分项工程总能源用量(kWh 或 kg)；

E_{cs}——措施项目总能源用量(kWh 或 kg)。

5.2.4 分部分项工程能源用量应按下列公式计算：

$$E_{fx}=\sum_{i=1}^{n}Q_{fx,i}f_{fx,i} \tag{5.2.4-1}$$

$$f_{fx,i}=\sum_{j=1}^{m}T_{i,j}R_j+E_{jj,i} \tag{5.2.4-2}$$

式中：$Q_{fx,i}$——分部分项工程中第 i 个项目的工程量；

$f_{fx,i}$——分部分项工程中第 i 个项目的能耗系数(kWh/工程量计量单位)；

$T_{i,j}$——第 i 个项目单位工程量第 j 种施工机械台班消耗量(台班)；

R_j——第 i 个项目第 j 种施工机械单位台班的能源用量(kWh/台班)，按本标准附录 C 确定，当有经验数据时，可按经验数据确定；

$E_{jj,i}$——第 i 个项目中，小型施工机具不列入机械台班消耗量，但其消耗的能源列入材料的部分能源用量(kWh)；

i——分部分项工程中项目序号；

j——施工机械序号。

5.2.5 措施项目的能耗计算应符合下列规定：

1 脚手架、模板及支架、垂直运输、建筑物超高等可计算工程量的措施项目，其能耗应按下列公式计算：

住房城乡建设部信息公开
浏览专用

$$E_{cs}=\sum_{i=1}^{n}Q_{cs,i}f_{cs,i} \tag{5.2.5-1}$$

$$f_{cs,i}=\sum_{j=1}^{m}T_{A\text{-}i,j}R_j \tag{5.2.5-2}$$

式中：$Q_{cs,i}$——措施项目中第 i 个项目的工程量；

$f_{cs,i}$——措施项目中第 i 个项目的能耗系数(kWh/工程量计量单位)；

$T_{A\text{-}i,j}$——第 i 个措施项目单位工程量第 j 种施工机械台班消耗量(台班)；

R_j——第 i 个项目第 j 种施工机械单位台班的能源用量(kWh/台班)，按本标准附录 C 对应的机械类别确定；

i——措施项目序号；

j——施工机械序号。

2 施工降排水应包括成井和使用两个阶段，其能源消耗应根据项目降排水专项方案计算。

3 施工临时设施消耗的能源应根据施工企业编制的临时设施布置方案和工期计算确定。

5.3 建 筑 拆 除

5.3.1 建筑拆除阶段的单位建筑面积的碳排放量应按下式计算：

$$C_{CC}=\frac{\sum_{i=1}^{n}E_{cc,i}EF_i}{A} \tag{5.3.1}$$

式中：C_{CC}——建筑拆除阶段单位建筑面积的碳排放量($kgCO_2/m^2$)；

$E_{cc,i}$——建筑拆除阶段第 i 种能源总用量(kWh 或 kg)；

EF_i——第 i 类能源的碳排放因子($kgCO_2$/kWh)，按本标准附录 A 确定；

住房城乡建设部信息公开
浏览专用

A——建筑面积(m^2)。

5.3.2 建筑物人工拆除和机械拆除阶段的能源用量应按下列公式计算：

$$E_{cc} = \sum_{i=1}^{n} Q_{cc,i} f_{cc,i} \tag{5.3.2-1}$$

$$f_{cc,i} = \sum_{j=1}^{m} T_{B\text{-}i,j} R_j + E_{jj,i} \tag{5.3.2-2}$$

式中：E_{cc}——建筑拆除阶段能源用量(kWh 或 kg)；

$Q_{cc,i}$——第 i 个拆除项目的工程量；

$f_{cc,i}$——第 i 个拆除项目每计量单位的能耗系数(kWh/工程量计量单位或 kg/工程量计量单位)；

$T_{B\text{-}i,j}$——第 i 个拆除项目单位工程量第 j 种施工机械台班消耗量；

R_j——第 i 个项目第 j 种施工机械单位台班的能源用量；

i——拆除工程中项目序号；

j——施工机械序号。

5.3.3 建筑物爆破拆除、静力破损拆除及机械整体性拆除的能源用量应根据拆除专项方案确定。

5.3.4 建筑物拆除后的垃圾外运产生的能源用量应按本标准第 6.3 节的规定计算。

住房城乡建设部信息公开
浏览专用

6　建材生产及运输阶段碳排放计算

6.1　一 般 规 定

6.1.1　建材碳排放应包含建材生产阶段及运输阶段的碳排放，并应按现行国家标准《环境管理　生命周期评价　原则与框架》GB/T 24040、《环境管理　生命周期评价　要求与指南》GB/T 24044 计算。

6.1.2　建材生产及运输阶段的碳排放应为建材生产阶段碳排放与建材运输阶段碳排放之和，并应按下式计算：

$$C_{JC}=\frac{C_{sc}+C_{ys}}{A} \tag{6.1.2}$$

式中：C_{JC}——建材生产及运输阶段单位建筑面积的碳排放量（kg CO_2e/m^2）；

C_{sc}——建材生产阶段碳排放（kg CO_2e）；

C_{ys}——建材运输过程碳排放（kg CO_2e）；

A——建筑面积（m^2）。

6.1.3　建材生产及运输阶段碳排放计算应包括建筑主体结构材料、建筑围护结构材料、建筑构件和部品等，纳入计算的主要建筑材料的确定应符合下列规定：

1　所选主要建筑材料的总重量不应低于建筑中所耗建材总重量的 95%；

2　当符合本条第 1 款的规定时，重量比小于 0.1%的建筑材料可不计算。

6.2　建 材 生 产

6.2.1　建材生产阶段碳排放应按下式计算：

$$C_{sc}=\sum_{i=1}^{n}M_iF_i \tag{6.2.1}$$

住房城乡建设部信息公开
浏览专用

式中：C_{sc}——建材生产阶段碳排放($kg\ CO_2e$)；

M_i——第 i 种主要建材的消耗量；

F_i——第 i 种主要建材的碳排放因子($kg\ CO_2e$/单位建材数量)，按本标准附录D取值。

6.2.2 建筑的主要建材消耗量(M_i)应通过查询设计图纸、采购清单等工程建设相关技术资料确定。

6.2.3 建材生产阶段的碳排放因子(F_i)应包括下列内容：

1 建筑材料生产涉及原材料的开采、生产过程的碳排放；

2 建筑材料生产涉及能源的开采、生产过程的碳排放；

3 建筑材料生产涉及原材料、能源的运输过程的碳排放；

4 建筑材料生产过程的直接碳排放。

6.2.4 建材生产阶段的碳排放因子宜选用经第三方审核的建材碳足迹数据。当无第三方提供时，缺省值可按本标准附录D执行。

6.2.5 建材生产时，当使用低价值废料作为原料时，可忽略其上游过程的碳过程。当使用其他再生原料时，应按其所替代的初生原料的碳排放的50%计算；建筑建造和拆除阶段产生的可再生建筑废料，可按其可替代的初生原料的碳排放的50%计算，并应从建筑碳排放中扣除。

6.3 建材运输

6.3.1 建材运输阶段碳排放应按下式计算：

$$C_{ys}=\sum_{i=1}^{n}M_iD_iT_i \qquad (6.3.1)$$

式中：C_{ys}——建材运输过程碳排放($kg\ CO_2e$)；

M_i——第 i 种主要建材的消耗量(t)；

D_i——第 i 种建材平均运输距离(km)；

T_i——第 i 种建材的运输方式下，单位重量运输距离的碳排放因子[$kg\ CO_2e/(t\cdot km)$]。

6.3.2 主要建材的运输距离宜优先采用实际的建材运输距离。

住房城乡建设部信息公开 浏览专用

当建材实际运输距离未知时，可按本标准附录 E 中的默认值取值。

6.3.3 建材运输阶段的碳排放因子（T_i）应包含建材从生产地到施工现场的运输过程的直接碳排放和运输过程所耗能源的生产过程的碳排放。建材运输阶段的碳排放因子（T_i）可按本标准附录 E 的缺省值取值。

住房城乡建设部信息公开
浏览专用

附录A　主要能源碳排放因子

A.0.1　化石燃料碳排放因子应按表A.0.1选取。

表A.0.1　化石燃料碳排放因子

分类	燃料类型	单位热值含碳量(tC/TJ)	碳氧化率(%)	单位热值CO_2排放因子(tCO_2/TJ)
固体燃料	无烟煤	27.4	0.94	94.44
	烟煤	26.1	0.93	89.00
	褐煤	28.0	0.96	98.56
	炼焦煤	25.4	0.98	91.27
	型煤	33.6	0.90	110.88
	焦炭	29.5	0.93	100.60
	其他焦化产品	29.5	0.93	100.60
液体燃料	原油	20.1	0.98	72.23
	燃料油	21.1	0.98	75.82
	汽油	18.9	0.98	67.91
	柴油	20.2	0.98	72.59
	喷气煤油	19.5	0.98	70.07
	一般煤油	19.6	0.98	70.43
	NGL天然气凝液	17.2	0.98	61.81
	LPG液化石油气	17.2	0.98	61.81
	炼厂干气	18.2	0.98	65.40
	石脑油	20.0	0.98	71.87
	沥青	22.0	0.98	79.05
	润滑油	20.0	0.98	71.87

住房城乡建设部信息公开
浏览专用

续表 A.0.1

分类	燃料类型	单位热值含碳量（tC/TJ）	碳氧化率（%）	单位热值 CO_2 排放因子（tCO_2/TJ）
液体燃料	石油焦	27.5	0.98	98.82
	石化原料油	20.0	0.98	71.87
	其他油品	20.0	0.98	71.87
气体燃料	天然气	15.3	0.99	55.54

A.0.2 其他能源碳排放因子应按表 A.0.2 选取。

表 A.0.2 其他能源碳排放因子

能源类型		缺省碳含量（tC/TJ）	缺省氧化因子	有效 CO_2 排放因子（tCO_2/TJ）		
				缺省值	95%置信区间	
					较低	较高
城市废弃物（非生物量比例）		25.0	1	91.7	73.3	121
工业废弃物		39.0	1	143.0	110.0	183.0
废油		20.0	1	73.3	72.2	74.4
泥炭		28.9	1	106.0	100.0	108.0
固体生物燃料	木材/木材废弃物	30.5	1	112.0	95.0	132.0
	亚硫酸盐废液（黑液）	26.0	1	95.3	80.7	110.0
	木炭	30.5	1	112.0	95.0	132.0
	其他主要固体生物燃料	27.3	1	100.0	84.7	117.0
液体生物燃料	生物汽油	19.3	1	70.8	59.8	84.3
	生物柴油	19.3	1	70.8	59.8	84.3
	其他液体生物燃料	21.7	1	79.6	67.1	95.3
气体生物燃料	填埋气体	14.9	1	54.6	46.2	66.0
	污泥气体	14.9	1	54.6	46.2	66.0
	其他生物气体	14.9	1	54.6	46.2	66.0
其他非化石燃料	城市废弃物（生物量比例）	27.3	1	100.0	84.7	117.0

住房城乡建设部信息公开 浏览专用

附录B 建筑物运行特征

B.0.1 计算建筑物碳排放时建筑物运行特征应符合表B.0.1的规定。

表B.0.1 建筑物运行特征

建筑类型	房间类型	是否空调	是否供暖	夏季设计温度(℃)	夏季设计相对湿度(%)	冬季设计温度(℃)	冬季设计相对湿度(%)	设计照度(lux)	设备能耗密度(W/m^2)	月照明小时数(h)	照明功率密度(W/m^2)	人均新风量[m^3/(h·人)]
居住建筑	起居室	是	是	26	65	18	—	100	9.3	165	6	70
	卧室	是	是	26	65	18	—	75	12.7	135	6	20
	餐厅	是	是	26	65	18	—	150	9.3	75	6	20
	厨房	否	是	30	70	15	—	100	48.2	96	6	20
	洗手间	否	是	26	70	18	—	100	0	165	6	20
	储物间	否	是	26	65	5	—	0	0	0	0	20
	车库	否	是	26	65	5	—	30	0	30	2	20
公共建筑	办公室	是	是	26	65	20	—	500	13	294	18	30
	密集办公室	是	是	26	65	20	—	300	20	294	11	30

住房城乡建设部信息公开 浏览专用

续表 B.0.1

建筑类型	房间类型	是否空调	是否供暖	夏季设计温度（℃）	夏季设计相对湿度（%）	冬季设计温度（℃）	冬季设计相对湿度（%）	设计照度（lux）	设备能耗密度（W/m^2）	月照明小时数（h）	照明功率密度（W/m^2）	人均新风量[m^3/(h·人)]
公共建筑	会议室	是	是	26	65	20	—	300	5	420	11	30
	大堂门厅	是	是	26	65	20	—	300	0	585	15	20
	休息室	是	是	25	65	18	—	300	0	420	11	30
	设备用房	否	是	26	65	18	—	150	0	0	5	30
	库房	否	是	26	65	18	—	0	0	0	0	0
	车库	否	是	26	65	18	—	75	30	294	5	—
	酒店客房(三星以下)	是	是	26	65	18	—	150	20	207	15	20
	酒店客房(三星)	是	是	26	65	20	—	150	13	207	15	30
	酒店客房(四星)	是	是	25	60	21	—	150	13	207	15	40
	酒店客房(五星)	是	是	24	60	22	—	150	13	207	15	50
	多功能厅	是	是	26	65	20	—	300	5	420	18	30
	一般商店、超市	是	是	27	65	20	—	300	13	390	12	20
	高档商店	是	是	27	65	20	—	500	13	390	19	20
	中餐厅	是	是	25	60	20	—	200	0	393	13	20

住房城乡建设部信息公开
浏览专用

续表 B.0.1

建筑类型	房间类型	是否空调	是否供暖	夏季设计温度（℃）	夏季设计相对湿度（%）	冬季设计温度（℃）	冬季设计相对湿度（%）	设计照度（lux）	设备能耗密度（W/m^2）	月照明小时数（h）	照明功率密度（W/m^2）	人均新风量[m^3/(h·人)]
公共建筑	西餐厅	是	是	25	60	20	—	100	0	393	9	20
	火锅店	是	是	25	60	18	—	200	0	168	13	20
	快餐店	是	是	25	60	20	—	200	0	393	13	20
	酒吧、茶座	是	是	25	60	20	—	100	0	393	9	20
	厨房	否	是	28	65	18	—	200	0	393	13	—
	游泳池	是	是	30	75	26	—	300	0	168	18	25
	健身房	是	是	25	60	18	—	200	0	168	11	25
	保龄球房	是	是	25	60	18	—	300	0	288	18	25
	台球房	是	是	25	60	18	—	300	0	288	18	25
	教室	是	是	26	60	20	—	300	10	150	10	17
	阅览室	是	是	26	60	20	—	300	10	150	10	17
	电脑机房	是	是	25	60	18	—	300	40	390	11	30

住房城乡建设部信息公开 浏览专用

续表 B.0.1

建筑类型	房间类型	是否空调	是否供暖	夏季设计温度（℃）	夏季设计相对湿度（%）	冬季设计温度（℃）	冬季设计相对湿度（%）	设计照度（lux）	设备能耗密度（W/m^2）	月照明小时数（h）	照明功率密度（W/m^2）	人均新风量[m^3/(h·人)]
公共建筑	影剧院	是	是	28	65	20	—	200	0	480	11	20
	舞台	是	是	28	65	20	—	300	40	480	11	40
	舞厅	是	是	25	60	18	—	300	30	258	11	30
	棋牌室	是	是	27	60	20	—	200	0	132	11	20
	展览厅	是	是	27	60	18	—	300	20	300	11	20
	病房	是	是	27	60	22	—	100	0	129	5	50
	手术室	是	是	25	60	22	—	750	0	381	20	60
	候诊室	是	是	27	55	20	—	300	0	468	5	30
	门诊办公室	是	是	26	65	22	—	300	0	468	5	30
	婴儿室	是	是	27	60	25	—	300	0	315	5	60
	药品储存库	是	是	16	60	16	—	300	0	615	5	0
	档案库房	是	是	24	60	14	—	200	0	540	5	0
	美容院	是	是	27	60	22	—	750	5	345	15	35

住房城乡建设部信息公开 浏览专用

附录C 常用施工机械台班能源用量

C.0.1 常用施工机械的单位台班的能源消耗量可按表C.0.1选用。

表C.0.1 常用施工机械台班能源用量

序号	机械名称	性能规格		能源用量 汽油(kg)	能源用量 柴油(kg)	能源用量 电(kWh)
1	履带式推土机	功率	75kW	—	56.50	—
2			105kW	—	60.80	—
3			135kW	—	66.80	—
4	履带式单斗液压挖掘机	斗容量	$0.6m^3$	—	33.68	—
5			$1m^3$	—	63.00	—
6	轮胎式装载机	斗容量	$1m^3$	—	52.73	—
7			$1.5m^3$	—	58.75	—
8	钢轮内燃压路机	工作质量	8t	—	19.79	—
9			15t	—	42.95	—
10	电动夯实机	夯击能量	250N·m	—	—	16.6
11	强夯机械	夯击能量	1200kN·m	—	32.75	—
12			2000kN·m	—	42.76	—
13			3000kN·m	—	55.27	—
14			4000kN·m	—	58.22	—
15			5000kN·m	—	81.44	—
16	锚杆钻孔机	锚杆直径	32mm	—	69.72	—
17	履带式柴油打桩机	冲击质量	2.5t	—	44.37	—
18			3.5t	—	47.94	—
19			5t	—	53.93	—
20			7t	—	57.40	—
21			8t	—	59.14	—

住房城乡建设部信息公开
浏览专用

续表 C.0.1

序号	机械名称	性能规格		能源用量 汽油(kg)	能源用量 柴油(kg)	能源用量 电(kWh)
22	轨道式柴油打桩机	冲击质量	3.5t	—	56.90	—
23			4t	—	61.70	—
24	步履式柴油打桩机	功率	60kW	—	—	336.87
25	振动沉拔桩机	激振力	300kN	—	17.43	—
26			400kN	—	24.90	—
27	静力压桩机	压力	900kN	—	—	91.81
28			2000kN	—	77.76	—
29			3000kN	—	85.26	—
30			4000kN	—	96.25	—
31	汽车式钻机	孔径	1000mm	—	48.80	—
32	回旋钻机	孔径	800mm	—	—	142.5
33			1000mm	—	—	163.72
34			1500mm	—	—	190.72
35	螺旋钻机	孔径	600mm	—	—	181.27
36	冲孔钻机	孔径	1000mm	—	—	40.00
37	履带式旋挖钻机	孔径	1000mm	—	146.56	—
38			1500mm	—	164.32	—
39			2000mm	—	172.32	—
40	三轴搅拌桩基	轴径	650mm	—	—	126.42
41			850mm	—	—	156.42
42	电动灌浆机			—	—	16.20
43	履带式起重机	提升质量	5t	—	18.42	—
44			10t	—	23.56	—
45			15t	—	29.52	—
46			20t	—	30.75	—

住房城乡建设部信息公开
浏览专用

续表 C.0.1

序号	机械名称	性能规格		能源用量		
				汽油 (kg)	柴油 (kg)	电 (kWh)
47	履带式起重机	提升质量	25t	—	36.98	—
48			30t	—	41.61	—
49			40t	—	42.46	—
50			50t	—	44.03	—
51			60t	—	47.17	—
52	轮胎式起重机	提升质量	25t	—	46.26	—
53			40t	—	62.76	—
54			50t	—	64.76	—
55	汽车式起重机	提升质量	8t	—	28.43	—
56			12t	—	30.55	—
57			16t	—	35.85	—
58			20t	—	38.41	—
59			30t	—	42.14	—
60			40t	—	48.52	—
61	叉式起重机	提升质量	3 t	26.46	—	—
62	自升式塔式起重机	提升质量	400t	—	—	164.31
63			60t	—	—	166.29
64			800t	—	—	169.16
65			1000t	—	—	170.02
66			2500t	—	—	266.04
67			3000t	—	—	295.60
68	门式起重机	提升质量	10t	—	—	88.29
69	载重汽车	装载质量	4t	25.48	—	—
70			6t	—	33.24	—
71			8t	—	35.49	—
72			12t	—	46.27	—
73			15t	—	56.74	—
74			20t		62.56	—

住房城乡建设部信息公开
浏览专用

续表 C.0.1

序号	机械名称	性能规格		能源用量 汽油（kg）	能源用量 柴油（kg）	能源用量 电（kWh）
75	自卸汽车	装载质量	5t	31.34	—	—
76			15t	—	52.93	—
77	平板拖车组	装载质量	20t	—	45.39	—
78	机动翻斗车	装载质量	1t	—	6.03	—
79	洒水车	灌容量	4000L	30.21	—	—
80	泥浆罐车	灌容量	5000L	31.57	—	—
81	电动单筒快速卷扬机	牵引力	10kN	—	—	32.90
82	电动单筒慢速卷扬机	牵引力	10kN	—	—	126.00
83			30kN	—	—	28.76
84	单笼施工电梯	提升质量 1t	提升高度 75m	—	—	42.32
85			提升高度 100m	—	—	45.66
86	双笼施工电梯	提升质量 2t	提升高度 100m	—	—	81.86
87			提升高度 200m	—	—	159.94
88	平台作业升降车	提升高度	20m	—	48.25	
89	涡桨式混凝土搅拌机	出料容量	250L	—	—	34.10
90			500L	—	—	107.71
91	双锥反转出料混凝土搅拌机	出料容量	500L	—	—	55.04
92	混凝土输送泵	输送量	$45m^3/h$	—	—	243.46
93			$75m^3/h$	—	—	367.96
94	混凝土湿喷机	生产率	$5m^3/h$	—	—	15.40
95	灰浆搅拌机	拌筒容量	200L	—	—	8.61
96	干混砂浆罐式搅拌机	公称储量	20000L	—	—	28.51

住房城乡建设部信息公开
浏览专用

续表 C.0.1

序号	机械名称	性能规格		能源用量		
				汽油 (kg)	柴油 (kg)	电 (kWh)
97	挤压式灰浆输送泵	输送量	$3m^3/h$	—	—	23.70
98	偏心振动筛	生产率	$16m^3/h$	—	—	28.60
99	混凝土抹平机	功率	5.5kW	—	—	23.14
100	钢筋切断机	直径	40mm	—	—	32.10
101	钢筋弯曲机	直径	40mm	—	—	12.80
102	预应力钢筋拉伸机	拉伸力	650kN	—	—	17.25
103			900kN	—	—	29.16
104	木工圆锯机	直径	500mm	—	—	24.00
105	木工平刨床	刨削宽度	500mm	—	—	12.90
106	木工三面压刨床	刨削宽度	400mm	—	—	52.40
107	木工榫机	榫头长度	160mm	—	—	27.00
108	木工打眼机	榫槽宽度	—	—	—	4.7
109	普通车床	工件直径×工件长度	400mm×2000mm	—	—	22.77
110	摇臂钻床	钻孔直径	50mm	—	—	9.87
111			63mm	—	—	17.07
112	锥形螺纹车丝机	直径	45mm	—	—	9.24
113	螺栓套丝机	直径 mm	—	—	—	25.00
114	板料校平机	厚度×宽度	16mm×2000mm	—	—	120.60
115	刨边机	加工长度	12000mm	—	—	75.90
116	半自动切割机	厚度	100mm	—	—	98.00
117	自动仿形切割机	厚度	60mm	—	—	59.35
118	管子切断机	管径	150mm	—	—	12.90
119			250mm	—	—	22.50

住房城乡建设部信息公开 浏览专用

续表 C. 0. 1

序号	机械名称	性能规格		能源用量		
				汽油 (kg)	柴油 (kg)	电 (kWh)
120	型钢剪断机	剪断宽度	500mm	—	—	53. 20
121	型钢矫正机	厚度×宽度	60mm×800mm	—	—	64. 20
122	电动弯管机	管径	108mm	—	—	32. 10
123	液压弯管机	管径	60mm	—	—	27. 00
124	空气锤	锤体质量	75kg	—	—	24. 20
125	摩擦压力机	压力	3000kN	—	—	96. 50
126	开式可倾压力机	压力	1250kN	—	—	35. 00
127	钢筋挤压连接机	直径	—	—	—	15. 94
128	电动修钎机	—	—	—	—	100. 80
129	岩石切割机	功率	3kW	—	—	11. 28
130	平面水磨机	功率	3kW	—	—	14. 00
131	喷砂除锈机	能力	$3m^3/min$	—	—	28. 41
132	抛丸除锈机	直径	219mm	—	—	34. 26
133	内燃单级离心清水泵	出口直径	50mm	3. 36	—	—
134	电动多级离心清水泵	出口直径 100mm	扬程 120m 以下	—	—	180. 4
135		出口直径 150mm	扬程 180m 以下	—	—	302. 60
136		出口直径 200mm	扬程 280m 以下	—	—	354. 78
137	泥浆泵	出口直径	50mm	—	—	40. 90
138		出口直径	100mm	—	—	234. 60
139	潜水泵	出口直径	50mm	—	—	20. 00
140			100mm	—	—	25. 00
141	高压油泵	压力	80MPa	—	—	209. 67
142	交流弧焊机	容量	21kV · A	—	—	60. 27
143			32kV · A	—	—	96. 53
144			40kV · A	—	—	132. 23

住房城乡建设部信息公开
浏览专用

续表 C.0.1

序号	机械名称	性能规格		能源用量 汽油(kg)	能源用量 柴油(kg)	能源用量 电(kWh)
145	点焊机	容量	75kV·A	—	—	154.63
146	对焊机	容量	75kV·A	—	—	122.00
147	氩弧焊机	电流	500A	—	—	70.70
148	二氧化碳气体保护焊机	电流	250A	—	—	24.50
149	电渣焊机	电流	1000A	—	—	147.00
150	电焊条烘干箱	容量	45×35×45(cm^3)	—	—	6.70
151	电动空气压缩机	排气量	0.3m^3/min	—	—	16.10
152			0.6m^3/min	—	—	24.20
153			1m^3/min	—	—	40.30
154			3m^3/min	—	—	107.50
155			6m^3/min	—	—	215.00
156			9m^3/min	—	—	350.00
157			10m^3/min	—	—	403.20
158	导杆式液压抓斗成槽机	—	—	—	163.39	—
159	超声波侧壁机	—	—	—	—	36.85
160	泥浆制作循环设备	—	—	—	—	503.90
161	锁扣管顶升机	—	—	—	—	64.00
162	工程地质液压钻机	—	—	—	30.80	—
163	轴流通风机	功率	7.5kW	—	—	40.30
164	吹风机	能力	4m^3/min	—	—	6.98
165	井点降水钻机	—	—	—	—	5.70

住房城乡建设部信息公开
浏览专用

附录 D　建材碳排放因子

D.0.1　建筑材料碳排放因子应按表 D.0.1 选取。

表 D.0.1　建筑材料碳排放因子

建筑材料类别	建筑材料碳排放因子
普通硅酸盐水泥(市场平均)	735 kg CO_2e/t
C30 混凝土	295 kg CO_2e/m^3
C50 混凝土	385 kg CO_2e/ m^3
石灰生产(市场平均)	1190 kg CO_2e/t
消石灰(熟石灰、氢氧化钙)	747 kg CO_2e/t
天然石膏	32.8 kg CO_2e/t
砂(f=1.6～3.0)	2.51 kg CO_2e/t
碎石(d=10mm～30mm)	2.18 kg CO_2e/t
页岩石	5.08 kg CO_2e/t
黏土	2.69 kg CO_2e/t
混凝土砖(240mm×115mm×90mm)	336 kg CO_2e/m^3
蒸压粉煤灰砖(240mm×115mm×53mm)	341 kg CO_2e/m^3
烧结粉煤灰实心砖(240mm×115mm×53mm，掺入量为 50%)	134 kg CO_2e/m^3
页岩实心砖(240mm×115mm×53mm)	292 kg CO_2e/m^3
页岩空心砖(240mm×115mm×53mm)	204 kg CO_2e/m^3
黏土空心砖(240mm×115mm×53mm)	250 kg CO_2e/m^3
煤矸石实心砖(240mm×115mm×53mm，90%掺入量)	22.8 kg CO_2e/m^3
煤矸石空心砖(240mm×115mm×53mm，90%掺入量)	16.0 kg CO_2e/m^3
炼钢生铁	1700 kg CO_2e/t
铸造生铁	2280 kg CO_2e/t

住房城乡建设部信息公开
浏览专用

续表 D.0.1

建筑材料类别	建筑材料碳排放因子
炼钢用铁合金(市场平均)	9530 kg CO_2e/ t
转炉碳钢	1990 kg CO_2e/t
电炉碳钢	3030 kg CO_2e/t
普通碳钢(市场平均)	2050 kg CO_2e/ t
热轧碳钢小型型钢	2310 kg CO_2e/t
热轧碳钢中型型钢	2365 kg CO_2e/t
热轧碳钢大型轨梁(方圆坯、管坯)	2340 kg CO_2e/t
热轧碳钢大型轨梁(重轨、普通型钢)	2380 kg CO_2e/t
热轧碳钢中厚板	2400 kg CO_2e/t
热轧碳钢 H 钢	2350 kg CO_2e/t
热轧碳钢宽带钢	2310 kg CO_2e/t
热轧碳钢钢筋	2340 kg CO_2e/t
热轧碳钢高线材	2375 kg CO_2e/t
热轧碳钢棒材	2340 kg CO_2e/t
螺旋埋弧焊管	2520 kg CO_2e/t
大口径埋弧焊直缝钢管	2430 kg CO_2e/t
焊接直缝钢管	2530 kg CO_2e/t
热轧碳钢无缝钢管	3150 kg CO_2e/t
冷轧冷拔碳钢无缝钢管	3680 kg CO_2e/t
碳钢热镀锌板卷	3110 kg CO_2e/t
碳钢电镀锌板卷	3020 kg CO_2e/t
碳钢电镀锡板卷	2870 kg CO_2e/t
酸洗板卷	1730 kg CO_2e/t
冷轧碳钢板卷	2530 kg CO_2e/t
冷硬碳钢板卷	2410 kg CO_2e/t
平板玻璃	1130 kg CO_2e/t
电解铝(全国平均电网电力)	20300 kg CO_2e/t
铝板带	28500 kg CO_2e/t

住房城乡建设部信息公开 浏览专用

续表 D.0.1

建筑材料类别		建筑材料碳排放因子
断桥铝合金窗	100%原生铝型材	254 kg CO_2e/m^2
	原生铝：再生铝=7：3	194 kg CO_2e/m^2
铝木复合窗	100%原生铝型材	147 kg CO_2e/m^2
	原生铝：再生铝=7：3	122.5 kg CO_2e/m^2
铝塑共挤窗		129.5 kg CO_2e/m^2
塑钢窗		121 kg CO_2e/m^2
无规共聚聚丙烯管		3.72 kg CO_2e/kg
聚乙烯管		3.60 kg CO_2e/kg
硬聚氯乙烯管		7.93 kg CO_2e/kg
聚苯乙烯泡沫板		5020 kg CO_2et
岩棉板		1980 kg CO_2e/t
硬泡聚氨酯板		5220 kg CO_2e/t
铝塑复合板		8.06 kg CO_2e/m^2
铜塑复合板		37.1 kg CO_2e/m^2
铜单板		218 kg CO_2e/m^2
普通聚苯乙烯		4620 kg CO_2e/t
线性低密度聚乙烯		1990 kg CO_2e/t
高密度聚乙烯		2620 kg CO_2e/t
低密度聚乙烯		2810 kg CO_2e/t
聚氯乙烯(市场平均)		7300 kg CO_2e/t
自来水		0.168 kg CO_2e/t

住房城乡建设部信息公开
浏览专用

附录E　建材运输碳排放因子

E.0.1　混凝土的默认运输距离值应为40km，其他建材的默认运输距离值应为500km。各类运输方式的碳排放因子应按表E.0.1选取。

表E.0.1　各类运输方式的碳排放因子［kg CO_2e/（t·km）］

运输方式类别	碳排放因子
轻型汽油货车运输（载重2t）	0.334
中型汽油货车运输（载重8t）	0.115
重型汽油货车运输（载重10t）	0.104
重型汽油货车运输（载重18t）	0.104
轻型柴油货车运输（载重2t）	0.286
中型柴油货车运输（载重8t）	0.179
重型柴油货车运输（载重10t）	0.162
重型柴油货车运输（载重18t）	0.129
重型柴油货车运输（载重30t）	0.078
重型柴油货车运输（载重46t）	0.057
电力机车运输	0.010
内燃机车运输	0.011
铁路运输（中国市场平均）	0.010
液货船运输（载重2000t）	0.019
干散货船运输（载重2500t）	0.015
集装箱船运输（载重200TEU）	0.012

住房城乡建设部信息公开
浏览专用

本标准用词说明

1 为便于在执行本标准条文时区别对待，对要求严格程度不同的用词说明如下：

1）表示很严格，非这样做不可的：

正面词采用“必须”，反面词采用“严禁”；

2）表示严格，在正常情况下均应这样做的：

正面词采用“应”，反面词采用“不应”或“不得”；

3）表示允许稍有选择，在条件许可时首先应这样做的：

正面词采用“宜”，反面词采用“不宜”；

4）表示有选择，在一定条件下可以这样做的，采用“可”。

2 条文中指明应按其他有关标准执行的写法为：“应符合……的规定”或“应按……执行”。

住房城乡建设部信息公开
浏览专用

引用标准名录

1 《民用建筑节水设计标准》GB 50555
2 《环境管理　生命周期评价　原则与框架》GB/T 24040
3 《环境管理　生命周期评价　要求与指南》GB/T 24044
4 《建筑节能气象参数标准》JGJ/T 346

住房城乡建设部信息公开
浏览专用
