# Supplementary material for: Carbon emission quantification analysis of excavation engineering under road transport conditions
Source: PLoS One. 2024 Dec 31;19(12):e0315765. doi: 10.1371/journal.pone.0315765 (PMC11687867; doi:10.1371/journal.pone.0315765)
Supplement: S3 File — (PDF) [file pone.0315765.s003.pdf]

ICS 43.020
CCS R 06

中华人民共和国国家标准

GB/T 4352—XXXX
代替 GB/T 4352—2007

载货汽车运行燃料消耗量

Fuel consumption for trucks in operation

（征求意见稿）

在提交反馈意见时，请将您知道的相关专利连同支持性文件一并附上

XXXX－XX－XX 发布　　XXXX－XX－XX 实施

国家市场监督管理总局
国家标准化管理委员会　发布

目　次

前言 II
1　范围 1
2　规范性引用文件 1
3　术语和定义 1
4　载货汽车运行条件分类及修正系数 2
5　载货汽车运行燃料消耗量计算 3
附录 A（资料性）载货汽车运行燃料消耗量计算推荐值 6
附录 B（资料性）最大总质量超过 3500kg 的载货汽车运行燃料消耗量计算示例 7
附录 C（资料性）最大总质量不超过 3500kg 的载货汽车运行燃料消耗量计算示例 8

前　言

本文件按照GB/T 1.1—2020《标准化工作导则 第1部分：标准化文件的结构和起草规则》的规定起草。

本文件代替GB/T 4352—2007《载货汽车运行燃料消耗量》。与GB/T 4352—2007相比，除结构调整和编辑性改动外，主要技术变化如下：

——更改了术语和定义中的“载货汽车基本燃料消耗量”（见3.2，2007年版3.2）；

——更改了术语和定义中的“载货汽车满载燃料消耗量”（见3.3，2007年版3.3）；

——更改了术语和定义中的“载货汽车质量变化附加燃料消耗量”，并将条款名称更改为“载货汽车单位载质量变化燃料消耗量”（见3.4，2007年版3.4）；

——增加了术语和定义中的“载货汽车附加燃料消耗量”（见3.5）；

——更改了术语和定义中的“道路修正系数”，条款名称更改为“燃料消耗量道路修正系数”（见3.6，2007年版3.5）；

——更改了术语和定义中的“气温修正系数”，条款名称更改为“燃料消耗量气温修正系数”（见3.7，2007年版3.6）；

——删除了术语和定义中的“海拔高度修正系数”（见2007年版3.7）；

——增加了术语和定义中的“燃料消耗量交通拥堵修正系数”（见3.8）；

——更改了“载货汽车运行条件分类”，条款名称更改为“载货汽车运行条件分类及修正系数”（见第4章，2007年版第4章）；

——删除了“基本运行条件”（见2007年版4.1）；

——更改了“道路类别”，将原标准中的道路修正系数移入本条款，相应更改名称及内容（见4.1，2007年版4.2.1、5.4.1）；

——更改了“气温区间”，将原标准中的气温修正系数移入本条款，并对系数进行了调整，相应更改名称及内容（见4.2，2007年版4.2.2、5.4.2）；

——删除了“海拔高度区间”（见2007年版4.2.3）；

——增加了“交通拥堵及燃料消耗量交通拥堵修正系数K_v”（见4.3）；

——删除了“载货汽车运行模式”（见2007年版4.3）；

——更改了“其他影响因素修正系数”的位置并对名称进行了调整（见4.4，2007年版5.4.4）；

——更改了“载货汽车运行燃料消耗量”的条款名称（见第5章，2007年版第5章）；

——删除了“载货汽车整备质量（空载）及总质量（满载）下的等速燃料消耗量”（见2007年版5.1）；

——删除了“载货汽车基本燃料消耗量及满载燃料消耗量”（见2007年版5.2）；

——更改了“载货汽车质量变化附加燃料消耗量”相关内容，并将条款名称更改为“载货汽车单位载质量变化燃料消耗量计算”（见5.1，2007年版5.3）；

——删除了“载货汽车运行燃料消耗量修正系数”（见2007年版5.4）；

——更改了“载货汽车运行燃料消耗量计算”，对计算公式进行了调整（见5.2，2007年版5.5）；

——增加了“附录A 载货汽车运行燃料消耗量计算推荐值”（见附录A）；

——更改了“附录A 载货汽车运行燃料消耗量计算示例”，调整为附录B，并将附录名称更改为“最大总质量超过3500kg的载货汽车运行燃料消耗量计算示例”（见附录B，2007年版附录

A）；

——增加了“附录 C 最大总质量不超过 3500kg 的载货汽车运行燃料消耗量计算示例”（见附录 C）；

本文件由全国道路运输标准化技术委员会（SAC/ TC 521）提出并归口。

本文件起草单位：交通运输部公路科学研究院、中公高远（北京）汽车检测技术有限公司。

本文件主要起草人：李臬、蔡凤田、余海涛、刘莉、石则强、高润泽、庞知非、赵洪雪。

本文件及其所代替文件的历次版本发布情况为：

——1984 年首次发布为 GB/T 4352—1984；

——2007 年修订为 GB/T 4352—2007；

——本次为第二次修订。

载货汽车运行燃料消耗量

1 范围

本文件规定了载货汽车运行条件分类和修正系数以及运行燃料消耗量的计算方法。

本文件适用于行驶在公路和城市道路上使用汽油或柴油作为燃料的载货汽车运行燃料消耗量的计算。

2 规范性引用文件

下列文件中的内容通过文中的规范性引用而构成本文件必不可少的条款。其中，注日期的引用文件，仅该日期对应的版本适用于本文件；不注日期的引用文件，其最新版本（包括所有的修改的）适用于本文件。

GB/T 19233 轻型汽车燃料消耗量试验方法

JT/T 719 营运货车燃料消耗量限值及测量方法

JTG B01 公路工程技术标准

3 术语和定义

JT/T 719 确立的以及下列术语和定义适用于本文件。

3.1

载货汽车运行燃料消耗量 fuel consumption for truck in operation

载货汽车在运行过程中消耗的燃料数量，单位为升（L）。

3.2

载货汽车基本燃料消耗量 basic fuel consumption of truck

载货汽车按照规定工况，以整备质量（空载）行驶时每百公里消耗的燃料数量，单位为升每百公里（L/100km）。

3.3

载货汽车满载燃料消耗量 fuel consumption of fully loaded truck

载货汽车按照规定工况，以最大总质量（满载）行驶时每百公里消耗的燃料数量，单位为升每百公里（L/100km）。

3.4

载货汽车单位载质量变化燃料消耗量 changes in fuel consumption per unit load of truck

载货汽车载质量每增加1t，行驶100km所增加的燃料消耗数量，单位为升每吨百公里（L/100km·t）。

3.5

载货汽车附加燃料消耗量 additional fuel consumption of truck

载货汽车不用于驱动车辆行驶的主要用能设备工作时消耗的燃料数量，如空调、冷藏车制冷机组、随车装卸机械等。

3.6

燃料消耗量道路修正系数 fuel consumption correction coefficient of road

汽车运行在某类道路上的燃料消耗量与在1类道路上（其他运行条件相同）的燃料消耗量的比值。

3.7

燃料消耗量气温修正系数 fuel consumption correction coefficient of temperature

汽车运行在某月平均气温区间时的燃料消耗量与月平均气温区间为5℃至28℃时（其他运行条件相同）的燃料消耗量的比值。

3.8

燃料消耗量交通拥堵修正系数 fuel consumption correction coefficient of traffic congestion

汽车运行在某平均速度区间时的燃料消耗量与平均时速为30km/h至40km/h时（其他运行条件相同）的燃料消耗量的比值。

4 载货汽车运行条件分类及修正系数

4.1 道路类别及燃料消耗量道路修正系数 K_r

道路类别及燃料消耗量道路修正系数 K_r 见表1。

表 1 道路类别及燃料消耗量道路修正系数 K_r 表

道路类别	公　路	城市道路	K_r
1类道路	平原、微丘地形的高速、一、二级公路	/	1.00
2类道路	平原、微丘地形的三、四级公路，山岭、重丘地形的高速公路	平原、微丘地形的一、二、三、四级道路	1.10
3类道路	山岭、重丘地形的一、二、三级公路	重丘地形的一、二、三、四级道路	1.25
4类道路	平原、微丘地形的级外公路	级外道路	1.35
5类道路	山岭、重丘地形的四级公路	/	1.45
6类道路	山岭、重丘地形的级外公路	/	1.70
注：公路等级按 JTG B01 规定划分			

4.2 气温区间及燃料消耗量气温修正系数 K_t

气温值以气象台（站）公布的当地月平均气温，分为低于-25℃（含）、-25℃～-15℃（含）、-15℃～

-5℃（含）、-5℃～5℃（含）、5℃～28℃（含）、高于28℃六个区间。

燃料消耗量气温修正系数K_t见表2。

表 2 燃料消耗量气温修正系数K_t表

月平均气温（*T*）℃	*T*≤ -25	-25＜*T*≤ -15	-15＜*T*≤ -5	-5＜*T*≤ 5	5＜*T*≤28	*T*＞28
K_t	1.13	1.09	1.06	1.03	1.00	0.98

4.3 拥堵及燃料消耗量交通拥堵修正系数K_v

交通拥堵程度以车辆平均运行速度划分，分别为低于20km/h（含）、20km/h～30km/h（含）、30km/h～40km/h（含）、40km/h～50km/h（含）、高于50km/h五个区间。

燃料消耗量交通拥堵修正系数K_v见表3。

表 3 燃料消耗量拥堵修正系数K_v表

平均行驶速度（*V*）km/h	*V*≤ 20	20＜*V*≤ 30	30＜*V*≤ 40	40＜*V*≤50	*V*＞50
K_v	1.30	1.15	1.00	0.90	0.80

4.4 燃料消耗量其他影响因素修正系数K_x

其他影响载货汽车运行燃料消耗量的因素（如载货汽车走合期，驾驶实习期，地方性雨季期，装载危险品，翻浆、冰雪道路等）的附加或修正系数K_x，由用车单位自行规定。

5 载货汽车运行燃料消耗量计算

5.1 载货汽车单位载质量变化燃料消耗量计算

载货汽车单位载质量变化燃料消耗量按式（1）计算

$$Q_b = \frac{Q_m - Q_k}{M_m - M_k} \quad \cdots\cdots\cdots\cdots(1)$$

式中：

Q_b—载货汽车单位载质量变化燃料消耗量，单位为升每吨百公里（L/100 km•t）；

Q_m—载货汽车满载燃料消耗量，车辆满载，对于最大总质量不超过 3500kg 的载货汽车，取值按照 GB/T 19233 标准规定的试验循环工况得出的综合燃料消耗量;对于最大总质量超过 3500kg 的载货汽车，取值按照 JT/T 719 标准规定的试验循环工况得出的综合燃料消耗量（或经交通运输部道路运输车辆达标车型表查询），单位为升每百公里（L/100km）。

Q_k—载货汽车基本燃料消耗量，车辆空载，对于最大总质量不超过 3500kg 的载货汽车，取值按照 GB/T 19233 标准规定的试验循环工况得出的综合燃料消耗量(或经轻型汽车燃料消耗量标识查询）；对于最大总质量超过 3500kg 的载货汽车，取值按照 JT/T 719 标准规定的试验循环工况得出的综合燃料消耗量，单位为升每百公里（L/100km）。

M_m—载货汽车最大总质量，单位为吨（t）；

M_k—载货汽车整备质量，单位为吨（t）。

对于最大总质量超过 3500kg 的载货汽车，当生产企业未给出载货汽车基本燃料消耗量数值时，宜参考附录 A 选取 Q_k 及 Q_b 值。

5.2 载货汽车运行燃料消耗量计算

某种运行模式的载货汽车运行燃料消耗量按式（2）计算：

$$Q_i = (Q_k \bullet \frac{S_i}{100} + Q_b \bullet \frac{\Delta G_i \bullet S_i}{100}) \bullet K_{ri} \bullet K_{ti} \bullet K_{vi} \bullet K_{xi} + Q_{ai} \qquad \text{………………（2）}$$

式中：

Q_i—第i种运行模式下的载货汽车运行燃料消耗量，单位为升（L）；

S_i—第i种运行模式下的载货汽车行驶里程，单位为公里（km）；

ΔG_i—第i种运行模式下的载货汽车载质量，单位为吨（t）；

K_{ri}—第i种运行模式下的载货汽车燃料消耗量道路修正系数，见表1；

K_{ti}—第i种运行模式下的载货汽车燃料消耗量气温修正系数，见表2；

K_{vi}—第i种运行模式下的载货汽车燃料消耗量交通拥堵修正系数，见表3；

K_{xi}—第i种运行模式下的载货汽车燃料消耗量其他影响因素修正系数，由用车单位自行规定；

Q_{ai}—第i种运行模式下的载货汽车附加燃料消耗量，具体数值由用车单位自行规定，单位为升（L）。

不同运行模式的载货汽车运行燃料消耗总量按式（3）计算：

$$Q=\sum_{i=1}^{n}Q_i \qquad \text{………………（3）}$$

式中：

Q—不同运行模式的载货汽车运行燃料消耗总量，单位为升（L）。

5.3 载货汽车运行燃料消耗量计算示例

最大总质量超过3500kg的载货汽车运行燃料消耗量计算示例见附录B。
最大总质量不超过3500kg的载货汽车运行燃料消耗量计算示例见附录C。

附 录 A
（资料性）
载货汽车运行燃料消耗量计算推荐值

载货汽车运行燃料消耗量计算推荐值适用于最大总质量超过3500kg的载货汽车，包括货车单车及半挂牵引车（列车）和牵引货车（列车）两类车型，见表A.1。

表A.1 最大总质量超过 3500kg的载货汽车运行燃料消耗量计算推荐值表

货车单车		
载质量（m_1）<br>t	基本燃料消耗量(Q_k)<br>L/100km	单位载质量变化燃料消耗量（Q_b）<br>L/100km•t
m≤2	8.7	1.17
2<m≤4	11.2	0.97
4<m≤5	13.6	0.87
5<m≤8	14.5	0.81
8<m≤10	18.3	0.60
10<m≤17	22.9	0.46
17<m≤21	24.6	0.42
半挂牵引车（列车）和牵引货车（列车）		
准拖挂车总质量（m_2）<br>t	基本燃料消耗量（Q_k）<br>L/100km	单位载质量变化燃料消耗量（Q_b）<br>L/100km•t
m≤19	14.7	1.06
19<m≤28	17.1	0.67
28<m≤35	18.3	0.53
35<m≤41	18.7	0.48
注 1：载质量 m_l=$M_m - M_k$<br>注 2：准拖挂车总质量 m_2=挂车整备质量+载质量		

附 录 B
（资料性）
最大总质量超过 3500kg 的载货汽车运行燃料消耗量计算示例

某载货汽车最大总质量为9.9t，整备质量4.4t，额定载质量5.5t，已行驶里程30000km，在月平均气温-3℃的城市之间平原地形的三级路上，载货2.75t行驶30km，卸货后空驶返回原地，运行时段为城市晚高峰时段，存在拥堵，平均行驶速度为22km/h，行驶过程中未开启空调等用能设备，求载货汽车运行燃料消耗总量。

a）由汽车生产企业提供的载货汽车基本燃料消耗量 Q_k 为16.1（L/100km），经查交通运输部道路运输车辆达标车型表得到载货汽车满载燃料消耗量 Q_m 为20.8（L/100km）。

b）按照公式（1）计算载货汽车质量变化附加燃料消耗量 Q_b：

$$Q_b=\frac{Q_m-Q_k}{M_m-M_k}=\frac{20.8-16.1}{9.9-4.4}=0.85\text{（L/100km·t）}$$

c）按照公式（2）计算载货汽车运行燃料消耗量

根据已知条件确定，道路类别2类的修正系数为1.10，月平均气温-3℃的修正系数为1.03，平均行驶速度为22km/h的交通拥堵修正系数为1.15，无其他影响因素的修正系数为1.0，行驶过程中没有使用空调等用能设备，因此载货行程的运行燃料消耗量为：

$$Q_1=(Q_k\bullet\frac{S_1}{100}+Q_b\bullet\frac{\Delta G_1\bullet S_1}{100})\bullet K_{r1}\bullet K_{t1}\bullet K_{v1}\bullet K_{x1}+Q_{a1}$$

$$=(16.1\times\frac{30}{100}+0.85\times\frac{2.75\times 30}{100})\times 1.10\times 1.03\times 1.15\times 1.00+0=7.21\text{（L）}$$

根据已确定的各项修正系数，空载行程运行燃料消耗量为：

$$Q_2=(Q_k\bullet\frac{S_2}{100}+Q_b\bullet\frac{\Delta G_2\bullet S_2}{100})\bullet K_{r2}\bullet K_{t2}\bullet K_{v2}\bullet K_{x2}+Q_{a2}$$

$$=(16.1\times\frac{30}{100}+0.85\times\frac{0\times 30}{100})\times 1.10\times 1.03\times 1.15\times 1.00+0=6.29\text{（L）}$$

d）按照公式（3）计算不同运行模式下的载货汽车运行燃料消耗总量：

$$Q=Q_1+Q_2=7.21+6.29=13.5\text{（L）}$$

附　录　C
（资料性）
最大总质量不超过 3500kg 的载货汽车运行燃料消耗量计算示例

某载货汽车最大总质量为3.4t，整备质量1.6t，额定载质量1.8t，已行驶里程30000km，在月平均气温-3℃的城市之间平原地形的三级路上，满载行驶30km，卸货后空驶返回原地，运行时段为城市晚高峰时段，存在拥堵，平均行驶速度为22km/h，行驶过程中未开启空调等用能设备，求载货汽车运行燃料消耗总量。

a）经轻型汽车燃料消耗量标识查询得到载货汽车基本燃料消耗量 Q_k 为7.8（L/100km），由汽车生产企业提供的载货汽车满载燃料消耗量 Q_m 为10.2（L/100km）。

b）按照公式（1）计算载货汽车质量变化附加燃料消耗量 Q_b：

$$Q_b=\frac{Q_m-Q_k}{M_m-M_k}=\frac{10.2-7.8}{3.4-1.6}=1.33\text{（L/100km•t）}$$

c）按照公式（2）计算载货汽车运行燃料消耗量

根据已知条件确定，道路类别2类的修正系数为1.10，月平均气温-3℃的修正系数为1.03，平均行驶速度为22km/h的交通拥堵修正系数为1.15，无其他影响因素的修正系数为1.0，行驶过程中没有使用空调等用能设备，因此载货行程的运行燃料消耗量为：

$$Q_1=(Q_k\bullet\frac{S_1}{100}+Q_b\bullet\frac{\Delta G_1\bullet S_1}{100})\bullet K_{r1}\bullet K_{t1}\bullet K_{v1}\bullet K_{x1}+Q_{a1}$$

$$=(7.8\times\frac{30}{100}+1.33\times\frac{1.8\times 30}{100})\times 1.10\times 1.03\times 1.15\times 1.00+0=3.98\text{（L）}$$

根据已确定的各项修正系数，空载行程运行燃料消耗量为：

$$Q_2=(Q_k\bullet\frac{S_2}{100}+Q_b\bullet\frac{\Delta G_2\bullet S_2}{100})\bullet K_{r2}\bullet K_{t2}\bullet K_{v2}\bullet K_{x2}+Q_{a2}$$

$$=(7.8\times\frac{30}{100}+1.33\times\frac{0\times 30}{100})\times 1.10\times 1.03\times 1.15\times 1.00+0=3.05\text{（L）}$$

d）按照公式（3）计算不同运行模式下的载货汽车运行燃料消耗总量：

$$Q=Q_1+Q_2=3.98+3.05=7.03\text{（L）}$$
