# Supplementary material for: Carbon emission quantification analysis of excavation engineering under road transport conditions
Source: PLoS One. 2024 Dec 31;19(12):e0315765. doi: 10.1371/journal.pone.0315765 (PMC11687867; doi:10.1371/journal.pone.0315765)
Supplement: S4 File — (PDF) [file pone.0315765.s004.pdf]

2018年全国统一施工机械台班费用定额

一、土石方及筑路机械

编号	机械名称		机型	规格型号		折旧年限	台班基价	费用组成													
								折旧费	大修理费	经常修理费	安拆费及场外运输费	燃料动力费	其中						人工费	其中	养路费及车船使用税
								定额					汽油	柴油	煤	电	水	木柴		人工	
													9.90	8.60	600.00	1.000	2.00	2.00		53.00	
						定额	元	元	元	元	元	元	kg	kg	t	kw·h	m3	kg	元	工日	元
1-1	松土机		大	松土深度（m）	0.5	13	954.40	189.58	42.86	122.14		467.32		54.34					132.50	2.50	
1-2	松土机		大	松土深度（m）	1	13	987.14	193.41	50.37	143.54		467.32		54.34					132.50	2.50	
1-3	除荆机		大	清除宽度（m）	4	13	953.74	182.36	44.22	127.34		467.32		54.34					132.50	2.50	
1-4	除根机		大	清除宽度（m）	1.5	13	944.63	177.07	43.23	124.51		467.32		54.34					132.50	2.50	
1-5	履带式推土机		中	功率（Kw）	50	11	502.91	45.56	17.61	45.80		261.44		30.40					132.50	2.50	
1-6	履带式推土机		中	功率（Kw）	55	11	552.32	46.59	19.44	50.55		303.24		35.26					132.50	2.50	
1-7	履带式推土机		中	功率（Kw）	60	11	611.30	48.54	21.57	56.09		352.60		41.00					132.50	2.50	
1-8	履带式推土机		大	功率（Kw）	75	11	867.29	139.90	36.27	94.31		464.31		53.99					132.50	2.50	
1-9	履带式推土机		大	功率（Kw）	90	11	973.69	179.98	42.70	111.02		507.49		59.01					132.50	2.50	
1-10	履带式推土机		大	功率（Kw）	105	11	1001.58	199.70	44.73	116.30		508.35		59.11					132.50	2.50	
1-11	履带式推土机		大	功率（Kw）	135	11	1329.64	353.72	78.53	204.17		560.72		65.20					132.50	2.50	
1-12	履带式推土机		大	功率（Kw）	165	11	1553.60	421.56	107.35	279.10		613.09		71.29					132.50	2.50	
1-13	履带式推土机		特	功率（Kw）	240	11	2019.71	606.73	178.21	358.20		744.07		86.52					132.50	2.50	
1-14	履带式推土机		特	功率（Kw）	313	11	2266.22	708.49	228.50	422.73		774.00		90.00					132.50	2.50	
1-15	湿地推土机		大	功率（Kw）	105	11	931.54	211.82	44.94	110.56		431.72		50.20					132.50	2.50	
1-16	湿地推土机		大	功率（Kw）	135	11	1290.87	356.84	78.72	193.65		529.16		61.53					132.50	2.50	
1-17	湿地推土机		大	功率（Kw）	165	11	1510.98	422.95	107.52	264.50		583.51		67.85					132.50	2.50	
1-18	轮胎式推土机		大	功率（Kw）	150	11	1524.74	294.19	52.33	183.14		862.58		100.30					132.50	2.50	
1-19	自行式铲运机	单引擎	中	斗容量（m3）	3	12	800.15	189.50	48.56	130.14		299.45		34.82					132.50	2.50	
1-20	自行式铲运机	单引擎	大	斗容量（m3）	4	12	900.55	281.11	50.95	136.54		299.45		34.82					132.50	2.50	
1-21	自行式铲运机	单引擎	大	斗容量（m3）	6	12	1010.11	299.63	53.84	144.28		379.86		44.17					132.50	2.50	
1-22	自行式铲运机	单引擎	大	斗容量（m3）	8	12	1144.08	324.04	58.46	156.68		472.40		54.93					132.50	2.50	
1-23	自行式铲运机	单引擎	大	斗容量（m3）	10	12	1255.76	332.46	62.22	166.74		561.84		65.33					132.50	2.50	
1-24	自行式铲运机	单引擎	大	斗容量（m3）	12	12	1455.24	438.51	77.84	208.60		597.79		69.51					132.50	2.50	
1-25	自行式铲运机	双引擎	大	斗容量（m3）	12	12	2373.35	531.93	166.65	446.63		1095.64		127.40					132.50	2.50	
1-26	自行式铲运机	双引擎	大	斗容量（m3）	23	12	3683.71	1474.59	239.42	641.63		1195.57		139.02					132.50	2.50	
1-27	拖式铲运机		中	斗容量（m3）	3	3	593.87	37.90	28.39	93.39		301.69		35.08					132.50	2.50	
1-28	拖式铲运机		大	斗容量（m3）	8	8	1011.08	130.46	56.03	184.35		507.74		59.04					132.50	2.50	
1-29	拖式铲运机		大	斗容量（m3）	10	10	1252.11	151.50	112.32	262.82		592.97		68.95					132.50	2.50	
1-30	拖式铲运机		大	斗容量（m3）	12	12	1431.19	178.85	142.58	333.64		643.62		74.84					132.50	2.50	
1-31	平地机		大	功率（Kw）	75	11	613.41	155.75	25.57	88.20		211.39		24.58					132.50	2.50	
1-32	平地机		大	功率（Kw）	90	11	741.09	170.98	29.85	102.98		304.78		35.44					132.50	2.50	
1-33	平地机		大	功率（Kw）	120	11	1026.87	256.12	37.19	128.32		472.74		54.97					132.50	2.50	
1-34	平地机		大	功率（Kw）	135	11	1194.94	287.27	57.02	196.73		521.42		60.63					132.50	2.50	
1-35	平地机		大	功率（Kw）	150	11	1373.10	322.57	74.90	258.42		584.71		67.99					132.50	2.50	
1-36	平地机		大	功率（Kw）	180	11	1666.01	380.72	101.10	348.81		702.88		81.73					132.50	2.50	
1-37	平地机		大	功率（Kw）	220	11	2103.33	503.93	141.06	486.65		839.19		97.58					132.50	2.50	
1-38	轮胎式装载机		中	斗容量（m3）	1	12	714.85	91.60	22.70	80.82		453.48		52.73					66.25	1.25	
1-39	轮胎式装载机		大	斗容量（m3）	1.5	12	792.86	107.69	24.93	88.74		505.25		58.75					66.25	1.25	
1-40	轮胎式装载机		大	斗容量（m3）	2	12	917.01	151.44	30.36	108.07		560.89		65.22					66.25	1.25	
1-41	轮胎式装载机		大	斗容量（m3）	2.5	12	1043.72	176.69	33.95	120.88		645.95		75.11					66.25	1.25	
1-42	轮胎式装载机		大	斗容量（m3）	3	12	1222.49	193.51	39.23	139.67		717.58		83.44					132.50	2.50	
1-43	轮胎式装载机		大	斗容量（m3）	4.5	12	1408.11	249.60	50.46	179.62		795.93		92.55					132.50	2.50	
1-44	履带式拖拉机		中	功率（Kw）	55	14	576.77	39.19	16.20	43.42		345.46		40.17					132.50	2.50	
1-45	履带式拖拉机		中	功率（Kw）	60	14	629.37	45.85	19.97	53.51		377.54		43.90					132.50	2.50	
1-46	履带式拖拉机		大	功率（Kw）	75	11	829.87	107.29	33.36	89.40		467.32		54.34					132.50	2.50	
1-47	履带式拖拉机		大	功率（Kw）	90	11	966.49	180.67	39.63	106.20		507.49		59.01					132.50	2.50	
1-48	履带式拖拉机		大	功率（Kw）	105	11	1102.16	199.98	41.36	110.84		617.48		71.80					132.50	2.50	
1-49	履带式拖拉机		大	功率（Kw）	120	11	1265.01	239.16	55.71	149.30		688.34		80.04					132.50	2.50	
1-50	履带式拖拉机		大	功率（Kw）	135	11	1418.56	262.70	72.67	194.75		755.94		87.90					132.50	2.50	
1-51	轮胎式拖拉机		中	功率（Kw）	21	7	270.32	20.38	10.67	22.52		150.50		17.50					66.25	1.25	
1-52	轮胎式拖拉机		中	功率（Kw）	41	7	468.83	36.21	23.23	49.02		294.12		34.20					66.25	1.25	
1-53	手扶式拖拉机		小	功率（Kw）	9	7	161.93	6.49	3.82	8.06		77.31		8.99					66.25	1.25	
1-54	履带式单斗挖掘机	液压	大	斗容量（m3）	0.6	12	750.31	172.96	47.90	107.30		289.65		33.68					132.50	2.50	
1-55	履带式单斗挖掘机	液压	大	斗容量（m3）	0.8	12	992.79	254.00	56.05	118.26		431.98		50.23					132.50	2.50	
1-56	履带式单斗挖掘机	液压	大	斗容量（m3）	1	12	1143.72	281.21	60.52	127.69		541.80		63.00					132.50	2.50	
1-57	履带式单斗挖掘机	液压	大	斗容量（m3）	1.25	12	1347.32	309.64	74.70	157.62		672.86		78.24					132.50	2.50	
1-58	履带式单斗挖掘机	液压	大	斗容量（m3）	1.6	12	1633.35	357.41	87.35	184.31		871.78		101.37					132.50	2.50	
1-59	履带式单斗挖掘机	液压	大	斗容量（m3）	1.8	12	1829.15	373.44	101.16	213.44		1008.61		117.28					132.50	2.50	
1-60	履带式单斗挖掘机	液压	大	斗容量（m3）	2	12	1632.14	386.74	122.00	257.41		733.49		85.29					132.50	2.50	
1-61	履带式单斗挖掘机	机械	大	斗容量（m3）	1	12	1004.78	287.26	43.22	120.14		421.66		49.03					132.50	2.50	
1-62	履带式单斗挖掘机	机械	大	斗容量（m3）	1.5	12	1389.77	308.43	85.61	238.01		625.22		72.70					132.50	2.50	
1-63	轮胎式挖掘装载机		中	斗容量（m3）	0.2	12	469.54	70.39	19.92	53.00		259.98		30.23					66.25	1.25	
1-64	轮胎式挖掘装载机		大	斗容量（m3）	0.4	12	526.44	81.61	32.40	86.20		259.98		30.23					66.25	1.25	
1-65	轮胎式挖掘装载机		大	斗容量（m3）	0.6	12	549.37	94.79	35.07	93.28		259.98		30.23					66.25	1.25	
1-66	拖式羊角碾	单筒	小	不加载重量（t）	3	13	15.45	7.02	1.23	7.20	4.93										
1-67	拖式羊角碾	双筒	小	不加载重量（t）	6	13	26.36	12.45	2.03	11.88	9.81										
1-68	光轮压路机（内燃）		大	不加载重量（t）	6	11	290.45	65.76	12.71	40.81		104.92		12.20					66.25	1.25	
1-69	光轮压路机（内燃）		大	不加载重量（t）	8	11	373.98	72.68	15.41	49.45		170.19		19.79					66.25	1.25	
1-70	光轮压路机（内燃）		大	不加载重量（t）	12	11	508.18	87.77	18.57	59.62		275.97		32.09					66.25	1.25	
1-71	光轮压路机（内燃）		大	不加载重量（t）	15	11	611.47	93.80	19.49	62.56		369.37		42.95					66.25	1.25	
1-72	光轮压路机（内燃）		大	不加载重量（t）	18	11	1019.50	104.52	20.95	67.25		694.28		80.73					132.50	2.50	
1-73	振动压路机		大	重量（t）	8	11	607.77	122.69	35.52	109.40		273.91		31.85					66.25	1.25	
1-74	振动压路机		大	重量（t）	12	11	881.72	145.92	39.74	122.41		507.40		59.00					66.25	1.25	
1-75	振动压路机		大	重量（t）	15	11	1140.56	159.71	42.26	130.16		742.18		86.30					66.25	1.25	
1-76	手扶振动压实机		小	重量（t）	1	8	161.59	23.45	4.69	18.09	1.99	49.11		5.71					66.25	1.25	
1-77	轮胎压路机		大	不加载重量（t）	9	11	494.64	60.57	22.01	87.81		258.00		30.00					66.25	1.25	
1-78	夯实机	电动	小	夯击能力（kg·m）	20~62	6	28.40	5.45	1.13	5.22	2.49	16.60				16.60					
1-79	夯实机	内燃	小	夯足直径（mm）	φ265	6	34.50	4.98	2.18	10.14	2.49	17.20		2.00							
1-80	装岩机	风力	小	斗容量（m3）	0.12	12	175.32	24.36	6.03	12.43	10.90								132.50	2.50	
1-81	装岩机	电力	中	斗容量（m3）	0.2	12	247.08	29.71	8.36	14.21	10.90	62.30				62.30			132.50	2.50	
1-82	装岩机	电力	大	斗容量（m3）	0.6	12	365.11	45.40	14.40	24.48	10.90	148.33				148.33			132.50	2.50	
1-83	风动凿岩机		小	气腿式		7	13.26	3.77	1.18	8.31	1.99										
1-84	风动凿岩机		小	手持式		7	10.09	2.77	0.91	6.41	1.99										
1-85	凿岩钻车		中	轮胎式		12	279.85	96.17	17.83	33.35	10.90								132.50	2.50	
1-86	凿岩钻车		中	履带式		12	362.56	152.99	28.33	48.74	10.90								132.50	2.50	
1-87	汽车式沥青喷洒机		大	箱容量（L）	4000	11	654.89	159.21	44.70	75.55		309.18	31.23						66.25	1.25	
1-88	汽车式沥青喷洒机		大	箱容量（L）	7500	11	883.00	290.73	67.57	114.19		344.26		40.03					66.25	1.25	
1-89	沥青混凝土摊铺机		大	载重量（t）	4	12	661.43	113.10	35.91	70.74		309.18	31.23						132.50	2.50	
1-90	沥青混凝土摊铺机		大	载重量（t）	6	12	689.63	172.30	41.40	81.56		261.87		30.45					132.50	2.50	
1-91	沥青混凝土摊铺机		大	载重量（t）	8	12	977.74	237.69	66.34	130.70		344.26		40.03					198.75	3.75	
1-92	沥青混凝土摊铺机		大	载重量（t）	12	12	1165.69	251.83	66.93	131.84		516.34		60.04					198.75	3.75	
1-93	沥青混凝土摊铺机（带自动找平）		大	载重量（t）	8	12	1613.19	744.00	88.54	108.90		473.00		55.00					198.75	3.75	
1-94	沥青混凝土摊铺机（带自动找平）		大	载重量（t）	12	12	2647.04	1247.65	156.61	192.63		851.40		99.00					198.75	3.75	
1-95	内燃凿岩机		小	YN30A		4	265.30	5.67	2.08	14.97	1.49	110.08		12.80					132.50	2.50	

1-96	强夯机械	大	夯击能力（tm）	120	8	1050.91	455.47	55.44	125.85		281.65		32.75					132.50	2.50	
1-97	强夯机械	大	夯击能力（tm）	200	8	1724.03	972.46	76.86	174.47		367.74		42.76					132.50	2.50	
1-98	强夯机械	大	夯击能力（tm）	300	8	2006.94	1039.65	109.93	249.54		475.32		55.27					132.50	2.50	
1-99	灰土拌合机	大	功率（Kw）	90	11	803.89	13.81	42.40	107.69		507.49		59.01					132.50	2.50	
1-100	灰土拌合机	大	功率（Kw）	105	11	960.21	161.98	44.46	112.92		508.35		59.11					132.50	2.50	
1-101	灰土拌合机	大	功率（Kw）	135	11	1277.86	331.92	78.80	200.15		534.49		62.15					132.50	2.50	
1-102	颚式破碎机	小	进料口（mm）	250×400	9	192.43	27.25	2.59	35.14		61.20				61.20			66.25	1.25	
1-103	颚式破碎机	小	进料口（mm）	250×500	9	225.29	38.93	3.31	44.80		72.00				72.00			66.25	1.25	
1-104	颚式破碎机	中	进料口（mm）	400×600	9	300.28	66.18	4.11	55.74		108.00				108.00			66.25	1.25	
1-105	颚式破碎机	中	进料口（mm）	500×750	9	514.56	154.25	6.60	89.46		198.00				198.00			66.25	1.25	
1-106	颚式破碎机	中	进料口（mm）	600×900	9	701.70	231.62	7.96	107.87		288.00				288.00			66.25	1.25	
1-107	颚式破碎机（机动）	小	进料口（mm）	250×440	9	375.51	59.36	5.47	74.15		170.28		19.80					66.25	1.25	
二、打桩机械																				
2-1	履带式柴油打桩机	大	锤重（t）	2.5	12	1433.02	436.12	95.60	387.22		381.58		44.37					132.50	2.50	
2-2	履带式柴油打桩机	大	锤重（t）	3.5	12	2178.27	764.04	166.06	703.39		412.28		47.94					132.50	2.50	
2-3	履带式柴油打桩机	大	锤重（t）	5	12	3457.72	1664.95	285.06	911.41		463.80		53.93					132.50	2.50	
2-4	履带式柴油打桩机	特	锤重（t）	7	12	4175.49	1863.33	400.22	1285.80		493.64		57.40					132.50	2.50	
2-5	履带式柴油打桩机	特	锤重（t）	8	12	4465.22	1928.39	440.47	1455.26		508.60		59.14					132.50	2.50	
2-6	轨道式柴油打桩机	中	锤重（t）	0.6	12	483.22	60.66	47.42	128.84		113.80		9.00		36.40			132.50	2.50	
2-7	轨道式柴油打桩机	大	锤重（t）	1.2	12	821.70	171.34	56.15	145.43		316.28		28.80		68.60			132.50	2.50	
2-8	轨道式柴油打桩机	大	锤重（t）	1.8	12	991.59	206.35	62.33	205.17		385.24		33.40		98.00			132.50	2.50	
2-9	轨道式柴油打桩机	大	锤重（t）	2.5	12	1459.08	384.36	69.40	350.92		521.90		46.50		122.00			132.50	2.50	
2-10	轨道式柴油打桩机	大	锤重（t）	3.5	12	2082.84	533.62	119.49	636.89		660.34		56.90		171.00			132.50	2.50	
2-11	轨道式柴油打桩机	大	锤重（t）	4	12	2282.38	578.53	133.95	713.36		724.04		61.70		193.42			132.50	2.50	
2-12	导杆式柴油打桩机	中	锤重（t）	1.5	12	543.30	89.58	13.86	72.08		235.28		22.30		43.50			132.50	2.50	
2-13	震动打拔桩机	大	激震力（t）	30	9	954.07	461.48	12.16	66.74		281.19		17.43		131.29			132.50	2.50	
2-14	震动打拔桩机	大	激震力（t）	40	9	1231.86	586.24	17.18	94.30		401.64		24.90		187.50			132.50	2.50	
2-15	震动打拔桩机	大	激震力（t）	50	9	1514.05	741.94	21.19	116.32		502.10		31.13		234.38			132.50	2.50	
2-16	震动打拔桩机	大	激震力（t）	60	9	1779.28	880.76	25.20	138.36		602.46		37.35		281.25			132.50	2.50	
2-17	静力压桩机（液压）	大	压力（t）	90	11	1473.68	583.00	142.69	523.68		91.81				91.81			132.50	2.50	
2-18	静力压桩机（液压）	大	压力（t）	120	11	1913.85	815.28	180.48	662.34		123.25				123.25			132.50	2.50	
2-19	静力压桩机（液压）	大	压力（t）	160	11	2457.54	998.33	233.53	959.82		133.36				133.36			132.50	2.50	
2-20	静力压桩机（液压）	大	压力（t）	200	11	3643.72	1427.51	276.90	1138.07		668.74		77.76					132.50	2.50	
2-21	静力压桩机（液压）	大	压力（t）	300	11	4602.16	1811.30	376.75	1548.46		733.15		85.25					132.50	2.50	
2-22	静力压桩机（液压）	大	压力（t）	400	11	5460.32	2168.95	456.19	1874.93		827.75		96.25					132.50	2.50	
2-23	汽车式钻孔机	大	孔径（mm）	φ400	11	921.25	149.17	23.66	65.06		550.86	47.40			81.60			132.50	2.50	
2-24	汽车式钻孔机	大	孔径（mm）	φ1000	11	927.04	223.93	41.42	113.91		415.28		38.80		81.60			132.50	2.50	
2-25	汽车式钻孔机	大	孔径（mm）	φ2000	11	1328.85	295.58	44.15	121.42		735.20		76.00		81.60			132.50	2.50	
2-26	潜水钻机	大	孔径（mm）	φ800	11	457.11	79.61	16.95	45.61		182.44				182.44			132.50	2.50	
2-27	潜水钻机	大	孔径（mm）	φ1250	11	516.29	107.29	25.49	68.57		182.44				182.44			132.50	2.50	
2-28	潜水钻机	大	孔径（mm）	φ1500	11	622.37	158.17	30.56	82.21		218.93				218.93			132.50	2.50	
2-29	工程钻机	中	孔径（mm）	φ500	7	510.56	198.61	18.17	37.80		123.48				123.48			132.50	2.50	
2-30	工程钻机	大	孔径（mm）	φ800	7	647.51	308.36	20.91	43.49		142.25				142.25			132.50	2.50	
2-31	工程钻机	大	孔径（mm）	φ1500	7	707.18	308.36	24.55	51.05		190.72				190.72			132.50	2.50	
2-32	螺旋钻机	中	孔径（mm）	φ400	7	545.70	243.27	6.39	40.06		123.48				123.48			132.50	2.50	
2-33	螺旋钻机	中	孔径（mm）	φ600	7	652.86	285.81	7.33	45.95		181.27				181.27			132.50	2.50	
2-34	螺旋钻机	中	孔径（mm）	φ800	7	878.55	444.69	8.76	54.95		237.65				237.65			132.50	2.50	
2-35	短螺旋履带钻孔机	大	孔径（mm）	φ1200	11	1553.72	652.89	114.36	219.57		434.40		34.00		142.00			132.50	2.50	
2-36	冲击钻孔机	中	乌克斯		11	387.65	149.87	21.76	43.52		40.00				40.00			132.50	2.50	
2-37	锚杆钻孔机	大	DHR80A		11	2633.37	1450.67	197.63	353.77		498.80		58.00					132.50	2.50	
2-38	钻运立三用机	大	GH30-IC		11	1876.05	1303.91	49.30	46.34		344.00		40.00					132.50	2.50	
三、起重机械																				
3-1	履带式电动起重机	中	起重量（t）	3	10	119.58	69.61	5.66	3.31		41.00				41.00			66.25	1.25	
3-2	履带式电动起重机	中	起重量（t）	5	10	158.34	72.24	7.79	18.31		60.00				60.00			66.25	1.25	
3-3	履带式电动起重机	大	起重量（t）	40	10	1276.07	769.69	28.65	67.33		410.40				410.40			132.50	2.50	
3-4	履带式电动起重机	大	起重量（t）	50	10	1353.45	786.27	28.65	67.33		471.20				471.20			132.50	2.50	
3-5	履带式起重机	大	起重量（t）	10	10	519.27	213.65	18.65	71.97		215.00		25.00					132.50	2.50	
3-6	履带式起重机	大	起重量（t）	15	10	708.99	311.19	24.78	95.67		277.35		32.25					132.50	2.50	
3-7	履带式起重机	大	起重量（t）	20	10	752.96	320.48	40.12	73.82		318.54		37.04					132.50	2.50	
3-8	履带式起重机	大	起重量（t）	25	10	847.45	328.44	53.26	98.01		367.74		42.76					132.50	2.50	
3-9	履带式起重机	大	起重量（t）	30	10	1097.47	444.56	70.13	129.04		453.74		52.76					132.50	2.50	
3-10	履带式起重机	大	起重量（t）	40	10	1686.31	743.15	139.69	257.03		546.44		63.54					132.50	2.50	
3-11	履带式起重机	特	起重量（t）	50	10	1952.46	790.92	142.18	261.61		757.75		88.11					132.50	2.50	
3-12	履带式起重机	特	起重量（t）	60	10	2653.32	965.43	163.46	652.22		872.21		101.42					132.50	2.50	
3-13	履带式起重机	特	起重量（t）	70	10	2924.46	1091.49	173.13	690.79		969.05		112.68					132.50	2.50	
3-14	履带式起重机	特	起重量（t）	90	10	4377.33	2428.49	193.77	773.12		981.95		114.18					132.50	2.50	
3-15	履带式起重机	特	起重量（t）	100	10	4959.24	2886.32	216.87	865.33		990.72		115.20					132.50	2.50	
3-16	履带式起重机	特	起重量（t）	140	10	6718.94	4392.52	263.54	1051.52		1011.36		117.60					132.50	2.50	
3-17	履带式起重机	特	起重量（t）	150	10	6885.33	4432.33	275.00	1097.24		1080.76		125.67					132.50	2.50	
3-18	履带式起重机	特	起重量（t）	200	10	9128.59	6077.87	347.82	1387.79		1315.11		152.92					132.50	2.50	
3-19	履带式起重机	特	起重量（t）	300	10	13851.58	9948.86	581.87	1803.81		1517.04		176.40					198.75	3.75	
3-20	轮胎式起重机	大	起重量（t）	10	12	536.30	156.40	25.83	78.78		275.29		32.01					132.50	2.50	
3-21	轮胎式起重机	大	起重量（t）	16	12	666.78	236.45	29.30	89.37		311.66		36.24					132.50	2.50	
3-22	轮胎式起重机	大	起重量（t）	20	12	815.11	328.17	32.09	97.86		356.99		41.51					132.50	2.50	
3-23	轮胎式起重机	大	起重量（t）	25	12	1033.66	381.72	66.18	101.92		483.84		56.26					132.50	2.50	
3-24	轮胎式起重机	大	起重量（t）	40	12	1419.30	494.91	151.44	233.21		539.74		62.76					132.50	2.50	
3-25	轮胎式起重机	大	起重量（t）	50	12	1512.96	557.73	153.42	236.27		565.54		65.76					132.50	2.50	
3-26	轮胎式起重机	大	起重量（t）	60	12	1938.34	853.56	184.11	283.53		617.14		71.76					132.50	2.50	
3-27	汽车式起重机	中	起重量（t）	5	11	553.15	108.93	24.07	78.96		230.67	23.30						66.25	1.25	110.52
3-28	汽车式起重机	大	起重量（t）	8	11	686.76	148.83	39.71	82.20		244.50		28.43					132.50	2.50	171.52
3-29	汽车式起重机	大	起重量（t）	10	11	2138.02	177.21	47.40	98.12		256.02		29.77					132.50	2.50	1559.27
3-30	汽车式起重机	大	起重量（t）	12	11	874.00	218.05	56.32	116.58		262.73		30.55					132.50	2.50	220.32
3-31	汽车式起重机	大	起重量（t）	16	11	1139.04	301.11	84.85	175.65		308.31		35.85					132.50	2.50	269.12
3-32	汽车式起重机	大	起重量（t）	20	11	1437.80	357.18	144.78	299.69		330.33		38.41					132.50	2.50	305.82
3-33	汽车式起重机	大	起重量（t）	25	11	1566.79	379.34	159.50	330.17		350.28		40.73					132.50	2.50	347.50
3-34	汽车式起重机	大	起重量（t）	30	11	1686.05	411.87	168.30	348.39		378.40		44.00					132.50	2.50	379.09
3-35	汽车式起重机	大	起重量（t）	40	11	2571.56	631.30	346.37	716.98		417.27		48.52					132.50	2.50	459.64
3-36	汽车式起重机	大	起重量（t）	50	11	3524.93	1626.71	472.87	978.84		446.51		51.92					132.50	2.50	
3-37	汽车式起重机	特	起重量（t）	60	11	4049.79	1979.74	516.23	1068.61		485.21		56.42					132.50	2.50	
3-38	汽车式起重机	特	起重量（t）	70	11	4790.01	2595.82	547.31	1132.94		513.94		59.76					132.50	2.50	
3-39	汽车式起重机	特	起重量（t）	75	11	5004.31	2696.19	576.78	1193.93		537.41		62.49					132.50	2.50	
3-40	汽车式起重机	特	起重量（t）	80	11	5303.64	2872.70	611.49	1265.78		553.67		64.38					132.50	2.50	
3-41	汽车式起重机	特	起重量（t）	90	11	5635.63	3000.76	669.29	1385.42		580.16		67.46					132.50	2.50	
3-42	汽车式起重机	特	起重量（t）	100	11	6133.92	3288.04	722.33	1495.23		628.32		73.06					132.50	2.50	
3-43	汽车式起重机	特	起重量（t）	110	11	7411.13	4326.36	793.40	1642.33		649.04		75.47					132.50	2.50	
3-44	汽车式起重机	特	起重量（t）	120	11	8574.06	5316.23	839.77	1738.32		679.74		79.04					132.50	2.50	

3-45	汽车式起重机		特	起重量（t）	125	11	9113.48	5622.19	909.20	1882.05		700.04		81.40					132.50	2.50	
3-46	汽车式起重机		特	起重量（t）	136	11	10223.97	6403.02	983.81	2036.48		800.66		93.10					132.50	2.50	
3-47	汽车式起重机		特	起重量（t）	150	11	11162.07	6897.95	1106.03	2289.49		868.60		101.00					132.50	2.50	
3-48	龙门式起重机		中	起重量（t）	5	12	139.21	57.04	7.88	21.44		52.85				52.85			132.50	2.50	
3-49	龙门式起重机		中	起重量（t）	10	12	263.90	138.68	9.93	27.00		88.29				88.29			132.50	2.50	
3-50	龙门式起重机		大	起重量（t）	20	12	557.15	305.28	18.81	25.96		207.10				207.10			132.50	2.50	
3-51	龙门式起重机		大	起重量（t）	30	12	742.67	434.52	32.12	44.33		231.70				231.70			132.50	2.50	
3-52	龙门式起重机		大	起重量（t）	40	12	943.92	523.41	44.12	60.89		315.50				315.50			132.50	2.50	
3-53	龙门式起重机		大	起重量（t）	50	12	1300.17	833.37	53.28	73.52		340.00				340.00			132.50	2.50	
3-54	门座吊		大	起重量（t）	30	12	1220.26	934.41	38.58	36.27		211.00				211.00			132.50	2.50	
3-55	门座吊		特	起重量（t）	60	12	1935.27	1302.21	66.63	62.63		503.80				503.80			132.50	2.50	
3-56	叉式起重机		中	起重量（t）	3	12	386.71	60.30	14.42	50.04		261.95	26.46						66.25	1.25	
3-57	叉式起重机		中	起重量（t）	5	12	407.05	93.57	17.53	60.83		235.12		27.34					66.25	1.25	
3-58	叉式起重机		中	起重量（t）	6	12	447.19	115.11	18.88	65.52		247.68		28.80					66.25	1.25	
3-59	叉式起重机		大	起重量（t）	10	12	615.38	154.58	31.41	160.21		269.18		31.30					66.25	1.25	
3-60	塔式起重机		中	起重量（t）	2	14	153.16	76.94	6.89	27.13		42.20				42.20			132.50	2.50	
3-61	塔式起重机		大	起重量（t）	6	14	420.28	247.35	23.88	94.10		54.95				54.95			132.50	2.50	
3-62	塔式起重机		大	起重量（t）	8	14	472.11	277.50	25.27	99.56		69.78				69.78			132.50	2.50	
3-63	塔式起重机		大	起重量（t）	15	14	779.29	404.94	54.44	214.50		105.41				105.41			132.50	2.50	
3-64	塔式起重机		大	起重量（t）	25	14	1445.22	953.11	63.05	248.41		180.65				180.65			132.50	2.50	
3-65	塔式起重机		大	起重量（t）	40	14	1831.63	1062.41	97.40	383.77		288.05				288.05			132.50	2.50	
3-66	塔式起重机		特	起重量（t）	60	14	2739.29	1733.78	116.01	457.09		432.41				432.41			132.50	2.50	
3-67	塔式起重机		特	起重量（t）	80	14	3056.22	1832.72	131.07	516.43		576.00				576.00			132.50	2.50	
3-68	塔式起重机		特	起重量（t）	125	14	4741.57	2852.73	200.17	788.67		900.00				900.00			132.50	2.50	
3-69	自升式塔式起重机		大	起重力矩（t·m）	100	14	688.58	387.27	42.35	88.94		170.02				170.02			132.50	2.50	
3-70	自升式塔式起重机		大	起重力矩（t·m）	125	14	747.42	393.40	54.27	113.97		185.78				185.78			132.50	2.50	
3-71	自升式塔式起重机		大	起重力矩（t·m）	145	14	870.75	464.54	67.08	140.88		198.25				198.25			132.50	2.50	
3-72	自升式塔式起重机		大	起重力矩（t·m）	200	14	1030.31	527.67	85.86	180.31		236.47				236.47			132.50	2.50	
3-73	自升式塔式起重机		特	起重力矩（t·m）	300	14	1370.55	746.75	105.87	222.33		295.60				295.60			132.50	2.50	
3-74	自升式塔式起重机		特	起重力矩（t·m）	450	14	1680.05	1031.19	109.44	229.82		309.60				309.60			198.75	3.75	
3-75	电动双梁起重机		中	起重量（t）	5	10	141.17	67.36	11.71	25.40		36.70				36.70			66.25	1.25	
3-76	电动双梁起重机		中	起重量（t）	10	10	234.28	124.61	19.52	42.35		47.80				47.80			66.25	1.25	
3-77	桅杆式起重机		中	起重量（t）	5	14	179.34	64.36	4.91	20.60	18.37	71.10				71.10			132.50	2.50	
3-78	桅杆式起重机		中	起重量（t）	10	14	236.44	80.76	5.48	23.03	18.37	108.80				108.80			132.50	2.50	
3-79	桅杆式起重机		中	起重量（t）	15	14	347.68	107.47	7.12	29.92	18.37	184.80				184.80			132.50	2.50	
3-80	桅杆式起重机		大	起重量（t）	40	14	512.87	135.59	10.31	43.32	57.05	266.60				266.60			132.50	2.50	
3-81	平台吊		小	起重量（t）	0.75	9	38.49	10.02	0.40	3.24	4.93	19.90				19.90			66.25	1.25	
3-82	少先吊		小	起重量（t）	1	9	32.47	7.90	0.40	3.24	4.93	16.00				16.00			66.25	1.25	

四、水平运输机械

4-1	载重汽车		中	载重量（t）	2.5	8	371.12	38.82	5.82	32.65		201.56	20.36						66.25	1.25	26.02
4-2	载重汽车		中	载重量（t）	4	8	453.89	44.86	7.84	43.96		252.25	25.48						66.25	1.25	38.73
4-3	载重汽车		中	载重量（t）	5	8	503.77	51.33	8.76	49.17		276.83		32.19					66.25	1.25	51.43
4-4	载重汽车		中	载重量（t）	6	8	532.19	61.68	9.96	55.87		276.83		32.19					66.25	1.25	61.60
4-5	载重汽车		大	载重量（t）	8	8	651.73	99.21	20.11	79.02		305.21		35.49					66.25	1.25	81.93
4-6	载重汽车		大	载重量（t）	10	8	855.70	160.04	23.66	92.97		344.26		40.03					132.50	2.50	102.27
4-7	载重汽车		大	载重量（t）	12	8	973.99	190.66	27.30	107.28		397.92		46.27					132.50	2.50	118.33
4-8	载重汽车		大	载重量（t）	15	8	1133.69	225.17	31.17	122.51		487.96		56.74					132.50	2.50	134.38
4-9	自卸汽车		中	载重量（t）	2	8	344.89	55.40	5.47	24.29		170.97	17.27						66.25	1.25	22.51
4-10	自卸汽车		中	载重量（t）	4	8	555.78	76.97	10.53	46.74		310.27	31.34						66.25	1.25	45.02
4-11	自卸汽车		中	载重量（t）	6	8	648.26	116.68	15.86	70.43		311.84		36.26					66.25	1.25	67.20
4-12	自卸汽车		大	载重量（t）	8	8	763.17	142.18	26.12	87.24		352.00		40.93					66.25	1.25	89.38
4-13	自卸汽车		大	载重量（t）	10	8	946.48	201.01	29.95	100.02		371.43		43.19					132.50	2.50	111.57
4-14	自卸汽车		大	载重量（t）	12	8	1009.60	211.30	30.27	101.11		400.67		46.59					132.50	2.50	133.75
4-15	自卸汽车		大	载重量（t）	15	8	1294.21	367.70	39.59	132.20		455.20		52.93					132.50	2.50	167.02
4-16	自卸汽车		大	载重量（t）	20	8	1522.65	443.20	51.86	173.20		519.44		60.40					132.50	2.50	202.45
4-17	平板拖车组		大	载重量（t）	8	9	709.94	107.72	21.05	56.41		297.50	30.05						132.50	2.50	94.76
4-18	平板拖车组		大	载重量（t）	10	9	810.24	128.24	24.56	65.81		351.45	35.50						132.50	2.50	107.68
4-19	平板拖车组		大	载重量（t）	15	9	1103.16	206.29	30.73	145.37		441.44	44.59						132.50	2.50	146.83
4-20	平板拖车组		大	载重量（t）	20	9	1247.24	334.53	36.98	174.91		390.35		45.39					132.50	2.50	177.97
4-21	平板拖车组		大	载重量（t）	25	9	1398.04	390.28	44.86	212.20		422.52		49.13					132.50	2.50	195.68
4-22	平板拖车组		大	载重量（t）	30	9	1535.88	457.19	49.29	233.13		450.38		52.37					132.50	2.50	213.39
4-23	平板拖车组		大	载重量（t）	40	9	1870.50	615.53	60.18	284.66		493.38		57.37					132.50	2.50	284.25
4-24	平板拖车组		大	载重量（t）	50	9	2037.22	652.33	62.97	297.84		536.47		62.38					132.50	2.50	355.11
4-25	平板拖车组		大	载重量（t）	60	9	2231.76	702.51	64.87	306.83		599.08		69.66					132.50	2.50	425.97
4-26	平板拖车组		大	载重量（t）	80	9	2540.06	1226.61	79.25	374.83		726.87		84.52					132.50	2.50	
4-27	平板拖车组		大	载重量（t）	100	9	3029.07	1393.87	80.54	511.42		910.74		105.90					132.50	2.50	
4-28	平板拖车组		大	载重量（t）	150	9	4299.81	1984.88	105.62	670.71		1406.10		163.50					132.50	2.50	
4-29	管子拖车		大	载重量（t）	24	8	2020.67	475.56	44.39	179.33		1053.50		122.50					132.50	2.50	135.39
4-30	管子拖车		大	载重量（t）	27	8	2272.85	642.25	57.89	233.87		1053.50		122.50					132.50	2.50	152.84
4-31	管子拖车		大	载重量（t）	35	8	2482.40	754.03	68.34	276.10		1053.50		122.50					132.50	2.50	197.93
4-32	长材运输车		大	载重量（t）	8	8	701.12	166.00	16.26	93.82		358.79		41.72					66.25	1.25	
4-33	长材运输车		大	载重量（t）	12	8	930.61	274.28	20.24	116.76		386.83		44.98					132.50	2.50	
4-34	长材运输车		大	载重量（t）	15	8	1254.55	332.54	21.78	125.69		443.42		51.56					132.50	2.50	198.62
4-35	壁板运输车		大	载重量（t）	8	6	710.34	164.20	16.59	84.96		312.09		36.29					132.50	2.50	
4-36	壁板运输车		大	载重量（t）	15	6	1129.85	424.75	21.78	111.53		439.29		51.08					132.50	2.50	
4-37	自装自卸运输车		大	载重量（t）	6	8	755.92	115.43	18.23	77.84		478.17	48.30						66.25	1.25	
4-38	自装自卸运输车		大	载重量（t）	8	8	857.79	160.50	19.18	81.91		529.95	53.53						66.25	1.25	
4-39	机动翻斗车		小	载重量（t）	1	6	164.44	19.62	4.62	18.15	3.94	51.86		6.03					66.25	1.25	
4-40	机动翻斗车		中	载重量（t）	1.5	6	208.42	27.95	5.33	20.93	3.94	84.02		9.77					66.25	1.25	
4-41	油罐车		中	罐容量（L）	5000	8	560.00	87.14	10.18	51.82		303.34	30.64						66.25	1.25	41.27
4-42	油罐车		大	罐容量（L）	8000	8	662.84	116.47	17.65	89.86		290.68		33.80					66.25	1.25	81.93
4-43	洒水车		中	罐容量（L）	4000	8	552.22	81.96	12.50	53.64		296.60	29.96						66.25	1.25	41.27
4-44	洒水车		大	罐容量（L）	8000	8	659.87	110.86	22.12	94.91		283.80		33.00					66.25	1.25	81.93
4-45	轨道拖车头		中	功率（kW）	30	11	267.15	114.24	9.09	19.09		58.48		6.80					66.25	1.25	
4-46	轨道平车		小	载重量（t）	5	8	21.05	13.68	2.38	4.99											
4-47	轨道平车		中	载重量（t）	10	8	70.46	60.64	3.17	6.65											

五、垂直运输机械

5-1	电动卷扬机	单筒快速	小	牵引力（t）	0.5	10	91.87	1.84	1.19	3.19	4.70	14.70				14.70			66.25	1.25	
5-2	电动卷扬机	单筒快速	小	牵引力（t）	1	10	115.81	4.39	2.06	5.51	4.70	32.90				32.90			66.25	1.25	
5-3	电动卷扬机	单筒快速	小	牵引力（t）	1.5	10	137.06	6.02	2.94	7.85	4.70	49.30				49.30			66.25	1.25	
5-4	电动卷扬机	单筒快速	小	牵引力（t）	2	10	159.48	8.21	3.60	9.62	4.70	67.10				67.10			66.25	1.25	
5-5	电动卷扬机	双筒快速	小	牵引力（t）	1	10	176.71	12.18	2.20	5.88	4.70	85.50				85.50			66.25	1.25	
5-6	电动卷扬机	双筒快速	小	牵引力（t）	3	10	210.78	25.00	4.31	11.52	4.70	99.00				99.00			66.25	1.25	
5-7	电动卷扬机	双筒快速	小	牵引力（t）	5	10	245.89	30.80	4.94	13.20	4.70	126.00				126.00			66.25	1.25	
5-8	电动卷扬机	单筒慢速	小	牵引力（t）	3	10	128.51	8.57	4.22	11.27	4.70	33.50				33.50			66.25	1.25	
5-9	电动卷扬机	单筒慢速	小	牵引力（t）	5	10	133.21	10.76	4.88	13.02	4.70	33.60				33.60			66.25	1.25	
5-10	电动卷扬机	单筒慢速	小	牵引力（t）	8	10	198.58	31.30	6.08	27.25	4.70	63.00				63.00			66.25	1.25	
5-11	电动卷扬机	单筒慢速	中	牵引力（t）	10	10	275.60	103.38	6.98	31.29	4.70	63.00				63.00			66.25	1.25	
5-12	电动卷扬机	单筒慢速	中	牵引力（t）	20	10	509.26	164.28	10.72	64.02	17.49	186.50				186.50			66.25	1.25	
5-13	电动卷扬机	单筒慢速	中	牵引力（t）	30	10	782.70	227.30	11.63	69.43	17.49	390.60				390.60			66.25	1.25	
5-14	电动卷扬机	双筒慢速	小	牵引力（t）	3	10	147.55	24.43	4.48	12.49	4.70	35.20				35.20			66.25	1.25	
5-15	电动卷扬机	双筒慢速	小	牵引力（t）	5	10	171.21	29.39	5.19	14.48	4.70	51.20				51.20			66.25	1.25	
5-16	电动卷扬机	双筒慢速	小	牵引力（t）	8	10	231.27	38.38	6.11	45.43	4.70	70.40				70.40			66.25	1.25	

5-17	电动卷扬机	双筒慢速	中	牵引力（t）	10	10	300.00	99.49	7.01	52.15	4.70	70.40				70.40			66.25	1.25	
5-18	卷扬机带塔 （H=40m）		中	牵引力（t）	3~5	10	177.08	31.51	7.45	20.78	17.49	33.60				33.60			66.25	1.25	
5-19	皮带运输机		小	水平运输距离（m）	10	10	136.68	17.74	4.68	16.43	13.08	18.50				18.50			66.25	1.25	
5-20	皮带运输机		小	水平运输距离（m）	15	10	144.51	20.92	5.25	18.43	13.08	20.58				20.58			66.25	1.25	
5-21	皮带运输机		小	水平运输距离（m）	20	10	157.81	25.58	5.85	20.52	13.08	26.53				26.53			66.25	1.25	
5-22	皮带运输机		小	水平运输距离（m）	30	10	172.68	27.86	7.00	24.58	13.08	33.91				33.91			66.25	1.25	
5-23	单笼施工电梯		中	提升高度（m）	75	12	300.29	129.34	19.68	39.36		45.66				45.66			66.25	1.25	
5-24	单笼施工电梯		中	提升高度（m）	100	12	322.68	142.00	22.92	45.85		45.66				45.66			66.25	1.25	
5-25	单笼施工电梯		中	提升高度（m）	130	12	358.11	158.52	29.59	44.39		59.36				59.36			66.25	1.25	
5-26	双笼施工电梯		中	提升高度（m）	100	12	431.13	177.23	35.26	70.53		81.86				81.86			66.25	1.25	
5-27	双笼施工电梯		大	提升高度（m）	200	12	544.08	195.76	48.85	73.28		159.94				159.94			66.25	1.25	
5-28	电动葫芦	单速	小	起重量（t）	2	8	40.40	11.90	2.23	7.37		18.90				18.90					
5-29	电动葫芦	单速	小	起重量（t）	3	8	48.76	18.37	2.67	8.82		18.90				18.90					
5-30	电动葫芦	单速	小	起重量（t）	5	8	68.38	23.10	3.88	12.80		28.60				28.60					
5-31	电动葫芦	双速	小	起重量（t）	10	8	124.05	40.07	8.60	22.53		52.85				52.85					
5-32	电动葫芦	双速	小	起重量（t）	20	8	195.73	58.62	9.78	25.63		101.70				101.70					
5-33	电动葫芦	双速	小	起重量（t）	30	8	262.54	79.79	10.55	27.63		144.57				144.57					
六、混凝土及砂浆机械																					
6-1	滚筒式混凝土搅拌机	电动	小	出料容量（L）	250	10	121.79	18.54	3.03	7.58	5.48	20.91				20.91			66.25	1.25	
6-2	滚筒式混凝土搅拌机	电动	中	出料容量（L）	400	10	140.58	33.29	3.79	7.39	5.48	24.38				24.38			66.25	1.25	
6-3	滚筒式混凝土搅拌机	电动	中	出料容量（L）	500	10	156.11	41.97	4.42	8.63	5.48	29.36				29.36			66.25	1.25	
6-4	滚筒式混凝土搅拌机	内燃	小	出料容量（L）	250	10	153.47	9.19	3.75	9.37	5.48	59.43		6.91					66.25	1.25	
6-5	滚筒式混凝土搅拌机	内燃	中	出料容量（L）	500	10	250.57	43.08	5.73	11.18	5.48	118.85		13.82					66.25	1.25	
6-6	涡浆式混凝土搅拌机		小	出料容量（L）	250	10	143.25	24.10	3.94	9.38	5.48	34.10				34.10			66.25	1.25	
6-7	涡浆式混凝土搅拌机		小	出料容量（L）	350	10	205.10	34.64	6.49	15.44	5.48	76.80				76.80			66.25	1.25	
6-8	涡浆式混凝土搅拌机		中	出料容量（L）	500	10	273.03	60.26	9.86	23.47	5.48	107.71				107.71			66.25	1.25	
6-9	涡浆式混凝土搅拌机		中	出料容量（L）	1000	10	427.97	122.21	12.47	29.68	20.41	176.95				176.95			66.25	1.25	
6-10	双锥反转出料混凝土搅拌机		小	出料容量（L）	200	10	118.18	12.47	2.93	7.75	5.48	23.30				23.30			66.25	1.25	
6-11	双锥反转出料混凝土搅拌机		小	出料容量（L）	350	10	147.61	19.81	3.45	9.10	5.48	43.52				43.52			66.25	1.25	
6-12	双锥反转出料混凝土搅拌机		中	出料容量（L）	500	10	176.99	35.48	5.56	9.18	5.48	55.04				55.04			66.25	1.25	
6-13	双锥反转出料混凝土搅拌机		中	出料容量（L）	750	10	241.02	52.25	8.10	13.37	20.41	80.64				80.64			66.25	1.25	
6-14	单卧轴式混凝土搅拌机		小	出料容量（L）	150	10	134.28	13.65	3.05	12.31	5.48	33.54				33.54			66.25	1.25	
6-15	单卧轴式混凝土搅拌机		小	出料容量（L）	250	10	160.74	24.02	3.55	14.34	5.48	47.10				47.10			66.25	1.25	
6-16	单卧轴式混凝土搅拌机		小	出料容量（L）	350	10	195.87	34.98	4.89	19.76	5.48	64.51				64.51			66.25	1.25	
6-17	双卧轴式混凝土搅拌机		小	出料容量（L）	350	10	235.96	34.13	5.15	24.39	5.48	100.56				100.56			66.25	1.25	
6-18	双卧轴式混凝土搅拌机		中	出料容量（L）	400	10	248.15	41.72	5.48	25.95	5.48	103.27				103.27			66.25	1.25	
6-19	双卧轴式混凝土搅拌机		中	出料容量（L）	500	10	270.48	51.83	5.94	28.14	5.48	112.84				112.84			66.25	1.25	
6-20	双卧轴式混凝土搅拌机		中	出料容量（L）	800	10	353.99	89.34	11.01	31.50	20.41	135.48				135.48			66.25	1.25	
6-21	双卧轴式混凝土搅拌机		中	出料容量（L）	1000	10	416.92	126.42	13.55	38.74	20.41	151.55				151.55			66.25	1.25	
6-22	双卧轴式混凝土搅拌机		中	出料容量（L）	1500	10	491.86	161.82	14.90	42.62	20.41	185.86				185.86			66.25	1.25	
6-23	泡沫混凝土搅拌机		小	出料容量（L）	500	10	108.30	9.96	1.63	4.07	5.48	20.91				20.91			66.25	1.25	
6-24	灰浆搅拌机		小	出料容量（L）	200	10	87.58	3.58	0.73	2.93	5.48	8.61				8.61			66.25	1.25	
6-25	灰浆搅拌机		小	出料容量（L）	400	10	98.76	5.83	1.21	4.82	5.48	15.17				15.17			66.25	1.25	
6-26	散装水泥车		中	载重量（t）	4	9	453.79	54.84	17.75	55.57		259.38	26.20						66.25	1.25	
6-27	散装水泥车		大	载重量（t）	7	9	572.69	123.98	29.09	91.07		262.30		30.50					66.25	1.25	
6-28	散装水泥车		大	载重量（t）	10	9	762.04	242.41	34.81	108.97		309.60		36.00					66.25	1.25	
6-29	散装水泥车		大	载重量（t）	26	9	1777.66	680.32	98.90	309.55		622.64		72.40					66.25	1.25	
6-30	混凝土搅拌输送车		大	容量（m3）	3	8	901.55	273.87	60.88	250.81		249.74		29.04					66.25	1.25	
6-31	混凝土搅拌输送车		大	容量（m3）	4	8	1079.77	338.58	72.08	296.96		305.90		35.57					66.25	1.25	
6-32	混凝土搅拌输送车		大	容量（m3）	5	8	1266.29	414.55	82.77	341.00		361.72		42.06					66.25	1.25	
6-33	混凝土搅拌输送车		大	容量（m3）	6	8	1502.52	483.02	93.80	386.45		473.00		55.00					66.25	1.25	
6-34	混凝土搅拌输送车		大	容量（m3）	7	8	1838.99	648.09	105.94	436.46		516.00		60.00					132.50	2.50	
6-35	混凝土输送泵车		大	排出量（m3/h）	30	8	1266.21	582.44	46.85	127.91		376.51		43.78					132.50	2.50	
6-36	混凝土输送泵车		大	排出量（m3/h）	60	8	1743.76	689.36	79.01	215.69		627.20		72.93					132.50	2.50	
6-37	混凝土输送泵车		大	排出量（m3/h）	75	8	2117.84	895.69	98.76	269.61		721.28		83.87					132.50	2.50	
6-38	混凝土输送泵车		大	排出量（m3/h）	85	8	2580.82	1246.47	111.94	305.59		784.32		91.20					132.50	2.50	
6-39	混凝土输送泵车		大	排出量（m3/h）	90	8	3985.57	2325.99	245.89	472.10		809.09		94.08					132.50	2.50	
6-40	混凝土输送泵车		大	排出量（m3/h）	100	8	4621.76	2710.34	268.45	515.42		928.80		108.00					198.75	3.75	
6-41	混凝土输送泵		中	泵出量（m3/h）	10	5	430.35	177.70	21.99	49.04	18.37	97.00				97.00			66.25	1.25	
6-42	混凝土输送泵		中	泵出量（m3/h）	20	5	631.14	248.78	37.69	84.05	18.37	176.00				176.00			66.25	1.25	
6-43	混凝土输送泵		大	泵出量（m3/h）	30	5	782.28	297.54	59.70	133.12	18.37	207.30				207.30			66.25	1.25	
6-44	混凝土输送泵		大	泵出量（m3/h）	45	5	1109.90	493.76	104.34	145.04	57.05	243.46				243.46			66.25	1.25	
6-45	混凝土输送泵		大	泵出量（m3/h）	60	5	1639.91	743.85	177.81	247.15	57.05	347.80				347.80			66.25	1.25	
6-46	混凝土输送泵		大	泵出量（m3/h）	80	5	2126.38	923.40	256.13	356.02	57.05	467.53				467.53			66.25	1.25	
6-47	灰浆输送泵		小	输送量（m3/h）	3	5	130.33	18.91	2.85	13.69	4.93	23.70				23.70			66.25	1.25	
6-48	灰浆输送泵		小	输送量（m3/h）	4	5	143.77	23.48	3.36	16.11	4.93	29.64				29.64			66.25	1.25	
6-49	灰浆输送泵		小	输送量（m3/h）	5	5	157.50	25.64	4.33	20.77	4.93	35.58				35.58			66.25	1.25	
6-50	灰气联合泵		小	出灰量（m3/h）	3.5	5	123.88	11.68	2.99	14.33	4.93	23.70				23.70			66.25	1.25	
6-51	混凝土喷射机		小	生产率（m3/h）	5	5	217.70	41.25	4.66	18.96	4.93	15.40				15.40			132.50	2.50	
6-52	筛洗石子机		小	洗石量（m3/h）	10	6	110.35	14.23	2.66	6.73	5.48	15.00				15.00			66.25	1.25	
6-53	混凝土震动台		小	台面尺寸（m）	1.5×6	10	173.66	37.40	3.02	16.69		50.30				50.30			66.25	1.25	
6-54	混凝土震动台		中	台面尺寸（m）	2.4×6.2	10	311.20	71.16	5.36	29.63		138.80				138.80			66.25	1.25	
6-55	偏心式震动筛		小	能力（m3/h）	12~16	10	110.05	7.75	2.07	5.38		28.60				28.60			66.25	1.25	
6-56	混凝土搅拌站		大	生产能力（m3/h）	15	10	1126.19	225.67	28.77	76.53		198.97				198.97			596.25	11.25	
6-57	混凝土搅拌站		大	生产能力（m3/h）	25	10	1340.65	302.31	47.36	125.99		268.74				268.74			596.25	11.25	
6-58	混凝土搅拌站		大	生产能力（m3/h）	45	10	1645.40	447.08	83.98	134.37		383.72				383.72			596.25	11.25	
6-59	混凝土搅拌站		大	生产能力（m3/h）	50	10	1813.63	506.69	106.38	170.20		434.11				434.11			596.25	11.25	
6-60	混凝土搅拌站		大	生产能力（m3/h）	60	10	3289.90	1694.65	129.81	207.69		661.50				661.50			596.25	11.25	
七、加工机械																					
7-1	钢筋调直机		小	直径（mm）	φ14	10	44.18	21.56	2.11	5.62	2.99	11.90				11.90					
7-2	钢筋切断机		小	直径（mm）	φ40	10	53.80	10.71	1.47	6.53	2.99	32.10				32.10					
7-3	钢筋弯曲机		小	直径（mm）	φ40	10	29.69	7.41	1.06	5.43	2.99	12.80				12.80					
7-4	钢筋镦头机		小	直径（mm）	φ5	10	60.43	10.71	1.43	5.82		42.47				42.47					
7-5	预应力钢筋拉伸机		小	拉伸力（t）	60	10	37.66	15.32	1.52	5.55		15.27				15.27					
7-6	预应力钢筋拉伸机		小	拉伸力（t）	65	10	41.13	16.36	1.62	5.90		17.25				17.25					
7-7	预应力钢筋拉伸机		小	拉伸力（t）	85	10	55.25	20.07	1.95	7.09		26.14				26.14					
7-8	预应力钢筋拉伸机		小	拉伸力（t）	90	10	61.82	22.60	2.17	7.89		29.16				29.16					
7-9	预应力钢筋拉伸机		中	拉伸力（t）	120	10	83.04	30.78	2.86	10.42		38.98				38.98					
7-10	预应力钢筋拉伸机		中	拉伸力（t）	300	10	140.32	46.87	4.21	15.33		73.91				73.91					
7-11	预应力钢筋拉伸机		中	拉伸力（t）	500	10	297.54	136.06	10.91	39.70		110.87				110.87					
7-12	木工圆锯机		小	直径（mm）	φ500	9	36.79	7.97	0.90	1.93	1.99	24.00				24.00					
7-13	木工圆锯机		小	直径（mm）	φ600	9	46.44	8.16	0.98	2.11	1.99	33.20				33.20					
7-14	木工圆锯机		小	直径（mm）	φ1000	9	89.33	9.47	1.23	2.64	1.99	74.00				74.00					
7-15	木工带锯机		中	锯轮直径（mm）	φ1250	9	335.35	13.68	5.88	13.24		236.30				236.30			66.25	1.25	
7-16	木工平刨床		小	刨削宽度（mm）	300	10	17.09	3.84	0.62	2.37	1.66	8.60				8.60					

7-17	木工平刨床	小	刨削宽度（mm）	450	10	24.04	5.98	0.72	2.78	1.66	12.90				12.90					
7-18	木工压刨床	小	刨削宽度（mm）	单面600	10	41.96	6.62	1.81	4.93		28.60				28.60					
7-19	木工压刨床	小	刨削宽度（mm）	双面600	10	66.81	14.97	2.11	5.73		44.00				44.00					
7-20	木工压刨床	中	刨削宽度（mm）	三面400	10	79.87	17.78	2.99	6.70		52.40				52.40					
7-21	木工压刨床	中	刨削宽度（mm）	四面300	10	105.95	27.05	3.98	8.92		66.00				66.00					
7-22	木工开榫机	中	榫头长度（mm）	160	9	63.36	24.72	2.89	8.75		27.00				27.00					
7-23	木工打眼机	小	钻孔直径（mm）	φ50	9	12.57	4.00	0.72	3.15		4.70				4.70					
7-24	木工裁口机	小	宽度（mm）	多面400	10	52.28	8.51	1.95	5.82		36.00				36.00					
7-25	木工榫槽机	小	榫槽深度（mm）	100	9	43.36	5.16	1.27	5.43		31.50				31.50					
7-26	普通车床	小	工件直径×工件长度(mm)	φ400×1000	14	127.82	33.11	7.32	7.69		13.45				13.45			66.25	1.25	
7-27	普通车床	小	工件直径×工件长度(mm)	φ400×2000	14	150.53	39.58	10.70	11.23		22.77				22.77			66.25	1.25	
7-28	普通车床	中	工件直径×工件长度(mm)	φ630×1400	14	166.26	50.24	11.51	12.08		26.18				26.18			66.25	1.25	
7-29	普通车床	中	工件直径×工件长度(mm)	φ630×2000	14	181.11	56.77	13.62	14.30		30.17				30.17			66.25	1.25	
7-30	普通车床	中	工件直径×工件长度(mm)	φ650×2000	14	221.95	63.24	15.91	16.71		59.84				59.84			66.25	1.25	
7-31	普通车床	中	工件直径×工件长度(mm)	φ1000×5000	14	286.01	115.70	21.57	22.65		59.84				59.84			66.25	1.25	
7-32	管车床	小			14	126.10	15.68	8.57	9.00		26.60				26.60			66.25	1.25	
7-33	磨床	中	M131W		14	183.85	45.77	16.76	12.07		43.00				43.00			66.25	1.25	
7-34	龙门刨床	中	刨削宽度×长度（mm）	1000×3000	14	628.38	483.38	32.39	18.46		27.90				27.90			66.25	1.25	
7-35	龙门刨床	中	刨削宽度×长度（mm）	1000×4000	14	748.77	530.78	42.70	24.34		84.70				84.70			66.25	1.25	
7-36	龙门刨床	中	刨削宽度×长度（mm）	1000×6000	14	967.66	610.05	61.50	35.06		194.80				194.80			66.25	1.25	
7-37	牛头刨床	小	刨削长度（mm）	650	14	120.18	21.99	10.84	7.26		13.84				13.84			66.25	1.25	
7-38	立式铣床	中	台宽×台长（mm）	320×1250	14	170.46	67.34	8.64	6.83		21.40				21.40			66.25	1.25	
7-39	立式铣床	中	台宽×台长（mm）	350×1250	14	182.66	67.68	11.53	9.11		28.09				28.09			66.25	1.25	
7-40	卧式铣床	中	台宽×台长（mm）	400×1600	14	176.20	66.11	11.54	9.12		23.18				23.18			66.25	1.25	
7-41	立式钻床	小	钻孔直径（mm）	φ25	14	93.83	13.81	5.10	4.64		4.03				4.03			66.25	1.25	
7-42	立式钻床	小	钻孔直径（mm）	φ35	14	106.19	20.79	6.65	6.05		6.45				6.45			66.25	1.25	
7-43	立式钻床	小	钻孔直径（mm）	φ50	14	122.85	33.39	6.94	6.32		9.95				9.95			66.25	1.25	
7-44	台式钻床	小	钻孔直径（mm）	φ16	14	76.15	1.71	0.86	1.59	1.76	3.98				3.98			66.25	1.25	
7-45	摇臂钻床	小	钻孔直径（mm）	φ25	14	100.38	17.18	7.92	4.36		4.67				4.67			66.25	1.25	
7-46	摇臂钻床	中	钻孔直径（mm）	φ50	14	125.43	34.01	9.87	5.43		9.87				9.87			66.25	1.25	
7-47	摇臂钻床	中	钻孔直径（mm）	φ63	14	144.52	43.40	11.48	6.32		17.07				17.07			66.25	1.25	
7-48	剪扳机	中	厚度×宽度（mm）	6.3×2000	14	167.03	59.44	8.30	4.40		28.64				28.64			66.25	1.25	
7-49	剪扳机	中	厚度×宽度（mm）	13×3000	14	228.73	95.85	10.02	5.31		51.30				51.30			66.25	1.25	
7-50	剪扳机	中	厚度×宽度（mm）	20×2000	14	273.78	144.98	12.61	6.68		43.26				43.26			66.25	1.25	
7-51	剪扳机	中	厚度×宽度（mm）	20×2500	14	303.22	157.35	14.54	7.71		57.37				57.37			66.25	1.25	
7-52	剪扳机	中	厚度×宽度（mm）	20×4000	14	470.01	281.72	18.72	9.92		93.40				93.40			66.25	1.25	
7-53	剪扳机	大	厚度×宽度（mm）	32×4000	14	670.15	441.56	22.18	11.76		128.40				128.40			66.25	1.25	
7-54	剪扳机	大	厚度×宽度（mm）	40×3100	14	814.24	598.46	29.24	15.49		104.80				104.80			66.25	1.25	
7-55	型钢剪板机	中	剪断宽度（mm）	500	14	229.85	93.10	8.78	8.52		53.20				53.20			66.25	1.25	
7-56	钢材电动煨弯机	中	弯曲直径（mm）	φ500~180	14	152.79	106.60	6.97	4.81	2.30	32.11				32.11					
7-57	弯管机	中	直径（mm）	φ108	12	87.27	44.13	5.11	5.93		32.10				32.10					
7-58	弯管机（带胎芯空压机	）中	PB16-30		12	1564.40	1033.18	156.75	329.17		45.30				45.30					
7-59	液压弯管机	中	弯曲能力（mm）	φ60	12	145.09	43.10	2.98	3.46	2.30	27.00				27.00			66.25	1.25	
7-60	板料校平机	大	厚度×宽度（mm）	10×2000	13	1284.02	1051.10	58.07	30.20		78.40				78.40			66.25	1.25	
7-61	板料校平机	大	厚度×宽度（mm）	16×2500	13	1931.11	1623.68	79.33	41.25		120.60				120.60			66.25	1.25	
7-62	卷板机	中	板厚×宽度（mm）	2×1600	14	125.60	22.06	4.91	3.78		28.60				28.60			66.25	1.25	
7-63	卷板机	中	板厚×宽度（mm）	20×2500	14	235.60	83.48	12.30	9.47		64.10				64.10			66.25	1.25	
7-64	卷板机	大	板厚×宽度（mm）	30×2000	14	330.45	168.01	13.67	10.52		72.00				72.00			66.25	1.25	
7-65	卷板机	大	板厚×宽度（mm）	40×3500	14	1247.68	840.07	58.11	44.75		238.50				238.50			66.25	1.25	
7-66	卷板机	大	板厚×宽度（mm）	45×3500	14	1919.69	1404.51	104.20	80.23		264.50				264.50			66.25	1.25	
7-67	联合冲剪机	中	板厚（mm）	16	11	178.91	78.90	10.23	10.53		13.00				13.00			66.25	1.25	
7-68	折方机	中	厚度×宽度（mm）	4×2000	12	56.62	31.08	8.97	3.77		12.80				12.80					
7-69	刨边机	大	加工长度（mm）	9000	14	683.38	407.99	32.36	34.63		75.90				75.90			132.50	2.50	
7-70	刨边机	大	加工长度（mm）	12000	14	786.42	485.52	44.69	47.81		75.90				75.90			132.50	2.50	
7-71	管子切断机	小	直径（mm）	φ60	12	21.41	7.25	1.86	5.20	2.30	4.80				4.80					
7-72	管子切断机	小	直径（mm）	φ150	12	50.86	21.76	4.50	9.40	2.30	12.90				12.90					
7-73	管子切断机	小	直径（mm）	φ250	12	65.92	23.72	5.63	11.77	2.30	22.50				22.50					
7-74	切管机	中	9A151		12	146.32	63.71	15.60	32.60	2.30	32.11				32.11					
7-75	螺栓套丝机	小	直径（mm）	φ39	12	42.93	5.28	3.83	6.52	2.30	25.00				25.00					
7-76	管子切断套丝机	小	直径（mm）	φ159	12	24.21	7.24	2.63	8.68	2.30	3.36				3.36					
7-77	咬口机	小	板厚（mm）	1.5	8	86.78	4.67	0.81	2.25		12.80				12.80			66.25	1.25	
7-78	坡口机	小	功率（Kw）	2.8	8	31.66	15.59	1.88	4.50	2.99	6.70				6.70					
7-79	弓锯床	小	锯料直径（mm）	φ250	12	35.71	19.62	3.24	4.89	2.99	4.97				4.97					
7-80	手提圆锯机	小			9	23.19	7.86	1.40	4.14	1.99	7.80				7.80					
7-81	台式砂轮机	小	砂轮直径（mm）	φ250	8	18.43	3.21		8.03	2.49	4.70				4.70					
7-82	法兰卷圆机	小	L40×4（mm）		8	42.28	16.12	2.47	7.90	2.99	12.80				12.80					
7-83	电锤	小	功率（Kw）	520	8	9.94	2.26		4.52	1.76	1.40				1.40					
7-84	摩擦压力机	中	压力（t）	160	13	274.30	79.57	7.92	12.51		41.80				41.80			132.50	2.50	
7-85	摩擦压力机	中	压力（t）	300	13	385.22	123.23	12.79	20.20		96.50				96.50			132.50	2.50	
7-86	可倾压力机	中	压力（t）	63	10	150.58	60.71	4.05	6.77		12.80				12.80			66.25	1.25	
7-87	可倾压力机	中	压力（t）	80	10	182.05	75.65	5.75	9.60		24.80				24.80			66.25	1.25	
7-88	可倾压力机	中	压力（t）	125	10	210.28	90.71	6.86	11.46		35.00				35.00			66.25	1.25	
7-89	空气锤	小	锤重（kg）	75	11	114.82	15.19	4.03	5.15		24.20				24.20			66.25	1.25	
7-90	空气锤	小	锤重（kg）	150	11	156.91	26.09	4.33	5.54		54.70				54.70			66.25	1.25	
7-91	空气锤	中	锤重（kg）	400	11	294.97	84.71	6.67	8.54		128.80				128.80			66.25	1.25	

八、泵类机械																				
8-1	电动单级离心清水泵	小	出口直径（mm）	φ50	10	99.85	2.66	1.60	3.85	2.49	23.00				23.00			66.25	1.25	
8-2	电动单级离心清水泵	小	出口直径（mm）	φ100	10	138.95	8.62	2.81	6.78	2.49	52.00				52.00			66.25	1.25	
8-3	电动单级离心清水泵	小	出口直径（mm）	φ150	10	185.43	11.08	5.28	12.73	2.49	87.60				87.60			66.25	1.25	
8-4	电动单级离心清水泵	小	出口直径（mm）	φ200	10	208.62	15.12	6.09	14.67	2.49	104.00				104.00			66.25	1.25	
8-5	电动单级离心清水泵	小	出口直径（mm）	φ250	10	513.30	35.81	8.72	21.03	2.49	379.00				379.00			66.25	1.25	
8-6	内燃单级离心清水泵	小	出口直径（mm）	φ50	10	113.69	3.49	2.94	5.26	2.49	33.26	3.36						66.25	1.25	
8-7	内燃单级离心清水泵	小	出口直径（mm）	φ100	10	153.37	7.44	3.82	6.84	2.49	66.53	6.72						66.25	1.25	
8-8	内燃单级离心清水泵	小	出口直径（mm）	φ150	10	192.70	14.75	7.35	13.16	2.49	88.70	8.96						66.25	1.25	
8-9	内燃单级离心清水泵	小	出口直径（mm）	φ200	10	219.00	18.59	7.45	13.34	2.49	110.88	11.20						66.25	1.25	
8-10	内燃单级离心清水泵	小	出口直径（mm）	φ250	10	252.17	39.78	7.77	13.91	2.49	121.97	12.32						66.25	1.25	
8-11	电动多级离心清水泵	小	出口直径（mm）	φ50	10	132.88	7.26	3.04	7.84	2.49	46.00				46.00			66.25	1.25	
8-12	电动多级离心清水泵	小	出口直径(mm)φ100	扬程(m)120以下	10	280.77	11.97	5.49	14.17	2.49	180.40				180.40			66.25	1.25	
8-13	电动多级离心清水泵	小	出口直径(mm)φ100	扬程(m)120以上	10	371.08	15.96	7.12	18.36	2.49	260.90				260.90			66.25	1.25	
8-14	电动多级离心清水泵	小	出口直径(mm)φ150	扬程(m)180以下	10	750.27	33.71	10.76	27.76	2.49	609.30				609.30			66.25	1.25	
8-15	电动多级离心清水泵	小	出口直径(mm)φ150	扬程(m)180以上	10	153.58	43.63	11.51	29.70	2.49					1100.20			66.25	1.25	
8-16	电动多级离心清水泵	小	出口直径(mm)φ200	扬程(m)280以下	10	1858.31	53.53	12.86	33.18	2.49	1690.00				1690.00			66.25	1.25	
8-17	电动多级离心清水泵	小	出口直径(mm)φ200	扬程(m)280以上	10	3232.00	56.51	16.41	42.34	2.49	3048.00				3048.00			66.25	1.25	
8-18	单级自吸水泵	小	出口直径（mm）	φ150	10	372.29	28.25	3.05	6.15	1.99	266.60		31.00					66.25	1.25	
8-19	污水泵	小	出口直径（mm）	φ70	7	168.13	5.20	1.06	3.43	2.49	89.70				89.70			66.25	1.25	
8-20	污水泵	小	出口直径（mm）	φ100	7	209.14	9.88	1.30	4.22	2.49	125.00				125.00			66.25	1.25	
8-21	污水泵	小	出口直径（mm）	φ150	7	314.39	11.66	1.83	4.16	2.49	228.00				228.00			66.25	1.25	
8-22	污水泵	小	出口直径（mm）	φ200	7	438.65	48.31	3.12	7.08	2.49	311.40				311.40			66.25	1.25	
8-23	泥浆泵	小	出口直径（mm）	φ50	7	121.00	5.20	1.45	4.71	2.49	40.90				40.90			66.25	1.25	
8-24	泥浆泵	小	出口直径（mm）	φ100	7	357.91	33.29	5.02	16.26	2.49	234.60				234.60			66.25	1.25	
8-25	耐腐蚀泵	小	出口直径（mm）	φ40	7	121.87	10.42	2.82	15.19	2.49	24.70				24.70			66.25	1.25	
8-26	耐腐蚀泵	小	出口直径（mm）	φ50	7	138.81	11.66	3.06	16.49	2.49	38.86				38.86			66.25	1.25	
8-27	耐腐蚀泵	小	出口直径（mm）	φ80	7	237.68	13.17	3.52	18.98	2.49	133.27				133.27			66.25	1.25	
8-28	耐腐蚀泵	小	出口直径（mm）	φ100	7	318.12	14.41	3.73	20.10	2.49	211.14				211.14			66.25	1.25	
8-29	真空泵	小	抽气速度（m^3/h）	204	8	145.21	12.44	3.09	6.64	2.99	53.80				53.80			66.25	1.25	
8-30	真空泵	小	抽气速度（m^3/h）	660	8	223.12	17.24	4.36	9.38	2.99	122.90				122.90			66.25	1.25	
8-31	潜水泵	小	出口直径（mm）	φ100	5	77.00	3.53	0.81	4.42	1.99					25.00			66.25	1.25	
8-32	潜水泵	小	出口直径（mm）	φ150	5	137.11	10.51	1.30	7.06	1.99	50.00				50.00			66.25	1.25	
8-33	砂泵	小	出口直径（mm）	φ65	8	179.96	9.24	2.41	9.07	2.99	90.00				90.00			66.25	1.25	
8-34	砂泵	小	出口直径（mm）	φ100	8	224.70	15.05	3.68	13.83	2.99	122.90				122.90			66.25	1.25	
8-35	砂泵	小	出口直径（mm）	φ125	8	359.25	31.15	7.07	26.59	2.99	225.20				225.20			66.25	1.25	
8-36	高压油泵	小	压力（Mpa）	50	7	218.25	6.98	2.15	7.16	1.99	133.72				133.72			66.25	1.25	
8-37	高压油泵	小	压力（Mpa）	80	7	308.83	15.03	2.68	8.93	1.99	213.95				213.95			66.25	1.25	
8-38	试压泵	小	压力（Mpa）	25	8	93.71	6.02	1.03	3.12	1.99	15.30				15.30			66.25	1.25	
8-39	试压泵	小	压力（Mpa）	30	8	79.46	6.21	1.24	3.77	1.99					15.66			66.25	1.25	
8-40	试压泵	小	压力（Mpa）	60	8	102.30	6.79	2.46	7.49	1.99	17.32				17.32			66.25	1.25	
8-41	试压泵	小	压力（Mpa）	80	8	106.41	7.12	3.14	9.55	1.99	18.36				18.36			66.25	1.25	
8-42	比例泵	小	2DB-5/10		8	111.15	11.25	4.66	11.10	1.49	16.40				16.40			66.25	1.25	
8-43	比例泵	小	3DS-1.8/200		8	162.15	16.63	6.06	14.42	1.49	57.30				57.30			66.25	1.25	
8-44	比例泵	小	2DB-3/37		8	151.54	22.88	8.94	21.28	1.49	30.70				30.70			66.25	1.25	
8-45	衬胶泵	小	出口直径（mm）	φ100	10	330.92	26.02	3.65	7.31	2.49	225.20				225.20			66.25	1.25	
8-46	射流井点泵	小	最大抽吸深度（m）	9.50	8.00	137.11	8.14	1.46	5.42	1.99	53.85				53.85			66.25	1.25	
九、焊接机械																				
9-1	交流电焊机	小	容量（Kv·A）	21	10	141.10	3.89	0.95	3.17	6.57	60.27				60.27			66.25	1.25	
9-2	交流电焊机	小	容量（Kv·A）	30	10	169.91	4.94	1.14	3.81	6.57	87.20				87.20			66.25	1.25	
9-3	交流电焊机	小	容量（Kv·A）	32	10	179.66	5.15	1.19	3.97	6.57	96.53				96.53			66.25	1.25	
9-4	交流电焊机	小	容量（Kv·A）	40	10	219.87	5.25	1.27	4.23	6.57	136.30				136.30			66.25	1.25	
9-5	交流电焊机	小	容量（Kv·A）	42	10	226.44	5.51	1.34	4.47	6.57	142.30				142.30			66.25	1.25	
9-6	交流电焊机	小	容量（Kv·A）	50	10	241.48	6.10	1.41	4.70	6.57	156.45				156.45			66.25	1.25	
9-7	交流电焊机	小	容量（Kv·A）	80	10	303.27	6.20	1.70	5.65	6.57	216.90				216.90			66.25	1.25	
9-8	直流电焊机	小	功率（kw）	10	10	120.29	6.74	0.91	3.62	6.57	36.20				36.20			66.25	1.25	
9-9	直流电焊机	小	功率（kw）	12	10	127.26	7.06	1.06	3.92	6.57	42.40				42.40			66.25	1.25	
9-10	直流电焊机	小	功率（kw）	14	10	136.33	7.73	1.20	4.44	6.57	50.14				50.14			66.25	1.25	
9-11	直流电焊机	小	功率（kw）	15	10	141.99	8.03	1.22	4.54	6.57	55.38				55.38			66.25	1.25	
9-12	直流电焊机	小	功率（kw）	20	10	161.05	8.72	1.50	5.55	6.57	72.46				72.46			66.25	1.25	
9-13	直流电焊机	小	功率（kw）	30	10	182.18	9.76	1.87	6.93	6.57	90.80				90.80			66.25	1.25	
9-14	直流电焊机	小	功率（kw）	32	10	186.10	10.41	1.97	7.30	6.57	93.60				93.60			66.25	1.25	
9-15	直流电焊机	小	功率（kw）	40	10	191.68	11.40	2.23	8.29	6.57	96.94				96.94			66.25	1.25	
9-16	对焊机	小	容量（Kv·A）	10	8	97.73	4.53	0.97	3.03	6.57	16.38				16.38			66.25	1.25	
9-17	对焊机	小	容量（Kv·A）	25	8	126.01	5.88	1.54	4.81	6.57	40.96				40.96			66.25	1.25	
9-18	对焊机	小	容量（Kv·A）	75	8	214.82	10.18	2.16	6.76	6.57	122.90				122.90			66.25	1.25	
9-19	硅整流焊机	小	容量（Kv·A）	15	9	128.65	6.91	2.39	8.24	6.16	38.70				38.70			66.25	1.25	
9-20	硅整流焊机	小	容量（Kv·A）	20	9	145.20	7.93	2.98	10.28	6.16	51.60				51.60			66.25	1.25	
9-21	磁放大弧焊整流器	小	ZXG2-400		8	182.32	11.60	2.84	9.45	6.16	86.02				86.02			66.25	1.25	
9-22	氩弧焊机	中	电流（A）	500	8	189.40	20.82	4.95	16.82	9.86	70.70				70.70			66.25	1.25	
9-23	二氧化碳气体保护焊机	小	电流（A）	250	8	142.63	19.60	3.65	18.77	9.86	24.50				24.50			66.25	1.25	
9-24	等离子弧焊机	小	电流（A）	300	8	325.47	29.05	3.75	20.26	9.86	196.30				196.30			66.25	1.25	
9-25	等离子切割机	小	电流（A）	400	8	316.04	25.90	2.93	17.50	9.86	193.60				193.60			66.25	1.25	
9-26	半自动切割机	小	厚度（mm）	100	10	180.46	4.31	0.73	4.60	6.57	98.00				98.00			66.25	1.25	
9-27	自动仿形切割机	小	厚度（mm）	60	10	152.77	10.90	1.34	8.36	6.57	59.35				59.35			66.25	1.25	
9-28	自动埋弧焊机	小	电流（A）	500	10	199.18	20.32	1.87	9.97	6.57	94.20				94.20			66.25	1.25	
9-29	自动埋弧焊机	小	电流（A）	1200	10	302.66	25.58	2.08	11.08	6.57	191.10				191.10			66.25	1.25	
9-30	自动埋弧焊机	小	电流（A）	1500	10	403.60	30.83	2.78	14.77	6.57	282.40				282.40			66.25	1.25	
9-31	电渣焊机	中	电流（A）	1000	10	299.59	55.51	5.80	18.46	6.57	147.00				147.00			66.25	1.25	
9-32	缝焊机	中	容量（Kv·A）	150	10	487.18	35.19	3.77	12.00	6.57	363.40				363.40			66.25	1.25	
9-33	点焊机	小	容量（Kv·A）	短臂50	10	188.32	5.15	1.82	5.31	6.57	103.22				103.22			66.25	1.25	
9-34	点焊机	中	容量（Kv·A）	长臂75	10	250.93	14.08	2.40	7.00	6.57	154.63				154.63			66.25	1.25	
9-35	点焊机	中	容量（Kv·A）	100	10	305.94	14.97	3.04	8.87	6.57	206.24				206.24			66.25	1.25	
9-36	点焊机	中	容量（Kv·A）	多头6×35	10	644.45	41.14	5.53	16.16	6.57	508.80				508.80			66.25	1.25	
9-37	汽油电焊机	小	电流（A）	160	6	373.59	17.50	3.07	3.65	4.93	278.19	28.10						66.25	1.25	
9-38	柴油电焊机	中	电流（A）	500	6	636.13	31.18	4.58	5.45	4.93	523.74		60.90					66.25	1.25	

9-39	拖拉机驱动弧焊机	中	电流（A）	二弧 2×250	8	827.88	112.58	12.20	6.47		630.38		73.30					66.25	1.25	
9-40	拖拉机驱动弧焊机	大	四弧		8	1635.82	707.25	68.97	36.55		756.80		88.00					66.25	1.25	
十、动力机械																				
10-1	柴油发电机	中	功率（kw）	30	15	563.25	34.97	7.88	25.69	13.08	415.38		48.30					66.25	1.25	
10-2	柴油发电机	中	功率（kw）	50	15	841.00	39.47	8.83	28.78	13.08	618.34		71.90					132.50	2.50	
10-3	柴油发电机	中	功率（kw）	60	15	863.14	46.97	9.64	31.43	13.08	629.52		73.20					132.50	2.50	
10-4	柴油发电机	中	功率（kw）	100	15	1167.88	55.76	11.48	37.44	13.08	917.62		106.70					132.50	2.50	
10-5	柴油发电机	中	功率（kw）	120	15	1614.97	68.19	16.41	53.51	13.08	1331.28		154.80					132.50	2.50	
10-6	柴油发电机	中	功率（kw）	160	15	1962.47	115.16	17.11	55.78	13.08	1628.84		189.40					132.50	2.50	
10-7	柴油发电机	中	功率（kw）	200	15	2438.61	140.17	18.84	61.42	13.08	2072.60		241.00					132.50	2.50	
10-8	柴油发电机	大	功率（kw）	320	15	3609.09	218.18	33.58	91.67	13.08	3120.08		362.80					132.50	2.50	
10-9	汽油发电机	小	功率（kw）	10	15	300.49	18.30	4.10	15.81	10.90	185.13	18.70						66.25	1.25	
10-10	电动空气压缩机	小	排气量（m3/min）	0.3	10	100.47	2.30	1.04	4.97	9.81	16.10				16.10			66.25	1.25	
10-11	电动空气压缩机	小	排气量（m3/min）	0.6	10	112.74	2.94	1.65	7.89	9.81	24.20				24.20			66.25	1.25	
10-12	电动空气压缩机	小	排气量（m3/min）	1	10	132.47	3.90	2.11	10.10	9.81	40.30				40.30			66.25	1.25	
10-13	电动空气压缩机	小	排气量（m3/min）	3	10	232.28	23.12	8.23	17.37	9.81	107.50				107.50			66.25	1.25	
10-14	电动空气压缩机	小	排气量（m3/min）	6	10	354.54	33.16	9.75	20.57	9.81	215.00				215.00			66.25	1.25	
10-15	电动空气压缩机	中	排气量（m3/min）	10	10	557.39	46.47	10.18	21.48	9.81	403.20				403.20			66.25	1.25	
10-16	电动空气压缩机	中	排气量（m3/min）	20	10	914.21	78.81	16.68	35.20	18.37	698.90				698.90			66.25	1.25	
10-17	电动空气压缩机	大	排气量（m3/min）	40	10	1640.53	236.64	26.14	43.13	18.37	1250.00				1250.00			66.25	1.25	
10-18	内燃空气压缩机	中	排气量（m3/min）	3	10	379.64	28.40	12.74	42.28	9.81	220.16		25.60					66.25	1.25	
10-19	内燃空气压缩机	中	排气量（m3/min）	6	10	504.41	48.77	15.80	52.46	9.81	311.32		36.20					66.25	1.25	
10-20	内燃空气压缩机	中	排气量（m3/min）	9	10	654.38	64.31	16.46	54.65	9.81	442.90		51.50					66.25	1.25	
10-21	内燃空气压缩机	中	排气量（m3/min）	12	10	795.41	68.77	21.00	69.72	9.81	559.86		65.10					66.25	1.25	
10-22	内燃空气压缩机	中	排气量（m3/min）	17	10	1632.98	75.84	33.29	110.53	18.37	1328.70		154.50					66.25	1.25	
10-23	内燃空气压缩机	大	排气量（m3/min）	40	10	4896.61	212.23	57.90	137.80	18.37	4404.06		512.10					66.25	1.25	
10-24	无油空气压缩机	中	排气量（m3/min）	9	10	575.29	107.81	26.75	36.91	18.37	319.20				319.20			66.25	1.25	
10-25	无油空气压缩机	中	排气量（m3/min）	20	10	1107.28	221.56	47.06	64.94	18.37	689.10				689.10			66.25	1.25	
10-26	工业锅炉	中	蒸发量（t/h）	1	7	1009.91	127.00	16.93	8.80	54.33	736.60			1.150		7.30	16.00	66.25	1.25	
10-27	工业锅炉	中	蒸发量（t/h）	2	7	1684.89	153.35	24.45	12.71	54.33	1373.80			2.173		14.00	21.00	66.25	1.25	
10-28	工业锅炉	中	蒸发量（t/h）	4	7	2178.91	243.45	38.08	19.80	54.33	1757.00			2.785		19.00	24.00	66.25	1.25	
十一、地下工程机械																				
11-1	干式出土盾构掘进机	特	直径（mm）	φ3500	8	1355.85	973.54	140.04	242.27											
11-2	干式出土盾构掘进机	特	直径（mm）	φ5000	8	2028.72	1456.67	209.54	362.51											
11-3	干式出土盾构掘进机	特	直径（mm）	φ7000	8	2697.40	1936.80	278.61	481.99											
11-4	干式出土盾构掘进机	特	直径（mm）	φ10000	8	2979.30	1998.10	287.74	693.46											
11-5	干式出土盾构掘进机	特	直径（mm）	φ12000	8	4250.64	2850.73	410.53	989.38											
11-6	水力出土盾构掘进机	特	直径（mm）	φ3500	8	1493.59	1106.73	143.28	243.58											
11-7	水力出土盾构掘进机	特	直径（mm）	φ5000	8	2113.91	1566.38	202.79	344.74											
11-8	水力出土盾构掘进机	特	直径（mm）	φ7000	8	2793.57	2070.00	267.99	455.58											
11-9	水力出土盾构掘进机	特	直径（mm）	φ10000	8	3376.42	2204.80	285.76	885.86											
11-10	水力出土盾构掘进机	特	直径（mm）	φ12000	8	4800.85	3134.95	406.32	1259.58											
11-11	气压平衡式盾构掘进机	特	直径（mm）	φ3500	10	2665.94	1985.06	269.12	411.76											
11-12	气压平衡式盾构掘进机	特	直径（mm）	φ5000	10	3348.12	2493.01	337.99	517.12											
11-13	气压平衡式盾构掘进机	特	直径（mm）	φ7000	10	4376.51	3258.75	441.80	675.96											
11-14	刀盘式干出土土压平衡盾构掘进机	特	管径（mm）	φ3500	10	2323.71	1695.99	229.93	397.79											
11-15	刀盘式干出土土压平衡盾构掘进机	特	管径（mm）	φ5000	10	3476.74	2537.54	344.03	595.17											
11-16	刀盘式干出土土压平衡盾构掘进机	特	管径（mm）	φ7000	10	4622.84	3374.04	457.44	791.36											
11-17	刀盘式水力出土泥水平衡盾构掘进机	特	管径（mm）	φ3500	10	2409.74	1812.59	221.17	375.98											
11-18	刀盘式水力出土泥水平衡盾构掘进机	特	管径（mm）	φ5000	10	3605.55	2712.07	330.92	562.56											
11-19	刀盘式水力出土泥水平衡盾构掘进机	特	管径（mm）	φ7000	10	4806.40	3615.34	441.13	749.93											
11-20	盾构同步压浆泵	大	φ2.1m×7m		8	631.77	394.13	42.88	50.17	26.24	52.10				52.10			66.25	1.25	
11-21	医疗闸设备	中	φ2.1m×7m		11	290.22	125.95	12.85	28.39	18.37	38.41				38.41			66.25	1.25	
11-22	垂直顶升设备	中			10	1386.70	329.12	37.58	73.66	24.49	590.60				590.60			331.25	6.25	
11-23	履带式绳索抓斗成槽机	特	550A-50 MHL-630		12	2657.24	1059.77	158.08	222.89		951.50		110.64					265.00	5.00	
11-24	履带式液压抓斗成槽机	特	KH180 MHL-800		12	3640.64	1608.14	239.88	338.24		1189.38		138.30					265.00	5.00	
11-25	履带式液压抓斗成槽机	特	KH180 MHL-1200		12	5478.61	2588.25	386.09	544.38		1694.89		197.08					265.00	5.00	
11-26	导杆式液压抓斗成槽机	特	E50 KRC2/45 K2502		12	5377.58	2992.08	296.83	418.52		1405.15		163.39					265.00	5.00	
11-27	井架式液压抓斗成槽机	大			12	894.62	187.50	27.97	137.05		277.10				277.10			265.00	5.00	
11-28	反循环钻机	特	60P45A		12	2678.01	1617.19	174.95	306.17		447.20		52.00					132.50	2.50	
11-29	多头钻成槽机	特	BW		10	4851.42	2820.92	323.34	1008.81		499.60				499.60			198.75	3.75	
11-30	超声波测壁仪	小			6	188.15	52.10	11.68	19.28	1.99	36.85				36.85			66.25	1.25	
11-31	泥浆制作循环设备	大			10	1982.29	971.62	44.67	67.46	63.39	503.90				503.90			331.25	6.25	
11-32	锁口管顶升机	中			10	311.65	75.53	6.98	30.65	1.99	64.00				64.00			132.50	2.50	
11-33	沉井钻吸机组	大	KH180-2 配GZQ1250A		13	4083.21	1286.76	297.00	403.92		1764.28		67.80		1181.20			331.25	6.25	
11-34	潜水电钻	中	75型		8	188.57	44.36	10.53	24.42	9.86	99.40				99.40					
11-35	潜水电钻	中	80型		8	213.89	58.52	13.89	32.22	9.86	99.40				99.40					
11-36	液压钻机	中	G-2A		7	482.60	56.05	11.71	12.53	4.93	264.88		30.80					132.50	2.50	
11-37	双液压注浆泵	中	PH2×5		9	302.97	151.65	23.26	22.33	8.22	31.26				31.26			66.25	1.25	
11-38	液压注浆泵	中	HYB50/50-1型		9	175.36	61.74	12.00	11.52	8.22	15.63				15.63			66.25	1.25	
11-39	刀盘式土压平衡顶管掘进机	特	管径（mm）	φ1650	10	1056.53	509.17	59.18	108.89	16.33	362.96				362.96					
11-40	刀盘式土压平衡顶管掘进机	特	管径（mm）	φ1800	10	1292.89	677.90	72.77	133.89	16.33	392.00				392.00					
11-41	刀盘式土压平衡顶管掘进机	特	管径（mm）	φ2200	10	1490.03	805.10	86.35	158.89	16.33	423.36				423.36					
11-42	刀盘式土压平衡顶管掘进机	特	管径（mm）	φ2460	10	2542.06	1536.47	164.48	302.64	16.33	522.14				522.14					
11-43	刀盘式土压平衡顶管掘进机	特	管径（mm）	φ2800	10	2782.08	1688.57	180.73	332.54	16.33	563.91				563.91					
11-44	刀盘式土压平衡顶管掘进机	特	管径（mm）	φ3000	10	3000.35	1821.43	194.92	358.65	16.33	609.02				609.02					
11-45	人工挖土法顶管设备	小	管径（mm）	φ1200	10	177.96	15.04	2.19	9.54	1.33	149.86				149.86					
11-46	人工挖土法顶管设备	小	管径（mm）	φ1650	10	231.56	17.60	2.31	10.06	1.33	200.26				200.26					
11-47	人工挖土法顶管设备	小	管径（mm）	φ2000	10	286.85	19.52	2.86	12.48	1.33	250.66				250.66					
11-48	人工挖土法顶管设备	中	管径（mm）	φ2460	10	289.69	21.09	3.10	13.51	1.33	250.66				250.66					

11-49	挤压法顶管设备	中	管径（mm）	φ1000	10	198.20	27.86	3.99	15.16	1.33	149.86				149.86					
11-50	挤压法顶管设备	中	管径（mm）	φ1200	10	207.63	33.45	4.79	18.20	1.33	149.86				149.86					
11-51	挤压法顶管设备	中	管径（mm）	φ1400	10	262.58	36.14	5.18	19.67	1.33	200.26				200.26					
11-52	挤压法顶管设备	中	管径（mm）	φ1500	10	271.44	45.49	6.51	17.85	1.33	200.26				200.26					
11-53	挤压法顶管设备	中	管径（mm）	φ1650	10	287.80	56.14	8.04	22.03	1.33	200.26				200.26					
11-54	挤压法顶管设备	中	管径（mm）	φ1800	10	354.86	68.46	9.80	24.61	1.33	250.66				250.66					
11-55	挤压法顶管设备	中	管径（mm）	φ2000	10	381.91	86.46	12.38	31.08	1.33	250.66				250.66					
11-56	挤压法顶管设备	中	管径（mm）	φ2200	10	417.90	115.70	16.57	33.64	1.33	250.66				250.66					
11-57	挤压法顶管设备	中	管径（mm）	φ2400	10	469.27	151.53	21.70	44.05	1.33	250.66				250.66					
11-58	液压柜（动力系统）	中			10	269.88	10.03	1.44	7.75		250.66				250.66					
11-59	遥控顶管掘进机	特	管径（mm）	φ800	10	2475.66	1469.85	256.48	471.93	16.33	261.07				261.07					
11-60	遥控顶管掘进机	特	管径（mm）	φ1200	10	2561.62	1520.03	269.08	495.11	16.33	261.07				261.07					
11-61	遥控顶管掘进机	特	管径（mm）	φ1350	10	2695.52	1591.10	281.68	518.29	16.33	288.12				288.12					
11-62	遥控顶管掘进机	特	管径（mm）	φ1650	10	2892.86	1704.37	301.76	555.23	16.33	315.17				315.17					
11-63	遥控顶管掘进机	特	管径（mm）	φ1800	10	3198.52	1907.68	337.80	621.54	16.33	315.17				315.17					
11-64	三臂凿岩台车	大	H178		12	7177.92	3640.48	953.49	2583.95											
11-65	三向倾卸轮胎式装载机	大	966D		12	1780.26	1078.67	189.11	512.48											
11-66	装药台车	大	DT-100		12	1819.45	1146.07	181.50	491.88											
十二、其他机械																				
12-1	轴流风机	小	功率（Kw）	7.5	10	49.77	4.34	0.61	1.53	2.99	40.30				40.30					
12-2	轴流风机	小	功率（Kw）	30	10	179.82	11.24	1.22	3.07	2.99	161.30				161.30					
12-3	轴流风机	小	功率（Kw）	100	10	571.84	23.49	2.70	5.06	2.99	537.60				537.60					
12-4	离心通风机	小	能力（m^3/min）	335-1300	7	110.06	11.04	2.45	4.58	1.99	90.00				90.00					
12-5	离心通风机	小	能力（m^3/min）	464-1717	7	183.63	13.28	3.26	6.10	1.99	159.00				159.00					
12-6	离心通风机	小	能力（m^3/min）	585-2463	7	326.11	21.34	4.97	6.21	1.99	291.60				291.60					
12-7	离心通风机	小	能力（m^3/min）	747-3132	7	608.74	29.28	6.70	8.37	1.99	562.40				562.40					
12-8	吹风机	小	能力（m^3/min）	4	13	86.74	9.73	0.92	2.31	3.98	69.80				69.80					
12-9	鼓风机	小	能力（m^3/min）	18	13	250.97	26.16	1.70	4.13	3.98	215.00				215.00					
12-10	风动锻钎机	小			12	164.37	16.24	4.73	3.31	7.59								132.50	2.50	
12-11	液压锻钎机	中	功率（Kw）	11.25	12	262.30	26.71	8.18	5.72	7.59	81.60				81.60			132.50	2.50	
12-12	电动修钎机	中			11	288.92	37.24	6.75	4.59	7.04	100.80				100.80			132.50	2.50	
12-13	液压千斤顶	小	起重量（t）	100	12	14.68	6.26	2.30	3.82	2.30										
12-14	液压千斤顶	小	起重量（t）	200	12	19.68	8.68	3.27	5.43	2.30										
12-15	液压千斤顶	小	起重量（t）	300	12	26.47	12.92	4.23	7.02	2.30										
12-16	磨砖机	小	功率（Kw）	4	6	99.41	14.00	2.73	3.44	2.99	10.00				10.00			66.25	1.25	
12-17	切砖机	小	功率（Kw）	5.5	6	99.38	7.79	4.75	4.60	2.99	13.00				13.00			66.25	1.25	
12-18	钻砖机	小	直径（mm）	φ13	6	83.82	6.24	1.02	1.72	2.99	5.60				5.60			66.25	1.25	
12-19	平面磨石机	小	功率（Kw）	3	8	27.88	4.38	0.74	6.27	2.49	14.00				14.00					
12-20	立面磨石机	小	功率（Kw）	1.1	8	36.29	10.75	0.95	8.10	2.49	14.00				14.00					
12-21	除锈喷砂机	小	能力（m^3/min）	3	12	22.84	13.05	3.08	4.41	2.30										
12-22	箱式加热炉	小	RJX-45-9		8	169.89	20.80	5.19	5.50	2.30	136.10				136.10					
12-23	箱式加热炉	小	RJX-75-9		8	278.90	33.28	8.02	8.50	2.30	226.80				226.80					
12-24	箱式加热炉	小	RJX-50-13		8	208.78	36.88	9.03	9.57	2.30	151.00				151.00					
12-25	立爪扒渣机	大	瑞典 9HR		18	987.95	387.51	76.67	175.57		215.70				215.70			132.50	2.50	
12-26	梭式矿车	中	S8m^3		9	327.46	90.88	17.50	16.10	5.48	65.00				65.00			132.50	2.50	
12-27	电瓶车	小	载重量（t）	2.5	9	144.82	41.48	10.11	16.08	10.90								66.25	1.25	
12-28	电瓶车	中	载重量（t）	5	9	255.51	63.71	12.19	26.70	20.41								132.50	2.50	
12-29	电瓶车	中	载重量（t）	7	9	280.20	80.64	14.62	32.03	20.41								132.50	2.50	
12-30	电瓶车	中	载重量（t）	8	9	285.38	81.08	16.11	35.28	20.41								132.50	2.50	
12-31	电瓶车	中	载重量（t）	10	9	314.25	97.54	20.00	43.80	20.41								132.50	2.50	
12-32	电瓶车	中	载重量（t）	12	9	359.76	125.34	25.55	55.96	20.41								132.50	2.50	
12-33	硅整流充电机	小	90A/190V		10	170.43	13.88	3.20	10.11	1.99	75.00				75.00			66.25	1.25	
12-34	泥浆拌合机	小	100-150L		10	85.16	3.03	0.81	3.41	1.66	10.00				10.00			66.25	1.25	
12-35	潜水设备	小			7	873.47	36.09	2.67	37.41	2.30								795.00	15.00	
12-36	潜水减压仓	中			8	209.59	145.82	13.78	44.51	5.48										
12-37	风动灌浆机	小			6	82.17	6.02	1.32	6.92	1.66								66.25	1.25	
12-38	电动灌浆机	小			6	101.52	8.25	1.47	7.69	1.66	16.20				16.20			66.25	1.25	
12-39	组合烘箱	小			7	188.83	36.23	7.15	7.36	1.99	136.10				136.10					
12-40	液压升降机	小	提升高度（m）	2	10	34.83	19.83	2.51	10.00	2.49										
12-41	平台升降车	中	提升高度（m）	2.5	10	926.30	257.09	50.94	71.31	1.66	412.80		48.00					132.50	2.50	
12-42	平台升降车	中	提升高度（m）	3	10	2263.51	1445.26	70.04	98.05	1.66	516.00		60.00					132.50	2.50	
12-43	反吸式除尘机	小	D2-FX1		7	108.34	50.86	3.99	4.50	1.99	47.00				47.00					
12-44	电焊条烘干箱	小	容积（cm^3）	45×35×45	7	20.00	8.19	1.87	3.24		6.70				6.70					
12-45	电焊条烘干箱	小	容积（cm^3）	55×45×55	7	26.71	10.72	2.19	3.80		10.00				10.00					
12-46	电焊条烘干箱	小	容积（cm^3）	60×50×75	7	34.91	14.22	2.49	4.30		13.90				13.90					
12-47	电焊条烘干箱	小	容积（cm^3）	80×80×100	7	68.84	19.32	3.60	6.22		39.70				39.70					
12-48	超声波探伤仪	小	CTS-8		9	120.53	21.17	7.73	14.39	2.99	8.00				8.00			66.25	1.25	
12-49	超声波探伤仪	小	CTS-22		9	157.75	27.19	9.73	18.09	2.99	33.50				33.50			66.25	1.25	
12-50	超声波探伤仪	小	CTS-26		12	177.68	34.11	10.35	23.39	3.98	39.60				39.60			66.25	1.25	
12-51	X光探伤仪	小	1605		12	143.48	35.33	10.68	24.14	3.98	3.10				3.10			66.25	1.25	
12-52	X光探伤仪	小	2005		12	151.72	40.93	11.28	25.48	3.98	3.80				3.80			66.25	1.25	
12-53	X光探伤仪	小	2505		12	178.62	58.07	12.46	28.16	3.98	9.70				9.70			66.25	1.25	
12-54	X光探伤仪	小	3005		12	199.61	70.44	13.91	31.43	3.98	13.60				13.60			66.25	1.25	
12-55	周向X光探伤仪	小	携带式2005		12	178.21	53.34	19.26	31.58	3.98	3.80				3.80			66.25	1.25	
12-56	磁粉探伤机	小	周向磁化电流（A）	6000	8	192.25	57.42	23.61	35.18	2.49	7.30				7.30			66.25	1.25	
12-57	磁粉探伤机	小	周向磁化电流（A）	9000	8	241.56	60.79	26.52	39.51	2.49	46.00				46.00			66.25	1.25	
12-58	磁粉探伤机	中	周向磁化电流（A）	12500	8	976.97	259.09	102.02	152.02	2.49	395.10				395.10			66.25	1.25	
12-59	冷缠机	大	功率（Kw）	157	10	1026.74	269.45	26.26	72.21		526.32		61.20					132.50	2.50	
12-60	打洞立杆机	大	功率（Kw）	92	10	734.51	179.46	24.02	64.85		333.68		38.80					132.50	2.50	
12-61	通井机	大	功率（Kw）	66	11	851.61	154.64	27.28	73.65		463.54		53.90					132.50	2.50	
12-62	抓管机	大	功率（Kw）	80	14	881.30	123.29	34.01	91.84		499.66		58.10					132.50	2.50	
12-63	抓管机	大	功率（Kw）	120	14	1228.42	300.38	55.33	149.39		590.82		68.70					132.50	2.50	
12-64	抓管机	大	功率（Kw）	160	14	1508.53	332.51	74.24	200.44		768.84		89.40					132.50	2.50	
12-65	吊管机	大	功率（Kw）	75	14	828.90	133.98	27.19	73.41		461.82		53.70					132.50	2.50	
12-66	吊管机	大	功率（Kw）	165	14	1567.44	428.16	75.70	204.38		726.70		84.50					132.50	2.50	
12-67	吊管机	大	功率（Kw）	240	14	1851.13	571.62	88.03	237.68		821.30		95.50					132.50	2.50	
12-68	高压压风机车	大	功率（Kw）	300	13	3914.03	432.07	71.52	200.27		2954.10		343.50					66.25	1.25	189.82

12-69	水泥车	大	最高工作压力（Mpa）	30	8	1386.75	356.40	24.64	66.53		806.68		93.80					132.50	2.50	
12-70	水泥车	大	最高工作压力（Mpa）	40	8	2377.94	760.82	50.34	135.91		1108.54		128.90					132.50	2.50	189.83
12-71	横孔钻机	大	功率（Kw）	40	12	1075.96	624.16	48.45	106.59		164.26		19.10					132.50	2.50	
12-72	履带式钻孔机	大	孔径（mm）	φ400~700	14	823.38	285.28	18.48	63.76		323.36		37.60					132.50	2.50	
12-73	轻便钻机	中	XJ-100		11	206.78	13.56	2.99	12.41	1.49	110.08		12.80					66.25	1.25	
12-74	液压钻机	中	XU-100		11	273.74	47.34	4.56	21.66	1.49	132.44		15.40					66.25	1.25	
12-75	工程修理车	中	JX-12A		8	763.26	109.37	19.17	44.09		259.38	26.20						331.25	6.25	
12-76	工程修理车	大	EQ-141		8	991.53	196.19	29.03	66.78		368.28	37.20						331.25	6.25	
12-77	滤油机	小	LX100型		8	110.32	4.99	0.48	1.11	1.49	36.00				36.00			66.25	1.25	
12-78	对口器	中	φ426mm		8	149.32	40.40	15.84	25.34	1.49								66.25	1.25	
12-79	对口器	中	φ529mm		8	159.31	45.49	17.72	28.36	1.49								66.25	1.25	
12-80	对口器	中	φ720mm		8	221.56	86.84	25.76	41.22	1.49								66.25	1.25	
12-81	喷砂机	小	HP2V-5		10	34.41	17.40	1.37	3.15	1.99	10.50				10.50					
12-82	布袋除尘切砖机	小	φ400mm		10	29.69	10.23	0.56	1.31	1.99	15.60				15.60					
12-83	热熔焊接机	小	SH-63		7	22.46	11.27	2.52	2.67	1.99	4.01				4.01					
12-84	热熔焊接机	小	SHD-160C		7	86.61	53.14	11.88	12.59	1.99	7.01				7.01					
12-85	热熔焊接机	小	SHD-630		7	419.35	263.41	58.89	62.42	1.99	32.64				32.64					
12-86	多角焊接机	小	DSH-250		7	260.08	169.48	37.89	40.16	1.99	10.56				10.56					
12-87	电熔焊接机	小	DRH-160A		7	45.11	24.96	5.58	5.91	1.99	6.67				6.67					
