# Supplementary material for: Carbon emission quantification analysis of excavation engineering under road transport conditions
Source: PLoS One. 2024 Dec 31;19(12):e0315765. doi: 10.1371/journal.pone.0315765 (PMC11687867; doi:10.1371/journal.pone.0315765)
Supplement: Attachment — Submitted filename: Response to Reviewers.docx [file pone.0315765.s005.docx]

Dear editor, Thanks for your letter and for reviewers' comments concerning our manuscript entitled"**Carbon emission quantification analysis of excavation engineering under road transport conditions**"(Manuscript ID: **PONE-D-24-19328**). Those comments are allvaluable and helpful for revising and improving our paper. We have studied all comments carefully and have made conscientious correction. Revised portion are marked in the paper. The responds to the reviewers' comments are as flowing:

**Reviewer 1**

The article deals with an important aspect of the pit construction and its lifecycle. The following issues should be addressed before the acceptance.

**Comment 1:**The introduction is talking about building construction and its lifecycle which is not the scope of this research. It should be modified accordingly.

**Response:**We really appreciate your efforts and comments on our manuscript.We have revised the introduction section based on your suggestion, making it within the scope of this study.Therevised part has been noted in the paper.As follows:(The blue font represents the modified part.)

With the acceleration of industrialization, the development of society heavily depends on the combustion of fossil fuels, which leads to the emission of large amounts of greenhouse gases, resulting in the intensification of the global warming problem[1]. In the Sixth IPCC Assessment Report, it is noted that compared to the average temperature between 1850 and 1900, the surface temperature has increased by approximately 1.1 ℃. In addition, according to the Climate Sensitivity Index (CSI), it is predicted that the global average temperature will increase by approximately 3 ℃ without any change in the existing global development pattern[2]. In response, since 1990, countries worldwide have implemented energy conservation and emission reduction actions on a large scale[3], aiming to control global warming below the 2 ℃ warning line[4].

Energy consumption and carbon emissions originating from the construction industry account for 40% and 36%, respectively, of the total emissions[5]. China, as the world's largest energy consumer and carbon emitter, produces approximately 10 billion tons of carbon emissions annually[6], accounting for approximately 30% of the global emissions[7], of which the carbon emissions stemming from the construction industry account for approximately 35%–50% of China's total carbon emissions[8]. Thus, energy-saving and emission reduction efforts in the construction industry could help China quickly realize the dual-carbon goal.Moon et al.[9]found that the application of the process analysis method at the design stage only produced an error of 8% in calculating building carbon emissions. Keoleian et al.[10] evaluated the life cycle energy consumption and greenhouse gas emissions of residential buildings and found that the energy consumption of the operation and use phase exceeded 91% overall, which is the main source phase of carbon emissions. Yujie Cang[11]proposed a carbon emission calculation method based on the structure of the building as the basic unit to calculate the amount of building materials used at the materialization stage and the associated carbon emissions.

With global urbanization on the rise, limited urban surface space and increasing traffic congestion have become critical obstacles to further urban development. Metro systems, with their high-speed operations and large passenger capacity, are emerging as the optimal solution for many countries to address these challenges, becoming the preferred mode of modern urban transportation[12,13].As of early 2022, a total of 36854.2 kilometers of urban rail transit systems have been built across 541 cities in 76 countries worldwide[14].As of early 2024, mainland China has established urban rail transit systems in 59 cities, with 338 operational lines spanning a total length of 11,224.54 kilometers. Among these, metro lines constitute a significant 76.11%, covering 8,543.11 kilometers in operational length[15].Given the vast scale of underground space development, accurately assessing carbon emissions in foundation pit projects is essential due to the complex underground environment, constraints on above-ground conditions in urban areas, and the considerable construction challenges. Subway construction, in particular, often requires extensive excavation, which can lead to significant environmental impacts.

Foundation pit construction is the first step in building and rail transit projects; however, there has been limited research on the characteristics of carbon emissions specific to foundation pit engineering. Furthermore, existing carbon emission assessment models have not effectively accounted for the relevant influencing factors.This study, based on lifecycle assessment and process analysis methods, reasonably considers the impact of vehicle unladen conditions on the carbon emission calculation model for the transportation phase. It aims to accurately evaluate the carbon emissions associated with the construction of foundation pits, conduct a comparative analysis of the carbon emissions across various stages of the lifecycle of foundation pit projects, summarize the carbon emission characteristics of such projects, and provide a theoretical basis for energy-saving and emission-reduction measures in foundation pit construction.

**Comment 2:**Section 2 and 3 can be merged together.

**Response:**We have taken your advice and merged the second and third parts together.Thank you for your suggestion.

**Comment 3:**There is no reference for Equations (1), (2), (6) and Tables 1 to 4.

**Response:**We have taken your advice and added references to Equations (1), (2), (6) and Tables 1 to 4.Thank you for your suggestion.

**Comment 4:**It is not clear what updates the authors made with the emission model? Is it one of the equations mentioned in the previous comment? If yes, then the basis of developing these equations should be mentioned.

**Response: (1)** We are apologize for cinfusing you that not clear what updates the authors made with the emission model. The updated carbon emission calculation model is Equation 5. We have rewritten the abstract and added a "Research Methods" section to the text, detailing which parts of the model we have updated. As follows:(The blue font represents the updates we have made to our carbon emission model.)

***a.***Abstract: Current research on building carbon emissions primarily focuses on various carbon emission assessment models and the use of life cycle analysis to evaluate overall building carbon emissions, with limited attention given to excavation engineering.Based on the life cycle method and process analysis, this study analyzes carbon emissions in excavation engineering by optimizing the evaluation model for fuel consumption standards of freight vehicles during the transportation phase in China.To account for the difference between actual and rated fuel consumption of transport vehicles, factors such as road conditions, traffic congestion, and temperature are introduced to adjust the carbon emission calculation model for the transportation phase. This approach reasonably incorporates the impact of fuel consumption during vehicle idling on carbon emission calculations.Using the 02B excavation of the Beijing Sub-Center Station transportation hub as a case study to validate the proposed method, the analysis reveals that the primary source of carbon emissions in excavation engineering is earthwork transportation, accounting for 40.50% of total emissions. Among these, earthwork transportation contributes 95.28% of emissions within the transportation phase.Due to adjustments in the carbon emission calculation model for the transportation phase, carbon emissions increased by 1,226.79 tons, accounting for 9.2% of the total. The revised model provides a theoretical basis for accurately assessing carbon emissions in excavation engineering.

***b.*** Research method

The research process of this study can be divided into four stages. First, through a literature review and data survey, it highlights the proportion of carbon emissions contributed by the construction industry globally, and emphasizes the growing necessity of analyzing the carbon emissions of foundation pit engineering as urbanization continues. With the saturation of above-ground construction and the rising trend of rail transit development, this shift further underscores the importance of such an analysis.Next, a life cycle assessment (LCA) is used to analyze the carbon emissions of foundation pit engineering. The calculation model adopts the process analysis method, which is further optimized by incorporating the fuel consumption standards for freight trucks in China. Specifically, the transportation stage model within the process analysis is adjusted to account for the discrepancy between actual and rated fuel consumption. Factors such as road conditions, traffic congestion, and temperature are introduced to refine the model. Additionally, the impact of empty-load fuel consumption on carbon emissions is reasonably considered. The optimized model can accurately assess the carbon emissions during the transportation phase.Finally, the impact of the model adjustments on carbon emissions is demonstrated through the case of the foundation pit for the 02B station at the Beijing Sub-Center Transportation Hub. The carbon emission characteristics of foundation pit engineering are then analyzed. The study concludes by identifying the existing issues and limitations, and outlining the potential directions for future research and development.

**(2)** The basis for developing these equations has already been described in the text, but our expression was not clear enough to confuse you. We introduced the basis for developing these equations in (1) and provided a detailed explanation of the development principles in section 3.3.2.As follows:

*3.3.2 Model for calculating the carbon emissions at the transportation stage*

The carbon emissions during the transportation phase mainly result from vehicle fuel consumption, and more than 95% of vehicles rely on fossil fuels[21]. Building materials are mainly transported along road, railroad and water transport networks, and we study the carbon emissions of foundation pit projects under road transportation conditions in this paper. The traditional fuel consumption calculation method for truck operation is based on the rated fuel consumption of the vehicle, but under the influence of external factors[22], the actual consumption of the vehicle is often higher than the rated consumption, which leads to notable underestimation of the actual carbon emissions in the traditional calculation method. Therefore, according to the guidelines in Cargo Vehicle Operation Fuel Consumption (GB/T 4352-2022)[23]for optimizing the calculation of carbon emissions at the transportation stage, the actual fuel consumption of cargo vehicle operation can be calculated with Equations 3 and 4:

 (3)

 (4)

where *Q*_r_ - Actual fuel consumption (L) for a certain mode of cargo vehicle operation;

*Q*_b_ - Fuel consumption of cargo vehicles per unit load mass change (L/100 km);

*Q*_m_ - Full load fuel consumption of cargo vehicles (L/100 km);

*Q*_k_ - Vehicle no-load base fuel consumption (L/100 km);

*M*_m_ - Maximum gross mass of the cargo vehicle (t);

*M*_k_ - Full mass of the cargo vehicle (t);

*S*_i_ - Mileage (km) of the cargo vehicle for a certain mode of operation;

∆G - Mass of the loaded vehicle (t) for a certain mode of transportation;

Kr - Road correction factor (refer to Table 2);

*K*_t_ - Temperature correction factor (refer to Table 3);

*K*_v_ - Congestion correction factor (refer to Table 4); and

*K*_x_ - Correction factor for other influencing factors, mainly including the car walking-in period, driving practice period, local rainy period, loading of hazardous materials, overturning, and icy and snowy roads, which are generally specified by the car-using organization.

Table 2 Correction factors for the various road categories[23]

| Type of road | Category 1 roads | Category 2 roads | Category 3 roads | Category 4 roads | Category 4 roads | Category 6 roads |
| --- | --- | --- | --- | --- | --- | --- |
| *Kr* | 1.00 | 1.10 | 1.25 | 1.35 | 1.45 | 1.70 |

Table 3 Temperature correction factors[23]

| Monthly average temperature (°C) | t≤-25 | -25<t≤-15 | -15<t≤-5 | -5<t≤5 | 5<t≤28 | t>28 |
| --- | --- | --- | --- | --- | --- | --- |
| *Kt* | 1.13 | 1.09 | 1.06 | 1.03 | 1.00 | 1.02 |

Table 4 Congestion correction factors[23]

| Average travel speed (km/h) | V≤20 | 20<V≤30 | 30<V≤40 | 40<V≤50 | V>50 |
| --- | --- | --- | --- | --- | --- |
| *K*_v_ | 1.30 | 1.15 | 1.00 | 0.90 | 0.80 |

According to Equations 3 and 4, the actual fuel consumption of a vehicle is mainly affected by the type of road, weather and temperature conditions, degree of traffic congestion (defined by the average speed), and load. Regarding the influence of the load, first, we must determine whether the maximum total mass of the vehicle reaches the critical condition of 3500 kg. Second, we must assess the data provided by automobile manufacturers, Road Transportation Vehicle Compliance Vehicle Model List or Fuel Consumption Labeling for Light-Duty Vehicles of the Ministry of Transportation and Communications. Finally, we must calculate the change in the fuel consumption caused by the load. In this study, we show that the no-load fuel consumption of the vehicle is 0.67 times the full-load fuel consumption[24]. Based on this finding, Equations 3 and 4 are corrected, and the calculation models are expressed in Equation 5:

 (5)

where *Q*_t_ denotes the total carbon emission at the transportation stage (t); *m*_i_ is the transportation volume of the ith material (t); *d*_i_ is the transportation distance of the ith material (km); *a*_i_ is the carbon emission coefficient of the ith energy source; and *T*_c_ is the energy consumption per unit of the material transportation distance (MJ-(t-km)^-1^).

**Comment 5:**The analysis was applied to single project. Hence, the limitations of the work should be mentioned specifically with the conclusions. Accordingly, the future direction and implementation of the present research should be mentioned.

**Response:**Thank you for your kind suggestion.The paper has been revised carefully and thoroughly according to the comments from you. In the revised manuscript,the limitations of the work have been added and discussed. And the future direction and implementation of the present researchhave been supplemented. We have added a discussion on the above content at the conclusion of Chapter 5. As follows:

This study uses the Beijing Urban Sub-center Station Transportation Hub project as a case study for carbon emission research. The analysis results can provide a theoretical basis for energy conservation and emission reduction in foundation pit construction. However, considering the heavy workload of material transport vehicles and the internal factors of the vehicles that affect energy consumption, the analysis may still underestimate the actual carbon emissions.In addition, this study analyzes the construction process of a single project. Due to the complexity of the foundation pit construction process, there is uncertainty in the emission factors of materials used, actual transportation distances, and types of energy consumed, which limits the conclusions drawn. Therefore, future research should consider using uncertainty methods for carbon emission analysis in foundation pit projects to support energy conservation and emission reduction in such projects.

**Reviewer 2**

Please apply the following suggestions to improve the manuscript:

**Comment 1:**I don't think it is important to include the division of the life cycle of pit construction in the abstract. The abstract should contain general information about the work, the methods used, the results, and the novelty of the study.

**Response:**Thank you for your insightful question. Based on your suggestions,We have rewritten the abstract.As follows:

Abstract: Current research on building carbon emissions primarily focuses on various carbon emission assessment models and the use of life cycle analysis to evaluate overall building carbon emissions, with limited attention given to excavation engineering.Based on the life cycle method and process analysis, this study analyzes carbon emissions in excavation engineering by optimizing the evaluation model for fuel consumption standards of freight vehicles during the transportation phase in China.To account for the difference between actual and rated fuel consumption of transport vehicles, factors such as road conditions, traffic congestion, and temperature are introduced to adjust the carbon emission calculation model for the transportation phase. This approach reasonably incorporates the impact of fuel consumption during vehicle idling on carbon emission calculations.Using the 02B excavation of the Beijing Sub-Center Station transportation hub as a case study to validate the proposed method, the analysis reveals that the primary source of carbon emissions in excavation engineering is earthwork transportation, accounting for 40.50% of total emissions. Among these, earthwork transportation contributes 95.28% of emissions within the transportation phase.Due to adjustments in the carbon emission calculation model for the transportation phase, carbon emissions increased by 1,226.79 tons, accounting for 9.2% of the total. The revised model provides a theoretical basis for accurately assessing carbon emissions in excavation engineering.

**Comment 2:**The calculation model should be included in the keywords.

**Response:**Thank you for your remainder.We have added calculation models to the keywords.

**Comment 3:**In line 73, the phrase "In a large number of studies at home and abroad" should be revised. I suggest proofreading the entire text to improve the English.

**Response:**We really appreciate your efforts and comments on our manuscript.A careful proof-reading has been conducted for the revised manuscript to improve the language and grammar. We have tried our best to polish the language in the revised manuscript. AJE also helped us polish the English language of this article.

**Comment 4:**It is recommended to emphasize and highlight the novelty of the study in the introduction. While there are short reviews of previous works, more detailed information about the meaning of the life cycle should be provided, as you underline its importance.

**Response:**Thank you very much for the constructive feedback.We have rewritten the introduction section based on your suggestion.As follows:(The blue font represents the modified part.)

With the acceleration of industrialization, the development of society heavily depends on the combustion of fossil fuels, which leads to the emission of large amounts of greenhouse gases, resulting in the intensification of the global warming problem[1]. In the Sixth IPCC Assessment Report, it is noted that compared to the average temperature between 1850 and 1900, the surface temperature has increased by approximately 1.1 ℃. In addition, according to the Climate Sensitivity Index (CSI), it is predicted that the global average temperature will increase by approximately 3 ℃ without any change in the existing global development pattern[2]. In response, since 1990, countries worldwide have implemented energy conservation and emission reduction actions on a large scale[3], aiming to control global warming below the 2 ℃ warning line[4].

Energy consumption and carbon emissions originating from the construction industry account for 40% and 36%, respectively, of the total emissions[5]. China, as the world's largest energy consumer and carbon emitter, produces approximately 10 billion tons of carbon emissions annually[6], accounting for approximately 30% of the global emissions[7], of which the carbon emissions stemming from the construction industry account for approximately 35%–50% of China's total carbon emissions[8]. Thus, energy-saving and emission reduction efforts in the construction industry could help China quickly realize the dual-carbon goal.Moon et al.[9]found that the application of the process analysis method at the design stage only produced an error of 8% in calculating building carbon emissions. Keoleian et al.[10] evaluated the life cycle energy consumption and greenhouse gas emissions of residential buildings and found that the energy consumption of the operation and use phase exceeded 91% overall, which is the main source phase of carbon emissions. Yujie Cang[11]proposed a carbon emission calculation method based on the structure of the building as the basic unit to calculate the amount of building materials used at the materialization stage and the associated carbon emissions.

With global urbanization on the rise, limited urban surface space and increasing traffic congestion have become critical obstacles to further urban development. Metro systems, with their high-speed operations and large passenger capacity, are emerging as the optimal solution for many countries to address these challenges, becoming the preferred mode of modern urban transportation[12,13].As of early 2022, a total of 36854.2 kilometers of urban rail transit systems have been built across 541 cities in 76 countries worldwide[14].As of early 2024, mainland China has established urban rail transit systems in 59 cities, with 338 operational lines spanning a total length of 11,224.54 kilometers. Among these, metro lines constitute a significant 76.11%, covering 8,543.11 kilometers in operational length[15].Given the vast scale of underground space development, accurately assessing carbon emissions in foundation pit projects is essential due to the complex underground environment, constraints on above-ground conditions in urban areas, and the considerable construction challenges. Subway construction, in particular, often requires extensive excavation, which can lead to significant environmental impacts.

Foundation pit construction is the first step in building and rail transit projects; however, there has been limited research on the characteristics of carbon emissions specific to foundation pit engineering. Furthermore, existing carbon emission assessment models have not effectively accounted for the relevant influencing factors.This study, based on lifecycle assessment and process analysis methods, reasonably considers the impact of vehicle unladen conditions on the carbon emission calculation model for the transportation phase. It aims to accurately evaluate the carbon emissions associated with the construction of foundation pits, conduct a comparative analysis of the carbon emissions across various stages of the lifecycle of foundation pit projects, summarize the carbon emission characteristics of such projects, and provide a theoretical basis for energy-saving and emission-reduction measures in foundation pit construction.

**Comment 5:** Please create a "Methods" section where you describe the structure of your analysis and present the workflow. After that, you can describe the remaining aspects.

**Response:**Based on your suggestion, we have added a section on research methods and placed it in the second part, as shown below.

**2 Research method**

The research process of this study can be divided into four stages. First, through a literature review and data survey, it highlights the proportion of carbon emissions contributed by the construction industry globally, and emphasizes the growing necessity of analyzing the carbon emissions of foundation pit engineering as urbanization continues. With the saturation of above-ground construction and the rising trend of rail transit development, this shift further underscores the importance of such an analysis.Next, a life cycle assessment (LCA) is used to analyze the carbon emissions of foundation pit engineering. The calculation model adopts the process analysis method, which is further optimized by incorporating the fuel consumption standards for freight trucks in China. Specifically, the transportation stage model within the process analysis is adjusted to account for the discrepancy between actual and rated fuel consumption. Factors such as road conditions, traffic congestion, and temperature are introduced to refine the model. Additionally, the impact of empty-load fuel consumption on carbon emissions is reasonably considered. The optimized model can accurately assess the carbon emissions during the transportation phase.Finally, the impact of the model adjustments on carbon emissions is demonstrated through the case of the foundation pit for the 02B station at the Beijing Sub-Center Transportation Hub. The carbon emission characteristics of foundation pit engineering are then analyzed. The study concludes by identifying the existing issues and limitations, and outlining the potential directions for future research and development.

**Comment 6:**Regarding subsection 3.1: This section could be expanded with a more detailed discussion of recycling and material reuse methodologies. It would be helpful to provide examples of materials that have a significant impact on emissions, such as aluminum compared to concrete.

**Response:**Thank you for your suggestion, which will make our research more comprehensive. We have added this section by reviewing relevant materials. As shown below:

Research shows that metal waste has a higher recycling value compared to other types of waste, such as bricks and stones. For example, aluminum products, which make up 0.66% of the total weight, can contribute to a 45% reduction in carbon emissions and provide greater environmental benefits[19].The recycling rate of aluminum typically exceeds 80%, and the energy consumption for recycling aluminum is only 5% of that required to produce the same weight of primary aluminum. While the extraction and processing of aluminum release significant greenhouse gases, recycling aluminum can greatly reduce emissions. From an economic perspective, due to its high recycling rate and low energy consumption, the market potential for aluminum recycling is promising.Concrete, as a major construction waste, typically has a recycling rate of around 50%, but its recycling process is relatively complex. The production of concrete is associated with high greenhouse gas emissions, and cement production, in particular, contributes significantly to emissions. Although recycled concrete can reduce the demand for new concrete, its mechanical properties are generally slightly inferior to those of virgin concrete.

**Comment 7:**The description of individual variables, such as energy consumption (Pi) and the carbon emission factor (EF), could be further developed. For example, it would be beneficial to explain what kind of energy is being considered (fuel, electricity) and how the emission factor is determined for different fuel types.

**Response:**Based on your suggestion, we have enriched the content of section 3.3.3. As shown below:

Carbon emissions during the construction phase primarily come from the energy consumption of construction machinery, with gasoline, diesel, and electricity being the main sources. The emission factors are selected based on the emission factor database, and the specific values will be provided in the next section. The calculation model is shown in Equation 6[25].It is important to note that the actual energy consumption of construction machinery is also influenced by factors such as workload, operating methods, maintenance conditions, construction environment, and construction techniques. By optimizing these factors, fuel consumption can be effectively reduced, construction efficiency can be improved, and environmental impact can be minimized. Since these factors are influenced by the specific conditions of the construction site, the influencing coefficients are determined by the construction company.

**Comment 8:**Regarding the model in subsection 3.3: Unlike other phases analyzed in previous sections, this model does not account for correction factors, such as machine operating conditions, climate factors, or variability in operational efficiency depending on the project. Including these elements could make the model more comprehensive.

**Response:**Thank you for your kind suggestion.We have taken into account the impact of these factors you mentioned in the model, making the analysis more comprehensive.As shown below:

 (6)

where *E*_c_ denotes the total carbon emissions (t) during the construction phase; n denotes the types of machinery needed for construction; *S*_i_ is the number of shifts needed for the ith type of construction machinery; *P*_i_ is the amount of energy consumed per shift by the ith type of construction machinery; *EF*_i_ is the energy carbon emission factor；and *K_S_* is correction factors for other influencing factors on energy consumption of construction machinery.

**Comment 9:**Please improve the size and quality of the figures.

**Response:**We are sorry for our careless mistakes.We have made modifications to the quality of the figures.
